# Validation and Implementation of Clinical Laboratory Improvements Act-Compliant Whole-Genome Sequencing in the Public Health Microbiology Laboratory

**DOI:** 10.1128/JCM.00361-17

**Published:** 2017-07-25

**Authors:** Varvara K. Kozyreva, Chau-Linda Truong, Alexander L. Greninger, John Crandall, Rituparna Mukhopadhyay, Vishnu Chaturvedi

**Affiliations:** Microbial Diseases Laboratory, California Department of Public Health, Richmond, California, USA; University of Iowa College of Medicine

**Keywords:** bacteria, whole-genome sequencing, performance specifications, laboratory-developed test, quality management, validation, CLIA, public health, bioinformatics pipeline, WGS

## Abstract

Public health microbiology laboratories (PHLs) are on the cusp of unprecedented improvements in pathogen identification, antibiotic resistance detection, and outbreak investigation by using whole-genome sequencing (WGS). However, considerable challenges remain due to the lack of common standards. Here, we describe the validation of WGS on the Illumina platform for routine use in PHLs according to Clinical Laboratory Improvements Act (CLIA) guidelines for laboratory-developed tests (LDTs). We developed a validation panel comprising 10 Enterobacteriaceae isolates, 5 Gram-positive cocci, 5 Gram-negative nonfermenting species, 9 Mycobacterium tuberculosis isolates, and 5 miscellaneous bacteria. The genome coverage range was 15.71× to 216.4× (average, 79.72×; median, 71.55×); the limit of detection (LOD) for single nucleotide polymorphisms (SNPs) was 60×. The accuracy, reproducibility, and repeatability of base calling were >99.9%. The accuracy of phylogenetic analysis was 100%. The specificity and sensitivity inferred from multilocus sequence typing (MLST) and genome-wide SNP-based phylogenetic assays were 100%. The following objectives were accomplished: (i) the establishment of the performance specifications for WGS applications in PHLs according to CLIA guidelines, (ii) the development of quality assurance and quality control measures, (iii) the development of a reporting format for end users with or without WGS expertise, (iv) the availability of a validation set of microorganisms, and (v) the creation of a modular template for the validation of WGS processes in PHLs. The validation panel, sequencing analytics, and raw sequences could facilitate multilaboratory comparisons of WGS data. Additionally, the WGS performance specifications and modular template are adaptable for the validation of other platforms and reagent kits.

## INTRODUCTION

Clinical microbiology laboratories and public health microbiology laboratories (PHLs) are undergoing transformative changes with the adoption of whole-genome sequencing (WGS) (1, 2). For several years, leading laboratories have reported proof-of-concept studies on WGS-enabled advances in the identification of pathogens, antibiotic resistance (ABR) detection, and disease outbreak investigations (3–6). Technologies also referred to as next-generation sequencing (NGS) have yielded more detailed information about the microbial features than was possible by using a combination of other laboratory approaches. Further developments of WGS platforms allowed remarkable in-depth inquiry of pathogenic genomes for the discovery of genetic variants and genome rearrangements that could have been missed by using other DNA methods (3, 7, 8). Enhanced investigations of disease outbreaks have led to a new understanding of routes of transmission of infectious agents (9–11). WGS-enabled metagenomics and microbiome discoveries have revealed a new appreciation for the role of microbes in health and disease (12–15). The innovations are continuing at such an unprecedented pace that WGS is expected to become an alternative to culture-dependent approaches in clinical and public health microbiology laboratories (16–18).

Notwithstanding its promises, several challenges remain for the adoption of WGS in microbiology laboratories (19–22). The accelerated obsolescence of sequencing platforms presents several obstacles in bridging the gap between research and routine diagnostics, including standardization efforts (23). The downstream bioinformatics pipelines are also unique challenges for microbiology laboratories regarding both infrastructure and skilled operators (24–27). Overall, WGS “wet-bench” and “dry-bench” workflows represent integrated processes, which are not easily amenable to the traditional quality metrics used by microbiology laboratories (27–29). The capital investments and recurring costs of WGS for clinical laboratories, although rapidly declining, remain relatively high to allow multilaboratory comparisons for the standardization of analytical parameters. Finally, regulatory agencies have not yet proposed standard WGS guidelines for clinical microbiology (30), and external proficiency testing (PT) programs for clinical and public health microbiology laboratories are still in development (31, 32).

There are a few notable developments toward the standardization and validation of next-generation sequencing in clinical laboratories. The U.S. Centers for Disease Control and Prevention (CDC) sponsored the Next-Generation Sequencing: Standardization of Clinical Testing (Nex-StoCT) workgroup to propose quality laboratory practices for the detection of DNA sequence variations associated with heritable human disorders (33, 34). This workgroup developed principles and guidelines for test validation, quality control, proficiency testing, and reference materials. Although not focused on infectious diseases, these guidelines provide a valuable roadmap for the implementation of WGS in clinical microbiology and public health laboratories. The College of American Pathologists (CAP) reported 18 requirements in an accreditation checklist for next-generation sequencing analytic (wet-bench) and bioinformatics (dry-bench) processes as part of its molecular pathology checklist (30). These “foundational” accreditation requirements were designed to be broadly applicable to the testing of inheritable disorders, molecular oncology, and infectious diseases. Along the same lines, the feasibility of *in silico* proficiency testing has been demonstrated for NGS (35). Recently, high accuracy and reproducibility were shown for WGS-based microbial strain typing performed in a ring trial study involving five laboratories; the results suggested that a proficiency testing program for WGS is feasible in clinical microbiology laboratories (36). The Clinical and Laboratory Standards Institute (CLSI) updated its guidelines for nucleic acid sequencing methods in diagnostic laboratory medicine with considerations specific to the application of next-generation sequencing in microbiology (37). Thus, a broad technical framework is now available to design WGS validation protocols that will be most relevant for clinical and public health laboratories. Our aims for the present study were to establish performance metrics for the typical workflow in public health microbiology laboratories, design modular templates for the validation of different platforms and chemistries, finalize a user-friendly report format, and identify a set of bacterial pathogens that could be used for WGS validation and performance assessments.

## RESULTS

### Accuracy of WGS.

**FIG 1:**
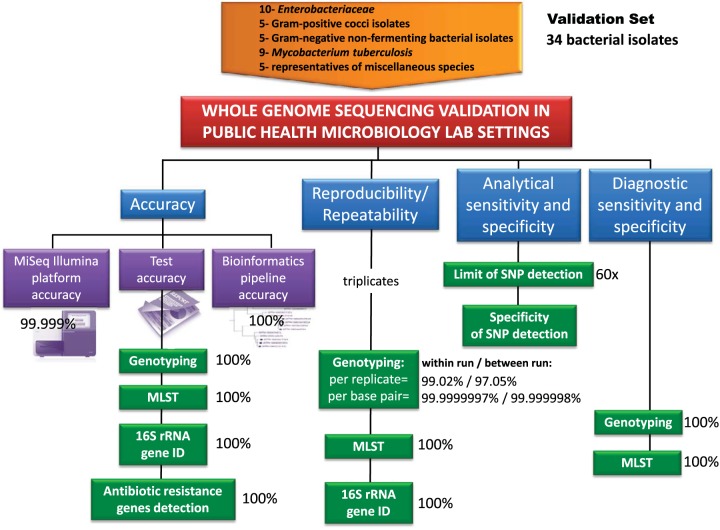
Summary of WGS validation. The estimated performance parameters are shown in blue boxes. The components of WGS accuracy determined in this study are shown in purple boxes. The WGS assays evaluated in order to deduce the corresponding performance parameters are shown in green boxes. Percentages alongside the boxes represent values measured during this validation for the corresponding parameters.

A number of Clinical Laboratory Improvements Act (CLIA)-required performance parameters were adopted, with modifications for the validation of WGS (Table 1). The modular validation template and a summary are presented in Fig. 1. The quality assurance (QA) and quality control (QC) measures are described in detail later in Results.

**TABLE 1:** Performance characteristics, definitions, and formulas used for validation^*a*^

Performance characteristic for WGS applications	Definition of performance characteristic for WGS applications	Formula used for calculation	Assay-specific definition	Result of assay used for validation of parameter			
				hqSNP-based genotyping	MLST	16S	Antibiotic resistance gene detection
Accuracy	Degree of agreement between the nucleic acid sequences derived from the assay (measured value) and those from a reference sequence (true value)						
Accuracy of platform	Accuracy of base calling against the reference sequence; the accuracy of the platform was established as the agreement between base calling made by the MiSeq sequencer (measured value) and the NCBI/CDC reference sequence (true value)	% agreement with reference = [(covered genome length) − (total no. of SNPs differing from reference)]/(covered genome length) × 100	Accuracy of the platform	99.999378%			
Accuracy of assay	Accuracy of assay is determined as an agreement of the assay result for validation sequences generated by the PHL with the assay result for reference sequences of the same strains	Accuracy = (no. of correct results)/(total no. of results) × 100	Definition of correct result	Congruence of phylogenetic trees built using reference sequences and validation sequences	Detection and correct identification of each of the MLST alleles	ID of the 16S rRNA sequence of the validation sample matches the ID of the 16S rRNA sequence of the reference sequence	Presence of ABR genes characteristic of the reference strain, absence of any other ABR genes
			Single test unit	Individual sample clustering	Allele	16S rRNA ID result	Antibiotic resistance gene
			Accuracy of assay	100%	100%	100%	100%
Accuracy of bioinformatics pipeline	Agreement of the clustering suggested by previous investigators with the clustering achieved by analysis using PHL validation bioinformatics pipeline	% agreement = (no. of outbreak isolates clustered correctly in validation tree)/(total no. of outbreak isolates clustered together in the study tree) × 100	Accuracy of bioinformatics pipeline	100%	NA	NA	NA
Precision	Degree to which repeated sequence analyses give the same result repeatably (within-run precision) and reproducibly (between-run precision)						
Repeatability	Repeatability was established by sequencing the same samples multiple times under the same conditions and evaluating the concordance of the assay results and performance	Repeatability = (no. of within-run replicates in agreement)/(total no. of tests performed for within-run replicates) × 100	Definition of correct result	Repeatability of single nucleotide variant detection	Repeatability of allele detection	Repeatability of 16S ID	NA
			Single test unit	SNP (precision per replicate), SNP (precision per genome size)	Allele	16S rRNA ID	NA
			Repeatability	99.02%, 99.9999997%	100%	100%	NA
Reproducibility	Reproducibility was assessed as the consistency of the assay results and performance characteristics for the same sample sequenced under different conditions, such as between different runs, operators, and sample preparations	Reproducibility = (no. of between-run replicates in agreement)/(total no. of tests performed for between-run replicates) × 100	Definition of correct result	Reproducibility of single nucleotide variant detection	Reproducibility of allele detection	Reproducibility of 16S ID	NA
			Single test unit	SNP (precision per replicate), SNP (precision per base pair)	Allele	16S rRNA ID	NA
			Reproducibility	97.05%, 99.999998%	100%	100%	NA
Analytical sensitivity (LOD)	Minimum coverage that allows accurate SNP detection (LOD_SNP_)	NA	LOD_SNP_	60×			NA
Analytical specificity (interference)	Ability of an assay to detect only the intended target in the presence of potentially cross-reacting nucleotide sequences	NA	Interference/cross-reactivity	Cross-reactivity and interference from contaminating sequencing reads are possible			NA
Diagnostic sensitivity	Likelihood that a WGS assay will detect sequence variation when present within the analyzed genomic region (this value reflects the false-negative rate of the assay)	Diagnostic sensitivity = TP/(TP + FN) × 100	Definition of true-positive result	Clustering of related samples (no. of validation samples with clustering results matching the reference)	No. of correctly identified alleles	NA	NA
			Definition of false-negative result	No. of validation samples that clustered together with samples genetically distant according to the reference tree	No. of unidentified or misidentified alleles in validation samples	NA	NA
			Single test unit	Individual sample clustering	Allele	NA	NA
			Diagnostic sensitivity	100%	100%	NA	NA
Diagnostic specificity	Probability that a WGS assay will not detect sequence variations when none are present within the analyzed genomic region (this value reflects an assay's false-positive rate)	Diagnostic specificity = TN/(TN + FP) × 100	Definition of true-negative result	No clustering between unrelated samples (no. of validation samples with clustering results matching the reference)	No. of unidentified alleles in negative-control samples	NA	NA
			Definition of false-positive result	No. of validation samples that failed to cluster together with samples genetically similar according to the reference tree	No. of identified alleles in negative-control samples	NA	NA
			Single test unit	Individual sample clustering	Allele	NA	NA
			Diagnostic specificity	100%	100%	NA	NA
Reportable range	Region of the genome in which a sequence of an acceptable quality can be derived by the laboratory assay	NA		Genome-wide hqSNPs	Housekeeping genes in MLST scheme	16S rRNA gene	Genes in ResFinder database

a See details in Document S1 in the supplemental material. Abbreviations: TP, true-positive results; TN, true-negative results; FP, false-positive results; FN, false-negative results; LOD, limit of detection; LOD_SNP_, limit of SNP detection; ID, identification; NA, the parameter was not defined for the given assay.

The accuracy of WGS was divided into three components: platform accuracy, assay accuracy, and bioinformatics pipeline accuracy.

### Platform accuracy.

Platform accuracy was assessed as the accuracy of the identification of individual base pairs (“base calling”) in the bacterial genome. The accuracy of the platform was established by determining the proximity of agreement between base callings made by the MiSeq sequencer (measured value) and the NCBI/CDC reference sequence (the true value). We determined the accuracy of the MiSeq Illumina platform by mapping generated reads to the corresponding reference sequence and identifying single nucleotide polymorphisms (SNPs) throughout the genome. A few validation samples differed from the reference genome by several SNPs. However, 99% (324 out of 327) of these SNPs were reproducible among all five replicates sequenced for each sample. Since amplification and sequencing errors were random between different library preparations, it was unlikely that the same erroneous SNP would occur in all five replicates. Therefore, we concluded that these discrepancies were not caused by sequencing errors but most likely were the result of the accumulation of mutations in the reference strains or previous sequencing mistakes in the reference sequence. Sanger sequencing confirmed the SNPs found in WGS results for the selected isolates (see Appendix 14 in Document S1 in the supplemental material). However, in several instances, it was impossible to design specific primers within reach of Sanger sequencing read lengths because SNPs were found in the repeat regions. In one case, the results of Sanger sequencing matched the results of mapping, but upon comparison with *de novo* assemblies, the same sequence was found in two variants, with and without the SNP detected by mapping. This highlighted the limitation of the short-read sequencing technology and the underlying caution that must be exercised in the interpretation of the SNPs called in the repeat regions of the genomes. In both cases, whether we took into account all SNPs detected between validation and reference sequences or only those SNPs that did not appear in all of the replicates (true sequencing errors), we observed >99.999% agreement of the generated whole-genome sequences with the reference sequences for each tested sample.

### Assay accuracy.

Assay accuracy was defined as the agreement of the assay results for the validation sequences with the assay results for the reference sequences of the same strains. Four applications of WGS were used to validate the accuracy of the assay: an *in silico* multilocus sequence typing (MLST) assay, a 16S rRNA gene species identification assay, an assay for the detection of ABR genes, and a genotyping assay using high-quality SNPs (hqSNPs).

The definition for a correct result for MLST corresponds to the correct identification of each of the MLST alleles in the validation sequence as well as in the reference sequence analyzed in parallel. For all validation samples, each of the sequences of the seven housekeeping genes used in the typing scheme (or six genes for Aeromonas hydrophila) were identified correctly, resulting in 100% allele identification accuracy.

For ABR gene detection, the sequences of the ATCC susceptibility control strains generated during validation were analyzed by using ResFinder. Data analysis using ResFinder implies comparison of the sequence against each entry in the ResFinder database, which at the moment of validation contained sequences of 1,719 antibiotic resistance genes, resulting in a total of 1,719 tests performed for each validation sample. The results of ResFinder detection were compared to the resistance genes known to be present in the ATCC strains. Two of the sequenced isolates contained one resistance gene each, which were also detected by ResFinder, with no additional genes being identified. No resistance genes were detected by ResFinder in the samples suggested to be negative susceptibility controls and lacking resistance determinants. Thus, the accuracy of the assay for ABR gene detection was 100% (see the section on detection of resistance genes in ATCC strains using ResFinder in Document S1 in the supplemental material). Moreover, 13 reference sequences of Gram-negative (7 sequences) and Gram-positive (6 sequences) bacterial isolates with various resistance genes from the U.S. Food and Drug Administration (FDA)-CDC Antimicrobial Resistance Isolate (AR) Bank were used for *in silico* testing of ABR gene detection accuracy using ResFinder. The resistance genes in the AR Isolate Bank isolates were previously detected by the CDC using PCR-based methods (for primary resistance types) and by ResFinder (database last updated 2 June 2016). Our analysis of reference sequences with ResFinder (last updated 17 February 2017) confirmed the presence of all genes that were detected by PCR-based methods (*n* = 8), resulting in 100% accuracy. At the moment of analysis, ResFinder did not have the ability to detect the truncation of porin genes; therefore, porin-related resistance mechanisms mentioned in the CDC database could not be detected with ResFinder. Upon ResFinder analysis at the CDC, the isolates harbored a total of 83 resistance genes (representative of 57 different alleles). The detection of the genes by the CDC using ResFinder was replicated in this study, with few discrepancies (see the section on *in silico* detection of resistance genes in FDA-CDC isolates using ResFinder and Appendix 15 in Document S1 in the supplemental material). In the case of Gram-negative bacteria, all discrepancies were represented by additional genes detected in comparison with CDC ResFinder results, and these discrepancies were most likely caused by the database update; e.g., the *aph(3′)-Ia* gene was not on the list of the genes detected with ResFinder by the CDC but was detected in the same sequences using ResFinder in this study. This particular gene discrepancy could be explained by the use of a later version of ResFinder, which was updated with additional aminoglycoside resistance genes. In the case of Gram-positive bacteria, several discrepancies were also caused by the additional genes detected in this study, but two genes that were present in the CDC results were missing from our results. These false-negative genes were detected by us but possessed <99% identity or an incomplete sequence and therefore were excluded from the final result. Nevertheless, the agreement between CDC ResFinder results and our ResFinder results was 99.97%.

For the 16S rRNA identification assay, variations in one gene were detected, so the species identification results as a whole (e.g., “Escherichia coli”) were considered a single test. The identity of the 16S rRNA sequences extracted from validation samples showed 100% matches with the 16S rRNA sequences extracted from the reference sequence.

To assess the accuracy of the genome-wide hqSNP-based genotyping assay, phylogenetic trees were built by using reference sequences and validation sequences, and the resulting trees were compared. For better comparison, we used at least five strains of the same species in the phylogenetic tree. The accuracy of the genotyping assay was determined by using two approaches: (i) topological similarity between the reference tree and the validation tree using Compare2Trees software and (ii) comparison of the clustering patterns of the validation tree and the reference tree. Therefore, in addition to the detection of SNP differences between the validation sequences and reference sequences, their effect on the final tree topology was also estimated to determine the accuracy of the genotyping assay. Phylogenetic trees were generated for five bacterial species (Escherichia coli, Salmonella enterica, Staphylococcus aureus, Enterococcus faecalis, and Stenotrophomonas maltophilia). All five validation trees had matching clustering patterns and 100% topological similarity with the corresponding reference trees (see Table S2 in the supplemental material).

### Accuracy of the bioinformatics pipeline.

**FIG 2:**
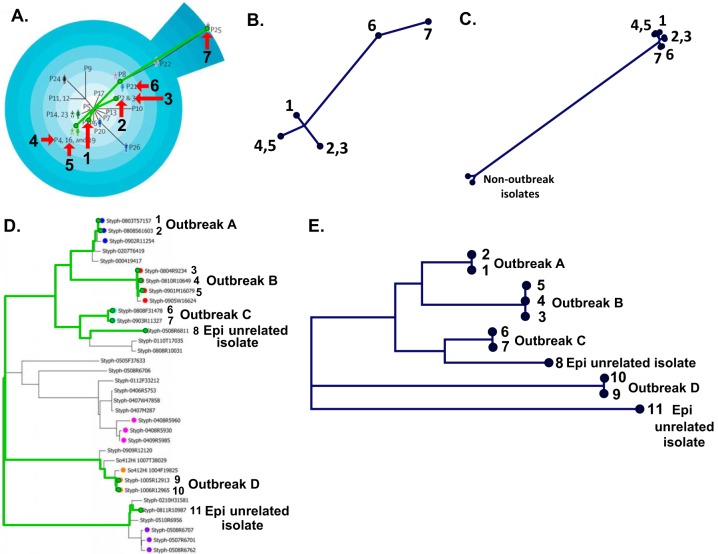
Bioinformatics pipeline validation with outbreak isolates from two previously published studies. (A) Phylogenetic tree of outbreak isolates reported in the “MRSA study” by Harris et al. (38). The isolates from the MRSA study that were picked for validation are indicated by arrows and numbers assigned for purposes of validation (1 to 7). (B) Phylogenetic tree validation using the samples from the MRSA study and the validation bioinformatics pipeline. The same isolates in the original tree and the validation tree are marked with the same numbers. (C) Comparison of the group of related isolates (isolates 1 to 7) from the MRSA study with epidemiologically unrelated isolates from the same study using the validation bioinformatics pipeline. (D) Phylogenetic tree combining epidemiologically related and nonrelated isolates reported in the “Salmonella study” by Leekitcharoenphon et al. (39). The isolates from the Salmonella study that were picked for validation are marked with green node circles and have the numbers 1 to 11 assigned for purposes of validation. Epi, epidemiologically. (E) Validation phylogenetic tree generated for the samples from the Salmonella study using the in-house bioinformatics pipeline. The same isolates in the tree from the Salmonella study and the validation tree are marked with the same numbers.

The accuracy of the bioinformatics pipeline for hqSNP-based genotyping by itself was assessed by the recapitulation of previously reported results using WGS raw reads of bacterial isolates included in two previous studies (Table 2). This was an additional assessment of the accuracy of the genotyping pipeline apart from the accuracy of base calling from in-house sequencing of the isolates. Phylogenetic analyses of isolates associated with outbreaks caused by a Gram-positive pathogen in the first study (MRSA [methicillin-resistant Staphylococcus aureus] study) (38) and a Gram-negative pathogen in the second study (Salmonella study) (39) were performed for the validation of the bioinformatics pipeline (Fig. 2). The clustering of the validation tree completely replicated the clustering of the tree from the MRSA study (38) (Fig. 2A and B); e.g., isolates 4 and 5 were identical and clustered together according to the MRSA study, and the same results were shown in the validation tree, with isolates 4 and 5 sharing the same node. All conclusions with regard to the genetic relatedness of the isolates that can be drawn from the tree reported in the MRSA study could also be made for the analysis of the corresponding validation tree. The group of outbreak isolates from the MRSA study was compared with epidemiologically unrelated isolates suggested by the same study (no tree was available from that report). Phylogenetic analysis using the in-house bioinformatics pipeline showed that epidemiologically unrelated isolates did not cluster with the group of outbreak isolates and appeared to be genetically distant (Fig. 2C). Thus, the resulting phylogenetic tree produced by our bioinformatics pipeline showed complete concordance with the epidemiological data.

**TABLE 2:** Summary of data from previous studies used for validation of the bioinformatics pipeline

Study parameter	Value for study	
	MRSA study^*a*^	Salmonella study^*b*^
Microorganism	Methicillin-resistant Staphylococcus aureus	Salmonella enterica serovar Typhimurium
Source of isolates	Human	Human
No. of isolates analyzed	7 outbreak isolates (1 outbreak cluster) + 2 epidemiologically unrelated isolates	9 outbreak isolates (4 outbreak clusters) + 2 epidemiologically unrelated isolates
Type of outbreak	Hospital-associated outbreak	Foodborne outbreaks
Samples used for validation	P1, P2, P3, P4, P16, P21, and P25; an isolate identified by infectious control investigation as belonging to nonoutbreak ST1; a MRSA isolate identified by searching a microbiology database as belonging to nonoutbreak ST772	0803T57157, 0808S61603, 0808F31478, 0903R11327, 0811R10987, 0804R9234, 0810R10649, 0901M16079, 0110T17035, 1005R12913, and 1006R12965
GenBank accession no. of corresponding samples	ERR070045, ERR070042, ERR070043, ERR070044, ERR124429, ERR124433, ERR128708, ERR070041, ERR072248	ERR277220, ERR277226, ERR277223, ERR277222, ERR277224, ERR277221, ERR277227, ERR277228, ERR277203, ERR277233, ERR277234
No. of clusters in the study tree	1	4
No. of clusters in the validation tree	1	4
No. of outbreak isolates in each cluster in the study tree	7 for cluster 1	2 for cluster 1, 3 for cluster 2, 2 for cluster 3, and 2 for cluster 4
No. of outbreak isolates in each cluster in the validation tree	7 for cluster 1	2 for cluster 1, 3 for cluster 2, 2 for cluster 3, and 2 for cluster 4
No. of epidemiologically unrelated isolates in the set	2	2
No. of epidemiologically unrelated isolates that clustered with outbreak isolates	0	0
% agreement {[(no. of outbreak isolates clustered correctly in the validation tree) × 100]/(total no. of outbreak isolates that clustered together in the study tree)}	(7 × 100/7) = 100	(9 × 100/9) = 100

a See reference 38. ST1, sequence type 1; ST772, sequence type 772.

b See reference 39.

From the Salmonella study (39), we selected nine isolates that were representative of four independent outbreaks and two isolates that were epidemiologically unrelated controls (Fig. 2D). The clustering of the validation tree was identical to the clustering of the tree from the Salmonella study. For example, isolates 6 and 7 were part of the same outbreak, while isolate 8 was an epidemiologically unrelated control used in that study. In agreement with the epidemiological data and the tree from the Salmonella study, the validation tree showed that isolates 6 and 7 clustered together but not with isolate 8 (Fig. 2E). All observations about the genetic relatedness of the isolates drawn from the tree from the Salmonella study could be replicated from the analysis of the validation tree. In summary, based on *in silico* analysis of data from both studies, 100% accuracy of our bioinformatics pipeline was established for phylogenetic analysis.

### WGS repeatability and reproducibility.

Repeatability (within-run precision) was established as the concordance of the assay results and quality metrics obtained for a sample tested multiple times within the same sequencing run. Reproducibility (between-run precision) was assessed as the consistency of the assay results and quality metrics for the same sample sequenced on different occasions. Thirty-four validation samples each were sequenced three times in the same sequencing run (for repeatability) and three times in different runs (for reproducibility). For within-run replicates, one DNA extract was used, but independent library preparations were done, with the final samples being included in a single sequencing run. Therefore, for each sample, the numbers of within-run replicates and between-run replicates were 3 each, and the total numbers of repeated results were 5. All quality parameters (depth of coverage, uniformity of coverage, and accuracy of base calling [*Q* score], etc.) did not change significantly for within- and between-run replicates, as determined by a two-tailed *t* test. For the quality values for all sequenced samples, see the section on inter- and intra-assay agreement and Appendix 8 in Document S1 in the supplemental material.

The reproducibility and repeatability of the WGS assay were evaluated with two methods: evaluation of base calling reproducibility and repeatability per replicate and evaluation of base calling reproducibility and repeatability relative to genome size. All validation samples except C50 yielded identical whole-genome sequences for all three within-run replicates. One out of three within-run replicates for isolate C50 (Pseudomonas aeruginosa ATCC 27853) had one SNP difference from other within-run replicates (Table S3). The repeatability per replicate was 99.02%. Three validation samples had one out of three between-run replicates that differed from the other two between-run replicates. Sample C47 (Staphylococcus epidermidis ATCC 12228) had one between-run replicate with two SNPs that differed from the other replicates. Samples C49 (Streptococcus pneumoniae ATCC 6305) and C55 (Escherichia coli ATCC 25922) each had one between-run replicate that differed from other replicated sequences by one SNP. The reproducibility of base calling per replicate was 97.05%. Both the reproducibility and repeatability of base calling relative to the genome size (in relation to the total number of base pairs of the covered genome size) were >99.9999%.

We also estimated the reproducibility and repeatability of MLST and 16S rRNA identification assays. For MLST, a total of 441 alleles were analyzed for within- or between-run replicates. Each single allele in all validation samples was identified consistently among within- and between-run replicates. Within- and between-run replicates had repeatable/reproducible sequences of 16S rRNA genes and resulted in the consistent identification of the species. The reproducibility and repeatability of allele detection and species identification within and between runs were 100%.

### WGS sensitivity and specificity.

The sensitivity of WGS was assessed as (i) analytical sensitivity (minimum coverage that allows accurate SNP detection) and (ii) diagnostic sensitivity (the likelihood that a WGS assay will detect sequence variation when it is present) (this value reflects the false-negative rate of the assay).

The specificity of WGS was determined as (i) analytical specificity (the ability of an assay to detect only the intended target in the presence of potentially cross-reacting nucleotide sequences) and (ii) diagnostic specificity (the probability that a WGS assay will not detect sequence variations when none are present) (this value reflects the false-positive rate of the assay).

### Analytical sensitivity.

The limit of detection (LOD) is traditionally defined as “the lowest actual concentration of an analyte in a specimen that can be consistently detected … with acceptable precision” (40). The LOD in this sense is not applicable to WGS, which utilizes pure bacterial cultures as starting material; the amount of the analyte (genomic DNA) that is added to the reaction mixture is strictly standardized, and the DNA concentration of each sample is measured by a fluorometric method before each assay. In our workflow, we established the minimum amount of the starting DNA input to be 1 ng (at a concentration of 0.2 ng/μl) and did not process DNA extracts with concentrations of <1 ng/μl. Instead, we determined the LOD of SNP detection (LOD_SNP_) by establishing the lowest coverage that allows accurate SNP calling. The LOD_SNP_ was estimated by modeling different mapping coverages and estimating the number of SNPs called at each of the coverage values. Nine samples representative of different species were *in silico* downsampled to coverages of 60×, 50×, 40×, 30×, 20×, 15×, 10×, and 5×. The LOD_SNP_ was established to be 60×, as it was the lowest coverage which yielded accurate SNP detection for all of the samples (see Table S4 and the section on analytical sensitivity of SNP detection in Document S1 in the supplemental material).

### Analytical specificity.

Analytical specificity is referred to as the “ability of an assay to detect only the intended target and that quantification of the target is not affected by cross-reactivity from related or potentially interfering nucleic acids or specimen-related conditions” (40). Since our WGS pipeline is not intended for clinical specimens directly, the conditions interfering with fragmentation, amplification, and sequencing processes are minimized and are monitored via multiple QC steps, including DNA and library purity and concentration measurements. However, interference from contaminating nucleotide sequences is much more consequential. Analytical specificity (interference) was determined by creating sequencing files containing a mixture of the reads from two different samples *in silico*, thus mimicking contamination and demonstrating its effect on mapping metrics (percentage of reads mapped, percentage of the reference sequence covered, etc.) and SNP detection (see Table S5 and the section on the analytical specificity of SNP detection in Document S1 in the supplemental material). As expected, contaminating reads led to a decrease in the percentage of mapped reads and an increase in the portion of unmapped reads. The percentage of reads in pairs decreased for samples containing contaminating reads. Modeled contamination with E. coli C1 and M. tuberculosis C57 sequencing reads did not cause any change in called SNPs. Contamination with any of the other reads led to additional SNPs being called both between the compared samples and with the reference sequence. In the sample contaminated with S. enterica C75, in addition to nonspecific SNPs, one of the SNPs detected previously was missing. The bioinformatics pipeline had a certain tolerance of contaminating reads depending on the nature of the contamination.

Diagnostic sensitivity and specificity were estimated for genotyping and MLST assays.

### Diagnostic sensitivity and specificity of genotyping.

To estimate the diagnostic sensitivity and specificity of WGS-based genotyping, the hqSNP phylogenetic trees generated from the validation sequences were compared to the trees generated from the reference sequences for the same strain. In the case of whole-genome sequence genotyping, the true sequence variation (SNP), which was not detected, represents a false-negative result. Changes introduced into the DNA sequence during library preparation/sequencing or data analysis errors could result in false-positive sequence variations. All generated validation trees repeated clustering and had 100% topological similarity to the corresponding reference trees, indicating the absence of false-negative or false-positive results of the genotyping assay. The hqSNP-based genotyping assay was 100% sensitive and specific.

### Diagnostic sensitivity and specificity of MLST.

The sequence types of validation sequences and reference sequences were determined by using organism-specific MLST databases. For MLST, the number of true-positive results corresponds to the number of alleles correctly identified in the validation samples. For true-negative results, we performed a comparison of validation sequences against MLST databases for unmatched species, e.g., a search of alleles for the Escherichia coli C1 validation sample against the MLST database for Salmonella enterica. In the latter case, the MLST assay was expected not to identify any alleles. All alleles in the positive validation samples were identified correctly. None of the alleles were identified in the negative controls. Both the diagnostic sensitivity and analytical specificity of the *in silico* MLST assay were 100%.

### Reportable range for WGS.

The following information about the sequenced genome was collected for the reportable range: genome-wide hqSNPs, housekeeping genes used in MLST schemes, 16S rRNA genes, and antibiotic resistance genes included in the ResFinder database. A reporting language was developed to assist in the interpretation of results by an end user with or without specific WGS knowledge. The appropriate template and examples are provided in Document S1 in the supplemental material.

### Quality assurance and quality control for WGS.

**FIG 3:**
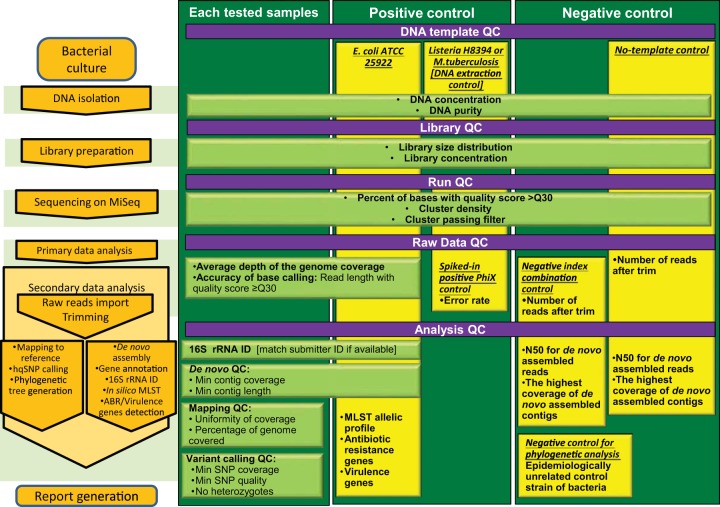
WGS quality control scheme. The preanalytical, analytical, and postanalytical steps of WGS are shown on the left in orange boxes. The QC metrics that are being evaluated at each step are presented in light green boxes. Three vertical blocks represent the types of samples to which major QC metrics are being applied: test samples, positive controls, and negative controls. The overlap between the boxes designating QC metrics and the vertical blocks shows which QC metrics are applicable to which of the three types of samples.

QA and QC measures were developed to ensure high quality and consistency of routine testing using the MiSeq Illumina platform. QC was performed during the preanalytical (DNA isolation and library preparation), analytical (quality metrics of the sequencing run), and postanalytical (data analysis) steps of WGS. Five QC checkpoints were implemented throughout the WGS procedure: DNA template QC, library QC, sequencing run QC, raw data QC, and data analysis QC. The quality parameters evaluated at each of these QC checkpoints are summarized in Fig. 3. The QA and QC manual established for WGS applications is presented in Document S1 in the supplemental material. Based on the preliminary quality thresholds, all runs passed from the first attempt, and none of the samples had to be resequenced. The final quality thresholds were set at the lower border of quality values, which still allowed the generation of accurate and reproducible assay results (phylogenetic analysis, MLST, 16S identification, or ABR detection). The WGS quality cutoff values are summarized in Table S6 in the supplemental material. We determined the optimal depth of coverage to be ≥60× based on the accuracy of SNP detection at various simulated genome coverages. Table S6 does not reflect the optimal data quality parameters but merely the thresholds below which data will be rejected and the sample will have to be resequenced.

We employed two levels of positive and negative controls. First, as an internal positive control, we used spiked-in PhiX Control (Illumina Inc., San Diego, CA, USA) and evaluated its sequencing error rate. As an internal negative control, at the demultiplexing step, we called index combinations, which did not correspond to any samples in the current sequencing run but were used in the previous run. This negative-index-combination control allowed us to capture carryover contamination with the library fragments generated in the previous runs. Second, as an external positive control, we included E. coli strain ATCC 25922 processed from the DNA extraction step all the way to the data analysis step. As an external negative control, a no-template control was processed through all WGS steps starting with DNA extraction. Additional positive controls representing one Gram-positive strain and one M. tuberculosis strain were introduced solely for the DNA extraction step in cases when the corresponding types of samples were processed (to control for differences in the DNA extraction protocols). The quality cutoff values for the controls can be found in Table S6.

### Validation summary.

Analytical sensitivity (LOD of SNP detection) was established at a 60× depth of genome coverage. Analytical specificity in the presence of interfering sequencing reads was demonstrated for different types of contamination. The WGS assay was shown to have >99.9% accuracy, >99.9% reproducibility/repeatability, and 100% diagnostic specificity and sensitivity. These parameters met the CLIA requirements for laboratory-developed tests (LDTs).

## DISCUSSION

This study established the workflow and reference materials for the validation of WGS for routine use in PHLs according to CLIA guidelines for LDTs. The validation panel, sequencing analytics, and raw sequences generated during this study could serve as resources for future multilaboratory comparisons of WGS data. Additionally, the WGS performance specifications and modular validation template developed in this study could be easily adopted for the validation of other platforms and reagent kits. These results could strengthen the concept of unified laboratory standards for WGS enunciated by some professional organizations, including the Global Microbial Identifier (GMI) initiative (30, 31, 33, 41). A few other groups have also highlighted the challenges and solutions for the implementation of WGS in clinical and public health microbiology laboratories (21, 42).

Using a combination of reference strains and corresponding publicly available genomes, we devised a framework of “best practices” for the quality management of the integrated “wet-lab” and “dry-lab” WGS workflows (“pipeline”). The importance of reference materials for the validation and QC of wet- and dry-lab WGS processes was noted previously (28, 31, 33). Unlike human genomics (43), there is no well-established resource of reference materials for the validation of WGS in public health microbiology laboratories. The main challenge for creating a customized validation set is a lack of reference materials, in other words, strains that can be easily acquired by PHLs and that have high-quality, well-characterized reference genomes available. While the use of genomic sequences of ATCC strains from the NCBI is an option, it is far from being perfect. The genome sequences available from public databases are generated by using different methods, chemistries, and platforms, which might yield different error rates; therefore, deposited sequences are not guaranteed to be free of such errors. With the ongoing development of new sequencing technologies and improvements in the quality of sequences, it is likely that the genomes sequenced with old methods might appear less accurate than the sequences generated by the laboratory during validation. Additionally, there is the possibility of mutation accumulation in control strains, e.g., ATCC cultures, after many rounds of subculture in different laboratories. Overall, there is no gold standard available yet for use as reference material for the validation of WGS for pathogenic bacteria. Nevertheless, NCBI, ENA, and similar public genome depositories remain the best resources for the genomic sequences of control strains that could be used for validation, such as the FDA Database for Regulatory-Grade Microbial Sequences (FDA-ARGOS) (https://www.ncbi.nlm.nih.gov/bioproject/231221). In the future, it would be helpful to have a network/agency/bank that could distribute panels of sequenced and curated isolates with genomic sequences available online for WGS validation. In the absence of such a resource, we developed a validation set of microorganisms, which can be used for future validations of WGS platforms. Bacterial genomes vary in size, GC content, the abundance of repeat regions, and other properties, which affect WGS results. We created a validation set that reflects the diversity of microorganisms with various genome sizes and GC contents that are routinely sequenced by PHLs. Different species of Gram-positive and Gram-negative microorganisms and M. tuberculosis were included to account for the differences in DNA extraction procedures as well.

Samples were validated based on four core elements reflected in the formal assay report: 16S rRNA-based species identification, *in silico* MLST, hqSNP phylogenetic analysis, and the presence of ABR determinants. Specific WGS assays should be validated, in addition to platform accuracy, to account for the ability to reach decent coverage in genomic areas of interest and tolerate certain base-calling error rates. Overall, we achieved high accuracy, reproducibility, repeatability, diagnostic sensitivity, and specificity for all assay analytes ranging from 99 to 100%, which exceeds the 90% threshold for LDT performance parameters per CLIA requirements. These findings are in agreement with several recent reports of 93% to 100% accuracies in WGS identification, subtyping, and antimicrobial resistance gene detection for a number of pathogens (44–47). We determined SNP detection at coverages of 5× to 60× and established an LOD for SNP detection at 60×, which was the lowest coverage that yielded accurate SNP detection in all of the samples. We determined the effect of contaminating reads on the analytical specificity of WGS. We point out that CLIA LDT performance parameters are difficult to apply to WGS analysis. For example, in determining the accuracy of the platform, CLIA would allow for up to 10% of base calls to be incorrect, which, in the case of the ∼5-Mb E. coli genome, would mean 500,000 inaccurate SNPs, which is clearly an unacceptable error rate for any WGS application. Genome-wide hqSNP detection can be used as a way to validate platform accuracy since it allows assessment of the accuracy of base calling throughout the genome. Additionally, validation of the actual hqSNP genotyping pipeline would be required, as the phylogenetic assay takes into account not only the number of SNPs detected between isolates but also how it affects the tree topology, because one erroneous SNP is unlikely to change genotyping conclusions in most instances.

Successful CLIA integration for WGS would also require a laboratory to implement a continuous performance measurement plan via an internal or external PT program. Such PT programs are under active development, with the GMI network, the Genetic Testing Reference Materials Coordination Program (Get-RM), the Genome in a Bottle (GIAB) Consortium, and CDC PulseNet NextGen being the most prominent (31, 43). A set of generic standards has been proposed by the CAP molecular pathology checklist (30). The proposed quality standards include both live cultures as well as “sequence-only” formats for a comprehensive assessment of the WGS pipeline. Our validation set of isolates is amenable to both internal and external quality assurance testing. In preliminary internal PT, we were able to successfully assess the entire workflow and personnel performance (details not shown).

Microbial WGS remains a dynamic technology, and therefore, any validated pipeline is unlikely to remain static. For this reason, the implementation of a modular validation template becomes crucial for the seamless and timely introduction of changes to the pipeline; e.g., we had to carry out several amendments to the protocol since its implementation in the laboratory. These amendments included a new processing algorithm for highly contagious pathogens and minor adjustments in the data analysis algorithm. The changes were accomplished via minor modifications of the pipeline with corroborative testing using a modular validation template developed by us. We also performed a two-sequencer agreement study to allow the processing of increased volumes of samples (see Document S1 in the supplemental material). The WGS report format could pose challenges for the end user. The report format in our study was designed to convey assay results to an end user with or without extensive knowledge of WGS. Additional disclaimers were used to avoid erroneous interpretations of results, for example, the disclaimer that the detection of antibiotic resistance genes by WGS does not guarantee the resistance of the strain *in vivo* and that phenotypic susceptibility testing is required to confirm antimicrobial resistance (see section on results reporting in Document S1 in the supplemental material).

This study possesses certain limitations. First, only a limited number of WGS-based assays were included in the validation study based on the most common PHL applications. Other types of WGS assays/analytics would have to be validated in a similar manner to determine the performance specifications, which are required to generate accurate and reproducible results, e.g., a threshold for the base-calling accuracy of the platform or a depth of coverage of specific genes. Second, not all validation set samples had available NCBI database entries to provide comparison sets. Third, the absence of any eukaryotic pathogens in the current validation scheme would require the implementation of a specialized pipeline for pathogenic fungi and parasites. Finally, the presence of extrachromosomal elements or polymorphic or repeat regions can negatively affect reference mapping and the *de novo* assembly of the reads generated on short-read platforms such as the Illumina platform; however, the direct effect of such genomic features on assay performance was not investigated in this study.

As the clinical and public microbiology community implements high-quality WGS, it would be opportune to consider the available models for the delivery of these services (48). Since their inception, most WGS activities have taken place in reference facilities with rather large supporting infrastructures. Although inevitable in the early stages, the centralization of services presents several challenges, such as turnaround time and access to specific expertise on the local population structure of a given pathogen, which is crucial for the management of infectious diseases at the local and regional levels. WGS services could be delivered locally and more easily with affordable sequencers, standardized reagents, and well-defined quality metrics. The local-delivery model would also be more responsive to the needs of the target clients and enhance the adoption of WGS across health care systems. Another alternative is a hybrid model with complementary central and local services to balance the need for speed with advanced expertise and resources (48). Two prominent examples of hybrid models in the United States are the FDA GenomeTrakr network for the tracking of foodborne pathogens and the CDC Advanced Molecular Detection (AMD) initiative for the improved surveillance of infectious diseases (49, 50). The AMD and GenomeTrakr frameworks rely on a participatory model with enhanced analysis, curation, and data storage at a central site. However, these resource-intensive networks focus on few selected pathogens at present. Notably, there are still significant challenges for the implementation of comprehensive WGS services at the local level (42, 51). We hope that the quality framework proposed in the present study will advance the localization of comprehensive WGS services in clinical and public health laboratories.

In summary, the salient achievements of this study included (i) the establishment of performance specifications for WGS applications in PHLs according to CLIA guidelines, (ii) development of quality assurance and quality control measures, (iii) a reporting format for end users with or without WGS expertise, (iv) availability of a validation set of microorganisms to be used for future validations, and (v) creation of a modular template for the validation of WGS processes in PHLs.

## MATERIALS AND METHODS

### Bacterial isolates and sequences.

A set of 34 bacterial isolates representing the typical workflow in PHLs was used for validation and quality control of WGS. These isolates included 10 Enterobacteriaceae isolates, 5 Gram-positive bacterial pathogens, 5 Gram-negative nonfermenting bacterial pathogens, 9 Mycobacterium tuberculosis isolates, and 5 miscellaneous bacterial pathogens (Tables 3 and 4). The bacterial pathogens for the validation set were selected to represent various genome sizes and GC contents and to account for differences in the DNA extraction protocols. We selected ATCC strains with whole-genome sequences available from the NCBI; the NCBI sequences served as references for the validation sequencing results. Several isolates with reference sequences available from the CDC were also included in the validation set.

**TABLE 3:** List of strains used for validation and corresponding reference materials^*a*^

MDL ID	Species	Reference genome	
		NCBI strain	NCBI accession no.
C1	Escherichia coli O157:H7 CDC EDL 933	**O157:H7 CDC EDL 933**	**NZ_CP008957.1**
C3	Escherichia coli ATCC 8739	**ATCC 8739**	**NC_010468.1**
C55	Escherichia coli ATCC 25922	**ATCC 25922**	**NZ_CP009072.1**
C4	Enterobacter cloacae ATCC 13047	**ATCC 13047**	**NC_014121**
C6	Salmonella enterica serovar Typhimurium ATCC 14028	**14028S**	**NC_016856**
C5	Staphylococcus aureus ATCC 25923	**ATCC 25923**	**NZ_CP009361**
C46	Enterococcus faecalis ATCC 29212	**ATCC 29212**	**NZ_CP008816**
C47	Staphylococcus epidermidis ATCC 12228	**ATCC 12228**	**NC_004461**
C48	Staphylococcus saprophyticus ATCC 15305	**ATCC 15305**	**NC_007350**
C49	Streptococcus pneumoniae ATCC 6305	ATCC 700669	FM211187
C50	Pseudomonas aeruginosa ATCC 27853	FRD1	NZ_CP010555
C51	Stenotrophomonas maltophilia ATCC 13637	**ATCC 13637**	**NZ_CP008838**
C52	Legionella pneumophila SG-12 ATCC 43290	**ATCC 43290**	**NC_016811**
C53	Moraxella catarrhalis 87A-3084	ATCC 25240	NZ_CP008804
C54	Acinetobacter baumannii ATCC 17945	PKAB07	NZ_CP006963
C103	Bacteroides fragilis ATCC 25285	638R	NC_016776
C104	Haemophilus influenzae ATCC 10211	KR494	NC_022356
C2	Aeromonas hydrophila ATCC 7966	**ATCC 7966**	**NC_008570**
C105	Corynebacterium jeikeium ATCC 43734	**ATCC 43734**	**GG700813**, **GG700833**
C106	Neisseria gonorrhoeae ATCC 49226	MS11	NC_022240
C56	Mycobacterium tuberculosis	H37Rv	NC_000962.3
C57	Mycobacterium tuberculosis	H37Rv	NC_000962.3
C58	Mycobacterium tuberculosis	H37Rv	NC_000962.3
C59	Mycobacterium tuberculosis	H37Rv	NC_000962.3
C61	Mycobacterium tuberculosis	H37Rv	NC_000962.3
C65	Mycobacterium tuberculosis	H37Rv	NC_000962.3
C67	Mycobacterium tuberculosis	H37Rv	NC_000962.3
C68	Mycobacterium tuberculosis	H37Rv	NC_000962.3
C69	Mycobacterium tuberculosis	H37Rv	NC_000962.3

a Boldface type indicates reference strains for which genomes are available from the NCBI database. Lightface type indicates cases where the genome is not available from the NCBI database and an alternative reference genome was used for mapping. MDL, Microbial Diseases Laboratory, California Department of Public Health, Richmond, CA.

**TABLE 4:** List of strains used for validation and corresponding reference materials available from the CDC^*a*^

MDL ID	Species	Reference raw reads generated by the CDC		Reference genome used for mapping	
		CDC strain	GenBank accession no.	NCBI strain	NCBI accession no.
C72	Escherichia coli O121:H19	**2014C-3857**	**SRR1610033**	2011C-3493	NC_018658
C73	Salmonella enterica serovar Enteritidis	**CDC_2010K-1543**	**SRR518749**	P125109	NC_011294.1
C74	Salmonella enterica serovar Infantis	**2014K-0434**	**SRR1616809**	1326/28	NZ_LN649235
C75	Salmonella enterica serovar Adelaide	**2014K-0941**	**SRR1686419**	P125109	NC_011294.1
C76	Salmonella enterica serovar Worthington	**2012K-1219**	**SRR1614868**	P125109	NC_011294.1
C77^*b*^	Salmonella enterica serovar Saintpaul	**2014K-0875**	**SRR1640105**	14028S	NC_016856

a Boldface type indicates the reference strains for which genomes are available from the NCBI database. MDL, Microbial Diseases Laboratory, California Department of Public Health, Richmond, CA.

b Sample C77 was sequenced by the MDL only for genotyping assay accuracy validation. No replicates were done.

### Reference whole genomes.

The genome sequences of ATCC strains, isolates characterized by the CDC, and other representative isolates were downloaded from the NCBI database (http://www.ncbi.nlm.nih.gov/genome/) to be used as a reference according to CLSI guidelines (37) (Tables 3 and 4).

### WGS wet-bench workflow.

Whole-genome sequencing was performed on an Illumina MiSeq sequencer (see Fig. S1 in the supplemental material). The Nextera XT library preparation procedure and 2 × 300-cycle MiSeq sequencing kits were used (Illumina Inc., San Diego, CA, USA). Illumina Nextera XT indexes were used for barcoding. Bacterial DNA was extracted by using the Wizard Genomic DNA kit (Promega, Madison, WI, USA). The bacterial DNA concentrations were measured by using Qubit fluorometric quantitation with a Qubit double-stranded RNA (dsDNA) BR assay kit (Thermo Fisher Scientific, Waltham, MA, USA). DNA purity was estimated by using a NanoDrop 2000 UV-visible (UV-Vis) spectrophotometer (NanoDrop Products, Wilmington, DE, USA). The Mastercycler nexus was used for incubation for tagmentation reaction and PCR (Eppendorf North America, Hauppauge, NY, USA). The library concentration was measured by using a Qubit HS kit. The DNA library size distribution was estimated by using a 2100 BioAnalyzer instrument and a High Sensitivity DNA analysis kit (Agilent Technologies, Santa Clara, CA, USA). Ampure beads were used for size selection. Manual normalization of libraries was performed. Genome sizes of samples pooled in one run were taken into account to ensure the equal representation of each genome per flow cell. The number of samples was kept at 16 per run (except for one run with 19 samples), resulting in a total genome load of 48 to 84 Mb/run (average, 69 Mb/run). The PhiX Control V3 sequencing control at 1% was spiked in for every sequencing run (Illumina Inc., San Diego, CA, USA). Genomes were generated with a depth of coverage (estimated for trimmed mapped reads) in the range of 15.71× to 216.4× (average, 79.72×; median, 71.55×).

The preliminary run and raw data acceptance criteria were set for (i) the percentage of bases with a ≥Q30 quality score for the run of ≥50%, with the requirement that the Q30 score for the generated genome sequences must be ≥75% for at least 80 bp of the read length; (ii) a minimum average coverage of the genome of 10×; (iii) a PhiX error rate that must be <6%; and (iv) the negative control meeting the following parameters: <10,000 reads after trimming, an N50 value of <1,000, and the highest coverage of *de novo*-assembled contigs of <10×. Data which did not meet these quality parameters were rejected. The preliminary quality thresholds were further adjusted based on the quality ranges observed during validation.

### Bioinformatics pipeline.

Paired-end reads were quality trimmed with the threshold of Q30 and then used for mapping to the reference and *de novo* assemblies on CLCbio Genomic Workbench 8.0.2 (Qiagen, Aarhus, Denmark). The BAM files generated after mapping to the reference genome were taken through series of software suites to generate the phylogenetic tree. A customized shell script was created to automate the subsequent steps after mapping, which included (i) calculating the genotype likelihood using SAMtools mpileup (v.1.2) (52), (ii) conversion to a VCF matrix using bcftools view (v0.1.19; http://samtools.github.io/bcftools/), (iii) single nucleotide polymorphism calling in coding and noncoding genome areas using bcftools call —c (v0.1.19), (iv) variant parsing using vcftools (v.0.1.12b) (53) to include only hqSNPs with a mean coverage of ≥30× and a minimum SNP quality (min*Q*) of ≥200 with indels and heterozygote calls excluded and the allele frequency defining a homozygous call at ≥93%, and (v) conversion of the SNP matrix to a FASTA alignment file for export back to CLCbio Genomic Workbench 8.0.2 for the generation of the phylogenetic tree.

### hqSNP-based genotyping.

Maximum likelihood phylogenetic trees were generated based on hqSNPs under the Jukes-Cantor nucleotide substitution model; bootstrapping included 100 replicates.

### 16S rRNA gene-based identification.

Genomes were annotated with prokka v1.1 (54), and species identification was performed by comparing 16S rRNA gene sequences against data in the Ribosomal Database Project (RDP) database (55).

### *In silico* MLST.

*In silico* MLST was performed by using the Center for Genomic Epidemiology (CGE) online tool (56).

### Detection of antibiotic resistance genes.

ABR gene detection was performed by using the CGE ResFinder online resource (57) with 99% identity and 100% query length coverage thresholds. Two sets of sequences were analyzed. For the first set, ATCC reference bacterial strains designated for use as antibiotic susceptibility controls were sequenced by the laboratory performing the validation. Negative controls were chosen among strains described as being susceptible, with no known antibiotic resistance genes, according to CLSI document M100-S25 (58). Positive controls were chosen among strains that possessed resistance determinants, according to CLSI document M100-S25. For the second set, reference sequences were acquired from the FDA-CDC Antimicrobial Resistance Isolate Bank (https://www.cdc.gov/drugresistance/resistance-bank/) for *in silico* testing. Thirteen isolates (7 Gram-negative and 6 Gram-positive organisms) with various resistance genes were analyzed.

### Validation plan.

Thirty-four bacterial isolates were sequenced in triplicate. The WGS pipeline, including wet- and dry-bench processes, was validated by assessing the performances of the platform, specific WGS-based assays, and the bioinformatics analysis pipeline (Fig. 1). Validated WGS assays included genome-wide SNP-based genotyping, MLST, 16S rRNA species identification, and antibiotic resistance gene detection. The following performance characteristics were assessed: accuracy, reproducibility (between-run precision), repeatability (within-run precision), analytical and diagnostic sensitivity, and analytical and diagnostic specificity.

For reproducibility assessments, all between-run replicates were generated starting from fresh cultures except for M. tuberculosis, where DNA samples were used. Between-run replicates were processed on three different days, alternating between two operators, as recommended in CLSI document MM11A (59). For repeatability, within-run replicates were prepared from one DNA extract, but independent library preparations were done, with the final samples being included in one sequencing run. The reproducibility and repeatability of the genotyping assay were evaluated with two methods: (i) evaluation of the reproducibility and repeatability of SNP calling per replicate takes into account the whole genome of one replicate as a single test, meaning that any number of single nucleotide changes in one out of three of the replicates was regarded as 33.3% disagreement for that validation sample, and (ii) the reproducibility and repeatability of SNP calling relative to the genome size were calculated by taking a discordant SNP into account as a percentage of the number of base pairs in the genome (genome size); e.g., a 10-SNP difference among the replicates with 5-Mb genomes equals 99.9998% reproducibility.

### Analytical sensitivity.

The LOD of SNP calling was estimated by modeling different mapping coverages and estimating the minimum coverage that allowed accurate SNP calling (LOD_SNP_). Mapping BAM files were downsampled in order to achieve different coverage values (60×, 50×, 40×, 30×, 20×, 15×, 10×, and 5×) for each of the nine samples representative of the different species. The original sequence mapping coverage was estimated from the BAM file by using the following command: samtools depth bamfile_sorted.bam ∣ awk ‘{sum+ = $3} END {print “Average =”,sum/NR}’ The bam files were downsampled to the desired coverage by using the command samtools view —h —s F bamfile_sorted.bam >bamfile_sorted_30x.bam, where F is a fraction of the desired coverage in relation to the original coverage. Downsampled BAM files were compared to other replicates for the same sample at their original coverage.

### Analytical specificity.

To determine analytical specificity (interference), we generated *in silico* sequencing files containing a mixture of reads from two different samples, thus mimicking contamination. The effect of potentially interfering sequencing reads on mapping metrics (percentage of reads mapped/not mapped, percentage of the reference sequence covered, etc.) and SNP detection was estimated. Sample C3 (Escherichia coli ATCC 8739) was selected as a “control, not-contaminated sample,” and equal parts of reads from different species were merged with it to generate mixed fastq files. This was done by using the concatenate command, e.g., cat file1 file2 > mixed file. To ensure that the correct file was generated, the numbers of lines in both files and the mixed file were counted. The number of lines in the mixed file was the sum of the numbers of lines in the two parent files. Also, the column headings were checked to ensure that the headers of the mixed file were similar to those of the parent file. “Contaminated” and “not-contaminated” reads were mapped to the same reference genome of E. coli ATCC 8739 from the NCBI. Further SNP calling analysis was performed by using the standardized pipeline under validation. The specificity of SNP calling in the samples containing potentially interfering reads was estimated by comparing (i) the number of SNPs detected between the contaminated sequence and the reference sequence and (ii) the number of SNPs detected between the contaminated and not-contaminated sequences included in the same SNP calling analysis (vcf file after filtering).

### Accession number(s).

Data from this whole-genome shotgun project have been deposited in GenBank under raw read accession numbers SRR4114366 to SRR4114399 and assembly accession numbers MTFS00000000 to MTGZ00000000 (see Table S1 in the supplemental material). All WGS data, including sequences of the replicates, are associated with BioProject accession number PRJNA341407.

## Supplementary Material

Supplemental material
