# Supplementary material for: Validation and Implementation of Clinical Laboratory Improvements Act-Compliant Whole-Genome Sequencing in the Public Health Microbiology Laboratory
Source: J Clin Microbiol. 2017 Jul 25;55(8):2502–20. doi: 10.1128/JCM.00361-17 (PMC5527429; doi:10.1128/JCM.00361-17)
Supplement: Supplemental material [file supp_55_8_2502__index.html]

Supplemental material 

# Validation and Implementation of Clinical Laboratory Improvements Act-Compliant Whole-Genome Sequencing in the Public Health Microbiology Laboratory

## Supplemental material

- Supplemental file 1 -

  Fig. S1 (WGS workflow); Tables S1 (NCBI accession numbers for WGS validation set sequences), S2 (Summary of phylogenetic trees built to assess genotyping accuracy), S3 (Reproducibility and repeatability of base calling), S4 (LOD for SNPs), S5 (Effect of the interfering sequencing reads on the mapping metrics and specificity of SNP calling), and S6 (WGS quality cutoff values); and Document S1 (Assay validation report for WGS on MiSeq Illumina platform)

  PDF, 13M
