# Supplementary material for: Validation and Implementation of Clinical Laboratory Improvements Act-Compliant Whole-Genome Sequencing in the Public Health Microbiology Laboratory
Source: J Clin Microbiol. 2017 Jul 25;55(8):2502–20. doi: 10.1128/JCM.00361-17 (PMC5527429; doi:10.1128/JCM.00361-17)
Supplement: Supplemental material [file JCM.00361-17_zjm999095603s1.pdf]

Figure S1. **WGS workflow (wet- and dry-bench)**

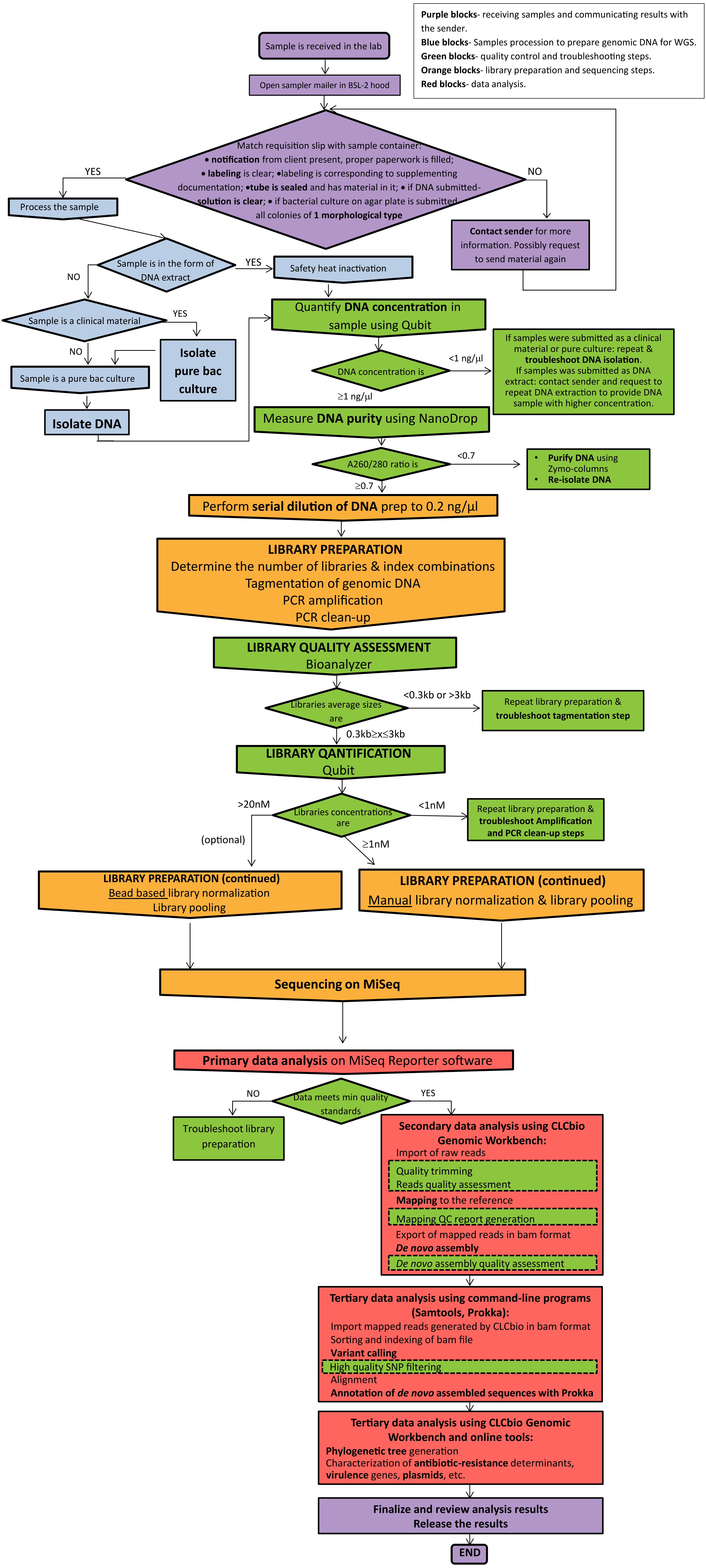

Table S1. NCBI accession numbers for the WGS validation set sequences.

Sample ID	Organisms	Library ID	Raw reads accession number	Assembly accession number
C1	*Escherichia coli* EDL 933	C1_2	SRR4114366	MTFS00000000
C2	*Aeromonas hydrophila* ATCC 7966	C2_1	SRR4114375	MTGJ00000000
C3	*Escherichia coli* ATCC 8739	C3_2a	SRR4114367	MTFT00000000
C4	*Enterobacter cloacae* ATCC 13047	C4_2a	SRR4114389	MTFV00000000
C5	*Staphylococcus aureus* ATCC 25923	C5_2	SRR4114395	MTFX00000000
C6	*Salmonella enterica* subsp. *enterica* serovar Typhimurium ATCC 14028	C6_3a	SRR4114394	MTFW00000000
C46	*Enterococcus faecalis* ATCC 29212	C46_1	SRR4114396	MTFY00000000
C47	*Staphylococcus epidermidis* ATCC 12228	C47_1c	SRR4114397	MTFZ00000000
C48	*Staphylococcus saprophyticus* subsp. *saprophyticus* ATCC 15305	C48_1a	SRR4114398	MTGA00000000
C49	*Streptococcus pneumoniae* ATCC 6305	C49_1	SRR4114399	MTGB00000000
C50	*Pseudomonas aeruginosa* ATCC 27853	C50_1a	SRR4114368	MTGC00000000
C51	*Stenotrophomonas maltophilia* ATCC 13637	C51_3a	SRR4114369	MTGD00000000
C52	*Legionella pneumophila* subsp. *pneumophila* ATCC 43290	C52_1b	SRR4114370	MTGE00000000
C53	*Moraxella catarrhalis* CDPH_C53	C53_1	SRR4114371	MTGF00000000
C54	*Acinetobacter baumannii* ATCC 17945	C54_1	SRR4114372	MTGG00000000
C55	*Escherichia coli* ATCC 25922	C55_3c	SRR4114378	MTFU00000000
C56	*Mycobacterium tuberculosis* CDPH_C56	C56_1a	SRR4114384	MTGR00000000
C57	*Mycobacterium tuberculosis* CDPH_C57	C57_1a	SRR4114385	MTGS00000000
C58	*Mycobacterium tuberculosis* CDPH_C58	C58_1a	SRR4114386	MTGT00000000
C59	*Mycobacterium tuberculosis* CDPH_C59	C59_1a	SRR4114387	MTGU00000000
C61	*Mycobacterium tuberculosis* CDPH_C61	C61_1a	SRR4114388	MTGV00000000
C65	*Mycobacterium tuberculosis* CDPH_C65	C65_1	SRR4114390	MTGW00000000
C67	*Mycobacterium tuberculosis* CDPH_C67	C67_1	SRR4114391	MTGX00000000
C68	*Mycobacterium tuberculosis* CDPH_C68	C68_1	SRR4114392	MTGY00000000
C69	*Mycobacterium tuberculosis* CDPH_C69	C69_1	SRR4114393	MTGZ00000000
C72	*Escherichia coli* CDPH_C72	C72_1a	SRR4114379	MTGM00000000
C73	*Salmonella enterica* subsp. *enterica* serovar Enteritidis CDPH_C73	C73_1a	SRR4114380	MTGN00000000
C74	*Salmonella enterica* subsp. *enterica* serovar Infantis CDPH_C74	C74_1a	SRR4114381	MTGO00000000
C75	*Salmonella enterica* subsp. *enterica* serovar Adelaide CDPH_C75	C75_1a	SRR4114382	MTGP00000000
C76	*Salmonella enterica* subsp. *enterica* serovar Worthington CDPH_C76	C76_1a	SRR4114383	MTGQ00000000
C103	*Bacteroides fragilis* ATCC 25285	C103_1	SRR4114373	MTGH00000000
C104	*Haemophilus influenzae* ATCC 10211	C104_1	SRR4114374	MTGI00000000
C105	*Corynebacterium jeikeium* ATCC 43734	C105_1	SRR4114376	MTGK00000000
C106	*Neisseria gonorrhoeae* ATCC 49226	C106_1	SRR4114377	MTGL00000000

Footnotes: Listed sequences were used for accuracy determination. Raw WGS data for all replicates is available from the BioProject PRJNA341407.

Table S2. Summary of the phylogenetic trees built to assess genotyping accuracy.

**Species**	**Sample ID**	**Reference sequences for the isolates sequenced during validation**	**Additional references used for comparison**	**Clustering of reference tree was replicated for all validation samples (Y/N)**	**% Tree similarity**	**% agreement** (average tree similarity for different species)
Escherichia coli	C1, C3, C55	NZ_CP008957.1, NC_010468.1, NZ_CP009072.1	NC_000913.3, NC_002695.1	Y	100%	100%
Salmonella enterica	C73, C74, C75, C76, C77	SRR518749, SRR1616809, SRR1686419, SRR1614868, SRR1640105	none	Y	100%	
Staphylococcus aureus	C5	NZ_CP009361	NC_007622, NC_007795, NC_009782, NC_017333	Y	100%	
Enterococcus faecalis	C46	NZ_CP008816	NC_018221, NC_019770, NZ_CP004081, NC_004668	Y	100%	
Stenotrophomonas maltophilia	C51	NZ_CP008838	NC_011071, NC_015947, NC_017671, NC_010943	Y	100%	

Table S3. Reproducibility and repeatability of base calling

Sample	Total # of SNP difference for within-run replicates	Total # of SNP difference for between-run replicated	# of within-run replicates in agreement	# of between-run replicates in agreement	Precision per replicate	Genome size of reference, bp	Length of covered genome, bp	Precision per base pair	
								Repeatability	Reproducibi lity
C1	0	0	3	3	Repeatability = 99.02% Reproducibility = 97.05%	5639400	5244642	100	100
C2	0	0	3	3		4744448	4744448	100	100
C3	0	0	3	3		4746220	4698758	100	100
C4	0	0	3	3		5598800	5486824	100	100
C5	0	0	3	3		2806340	2750213	100	100
C6	0	0	3	3		4964100	4914459	100	100
C46	0	0	3	3		2939973	2939973	100	100
C47	0	2	3	2		2499279	2499279	100	99.9999997
C48	0	0	3	3		2516575	2516575	100	100
C49	0	1	3	2		2221315	1954757	100	99.9999998
C50	1	0	2	3		6712339	6175352	99.9999999	100
C51	0	0	3	3		4989312	4989312	100	100
C52	0	0	3	3		3359001	3359001	100	100
C53	0	0	3	3		1941566	1844488	100	100
C54	0	0	3	3		4233806	3641073	100	100
C55	0	1	3	2		5203440	5047337	100	99.9999999
C72	0	0	3	3		5273097	4534863	100	100
C73	0	0	3	3		4685848	4638990	100	100
C74	0	0	3	3		4710675	4569355	100	100
C75	0	0	3	3		4685848	4310980	100	100
C76	0	0	3	3		4685848	4357839	100	100
C103	0	0	3	3		5373121	4567153	100	100
C104	0	0	3	3		1856176	1670558	100	100
C105	0	0	3	3		2492821	2368180	100	100
C106	0	0	3	3		2233640	2121958	100	100
C56	0	0	3	3		4411532	4279186	100	100
C57	0	0	3	3		4411532	4279186	100	100
C58	0	0	3	3		4411532	4279186	100	100
C59	0	0	3	3		4411532	4323301	100	100
C61	0	0	3	3		4411532	4279186	100	100
C65	0	0	3	3		4411532	4323301	100	100
C67	0	0	3	3		4411532	4323301	100	100
C68	0	0	3	3		4411532	4323301	100	100
C69	0	0	3	3		4411532	4323301	100	100
Total	1	4	101	99			Average:	**99.9999999**	**99.9999999**

Footnotes: The discrepancies between replicates in this table are highlighted with orange.

Table S4. **Limit of detection (LOD) for SNPs**

Sample ID	Species	Original coverage	Number of SNPs detected between the validation sample and reference								
			At original coverage	60x	50x	40x	30x	20x	15x	10x	5x
C1_3b	*Escherichia coli* O157:H7	69.3x	5	5	5	5	5	5	4	4	4
C2_2a	*Aeromonas hydrophilia*	116.5x	1	1	1	1	1	1	1	1	1
C3_2b	*Escherichia coli*	85.2x	22	22	21	20	19	18	17	17	15
C5_1	*Staphylococcus aureus*	145x	0	0	0	0	0	0	0	0	0
C6_3a	*Salmonella enterica* ser Typhimurium	86.6x	12	12	12	8	8	7	7	7	7
C46_3	*Enterococcus faecalis*	58x	3	-	3	3	3	3	3	3	4
C52_2	*Legionella pneumophila*	99.8x	2	2	2	2	2	2	2	2	2
C72_2	*Escherichia coli* O121:H19	53x	0	-	0	0	0	0	0	0	0
C73_1c	*Salmonella enterica* ser Enteritidis	76.8x	0	0	0	0	0	0	0	0	0

Footnotes: The LOD for SNPs was estimated by downsampling the original genome coverage of the selected isolates.
LOD for SNPs detection was 60x.

Table S5. **Effect of the interfering sequencing reads on the mapping metrics and specificity of SNP calling**

**Samples**		C3 *E.coli*	C3 *E.coli* + C75 *Salmonella enterica*	C3 *E.coli* + C1 *E.coli*	C3 *E.coli* + C54 *Acinetobacter baumannii*	C3 *E.coli* + C57 *Mycobacterium tuberculosis*	C3 *E.coli* + C5 *Staphylococcus aureus*
**Mapping quality metrics**	**% of Contamination reads**	no contamination	50%	50%	50%	50%	50%
	**% of Mapped reads**	99%	68%	91%	51%	67%	49%
	**% of Not mapped reads**	1%	32%	9%	49%	33%	51%
	**% of Reads in pairs**	90%	59.60%	81%	48.30%	60%	46.60%
	**% of Broken paired reads**	9%	8%	10%	3%	6%	3%
	**% of reference covered**	99%	99%	99%	99%	99%	99%
	**Average coverage**	86.54x	124.96x	115.15x	152.59x	150.73x	152.04x
**SNP calling specificity**	**SNPs between sequence and reference**	22	24	22	40	22	36
	**Number of missed SNPs**	NA	1	0	0	0	0
	**Number of nonspecific SNPs**	NA	1	0	18	0	14
	**SNPs between “contaminated” and “not-contaminated” sequence**	NA	2	0	18	0	14

Table S6. **WGS quality cut-off values**

Step	Parameter	Passing Threshold				
			Positive control		Negative control	
		Sample	Internal (PhiX)	External (*E.coli* ATCC 25922)	Internal (index combination)	External (no-template)
**DNA template QC**	Quality of the input DNA (260/280 absorbance)	>1.7	NA	>1.7	NA	NA
	Quantity of the input DNA (Qubit fluorometer)	≥1ng/µl	NA	≥1ng/µl	NA	≤0.1ng/µl
**Library QC**	DNA library size distribution (BioAnalyzer)	300bp-3kb	NA	300bp-3kb	NA	no peak
	DNA library concentration (Qubit fluorometer)	≥1nM	NA	≥1nM	NA	<0.5ng/µl
**Sequencing run QC**	Percent of bases with quality score >Q30 for the run (%Q30)	> 57%				
	Cluster density (optional)	800-1700K/mm^2				
	Cluster passing filter (optional)	>72%				
	PhiX error rate	NA	<4.9%	NA	NA	NA
**Raw data QC**	The average read length after trimming and discarding the base pairs with quality score <Q30(optional)	>109bp	NA	>109bp	NA	NA
	Minimum read length of the fragments which have ≥75% of bases with Q30 score	86bp	NA	86bp	NA	NA
	Average depth of coverage	≥ 15x*	NA	≥ 15x	< 10x	< 10x
**Data analysis QC**	Uniformity of coverage (optional):<br><br>% of positions on the target(coding sequence) with ≥10x coverage.<br><br>% of positions on the target (coding sequence) with ≥5x coverage.	>50%<br><br>>70%	NA	>50%<br><br>>70%	NA	NA
	Fraction of reference covered	> 0.8	NA	> 0.8	NA	NA
	Number of reads after trim	NA	NA	NA	<10,000	<10,000
	N50 for *de novo* assembled reads	> 30,132 (optional)	NA	> 30,132 (optional)	< 1,000	< 1,000
	The highest coverage of *de novo* assembled contigs	NA	NA	NA	< 10x	< 10x
	MLST result	NA	NA	ST73	NA	NA
	16S species ID	Must match submitter's ID	NA	*Escherichia coli*	NA	NA
	ResFinder	NA	NA	No genes found	NA	NA
	VirulenceFinder	NA	NA	*mchB, mchC, iss, mchF, mcmA, iha, sat, vat, iroN*	NA	NA
	Average genome coverage for SNP detection	≥ 60x*	NA	NA	NA	NA
	Phylogenetic analysis (Epidemiologically unrelated control)	Epidemiologically unrelated control should not cluster with tested samples on the phylogenetic tree	NA	NA	NA	NA

Footnotes: *Minimum coverage for SNP detection as well as optimal coverage for the raw data are 60x, however the minimum acceptance criteria is 15x for raw data in order to be considered for other types of analysis (16S ID, ABR genes detection, MLST)

Document S1. Assay validation report for the whole genome sequencing (WGS) on MiSeq Illumina platform.

California Department of Public Health (CDPH)
Microbial Diseases Laboratory (MDL)

ASSAY VALIDATION REPORT FOR THE WHOLE GENOME SEQUENCING ON MISEQ ILLUMINA PLATFORM

Version 05-24-2017

DOCUMENT CONTROL

Annual review / revisions as needed:

No.	Revision	Pages	Initials/Date

Purpose

Passed in 1988, the Clinical Laboratory Improvement Amendments (CLIA) establishes quality standards for all laboratory testing to ensure the accuracy, reliability and timeliness of patient test results regardless of where the test was performed. The CLIA regulations require validation of the performance specifications of in-house developed tests that not cleared or not approved by the FDA. Following this requirement, the MDL Core Laboratory performed validation of the performance characteristics of the **MiSeq Illumina platform for whole genome sequencing** application.

Table of Contents

1. Description of the assay 9
2. Intended use of the assay 15
3. Regulations 16
4. Acceptable and not acceptable specimens 19
4.1. Biosafety Risk group 2 specimens 19
4.1.1. Acceptable specimens for microorganisms belonging to Biosafety Risk group 2 19
4.1.2. Not acceptable specimens for microorganisms belonging to Biosafety Risk group-2 20
4.2. Biosafety Risk group 3 specimens 20
4.2.1. Acceptable specimens for *Mycobacterium tuberculosis* 20
4.2.2. Not acceptable specimens for *Mycobacterium tuberculosis* 20
4.2.3. Not acceptable specimens for other Biosafety Risk group 3 organisms 20
5. Positive control 21
6. Negative control 21
6.1. Negative control for sequencing process 21
6.2. Negative control for sequencing analysis 21
7. Quality assurance & Quality control 21
7.1. Each run QC 21
7.1.1. Input DNA and DNA library quality and quantity metrics 22
7.1.2. DNA library quality and quantity metrics 22
7.1.3. Quality metrics of the tested samples sequences 22
7.1.4. Quality metrics of spiked in positive PhiX control sequences 23
7.1.5. Quality metrics of negative control sequences 23
7.1.5.1. Quality metrics of negative control sequences for sequencing process 23
7.1.5.2. Quality metrics of negative control for sequencing analysis 23
7.2. Monthly QC 23
7.2.1. Monthly positive QC control 23
7.2.2. Monthly negative QC control 24
7.3. Equipment calibration 24
7.4. Test Rejection Criteria 25

8. Validation 26
8.1. Validation samples 26
8.2. Reference materials 26
8.3. Accuracy 30
8.3.1. MiSeq Platform Accuracy 30
8.3.1.1. Quality parameters affecting platform accuracy 30
8.3.1.1.1. Quality parameters of positive and negative controls. 38
8.3.1.2. Accuracy of base calling against reference sequence 40
8.3.2. Assay accuracy 43
8.3.2.1. Accuracy of *in silico* MLST assay 43
8.3.2.2. Accuracy of 16S rRNA gene identification assay 45
8.3.2.3. Accuracy of Genotyping assay 46
8.3.2.4. Accuracy of antibiotic resistance genes detection assay 48
8.3.2.4.1. Detection of resistance genes in the ATCC strains using ResFinder 48
8.3.2.4.2. *In silico* detection of resistance genes in sequences from the FDA-CDC AR Isolate Bank using ResFinder 49
8.3.3. Accuracy of bioinformatics pipeline 51
8.4. Inter- and Intra-assay Agreement 56
8.4.1. Repeatability and Reproducibility of Genotyping assay 68
8.4.2. Repeatability and Reproducibility for *in silico* MLST assay 71
8.4.3. Repeatability and Reproducibility in 16S rRNA gene ID assay 72
8.5. Analytical sensitivity and Analytical specificity 74
8.5.1. Analytical sensitivity of SNP detection 74
8.5.1.1. Approach 74
8.5.1.2. Limit of SNP detection 74
8.5.2. Analytical specificity of SNP detection 76
8.5.2.1. Approach 76
8.5.2.2. Mapping metrics 76
8.5.2.3. Specificity of SNP calling 76
8.6. Diagnostic sensitivity and Diagnostic specificity 77
8.6.1. Diagnostic sensitivity and specificity of *in silico* MLST assay 78

8.6.2. Diagnostic sensitivity and specificity of Genotyping assay 79
8.7. Reportable range for WGS 83
8.8. Summary of Performance Specifications of the assay 84
9. Summary of quality assurance (QA) and quality control (QC) measures developed during validation 85
10. Results reporting 89
Template form 90
Example of the report 97
References 104
Appendix 1. Reference and Validation tree comparison method 106
Analysis protocol 108
Log of the analysis for each tree comparison 113
Staphylococcus aureus 113
Enterococcus faecalis 114
Escherichia coli 115
Stenotrophomonas maltophilia 116
Salmonella enterica 117
Appendix 2. Calculation of platform performance parameters 118
Appendix 3. Genome size information 131
Appendix 4. Average covered genome size 132
Appendix 5. Data analysis for test validation 133
Trimming of the reads 133
Reads quality check 140
Preparing reference sequence for mapping 141
Using NCBI genome as a reference 141
Using one of the outbreak strains genome as a reference 146
Mapping 148
Mobile elements masking 148
Mapping 152
Detailed mapping report generation 161
Export of mapping files 164

Sorting of exported bam files 166
Parallel mpileup of sorted bam files 167
Filtering of High-quality SNPs 167
Alignment of SNPs in SVAMP 168
Alignment in CLCbio 171
Comparison matrix 174
Data interpretation 177
Appendix 6. MLST and 16s rRNA analysis protocol and results 180
Protocol for 16S rRNA gene analysis 180
Protocol for *in silico* MLST analysis 182
MLST negative controls 186
Appendix 7. Validation testing plan 189
Appendix 8. Quality parameters for sequences of the replicates 194
Appendix 9. Detection of antibiotic resistance genes from WGS data 199
Results 199
Positive controls 199
Negative controls 202
Appendix 10. MLST reproducibility and repeatability validation results 205
Appendix 11. 16S rRNA reproducibility and repeatability validation results 206
Appendix 12. Metrics of the negative controls collected during validation 208
Appendix 13. VirulenceFinder example of the output for positive *E.coli* ATCC 25922 control 210
Appendix 14. Resolution of the discrepancies between validation base calling results and reference sequences with Sanger sequencing 211
Samples selected for the confirmatory testing 211
Sanger sequencing results 212
Summary 216
Conclusion 216
Appendix 15. Comparison of the antibiotic resistance genes detection with PCR-based methods and ResFinder for the isolates from CDC/FDA AR Isolate Bank 217
Appendix 16. Determination of the Limit of SNP Detection. Results 218
Addendum 1. Validation Report for MiSeq Automated Pipeline (MiSeqPipeline Version 1.0 05/11/2016) 220

Addendum 2. Correlation study for two MiSeq Illumina sequencers 224

Standard Operating Procedure: Correlation Testing for Whole Genome Sequencing on Multiple MiSeq Instruments 224

Whole Genome Sequencing Correlation Report 226

1. Description of the assay

Whole genome sequencing (WGS) is an analytical process that determines the complete DNA sequence of an organism's genome in a single reaction. WGS provides the most comprehensive information on genetic variations within and between species, because it can identify low frequency variants and genome rearrangements that may be missed using other molecular methods. Sequencing the entire microbial genome is important for microbial identification, subtyping, and other comparative genomic studies. Illumina sequencing technology used in MiSeq desktop sequencer leverages clonal array formation and reversible terminator technology for rapid and accurate large-scale WGS.

First, genomic DNA (gDNA) is extracted from bacterial cells and the concentration of double stranded DNA is fluorometrically quantified. Next, the gDNA is randomly fragmented by enzyme into a library of small fragments that can be uniformly and accurately sequenced in multiple parallel reactions. Adaptors are ligated to both ends of the DNA fragments. The Nextera XT DNA Sample Preparation Kit uses an engineered transposome to simultaneously fragment and tag ("tagment") input DNA, adding special adapter sequences in the process:

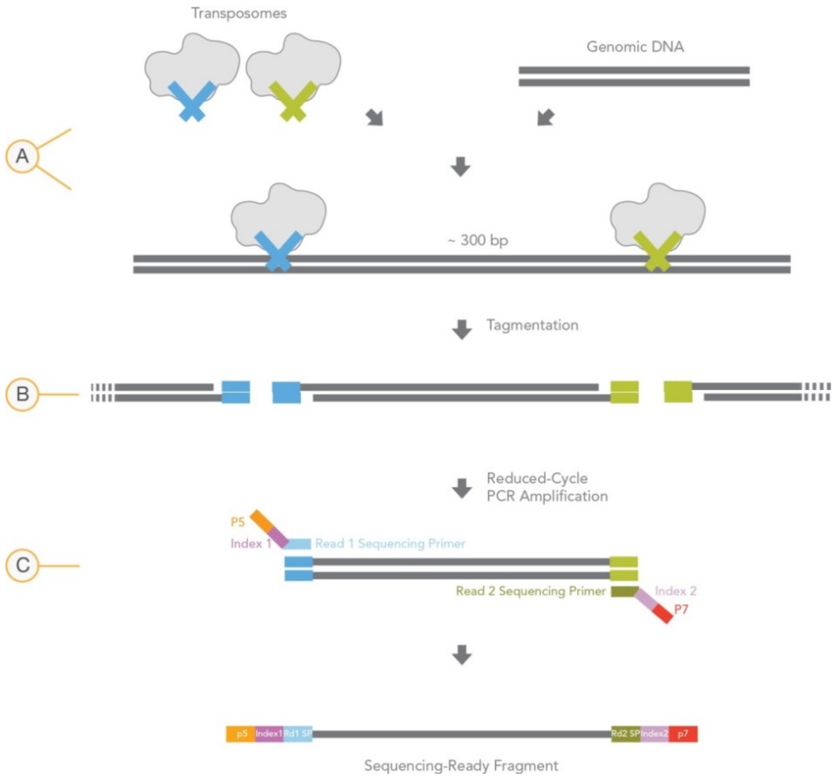

Generation of library fragments using Nextera XT kit

A- Nextera XT transposome with adapters is combined with template DNA
B- Tagmentation to fragment and add adapters
C- Limited cycle PCR to add sequencing primer sequences and indices

A limited-cycle PCR reaction uses these adapter sequences to amplify the insert DNA. A unique combination of index sequences is added to both ends of the tagmented DNA during the PCR reaction, thus enabling dual-indexed sequencing of pooled libraries on any Illumina MiSeq sequencer. All DNA fragments generated from one sample are barcoded with the same signature sequence, while different samples have individual barcodes; this allows multiplexing of up to 96 samples in a single run. The samples are later separated (demultiplexed) by computer algorithm during the data analysis. The quality of the generated libraries is analyzed by electrophoresis in a chip format. The concentration of the generated libraries is fluorometrically quantified. After libraries have passed quantity and quality control they are normalized by their concentrations, then pooled and loaded on MiSeq sequencer, where the sequencing is performed.

Sequencing is performed on a solid surface. Solid phase amplification creates up to 1,000 identical copies (cluster) of each single template molecule in close proximity (diameter of 1 micron or less), which helps signal amplification during sequencing:

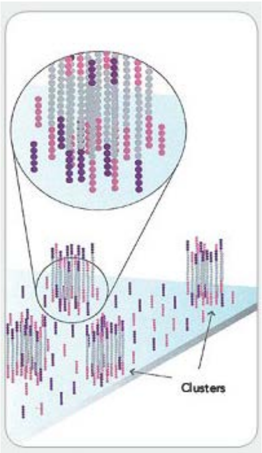

Such called “bridge amplification” is used to create clusters. It starts with random immobilization of single-stranded DNA fragments to the inside surface of the flow cell channels:

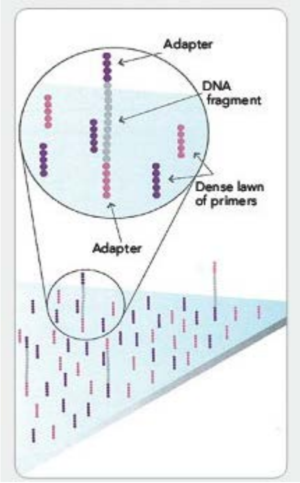

Then, unlabeled nucleotides and enzyme are added to the flow cell to initiate solid phase bridge amplification:

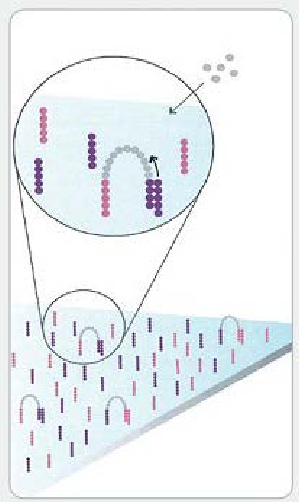

The enzyme incorporates nucleotides to build double-stranded bridges on the solid-phase substrate:

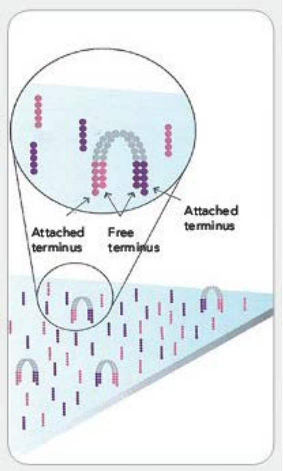

Double stranded molecules are denaturated leaving single-stranded templates anchored to the substrate:

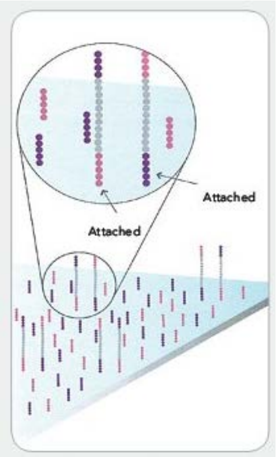

Multiple cycles of bridge amplification create a cluster composed of copies of a single DNA template molecule. Different DNA templates originate different clusters throughout the flow cell. Sequencing of all the molecules within single cluster happens simultaneously. The first sequencing cycle begins by adding 4 labeled reversible terminators, primers, and DNA polymerase:

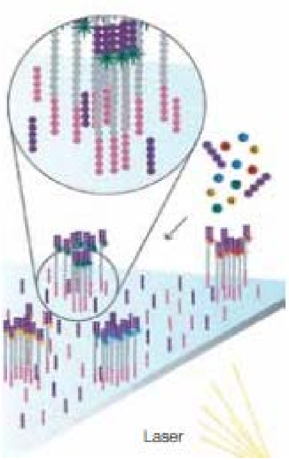

The laser excites fluorescent labels of incorporated nucleotides. The emitted fluorescence from each cluster is captured and the first base is identified:

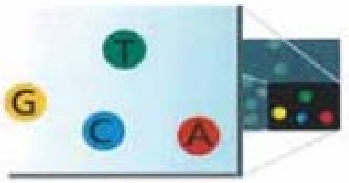

After that, the fluorescent labels of incorporated nucleotides are enzymatically cleaved and washed away. The next cycle repeats the incorporation of 4 labeled reversible terminators, primers, and DNA polymerase. During each sequencing cycle only one nucleotide can be incorporated into growing nucleic acid chain because the nucleotide label serves as a terminator for polymerization. Non-incorporated nucleotides, as well as primers, and DNA polymerase are washed away.

The sequencing cycles are repeated to determine the sequence of bases in a fragment, one base at a time:

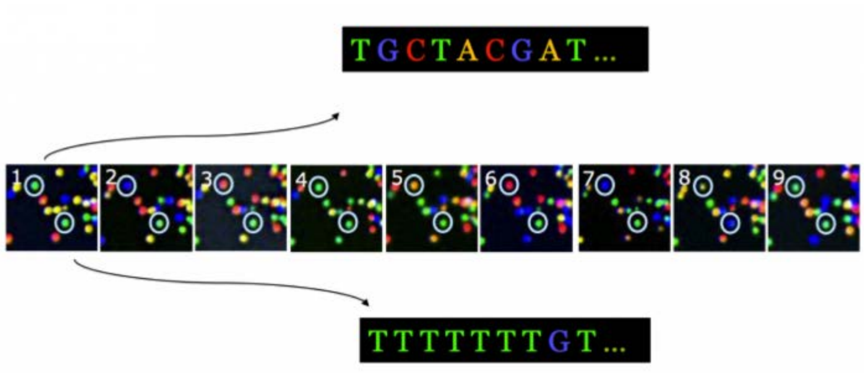

Identified short sequences of a sample are reassembled together by resequencing (using known genome as scaffold), or by *de novo* assembly (in the absence of a reference genome). Next generation sequencing is a universal tool which enables analysis of a genome of any biological organism, particularly any bacterial species.

The description and illustrations of the Illumina sequencing principle are adopted from Illumina, Inc. official site (www.illumina.com).

2. Intended use of the assay

WGS is used for:

- genotyping of the microorganisms
- molecular characterization of bacterial genomes for epidemiological purposes (particularly, antibiotic resistance and virulence genetic determinants detection)
- species identification or identification confirmation based on 16S rRNA sequences derived from WGS data

Other tests, such as PCR, PFGE, and Sanger sequencing, by themselves cannot simultaneously identify, determine the genotype of a pathogen, and detect antibiotic resistance/virulence genes. Whole genome sequencing is performed on MiSeq Illumina platform. The MDL Core Laboratory includes whole genome sequencing utilizing the MiSeq Illumina platform in assisting the identification of bacterial pathogens, epidemiological genotyping, and molecular characterization for epidemiological purposes. Multiple studies have proved the clinical validity of WGS for genotyping of the microorganisms of various species [1-12]. Detection of antibiotic resistance [2,5,10,13-15] and virulence genes [2,5,16] using WGS is also well-established in the literature. The 16S rRNA-based species identification derived from WGS dataset is described previously as well [17,18].

3. Regulations

Whole genome sequencing is considered to be a laboratory-developed test, not cleared or approved by the FDA. "Each laboratory that … introduces a test system not subject to FDA clearance or approval (including methods developed in-house and standardized methods such as textbook procedures), or uses a test system in which performance specifications are not provided by the manufacturer must, before reporting patient test results, establish for each test system the performance specifications for the following performance characteristics, as applicable: (i) Accuracy; (ii) Precision; (iii) Analytical sensitivity; (iv) Analytical specificity to include interfering substances; (v) Reportable range of test results for the test system; (vi) Reference intervals (normal values); (vii) Any other performance characteristic required for test performance" (Reference: Code of Federal Regulations Title 42, Volume3, Part 493.1253 (b)(2)) [19,20].

The performance characteristics defined in CLIA and professional guidance documents do not readily translate to NGS testing practices owing to the complexity of the technology and the informatics analysis required for large-scale genome characterization. Therefore, the **Next-generation Sequencing: Standardization of Clinical Testing (Nex-StoCT) workgroup** (including US Centers for Disease Control and Prevention (CDC), National Human Genome Research Institute, et al.) adapted the definitions of these performance characteristics to better fit the use of NGS in the clinical laboratory (Reference: Gargis et al., Nat Biotechnol, 2012.) [21]. Although the workgroup focused on detection of DNA sequence variations associated with heritable human disorders, many of the principles and recommendations described are also relevant to the application of NGS to other areas of laboratory medicine, including infectious-disease testing. The Nex-StoCT Workgroup considered the requirements of the Clinical Laboratory Improvement Amendments (CLIA) and provided recommendations for validation of clinical NGS tests.

Selected Nex-StoCT Workgroup recommendations for establishing validation of NGS test systems for clinical use:

Requirements for test establishment	Objective	NGS-specific recommendations
Validation	Document reliability of the platform, test, and informatics pipeline before testing of patient specimens	• Platform validation: establish that the system provides reliable sequence analysis across the genomic regions targeted by the test.<br>• Test validation: establish that the system correctly identifies variants in targeted regions of the genome.<br>• Informatics pipeline validation: establish that the algorithm(s) reliably analyze platform data to produce an accurate sequence.
Quality control (QC)	Document reliability of the sequence analysis during patient testing	• Utilize a combination of QC materials, both intrinsic and/or spiked in, that mimic genomic complexity and the types of mutations the test is designed to detect.<br>• During patient testing, quality metrics (for example, quality scores, depth of coverage, uniformity of coverage, mapping quality) should be assessed and compared to those established during validation.
Reference Materials (RMs)	The use of materials for quality management of the analytical phase of testing	• RMs are needed for test validation, QC procedures and the independent assessment of test performance.<br>• Electronic reference data files may serve as RMs for rare or challenging sequence variations.<br>• Efforts should be undertaken to establish a suitable NGS RM and the sequence of the RM should be refined as the technology changes.

This validation examines the following steps:

Performance characteristic	Definition for NGS applications*
Accuracy	The degree of agreement between the nucleic acid sequences derived from the assay and a reference sequence.
Precision	The degree to which repeated sequence analyses give the same result repeatability (within-run precision) and reproducibility (between-run precision).
Analytical sensitivity (Limit of detection)	The likelihood that the assay will detect the targeted sequence variations, if present.
Analytical specificity	The probability that the assay will not detect a sequence variation when none are present (the false positive rate is a useful measurement for sequencing assays).

* The definitions of CLIA performance characteristics for NGS were suggested by the Nex-StoCT Workgroup

According to Nex-StoCT Workgroup, the degree of agreement can be established by determining the proximity of agreement between a measured value and the true value, which for NGS is the accepted by National Center for Biotechnology Information (NCBI) reference sequence. As per Clinical and Laboratory Standards Institute (CLSI), "since typing procedures typically vary for different species, a standard strain of the species under study is generally appropriate" [22]. CLSI suggests downloading complete reference sequences "from publicly available sites such as … the National Center for Biotechnology Information (http://www.ncbi.nlm.nih.gov/genome/)" (Reference: Clinical and Laboratory Standards Institute (CLSI). Nucleic Acid Sequencing Methods in Diagnostic Laboratory Medicine: Approved Guideline- 2d edition. MM09-A2, 2014) [22].

Proficiency testing is available from two sources and will be used in future for the external quality assurance:

- CDC PulseNet certification set of the strains of *Escherichia coli* O157:H7, *Salmonella enterica* serotype Heidelberg, *Listeria monocytogenes,* and *Campylobacter jejuni*.
- Proficiency test available from Global Microbial Identifier (GMI) (http://www.globalmicrobialidentifier.org/) for *Salmonella enterica*, *Escherichia coli,* and *Staphylococcus aureus* (Reference: Moran-Gilad et al., BMC Infectious Diseases, 2015.) [23].

4. Acceptable and not acceptable specimens

4.1. Biosafety Risk group 2 specimens

4.1.1. Acceptable specimens for microorganisms belonging to Biosafety Risk group 2

- **Bacterial DNA extract**: Samples in the form of purified DNA can be received from other CDPH laboratories or from outside collaborators. DNA extract should be resuspended in buffer without Ethylenediaminetetraacetic acid (EDTA).
- **Pure bacterial culture**: live bacterial culture in the broth, motility agar, on agar plate/slant, or in storage media can be submitted to the Core lab by other CDPH laboratories on campus. From collaborators outside of CDPH live bacterial culture can be accepted only in motility agar, agar slant, or storage media in tubes with lids providing a leak-tight seal. Lyophilized cultures can be accepted from both CDPH laboratories on campus and collaborators outside of CDPH.

Following microorganisms belonging to Biosafety Risk group-2 will be processed by the Core laboratory:

--*Acinetobacter baumannii* (formerly *Acinetobacter calcoaceticus*)
--*Actinobacillus*
--*Actinomyces spp.*
--*Aeromonas hydrophila*
--*Bartonella henselae, B. quintana*
--*Campylobacter coli, C. fetus, C. jejuni*
--*Clostridium difficile*, *C. perfringens*, *Clostridium* spp. *(*excluding *C. tetani, C. botulinum)*
-- Diphtheroids *(C. bovis, C. xerosis, C. pyogenes, C. haemolyticum, C. pseudotuberculosis, C. ulcerans)*
--*Edwardsiella tarda*
--*Enterobacter* spp.
--*Enterococcus faecalis* and *Enterococcus faecium*
--*Escherichia coli* - all enteropathogenic, enterotoxigenic, enteroinvasive and strains bearing K1 antigen, including *E. coli* O157:H7
--*Haemophilus ducreyi, H. influenzae* non-b type
--*Helicobacter spp.*
--*Klebsiella* - all species except *K. oxytoca* (RG1)
--*Legionella* including *L. pneumophila*
--*Leptospira spp.*
-- *Listeria monocytogenes*
-- *Moraxella* spp.
-- *Mycobacterium leprae*
-- *Mycobacterium spp. (other than M. tuberculosis complex and M. leprae)*
--*Neisseria gonorrhoeae*
--*Nocardia asteroides, N. brasiliensis, N. otitidiscaviarum, N. transvalensis*
--*Pseudomonas aeruginosa*

--*Salmonella* including *S. arizonae, S. choleraesuis, S. enteritidis, S. gallinarum-pullorum, S. meleagridis, S. paratyphi,* A, B, C, *S. typhi, S. typhimurium*
--*Shigella* including *S. boydii, S. dysenteriae,* type 1, *S. flexneri, S. sonnei*
--*Serratia* spp.
--*Staphylococcus aureus*
--*Streptobacillus moniliformis*
--*Streptococcus* spp., *S. pyogenes*
--*Treponema pallidum*
--*Vibrio cholerae, V. parahaemolyticus*
--*Yersinia enterocolitica*
(List of Risk group 2 pathogens adopted from Biosafety in Microbiological and Biomedical Laboratories (BMBL), 5th Edition, 2009 [24])

4.1.2. Not acceptable specimens for microorganisms belonging to Biosafety Risk group-2

- Patient's material (stool, blood, serum, plasma, tissue, sputum, etc.)
- Blood culture bottle
- DNA extracts resuspended in buffer containing EDTA

4.2. Biosafety Risk group 3 specimens

4.2.1. Acceptable specimens for *Mycobacterium tuberculosis*

- **Bacterial DNA extract**: DNA extracts from *Mycobacterium tuberculosis* are processed by the Core laboratory. Samples in a form of purified DNA can be received from other CDPH laboratories or from outside collaborators. DNA extract should be resuspended in buffer without Ethylenediaminetetraacetic acid (EDTA).

4.2.2. Not acceptable specimens for *Mycobacterium tuberculosis*

- Pure bacterial culture in a liquid, semiliquid, or on solid media
- Pure bacterial culture in storage media
- Patient's material (sputum, etc.)
- DNA extracts resuspended in buffer containing EDTA

4.2.3. Not acceptable specimens for other Biosafety Risk group 3 organisms

Select agents cannot be processed by the personnel who are not cleared for the handling of the select agents. In the case of exceptional public health emergency this policy can be overturned by Lab Director.

5. Positive control

Use PhiX control in each run as a positive control. When sequencing is complete, read MiSeq reporter % error value which reflects sequencing accuracy for PhiX. This value should be < than 6%.

10 nM PhiX library control v.3 is ordered from Illumina, Cat. No. FC-110-3001.

Preparation of positive control and incorporation into the assay is described in Chapter "Manual Library Normalization and Pooling" of Standard Operating Procedure CORE-PROC_WGS _001.

6. Negative control

6.1. Negative control for sequencing process

To control for contamination during the library preparation and sequencing process, index combination which doesn't correspond to any sample in the current sequencing run should be added to the indexes demultiplexing step. Index combination for negative control should correspond to one of the index combinations used in the previous sequencing run, this way it would capture carry-over contamination with the library fragments generated in the previous run. Thresholds for negative control:

**Parameter**	**Required value for negative control**
Number of reads after trim	<10,000
N50 for *de novo* assembled reads	< 1,000
The highest coverage of *de novo* assembled contigs	< 10x

6.2. Negative control for sequencing analysis

In the case of epidemiological typing, unrelated strain of the same species as pathogen caused potential outbreak should be included in the analysis as a negative control. A sequence of epidemiologically unrelated negative control can be acquired from SRA NCBI database (http://www.ncbi.nlm.nih.gov/sra/) or European Nucleotide Archive (http://www.ebi.ac.uk/ena).

7. Quality assurance & Quality control

Preliminary quality thresholds were selected based on literature data and previous sequencing experience. The quality thresholds were used to determine when to exclude failed validation sequencing results from the analysis and to repeat sequencing. The preliminary quality metrics have to be reviewed after validation is completed and adjusted in accordance with acquired data.

7.1. Each run QC

At every run QC must be performed by monitoring following quality metrics:

a) quality of the input DNA for all tested samples
b) quantity of the input DNA for all tested samples

c) DNA library size distribution for representative samples
d) DNA library concentration for all tested samples
e) quality of the sequences for all tested samples
f) quality of spiked in positive PhiX control sequences
g) quality of negative sequencing process/analysis control.

Ensure proper record keeping for quality assurance. Mark all manipulations performed during the samples processing starting from the moment of reception of cultures/DNA samples till completion of sequencing analysis by filling out the Core Lab Tracking form and MiSeq Data. Indicate if any changes which have been introduced to the protocol. Note if quality metrics weren't met and any corrective measures were undertaken in Comments sections in the corresponding chapter of the Tracking form (metrics a-d) or in the corresponding field of MiSeq Data log (metrics e-g) .

7.1.1. Input DNA and DNA library quality and quantity metrics

Quality of the input DNA for all tested samples is estimated via ratio of absorbance at 260 nm to absorbance at 280 nm. A value of 260/280 absorbance must be >1.7.

Quantity of the input DNA for all tested samples is measured using Qubit fluorometer and corresponding reagents kit. DNA concentration should be ≥1ng/μl.

If abovementioned quality parameters are not met, repeat and troubleshoot DNA isolation step.

7.1.2. DNA library quality and quantity metrics

DNA library size distribution for representative samples must be measured using BioAnalyzer instrument and corresponding reagents kits. Representative samples should include the following variety (if present within the run): different species, Gram-positive and Gram-negative bacteria, and species with different GC content. An average size of the library must be within the range of 300bp-3kb.

DNA library concentration is measured using Qubit fluorometer and corresponding reagents kit and must be ≥ 1nM.

If abovementioned quality parameters are not met, repeat and troubleshoot tagmentation/amplification/post-PCR cleanup steps of library preparation.

In some cases BioAnalyzer run failure leads to missing or shifted library peaks, in this case repeat BioAnalyzer run.

7.1.3. Quality metrics of the tested samples sequences

Quality metrics of tested samples must meet the following parameters [preliminary quality thresholds]:

- Percent of bases with quality score >Q30 for the run must be ≥ 50%
- Q30 score for generated genome sequences must be ≥75% for at least 80bp of the read length.
- Average depth of coverage must be ≥ 10x across the whole genome

If abovementioned quality parameters are not met, repeat and troubleshoot library preparation and library pooling/loading.

7.1.4. Quality metrics of spiked in positive PhiX control sequences

[Preliminary] quality threshold for spiked-in positive control:

- PhiX error rate must be <6%

If abovementioned quality parameter is not met, verify expiration dates of PhiX stock and storage conditions, check that used denatured 20 pM PhiX library was within 3 weeks of preparation. Troubleshoot library denaturation and library pooling/loading. If the problem persists, contact Illumina tech support, hence the high PhiX error rate might be caused by MiSeq hardware malfunction.

7.1.5. Quality metrics of negative control sequences

7.1.5.1. Quality metrics of negative control sequences for sequencing process

Negative control of sequencing process represents an index combination which doesn't correspond to any sample in the current sequencing run but matches one of the index combinations used in the previous sequencing run. If negative control doesn't meet [preliminary] quality parameters below, this indicates a possibility of carry-over contamination with the library fragments generated in the previous run:

- Number of reads after trim must be <10,000
- N50 for *de novo* assembled reads must be < 1,000
- The highest coverage of de novo assembled contigs must be < 10x

Perform template line wash of MiSeq instrument with 0.01% sodium hypochlorite and clean all working surfaces with 10% bleach in a case of failure to meet abovementioned quality parameters.

7.1.5.2. Quality metrics of negative control for sequencing analysis

Epidemiologically unrelated negative control should not cluster with tested samples on the phylogenetic tree. If negative control clusters together with epidemiologically unrelated samples:

a) Repeat analysis; b) Ensure that coverage of all samples meets minimum quality parameters (see chapter 7.1.3); c)Use different Reference sequence for mapping if available; d) Add additional epidemiologically unrelated negative control for sequencing analysis.

7.2. Monthly QC

7.2.1. Monthly positive QC control

Perform monthly QC testing by including control *Escherichia coli* ATCC 25922 genome into library pool as the last sample. Perform assembly of *E.coli* ATCC 25922 genome. Sequences for monthly QC control must meet following [preliminary] metrics:

- Average coverage of the genome must be ≥ 10x.
- Q30 score for *E.coli* ATCC 25922 genome sequence must be ≥75% for at least 80bp of the read length.

Following assays results must be acquired for monthly QC control:

- MLST allelic profile acquired during the run must correspond to known ST73 profile described for this strain.

- 16S rRNA sequence must be identified as *Escherichia coli*
- No antibiotic resistance genes should be found with ResFinder analysis
- Following virulence genes should be found using VirulenceFinder analysis with 100% ID and 100% query length coverage: *mchB, mchC, iss, mchF, mcmA, iha, sat, vat, iroN.*

7.2.2. Monthly negative QC control

Perform monthly QC testing by including no-template (water) as a negative quality control for detection of reagents contamination. Start with the step of DNA isolation, for DNA resuspension use Tris HCl buffer, which was used in previous runs or prepare new buffer using MilliQ water source in the lab. Measure DNA concentration in the negative sample using NanoDrop and Qubit. Proceed through all library preparation steps. Measure library concentration and size distribution using Qubit and Bioanalyzer.

Negative quality control sample must meet following parameters:

- Genomic DNA concentration in the negative sample according to NanoDrop and Qubit must be 0±0.1ng/µl
- Library concentration for the negative sample according to Qubit must be <0.5ng/µl
- Bioanalyzer must show no peak for the negative sample
- If library concentration for the negative sample is >0.5ng/µl and/or Bioanalyzer shows a peak on the electrophoregram- load sample for sequencing. For pooling take the volume of the negative sample which corresponds to the average volume of other samples. Quality metrics of the sequencing data for negative sample must meet following [preliminary] parameters:
  - Number of reads after trim must be <10,000
  - N50 for de novo assembled reads must be < 1,000
  - The highest coverage of de novo assembled contigs must be < 10x

Record values for Monthly Positive and Negative controls into both MiSeq data log for the run and into the "Monthly QC log". Start a new Monthly QC log spreadsheet every calendar year.

7.3. Equipment calibration

Following equipment must be calibrated every 6 months:

- NanoDrop- see Appendix 20 of Standard Operating Procedure CORE-PROC_WGS _001.
- PCR thermocycler- see Appendix 21 of Standard Operating Procedure CORE-PROC_WGS _001.

Following equipment must be calibrated at every run:

- Qubit Fluorometer: by using 2 standards included in the kit.
- Agilent Bioanalyzer: by spiking in markers in each sample and including the ladder in each chip run. Markers and ladder are provided with the kit.

Thermometers for heat blocks and refrigerators must be calibrated once a year.

7.4. Test Rejection Criteria

Test results should be rejected and test must be troubleshot and repeated if:

- Read length at which 75% of bases have quality score ≥Q30 is less than 80bp
- Average depth of coverage is < 10x across the whole genome
- Calculated % error value for spike in PhiX is >6%
- Percent of bases with quality score >Q30 for the run is < 50%
- Negative control met two or more of the following parameters: ≥10,000 reads after trim, N50 ≥1,000, or highest coverage of *de novo* assembled contigs >10x

Data need to be re-analyzed if:

- Negative control clusters with epidemiologically unrelated samples on the phylogenetic tree

Monthly QC test needs to be repeated and procedure troubleshot if:

- The length of *E.coli* ATCC 25922 reads at which 75% of bases have quality score ≥Q30 is less than 80bp
- Average coverage of the *E.coli* ATCC 25922 genome is < 10x
- MLST allele profile of control *E.coli* ATCC 25922 genome sequence doesn't correspond to ST73 profile or identification failed
- 16S rRNA sequence identification in control *E.coli* ATCC 25922 genome failed or identification results didn't match *Escherichia coli*
- In the case of antibiotic resistance characterization, sequence of *Escherichia coli* ATCC 25922 upon analysis contains any known antibiotic resistance genes.
- Sequence of *Escherichia coli* ATCC 25922 upon analysis is missing virulence genes mentioned in Chapter 7.2.1 or has additional virulence genes [at 100% ID, 100% query length coverage].
- Negative monthly control results in the sequencing reads with above threshold quality metrics (see Chapter 7.2.2)

8. Validation

8.1. Validation samples

For the WGS validation, the ATCC bacterial strains or strains previously sequenced by CDC were sequenced by the MDL Core laboratory (further referred to as Core lab) using Illumina MiSeq sequencer. Reference strains with available whole genome sequences from NCBI or CDC were included in the validation in order to compare validation sequencing results with reference sequencing results. Reference strains used in validation of WGS by the Core lab are referred to as “validation samples”. Validation samples included 34 bacterial samples for whole genome sequencing:

10 *Enterobacteriaceae* bacterial samples
5 gram-positive cocci bacterial samples
5 gram-negative non-fermenting bacterial samples
9 *Mycobacterium tuberculosis* samples
5 representatives of miscellaneous bacterial species.

Sequences generated by Core laboratory by performing WGS of validation samples are referred to in this document as “validation sequences” (in opposite of “reference sequences” acquired from NCBI or CDC). Phylogenetic tree generated from validation sequences is referred to as “validation tree”. Validation samples repeated within/between runs are referred to as “validation replicates”.

8.2. Reference materials

Complete reference sequences of standard strains of the representative species were downloaded from the publicly available website of the National Center for Biotechnology Information (http://www.ncbi.nlm.nih.gov/genome/) to serve as reference materials.

Reference materials in this validation study are represented by:

1) The complete genome of the same ATCC strain which was sequenced by the laboratory was used when genome of the strain sequenced by the laboratory is available from NCBI database.
2) The complete genome of a strain belonging to the same species as tested by the laboratory isolates, though not identical to the strain sequenced by the Core lab, was used when genome of the strain sequenced by the laboratory is NOT available from NCBI database.
3) Raw reads generated by CDC for the same strains which were also sequenced in the Core lab.

In the cases #1 and #2, the reference was downloaded from NCBI Genome database and used as a reference for mapping of the Core lab- generated sequences. SNP differences between laboratory replicates are shown in both cases. SNP differences between the reference sequence and Core lab sequences are analyzed only for the case #1.

In the case #3, CDC reference raw reads were trimmed and mapped to the same complete genome scaffold as was used for mapping of Corelab sequences. The number of SNP differences between the laboratory sequences and CDC-generated sequences was determined.

**Reference materials**		**Reference**	
**Core lab ID**	**Sample**	**NCBI Strain name**	**NCBI Acc#**
C1	*Escherichia coli* O157:H7 CDC EDL 933	O157:H7 CDC EDL 933	NZ_CP008957.1
C3	*Escherichia coli* ATCC 8739	ATCC 8739	NC_010468.1
C55	*Escherichia coli* ATCC 25922	ATCC 25922	NZ_CP009072.1
C4	*Enterobacter cloacae* ATCC 13047	ATCC 13047	NC_014121
C6	*Salmonella enterica* ser Typhimurium ATCC 14028	14028S	NC_016856
C5	*Staphylococcus aureus* ATCC 25923	ATCC 25923	NZ_CP009361
C46	*Enterococcus faecalis* ATCC 29212	ATCC 29212	NZ_CP008816
C47	*Staphylococcus epidermidis* ATCC 12228	ATCC 12228	NC_004461
C48	*Staphylococcus saprophyticus* ATCC 15305	ATCC 15305	NC_007350
C49	*Streptococcus pneumoniae* ATCC 6305	ATCC 700669	FM211187
C50	*Pseudomonas aeruginosa* ATCC 27853	FRD1	NZ_CP010555
C51	*Stenotrophomonas maltophilia* ATCC 13637	ATCC 13637	NZ_CP008838
C52	*Legionella pneumophila* SG-12 ATCC 43290	ATCC 43290	NC_016811
C53	*Moraxella catarrhalis* 87A-3084	ATCC 25240	NZ_CP008804
C54	*Acinetobacter baumannii* ATCC 17945	AB07	NZ_CP006963
C103	*Bacteroides fragilis* ATCC 25285	638R	NC_016776
C104	*Haemophilus influenzae* ATCC 10211	KR494	NC_022356
C2	*Aeromonas hydrophilia* ATCC 7966	ATCC 7966	NC_008570
C105	*Corynebacterium jeikeium* ATCC 43734	ATCC 43734	GG700813:GG700833
C106	*Neisseria gonorrhoeae* ATCC 49226	MS11	NC_022240
C56	*Mycobacterium tuberculosis*	H37Rv	NC_000962.3
C57	*Mycobacterium tuberculosis*	H37Rv	NC_000962.3
C58	*Mycobacterium tuberculosis*	H37Rv	NC_000962.3
C59	*Mycobacterium tuberculosis*	H37Rv	NC_000962.3
C61	*Mycobacterium tuberculosis*	H37Rv	NC_000962.3
C65	*Mycobacterium tuberculosis*	H37Rv	NC_000962.3
C67	*Mycobacterium tuberculosis*	H37Rv	NC_000962.3
C68	*Mycobacterium tuberculosis*	H37Rv	NC_000962.3
C69	*Mycobacterium tuberculosis*	H37Rv	NC_000962.3

Reference materials- CDC strains

		Reference raw reads generated by CDC		Reference used for mapping	
**Core lab ID**	**Species**	**CDC Strain name**	**Accession #**	**NCBI Strain**	**NCBI Acc#**
C72	*Escherichia coli* O121:H19	2014C-3857	SRR1610033	O104:H4 str. 2011C-3493	NC_018658
C73	*Salmonella enterica* ser Enteritidis	CDC_2010K-1543	SRR518749	Enteritidis, str P125109	NC_011294.1
C74	*Salmonella enterica* ser Infantis	2014K-0434	SRR1616809	Infantis, str 1326/28	NZ_LN649235
C75	*Salmonella enterica* ser Adelaide	2014K-0941	SRR1686419	Enteritidis, str P125109	NC_011294.1
C76	*Salmonella enterica* ser Worthington	2012K-1219	SRR1614868	Enteritidis, str P125109	NC_011294.1
C77*	*Salmonella enterica* ser Saintpaul	2014K-0875	SRR1640105	Typhimurium, str 14028S	NC_016856

Green color designates cases when the genome of the strain sequenced by the Core lab is available from the NCBI database. Yellow color designates cases when the genome of the strain sequenced by the Core lab is NOT available from the NCBI database and an alternative reference genome was used for mapping.

*P.S.: Sample C77 was sequenced by Core lab only for genotyping assay accuracy validation. No replicates were done.

Thirty-four validation samples were sequenced in triplicate (within and between runs). For between run reproducibility assessment, all replicates were generated starting from fresh culture (exception: replicates for *Mycobacterium tuberculosis* samples were generated starting from DNA). Between run replicates were processed on different days, altering two operators (See testing schedule in Appendix 7). For within run replicates one DNA extract was used, but independent library preparations were done, with final samples being included in one sequencing run.

Data analysis is described in Appendix 5.

Confirmatory testing has to be performed if 16S rRNA ID or MLST results for validation samples don't match the reference results, by performing Sanger sequencing of 16S rRNA gene or MLST housekeeping genes, correspondingly. Confirmatory testing should be performed if results of antibiotic genes detection with WGS don't match the reference results, by performing antibiotic susceptibility testing via micro-broth dilution or disc-diffusion methods. All discrepancies must be recorded.

8.3. Accuracy

In accuracy of WGS can be divided into three components: accuracy of platform, assay accuracy, and accuracy of bioinformatics pipeline.

8.3.1. MiSeq Platform Accuracy

Platform accuracy is assessed as the accuracy of identification of individual base pairs in a bacterial genome. We determined MiSeq Illumina platform accuracy by comparing base calling results with the reference sequence.

Also, we validated quality parameters, which affect platform accuracy and determined ranges, which allow accurate identification of individual base pairs. The preliminary quality thresholds were adjusted based on validation data to match the most stringent values of quality parameters which were detected during the validation (±5%). In several cases, the threshold was left at the level which was even more stringent than any of the detected values.

8.3.1.1. Quality parameters affecting platform accuracy

We have identified the quality parameters of the sequencing data affecting platform accuracy and established quality parameters thresholds, which provide ≥ 90% accuracy of base calling. Following types of errors affect the MiSeq Illumina accuracy of the platform: a) sequence errors introduced by DNA library preparation technique (e.g. amplification-introduced errors); and b) base calling accuracy of the sequencer. The set of quality parameters to account for corresponding types of errors were established.

a) Sequence errors introduced by DNA library preparation technique:

The first type of sequencing errors which are introduced by PCR errors during libraries amplification are stochastic and independently performed library preps are not likely to have the same errors. High depth and good uniformity of coverage reduce impact of sequencing errors [25]. For that reason the thresholds for depth and uniformity of coverage providing accurate variant calling were determined empirically during this validation:

–<u>Average depth of coverage</u> must be ≥ 15x across genome. The minimum coverage of 15x was achieved for targeted areas used in gene-specific analysis: MLST scheme genes, 16S rRNA gene. If the minimum coverage threshold is not achieved for targeted areas, an alternate method such as Sanger sequencing should be used for sequencing of given genome region.

<u>Uniformity of coverage</u>: >50% of positions on the target (coding sequence) should have coverage ≥10x and >70% of positions should have coverage ≥5x.

b) Base calling accuracy of the sequencer:

The second type of errors, determined by the accuracy of base calling of the sequencer. The parameter used to estimate the accuracy of the base calling by the platform is the Phred quality score, which reflects a probability of incorrect base call. For Illumina sequencing platform the Phred score of Q30 is generally used and

it corresponds to a probability of 1 incorrect base call in 1,000 [26]. The MiSeq sequencer specifications cited on the internet site of the manufacturer suggests a following base calling accuracy, measured by the Phred quality score (Q score): > 70% bases in 300bp-long fragments should have Phred score higher than Q30, while it is noted that “actual performance parameters may vary based on sample type, sample quality, and clusters passing filter”.

While error rate in spiked in PhiX sequence is another quality metrics reflecting calling accuracy of the sequencer, it is not recommended for use as a sole quality control for platform accuracy [27]. For that reason we used error rate of the PhiX as an addition to other metrics in order to monitor platform base calling accuracy.

To account for base calling accuracy of the sequencer we evaluated following quality metrics:

Accuracy of base calling: Sequence reads must have ≥ Q30 for more than 75% bases for at least 86bp of the read length. The average read length after trimming and discarding the base pairs with quality score <Q30 should be >109bp.

PhiX error rate: PhiX error % reflects sequencing accuracy for positive control PhiX spiked into each run. The PhiX error rate for the run should be <4.9%.

Sequencer run metrics were assessed to establish the optimal performance of the platform:

Percent of bases with the quality score >Q30 for the run- MiSeq sequencer base calling accuracy metrics for the run. Should be > 57% for the 600 cycles MiSeq reagents.

Cluster density for the run- density of clusters formed by clonally amplified library fragments on the flow cell surface. Should be >800 K/mm^2, Maximum 1700K/mm^2. While preferably to have cluster density within the range of 800-1100 K/mm^2.

Cluster passing filter of the run – percentage of clusters that pass quality filter for the purity of the signal. Should be >72%

Below see the ranges established for the corresponding data metrics. The graphs represent a distribution of values by the number of samples with certain value.

Average read length with ≥ Q30 (= avg read length after trimming) = 121.5-156bp (median 139.05bp)

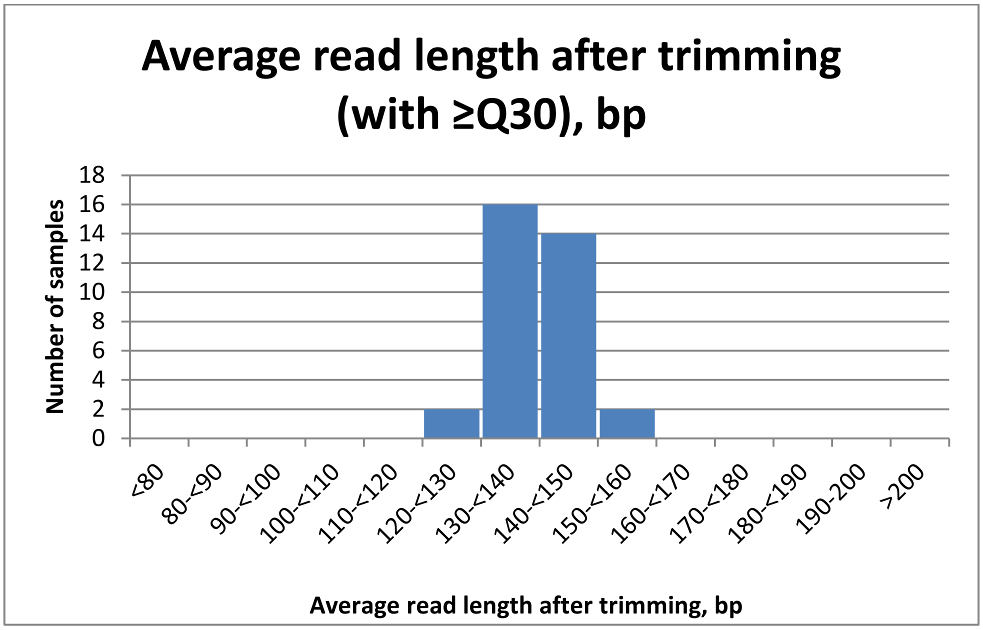

Read length at which 75% of bases have quality score ≥Q30 = 120-225bp (median 150bp)

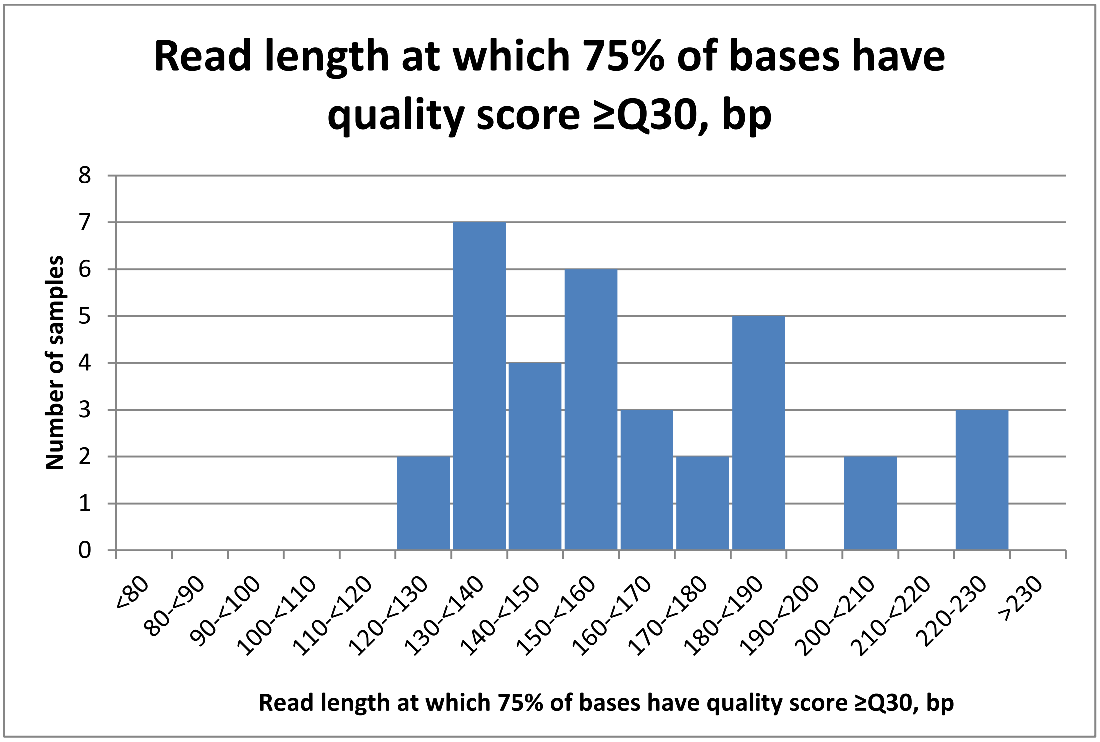

Average depth of coverage of genome= 30.26-216.4x (median 69.45x)

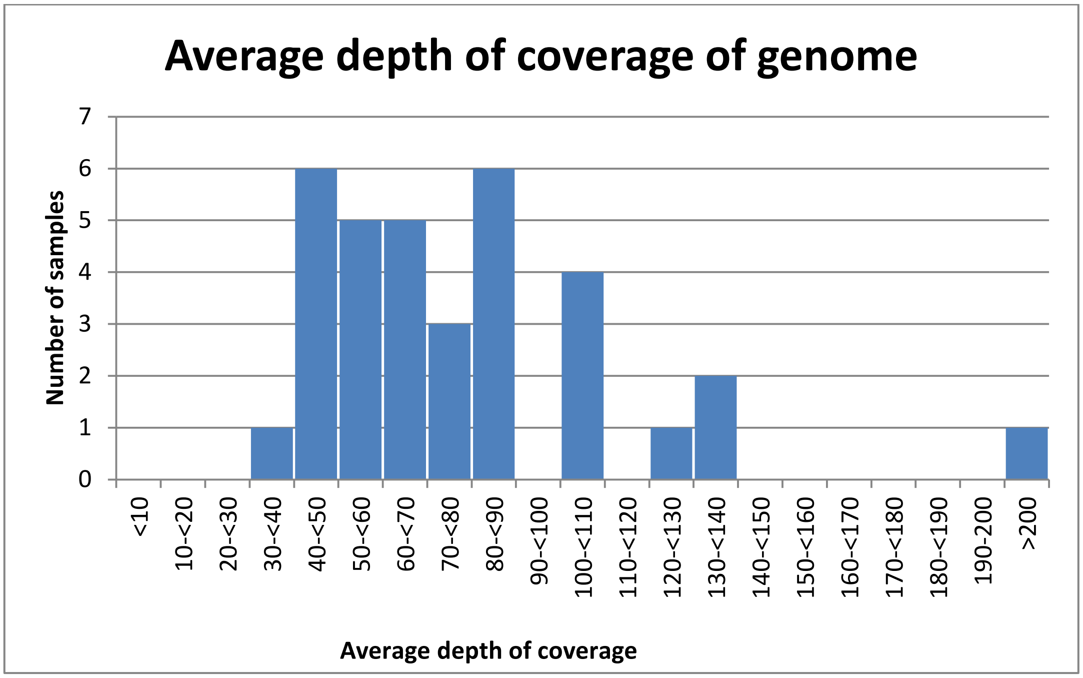

Percentage of genome covered (after mobile elements masking)= 85-100% (median 97%)

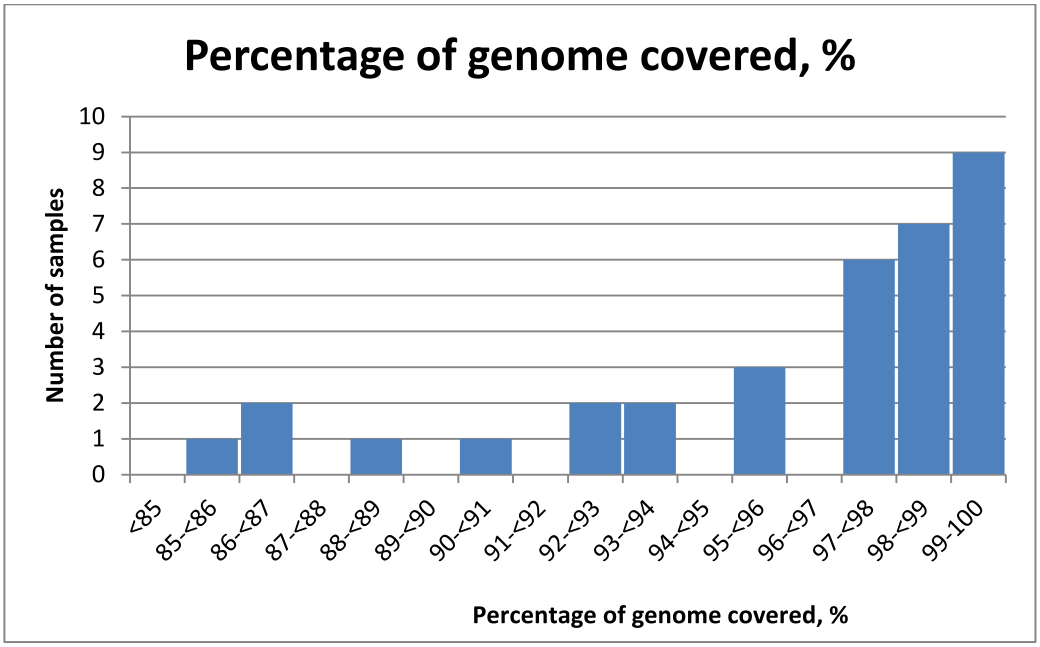

Uniformity of coverage at 10x (Percent of positions with coverage ≥10x)= 61.74-99.91% (median 95.19%)

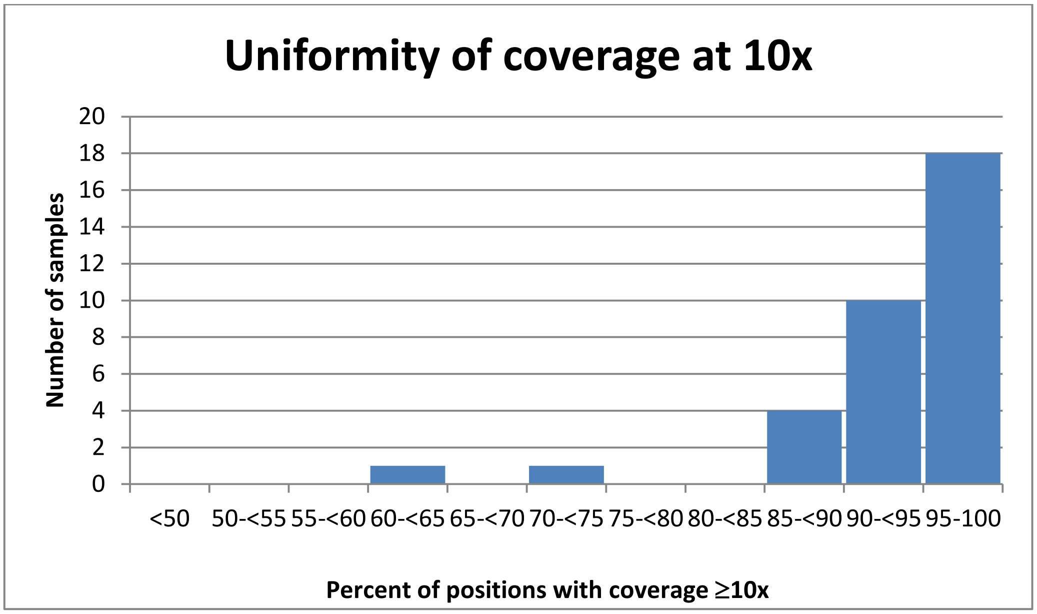

Uniformity of coverage at 5x (Percent of positions with coverage ≥5x)= 74.72-99.96% (median 96.16%)

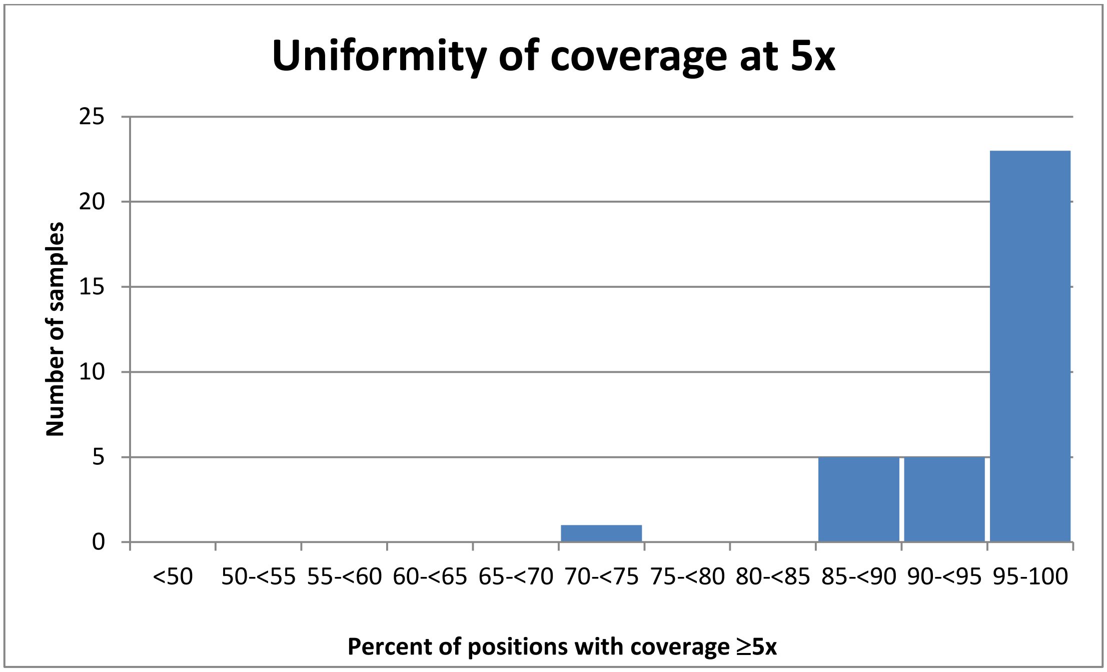

PhiX error rate =3.09-4.74% (median 3.51%)

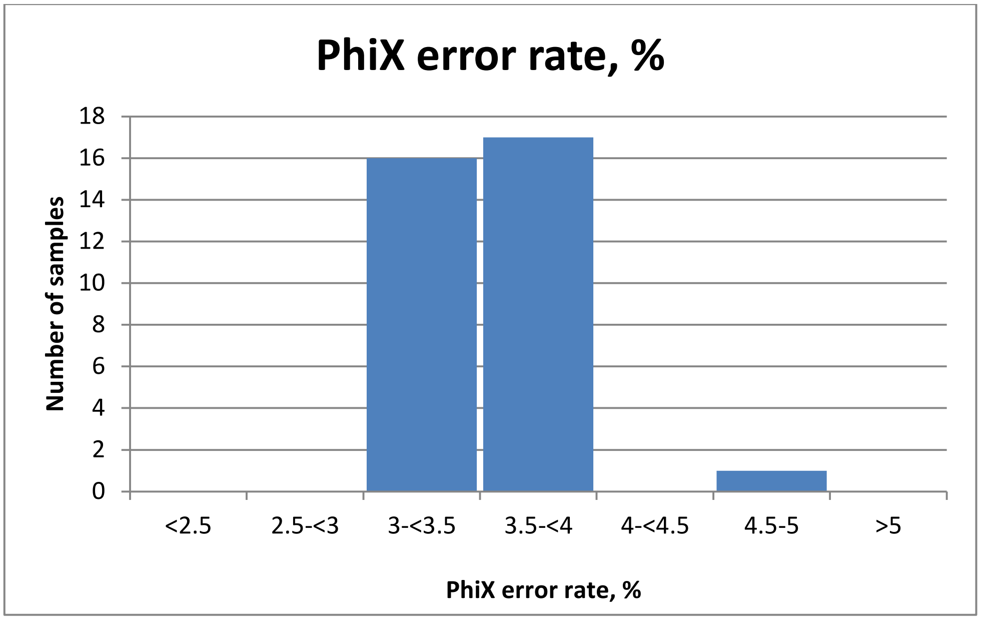

Percent of bases with quality score >Q30 for the run = 59.9-63.3% (median 60.70%)

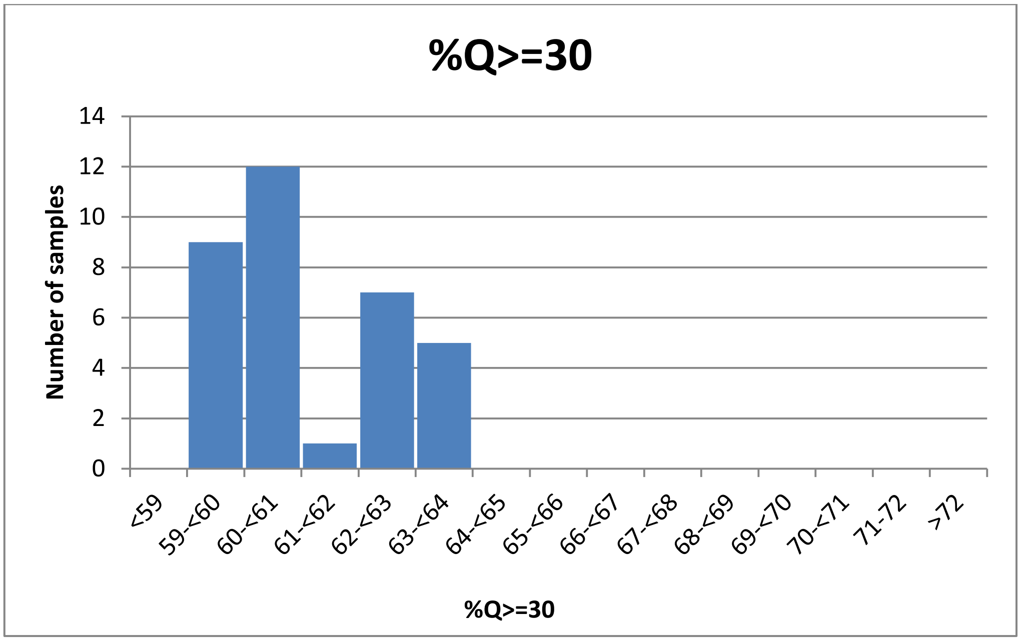

Cluster density for the run = 832-1693K/mm^2 (median 1272 K/mm^2)

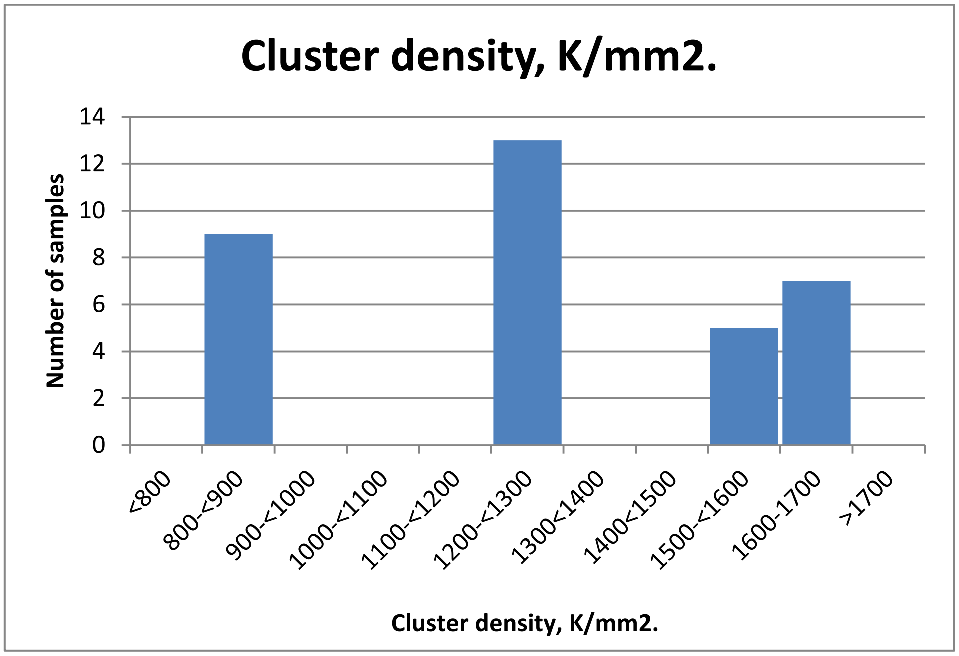

Cluster passing filter of the run = 76.1-93.2% (median 88.9%)

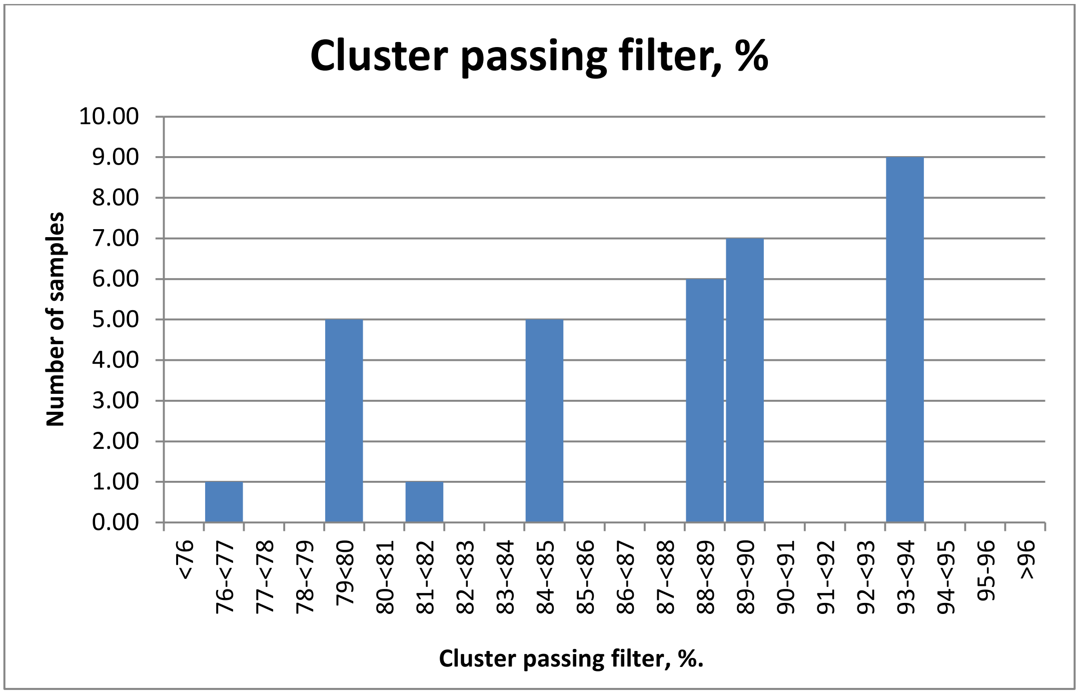

Quality parameters for validation samples:

Sample	Species	Average read length with ≥ Q30 (= avg read length after trimming), (bp)	Read length at which 75% of bases have quality score ≥Q30, (bp)	Average depth of coverage of genome	Percentage of genome covered (after mobile elements masking)	Uniformity of coverage at 10x	Uniformity of coverage at 5x	PhiX error rate	Percent of bases with quality score >Q30 for the run	Cluster density for the run, (K/mm^2)	Cluster passing filter of the run
C1	*Escherichia coli*	134.1	160	63.68	93	88.75%	88.93%	3.87%	61.1	1651	81.2%
C2	*Aeromonas hydrophilia*	132.3	140	85.69	100	99.66%	99.80%	3.67%	60.7	1272	88.9%
C3	*Escherichia coli*	139.6	170	86.54	99	99.70%	99.76%	3.57%	63.3	1505	84.3%
C4	*Enterobacter cloacae*	136.5	160	73.76	98	99.41%	99.48%	3.57%	63.3	1505	84.3%
C5	*Staphylococcus aureus*	156	225	216.40	98	99.75%	99.75%	3.57%	63.3	1505	84.3%
C6	*Salmonella enterica* ser Typhimurium	135.9	160	85.98	99	98.76%	98.81%	3.57%	63.3	1505	84.3%
C46	*Enterococcus faecalis*	144.7	200	104.33	100	99.69%	99.77%	3.33%	62.8	1203	89.1%
C47	*Staphylococcus epidermidis*	146.5	225	106.56	100	98.35%	99.47%	3.33%	62.8	1203	89.1%
C48	*Staphylococcus saprophyticus*	150.1	220	134.57	100	99.91%	99.94%	3.33%	62.8	1203	89.1%
C49	Streptococcus pneumoniae	132.8	185	105.38	88	86.27%	86.56%	4.74%	60.2	1693	76.1%
C50	*Pseudomonas aeruginosa*	121.5	120	47.50	92	91.17%	92.38%	3.33%	62.8	1203	89.1%
C51	*Stenotrophomonas maltophilia*	130.8	125	55.32	100	99.43%	99.58%	3.67%	60.7	1272	88.9%
C52	*Legionella pneumophila*	145.9	205	120.1	100	99.71%	99.76%	3.33%	62.8	1203	89.1%
C53	*Moraxella catarrhalis*	137.4	180	105.68	95	95.11%	95.18%	3.33%	62.8	1203	89.1%
C54	*Acinetobacter baumannii*	143.4	180	83.58	86	74.48%	74.72%	3.33%	62.8	1203	89.1%
C55	*Escherichia coli*	142.7	175	67.26	97	99.62%	99.73%	3.57%	63.3	1505	84.3%
C56	*Mycobacterium tuberculosis*	143.9	135	46.48	97	94.14%	95.53%	3.09%	59.9	832	93.2%
C57	*Mycobacterium tuberculosis*	144.0	135	41.60	97	93.23%	95.48%	3.09%	59.9	832	93.2%
C58	*Mycobacterium tuberculosis*	147.3	135	57.07	97	95.26%	95.99%	3.09%	59.9	832	93.2%
C59	*Mycobacterium tuberculosis*	148.1	150	68.43	98	95.71%	96.33%	3.09%	59.9	832	93.2%
C61	*Mycobacterium tuberculosis*	145.4	135	43.38	97	93.35%	95.21%	3.09%	59.9	832	93.2%
C65	*Mycobacterium tuberculosis*	142.0	135	49.72	98	61.74%	89.63%	3.09%	59.9	832	93.2%
C67	*Mycobacterium tuberculosis*	146.5	140	48.69	98	95.78%	96.51%	3.09%	59.9	832	93.2%

C68	*Mycobacterium tuberculosis*	144.7	135	52.37	98	91.59%	95.90%	3.09%	59.9	832	93.2%
C69	*Mycobacterium tuberculosis*	142.6	135	30.26	98	94.81%	96.55%	3.09%	59.9	832	93.2%
C72	*Escherichia coli O121:H19*	136	155	54.32	86	89.24%	89.54%	3.51%	60.4	1612	79.1%
C73	*Salmonella enterica ser* Enteritidis	137.8	150	64.16	99	98.74%	98.77%	3.51%	60.4	1612	79.1%
C74	*Salmonella Infantis*	138.4	145	62.01	97	98.22%	98.31%	3.51%	60.4	1612	79.1%
C75	*Salmonella Adelaide*	135.1	150	87.92	92	92.78%	92.85%	3.51%	60.4	1612	79.1%
C76	*Salmonella Worthington*	133.4	150	55.24	93	93.70%	93.85%	3.51%	60.4	1612	79.1%
C103	*Bacteroides fragilis*	138.5	180	85.05	85	85.47%	85.68%	3.67%	60.7	1272	88.9%
C104	*Haemophilus influenzae*	137.9	185	134.95	90	91.80%	91.95%	3.67%	60.7	1272	88.9%
C105	*Corynebacterium jeikeium*	130.4	150	70.47	95	99.82%	99.96%	3.67%	60.7	1272	88.9%
C106	*Neisseria gonorrhoeae*	127.1	140	71.55	95	93.24%	93.82%	3.67%	60.7	1272	88.9%

See details on acquisition of parameters in the table in Appendix 2.

8.3.1.1.1. Quality parameters of positive and negative controls.

Quality thresholds of positive and negative controls have been revised based on data acquired during validation.

8.3.1.1.1.1. Negative control for sequencing process (each-run QC)

The preliminary metrics are more stringent than acquired data (The results for negative controls included into the validation runs are summarized in the Appendix 12). Preliminary thresholds were left in place:

- Number of reads after trim must be <10,000
- N50 for *de novo* assembled reads must be < 1,000
- The highest coverage of *de novo* assembled contigs must be < 10x

8.3.1.1.1.2. Spiked in positive PhiX control

The positive spiked-in control PhiX error rate should be <4.9%.

8.3.1.1.1.3. Negative monthly control

Preliminary thresholds were adequate and weren't changed:

- Genomic DNA concentration in the negative sample according to NanoDrop and Qubit must be 0±0.1ng/ul
- Library concentration for the negative sample according to Qubit must be <0.5ng/µl

- Bioanalyzer must show no peak for the negative sample
- If library concentration for the negative sample is >0.5ng/μl and/or Bioanalyzer shows a peak on the electrophoregram- load sample for sequencing. For pooling take the volume of the negative sample which corresponds to the average volume of other samples. Quality metrics of the sequencing data for negative sample must meet following [preliminary] parameters:
  - Number of reads after trim must be <10,000
  - N50 for *de novo* assembled reads must be < 1,000
  - The highest coverage of *de novo* assembled contigs must be < 10x

8.3.1.1.1.4. Positive monthly control

Positive monthly control *E.coli* ATCC 25922 quality thresholds were adjusted in accordance with collected data:

- Average coverage of the genome must be ≥ 15x.
- Q30 score for *E.coli* ATCC 25922 genome sequence must be ≥75% for at least 86bp of the read length.

Following assays results must be acquired for monthly QC control:

- MLST allelic profile acquired during the run must correspond to known ST73 profile described for this strain.
- 16S rRNA sequence must be identified as *Escherichia coli*
- No antibiotic resistance genes should be found with ResFinder analysis
- Following virulence genes should be found using VirulenceFinder analysis with 100% ID and 100% query length coverage:

Virulence factor	Protein function
mchB	Microcin H47 part of colicin H
mchC	MchC protein
iss	Increased serum survival
mchF	ABC transporter protein MchF
mcmA	Microcin M part of colicin H
iha	Adherence protein
sat	Secreted autotransporter toxin
vat	Vacuolating autotransporter toxin
iroN	Enterobactin siderophore receptor protein

See Appendix 13 for the Virulence Finder example of the result for positive E.coli ATCC 25922 control.

8.3.1.2. Accuracy of base calling against reference sequence

The accuracy of the platform was established by determining the proximity of agreement between base calling made by MiSeq sequencer (measured value) and NCBI reference sequence (the true value). Reference sequences were available for 18 strains in the validation set: 10 *Enterobacteriaceae* bacterial samples, 4 gram-positive cocci bacterial samples, 2 gram-negative non-fermenting bacterial samples, and 2 representatives of miscellaneous bacterial species.

We determined MiSeq Illumina platform accuracy by mapping generated reads to the corresponding reference sequence and identifying Single Nucleotide Polymorphisms (SNPs).Certain variations in the sequences are expected due to the possibility of mutations accumulation in the genomes of reference strains during cultivation. This could result in a number of SNPs differences detected between the reference and Core lab-generated validation sequences. For this reason, when comparing validation sequence results against reference sequence, the within- and between-run triplicate sequences of validation samples were taken into account. Reference sequence and sequences generated during 5 independent library preparations were compared. The SNP which is detected between reference and validation sequences should be considered as a sequencing error only when the SNP is detected in less than all 5 replicates. If a SNP detected between reference and validation sequence is identical in all 5 validation replicates, this SNP is not considered as a sequencing error, and instead is considered as a possible mutation in the reference strain:

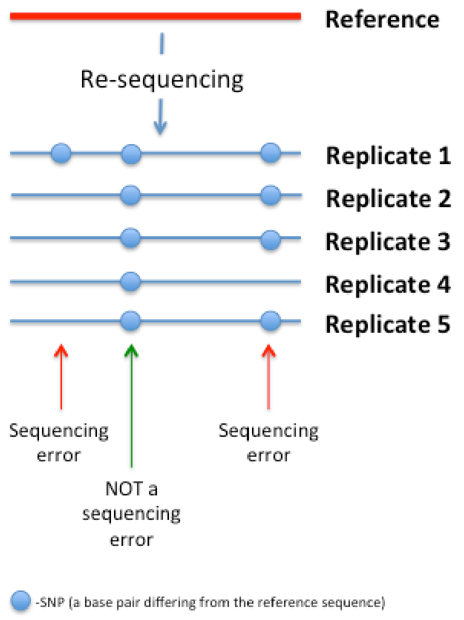

Accuracy here was estimated as percent agreement with reference sequence. For the purposes of platform accuracy measurement, each nucleotide call was considered as an independent test. For this reason, accuracy of base calling was estimated in relation to the number of base pairs in the reference genome sequence.

According to CLIA regulations: Accuracy = # of correct results/total # of results x 100% [20]. For WGS, "# of correct results" equals number of the base pairs sequenced correctly, and "total # of results" corresponds to the total number of base pairs in the reference genome. However, as often a case with resequencing, not the whole genome is covered by the reads and contains numerous gaps where no sequence was generated. Hence, only reference areas covered by the reads should be taken into the account. For Illumina technology, the lack of genome coverage may occur due to: 1) secondary structures or AT abundance in the DNA template, leading to failure to generate the library fragments representing "difficult" genome regions; 2) multiple repeat sequences which fail to assemble correctly; 3) insufficient library representation on the sequencing flow cell. The low coverage of the genome may represent a significant problem for the data analysis and may lead to incorrect results. The first two cases are often difficult to overcome, however they didn't seem to be a problem for any of the species/assay combination which we have used during this validation. The percentage of genome covered was within the range of 86-100% (median 98.4%). It is for each user to establish whether the coverage across the genome which can be achieved in ones hands is sufficient for particular application used in the laboratory. The third issue can be troubleshot for majority of the bacterial species, which are handled in the laboratory, e.g. by increasing the proportion of the sample loading onto the flow cell.

In addition to the absence of the reads coverage due to a failure to sequence certain parts of a genome, the other parts of genome were masked from a between-genome comparison. E.g. mobile elements can skew the hqSNP-based phylogeny and are often excluded from the analysis. Since in some cases masking of mobile elements from genome was performed during presented here validation, it was also taken into account by only calculating length of covered by sequencing portion of the genome, instead of using a number of base pairs in the whole reference genome.

The formula used for the platform accuracy calculation:

$$\% \textbf{ agreement with reference} =$$

$$= \frac{(\textbf{Covered genome length}) - (\textbf{Total \# of SNP difference with the reference})}{\textbf{Covered genome length}} \times \textbf{100\%}$$

See Appendix 5 data interpretation chapter for instructions for retrieving "Total # of SNP difference with the reference" and "# of sequencing errors (SNP is supported only by 4 or less validation replicates)". Retrieve genome size information from NCBI Genome site- see instructions in Appendix 3.

Several validation samples differed from reference genome by several SNPs. However, 99% (324 out of 327) of those SNPs were reproducible among all 5 replicates we have sequenced for each sample. Since sequencing errors are random between different library preparations and it is unlikely that the same erroneous SNP will occur in all 5 replicates, we can conclude that those discrepancies were not caused by sequencing errors, but most likely were a result of accumulation of mutations in the reference strains or previous sequencing mistakes in the reference sequence. This was confirmed by the Sanger sequencing (see Appendix 14).

In both cases, whether we take into the account all SNPs detected between validation and reference sequence, or only those SNPs which don't appear in all of the replicates (true sequencing errors), we observed > 99.999% agreement of generated whole genome sequences with the reference sequences for each tested sample:

Sample	Total # of SNP difference with the reference	# of sequencing errors (SNP is supported only by 4 or less validation replicates)	Genome size (Total # of base pairs in reference sequence), (bp)	Percentage of genome covered (after mobile elements masking)	Covered genome length, bp	% agreement with reference
C1	5	0	5639400	93	5244642	99.99990466
C2	1	0	4744448	100	4744448	99.99997892
C3	22	0	4746220	99	4698758	99.99953179
C4	10	0	5598800	98	5486824	99.99981775
C5	0	0	2806340	99.2	2783889	100
C6	12	0	4964100	99.8	4954172	99.99975778
C46	3	0	2939973	100	2939973	99.99989796
C47	184	2	2499279	96.4	2409305	99.99236294
C48	27	0	2516575	100	2516575	99.99892711
C51	39	0	4989312	100	4989312	99.99921833
C52	2	0	3359001	98.4	3305257	99.99993949
C55	14	1	5203440	98.4	5120185	99.99972657
C72	0	0	5273097	86	4534863	100
C73	0	0	4685848	99	4638990	100
C74	3	0	4710675	97	4569355	99.99993435
C75	0	0	4685848	92	4310980	100
C76	1	0	4685848	93	4357839	99.99997705
C105	4	0	2492821	95	2368180	99.99983109
					Average:	99.9993781

8.3.2. Assay accuracy

Assay accuracy was determined as an agreement of the assay result for the validation samples sequenced by the Core lab with the assay result for reference sequences of the same strains. Four applications of WGS were used to validate the accuracy of the assay: *in silico* Multilocus Sequence Typing (MLST) assay, 16S rRNA gene species identification (ID) assay, assay for detection of antibiotic resistance (ABR) genes, and genotyping assay using high quality Single Nucleotide Polymorphisms (hqSNPs). Sequences generated by the Core lab and corresponding reference sequences were analyzed in the same manner to extract and compare information about sequence type (ST), species ID, present ABR genes, or to generate a phylogenetic tree, correspondingly. One has to make the determination what to consider as a single test for the each specific assay performed in the laboratory. Assay accuracy could be measured only for those validation samples which have reference genomes available from NCBI database or CDC.

The platform accuracy and assay accuracy are interconnected, but it is important to make a distinction between these parts of WGS validation. For the assay accuracy we focus only on areas of the genome targeted by the assay (16S rRNA gene in case of species identification, or several housekeeping genes in case of MLST), while for the platform accuracy its ability to generate a correct base call across the genome is evaluated. The high quality SNP genotyping across the genome can be used as a main assay to validate the platform accuracy, since it allows to validate the accuracy of base calling throughout the genome. Even though, in all of the WGS-based assays we ultimately evaluate the accuracy of a single nucleotide base call made by the platform which directly affects the results of the above-mentioned assays, WGS assays may tolerate a certain error rate of the platform and still can yield accurate results, as long as the assay was validated with a given platform. This is especially true in cases when it is possible to reach a decent depth of the genome coverage in a particular area of the genome targeted by the assay. Certain base calling errors of the platform can also be removed using bioinformatics (56-58). From previously published studies [23,28,29], the SNP analysis or assays like whole-genome-MLST should be done with at least at 30x depth of genome coverage. We determined optimal depth of coverage to be ≥ 60x based on accuracy of SNP detection at various simulated genome coverages. However 15x coverage threshold was sufficient for other WGS assays (MLST, 16S ID, ABR genes detection) and 15x was determined as the minimum acceptance criteria for raw data in order to be considered for the mentioned types of analysis. If presence/absence of certain genes is a key diagnostic feature, the corresponding WGS assay should be added to the validation panel. Validation of the specific assays in turn allows to determine the threshold for the base calling accuracy of the platform which is required to generate accurate and reproducible assay results.

8.3.2.1. Accuracy of *in silico* MLST assay

Illumina-generated whole genome sequencing data was shown previously to be of sufficient quality to allow for high levels of allele identification from *in silico* MLST typing analysis. In a published study, among 80 strains with known MLST sequence-types concordance was 98.4% (551 out of 560 alleles) [30].

MLST is the method of bacterial genotyping which involves sequencing of 6-to-7 housekeeping genes throughout the bacterial genome. Sequence variation (or alleles) of those target genes are used to establish genetic relatedness of the isolates. Combination of known alleles (e.g. *aroC-dnaN-hemD-hisD-purE-sucA-*

thrA=116-7-12-9-5-9-2) of target genes allows assignment of sequence type number, e.g. ST328. There are two options for the single test definition in the case of MLST: 1) consider the final sequence type result as a single test result; or 2) evaluate the result of the each allele identification separately and consider each allele call as a separate test. We believe, that in complex assays, it is reasonable to consider the detection of each of the multiple genetic determinants as a separate test, especially when sequence variation of each of the determinants changes the end results of the assay. To illustrate it with the example of MLST, any sequence variation in the MLST alleles will lead to the change of the allele identification number and will results in a new allele profile, which will lead to sequence type change, therefore each allele identification was counted as a separate test. The definition of the correct result for MLST corresponds to a correct identification of each of the MLST alleles in the validation sequence.

Fifteen validation sequences and their corresponding reference sequences for the same strains were analyzed by *in silico* MLST. Only those isolates with matching reference sequences online and available MLST typing scheme were analyzes. MLST profiles were identified by the CGE tool included in Core lab analysis pipeline. The protocol for *in silico* MLST analysis and results for all samples is in the Appendix 6.

**Sample**	**Reference ST**	**Test sample ST**	**# of allele total/ # of allele different**	**Accuracy**
C1	ST-11	ST-11	7/0	(Number of correct allele results/Total number of alleles) x 100 = (104/104)*100= **100%**
C2	ST-1	ST-1	6/0	
C3	ST-3021	ST-3021	7/0	
C4	ST-1	ST-1	7/0	
C5	ST-243	ST-243	7/0	
C6	ST-19	ST-19	7/0	
C46	ST-30	ST-30	7/0	
C47	ST-8	ST-8	7/0	
C51	ST-14	ST-14	7/0	
C55	ST-73	ST-73	7/0	
C73	ST-11	ST-11	7/0	
C74	ST-32	ST-32	7/0	
C75	ST-440	ST-440	7/0	
C76	ST-592	ST-592	7/0	
C72	ST-655	ST-655	7/0	
		Total:	104/0	

Detection and correct identification of each of the MLST alleles in the typing scheme represents an independent test. Accuracy is represented by percent of allele agreement between all alleles detected in validation samples compared to reference sequences. For all validation samples each of the sequences of the 7 housekeeping genes used in the typing scheme (or 6 genes- for *Aeromonas hydrophilia*) were identified correctly, resulting in 100% allele identification accuracy.

All results for validation samples matched the reference results, no confirmatory testing is required.

8.3.2.2. Accuracy of 16S rRNA gene identification assay

For 16S rRNA ID assay variations only in one gene were detected, so the species ID results as a whole (e.g. "*Escherichia coli*") was considered as a single test. The correct test result, is when the identity of 16S rRNA sequence extracted from validation sample is matching the identity of 16S rRNA sequence extracted from the reference sequence.

16S rRNA sequences were extracted from annotated NCBI or CDC reference sequences and from annotated sequences of 18 test samples and compared against RDP database. See protocol for 16S rRNA gene identification and results for all samples in Appendix 6.

For all validation samples results of 16S rRNA gene ID assay matched reference results:

Sample	Reference 16S ID	Test sample 16S ID	Accuracy
C1	*Escherichia coli*	*Escherichia coli*	(Number of correct ID tests/Total number of tests) x 100 = (18/18)*100= **100%**
C2	*Aeromonas hydrophila*	*Aeromonas hydrophila*	
C3	*Escherichia coli*	*Escherichia coli*	
C4	*Enterobacter cloacae*	*Enterobacter cloacae*	
C5	*Staphylococcus aureus*	*Staphylococcus aureus*	
C6	*Salmonella enterica*	*Salmonella enterica*	
C46	*Enterococcus faecalis*	*Enterococcus faecalis*	
C47	*Staphylococcus epidermidis*	*Staphylococcus epidermidis*	
C48	*Staphylococcus saprophyticus*	*Staphylococcus saprophyticus*	
C51	*Stenotrophomonas maltophilia*	*Stenotrophomonas maltophilia*	
C52	*Legionella pneumophila*	*Legionella pneumophila*	
C55	*Escherichia coli*	*Escherichia coli*	
C72	*Escherichia coli*	*Escherichia coli*	
C73	*Salmonella enterica*	*Salmonella enterica*	
C74	*Salmonella enterica*	*Salmonella enterica*	
C75	*Salmonella enterica*	*Salmonella enterica*	
C76	*Salmonella enterica*	*Salmonella enterica*	
C105	*Corynebacterium jeikeium*	*Corynebacterium jeikeium*	

All results for validation samples matched the reference results, no confirmatory testing is required.

8.3.2.3. Accuracy of Genotyping assay

Accuracy of the genotyping assay is the ability of the assay to correctly determine genetic relatedness of the isolates. Validated here, high-quality SNP genotyping is based on mapping of the validation sequencing reads to a reference genome, which is followed by genome-wide SNP calling against the reference; the identified SNPs are used to build a phylogenetic tree and this way to determine the genetic relatedness of tested isolates. Topology of the phylogenetic tree reflects genetic distances between isolates: shorter branches equal closer related isolates, longer branches equal less related isolates. In other words, genetically related isolates cluster closer together than unrelated isolates. To assess accuracy of genotyping assay, phylogenetic trees were built using reference sequences and validation sequences, and resulting trees were compared. Trees were created for 25 samples belonging to 2 different species of *Enterobacteriaceae* family, 2 species of Gram-positive bacteria, and one species on Non-fermenting bacteria. See detail of sampling and data analysis in Appendix 1.

For better comparison we suggest using at least 5 strains of the same species to build a tree. If the set of microorganisms used for validation doesn't have 5 representative strains of the same species, it is possible to use genomes available from NCBI to include into the analysis in addition to either reference or validation sequences. E.g., if the validation set contains 3 strains of *E. coli*, one can download 2 more *E.coli* genomes from NCBI and use those sequences to build two trees: a) reference tree, containing sequenced of the 3 reference strains from NCBI and 2 additional NCBI genomes for comparison; and b) validation tree, built with 3 genomic sequences generated for the reference strains during validation and the same 2 additional NCBI genomes for comparison. See an example in the figure below:

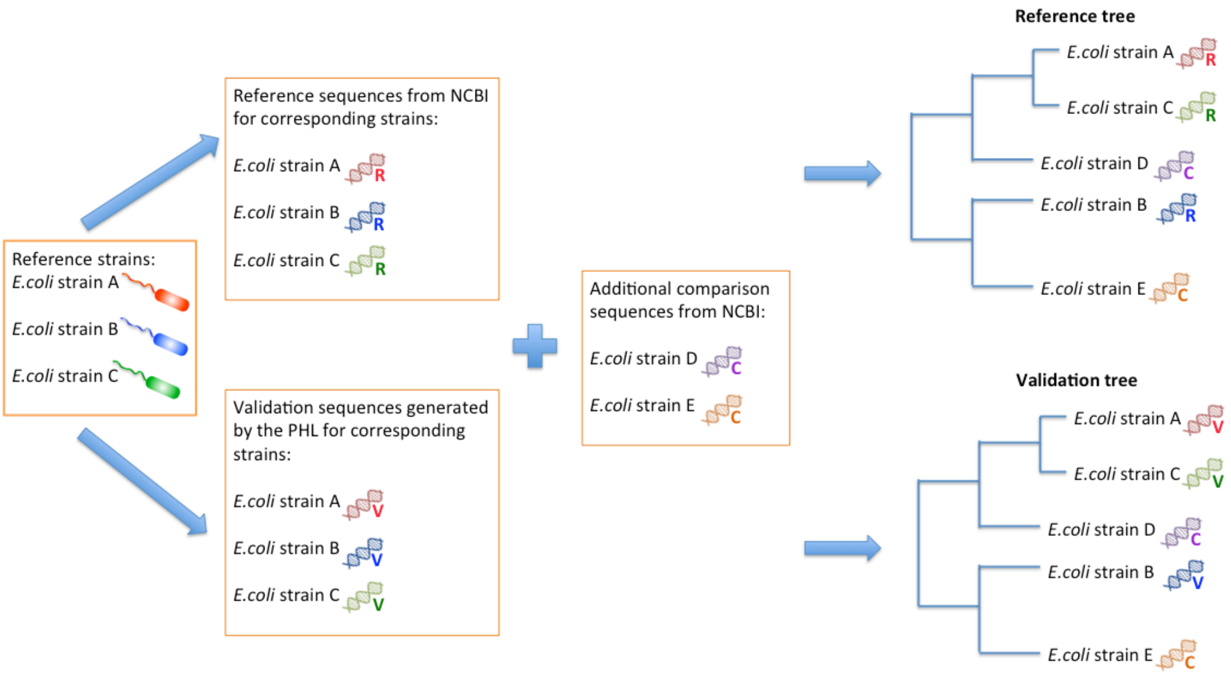

The accuracy of genotyping test was determined using two approaches:

1) Topological similarity between reference tree and validation tree. Tree agreement was statistically measured using Compare2Trees software, which provided a percentage of topological similarity between two trees.

2) Comparison of clustering pattern of validation tree and reference tree. The definition of the correct result used for genotyping assay is following: Clustering pattern upon the phylogenetic comparison of samples sequenced by Core lab must match the pattern generated from the clustering of reference sequences. In other words, conclusions made about the relatedness of the isolates drawn from validation tree and reference tree should be the same.

During this validation, the phylogenetic trees were generated for 5 microorganisms. All 5 validation trees had matching clustering patterns and 100% of topological similarity with corresponding reference trees. Concluded accuracy of genotyping test was 100%

**Species**	**Sample**	**Reference sequences for isolates sequenced by Core lab**	**Additional references used for comparison**	**Clustering of reference tree was replicated for all validation samples (Y/N)**	**% Tree similarity**	**% agreement** (average tree similarity for different species)
Escherichia coli	C1, C3, C55	NZ_CP008957.1, NC_010468.1, NZ_CP009072.1	NC_000913.3, NC_002695.1	Y	100%	100%
Salmonella enterica	C73, C74, C75, C76, C77	SRR518749, SRR1616809, SRR1686419, SRR1614868, SRR1640105	none	Y	100%	
Staphylococcus aureus	C5	NZ_CP009361	NC_007622, NC_007795, NC_009782, NC_017333	Y	100%	
Enterococcus faecalis	C46	NZ_CP008816	NC_018221, NC_019770, NZ_CP004081, NC_004668	Y	100%	
Stenotrophomonas maltophilia	C51	NZ_CP008838	NC_011071, NC_015947, NC_017671, NC_010943	Y	100%	

Interpretation of the phylogenetic analysis results for different species may vary due to the degree of bacterial population clonality or different mutation rate. This may raise a question in necessity of validation of phylogenetic analysis for each species, or maybe even serotype, individually. This question will need further investigation, but in our opinion, as long as the SNP calling and phylogenetic tree building algorithms are the same for the array of routinely tested species, it should be sufficient to test the phylogenetic pipeline for

representative species only. If different phylogenetic analysis approach is required for certain species which laboratory routinely works with, it has to be validated separately.

8.3.2.4. Accuracy of antibiotic resistance genes detection assay

To calculate an accuracy of the ABR genes detection assay, we detected presence/absence of antibiotic resistance genes using ResFinder database. Two sets of sequences were analyzed: 1) The ATCC reference bacterial strains designated for use as antibiotic susceptibility controls were sequenced by the laboratory performing the validation. 2) Reference sequences were acquired from the FDA-CDC Antimicrobial Resistance Isolate Bank for *in silico* testing.

8.3.2.4.1. Detection of resistance genes in the ATCC strains using ResFinder

Five ATCC reference strains (designated for use as antibiotic susceptibility controls) were sequenced and generated sequences were used for the analysis with ResFinder. Negative controls were chosen among strains which were described by the CLSI M100-S24 document as susceptible, with no known antibiotic resistance genes. Positive controls were chosen among strains, which according to the CLSI M100-S24 resistance determinants. The results of ResFinder detection were compared to the resistance genes known to be present in the ATCC strains. See details of the analysis and results reports for each sample in the Appendix 9.

The comparison of the tested sequences against each entry in the database of ABR genes was considered as an independent test. ResFinder database at the moment of validation contained sequences of 1719 antibiotic resistance genes, against which each of the validation samples was compared, resulting in a total of 1719 tests performed for each validation sample. The correct results for ABR genes detection assay would be defined as follows: In negative control samples all 1719 tests must give negative results. In chosen here positive controls 1 out of 1719 tests must give a positive result and the rest must remain negative. All of those criteria were met for all validation samples, therefore the accuracy of the assay for ABR genes detection was 100%.

The table below details the results of antibiotic resistance genes detection by ResFinder in validation samples:

	Sample ID	Strain	CLSI M100-S24 description	Results	Number of genetic determinants detected correctly	Accuracy
positive controls	C50	*Pseudomonas aeruginosa* ATCC 27853	contains inducible AmpC β-lactamase	positive (blaOXA-50 β-lactamase detected)	1719	(Number of correct ID tests/Total number of tests) x 100 = (8595/8595)*100= **100%**
	C103	*Bacteroides fragilis* ATCC 25285	β-lactamase positive	positive (cepA β-lactamase detected)	1719	
negative controls	C5	*Staphylococcus aureus* ATCC 25923	susceptible control	negative (no antibiotic resistance genes detected)	1719	
	C55	*Escherichia coli* ATCC 25922	susceptible control	negative (no antibiotic resistance genes detected)	1719	
	C46	*Enterococcus faecalis* 29212	susceptible control	negative (no antibiotic resistance genes detected)	1719	

Footnote: Total number of tests in this case corresponds to the number of database entries multiplied by the number of samples, because a comparison of sequencing data of each sample was performed against each entry in the database.

All results for validation samples matched the reference results, no confirmatory testing is required.

8.3.2.4.2. *In silico* detection of resistance genes in sequences from the FDA-CDC AR Isolate Bank using ResFinder

Reference sequences were acquired from the FDA-CDC Antimicrobial Resistance Isolate Bank (https://www.cdc.gov/drugresistance/resistance-bank/) for in silico testing. The fastq files of the corresponding isolates were downloaded from the NCBI Short Read Archive (SRA) and subjected to quality trimming, *de novo* assembly, and ResFinder analysis following standard procedure used earlier. Thirteen isolates (seven Gram-negative and six Gram-positive) with various resistance genes were analyzed (see results of the comparison for each sample in the Appendix 15).

The analyzed isolates harbored a total of 83 resistance genes (representative of 57 different alleles) which were detected by the CDC using both PCR-based methods (for the main resistance types) and by ResFinder.

Two types of comparison were performed:

1) Comparison of the results of PCR-based methods performed at the CDC (available for 8 isolates) with the ResFinder results acquired during the validation.

Our analysis of reference sequences with ResFinder confirmed presence of all genes which were detected by PCR-based methods (n=8) resulting in 100% accuracy:

Total genes detected by PCR-based method=8
Number of genes detected by ResFinder which are matching PCR-based results =8

Accuracy = (8/8) x 100% = 100%

ResFinder does not have ability to detect the truncation of porin genes; therefore porin-related resistance mechanisms mentioned in the CDC database could not be detected with ResFinder.

2) Comparison of the results of ResFinder analysis performed at the CDC (database last updated 06/02/16) with the ResFinder analysis performed during the validation (database last updated 02/17/17).

Total number of the resistance genes in the ResFinder database at the moment of data analysis= 2112
Number of samples = 13
Total number of the tests performed= 2112 x 13 = 27456

# of False positive results = 6
# of False Negative results= 2
Total # of tests in agreement = 27448

Agreement= (# Total tests in agreement/# Total tests) x 100% = (27448/27456) x 100% = 99.971%

The agreement between CDC ResFinder results and validated ResFinder results was 99.97%.

In case of Gram-negative bacteria, all discrepancies were caused by the additional genes detected during validation in comparison with CDC ResFinder results ("false-positive" results). Upon further investigation, several ResFinder database updates were carried out between the update dates. This explains the discrepancies.

In case of Gram-positive bacteria, several discrepancies were also caused by additional genes detected by us, but 2 genes were missing from our results while present in CDC results ("false-negative" results). Both false negative genes were detected by us but possessed <99% ID or incomplete sequence and therefore where excluded from the final result.

8.3.3. Accuracy of bioinformatics pipeline

As suggested previously, in addition to the data generated by in-house sequencing, the simulated data for optimization of variant and genotype calling was used [31]. Accuracy of the bioinformatics pipeline used for hqSNP genotyping was assessed by performing phylogenetic analysis on raw WGS reads of bacterial isolates from a well-characterized outbreaks and comparing validation results to previously published phylogenetic results. Two studies, presenting the phylogenetic analysis of outbreaks, caused by a gram-positive pathogen in one study [12] and a gram-negative pathogen in another study [8] (at least 6 isolates/study), were used for validation of the bioinformatics pipeline. Raw sequences of representative outbreak isolates were available from public databases. Isolates from both studies had human source. The relatedness of isolates to the outbreak was established in previous studies by whole genome sequencing and confirmed by epidemiological data. Epidemiologically unrelated isolate presented in the study were also analyzed for comparison.

**Study**	**Study 1.** SR Harris et al. Lancet Infect Dis 2013; 13: 130–36 [PMID: 23158674] [12]	**Study 2.** P Leekitcharoenphon et al. PLoS ONE 2014;9(2):e87991 [PMID: 24505344] [8]
**Microorganism**	Methicillin-resistant *Staphylococcus aureus*	*Salmonella enterica* serovar Typhimurium
**Source of isolates**	Human	Human
**Number of isolates analyzed**	7 outbreak isolates (1 outbreak cluster) + 2 epidemiologically unrelated isolates	9 outbreak isolates (4 outbreak clusters) + 2 epidemiologically unrelated isolates
**Type of outbreak**	Hospital-associated outbreak	Foodborne outbreaks
**ID of the samples in the study which were used for validation**	P1, P2, P3, P4, P16, P21, P25, Identified by Infectious Control Investigation non-outbreak ST1, MRSA identified by searching microbiology database non-outbreak ST772	0803T57157, 0808S61603, 0808F31478, 0903R11327, 0811R10987, 0804R9234, 0810R10649, 0901M16079, 0110T17035, 1005R12913, 1006R12965
**Accession ## of corresponding samples**	ERR070045, ERR070042, ERR070043, ERR070044, ERR124429, ERR124433, ERR128708, ERR070041, ERR072248	ERR277220, ERR277226, ERR277223, ERR277222, ERR277224, ERR277221, ERR277227, ERR277228, ERR277203, ERR277233, ERR277234
**# of clusters in study tree**	1	4
**# of clusters in validation tree**	1	4
**# of outbreak isolates in each cluster in the study tree**	Cluster 1= 7	Cluster 1= 2, Cluster 2= 3, Cluster 3= 2, Cluster 4= 2
**# of outbreak isolates in each cluster in validation tree**	Cluster 1= 7	Cluster 1= 2, Cluster 2= 3, Cluster 3= 2, Cluster 4= 2
**# of epidemiologically unrelated isolates in the set**	2	2
**# of epidemiologically unrelated isolates clustered with outbreak isolates**	0	0
**% agreement= (# of outbreak isolates clustered correctly in validation tree)x100%/ (Total # of outbreak isolates clustered together in the study tree)**	(7x100/7) = 100%	(9x100/9) = 100%

Bioinformatics pipeline accuracy criteria: Clustering suggested by previous investigators must match clustering achieved by the analysis using Core lab validation bioinformatics pipeline.

<u>Clustering reproduced for Study 1</u>

Below is a figure of the phylogenetic tree of outbreak isolates, which was published in the study 1 by Harris et al. [PMID: 23158674] ("Study 1 tree"). The isolates from the study which were picked for validation have arrows pointing at them and numbers 1 through 7 assigned for purposes of validation. Tree to the right from the Study 1 tree is a copy of phylogenetic connections between chosen isolates from original study tree. Phylogenetic tree generated using the Core lab bioinformatics pipeline is called "Validation tree 1" and presented below. The same isolates in the original tree and in the validation tree are marked with the same numbers.

Study 1 tree (Harris et al., PMID: 23158674):

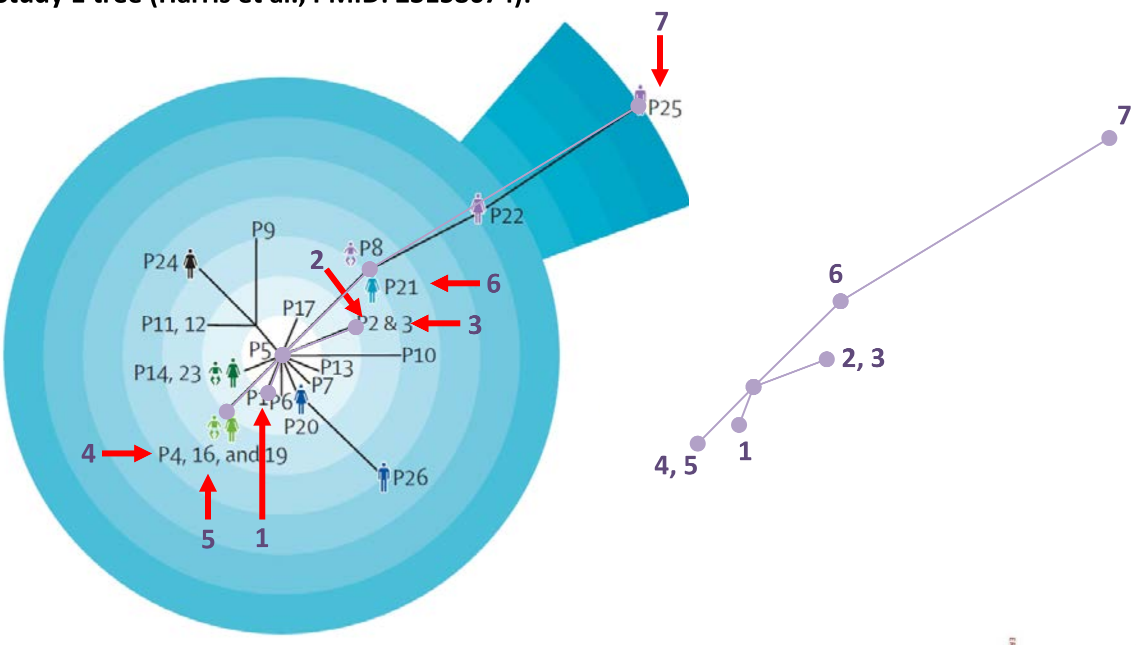

Validation tree 1:

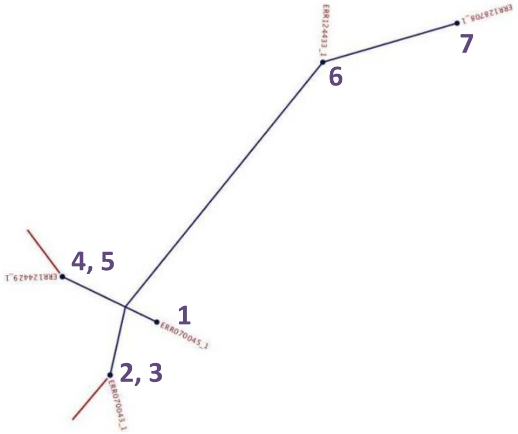

The topology of the trees may differ; however, the clustering of validation tree completely replicates clustering of Study 1 tree. E.g. isolates 4 and 5 were identical and clustered together according to the Study 1, and the same results were shown in validation tree, with isolates 4 and 5 sharing the same node. All conclusions in

regards to the genetic relatedness of the isolates that can be drawn from Study 1 tree can also be made from analysis of validation tree 1.

Group of related isolates from Study 1 was compared with epidemiologically unrelated isolates suggested by the same study (no tree available from publication by Harris et al.). Phylogenetic analysis using the Core Lab bioinformatics pipeline showed that epidemiologically unrelated isolates did not cluster with the group of outbreak isolates and appeared to be genetically distant. Thus, the resulting phylogenetic tree produced by Core lab bioinformatics pipeline showed complete concordance with the epidemiological data.

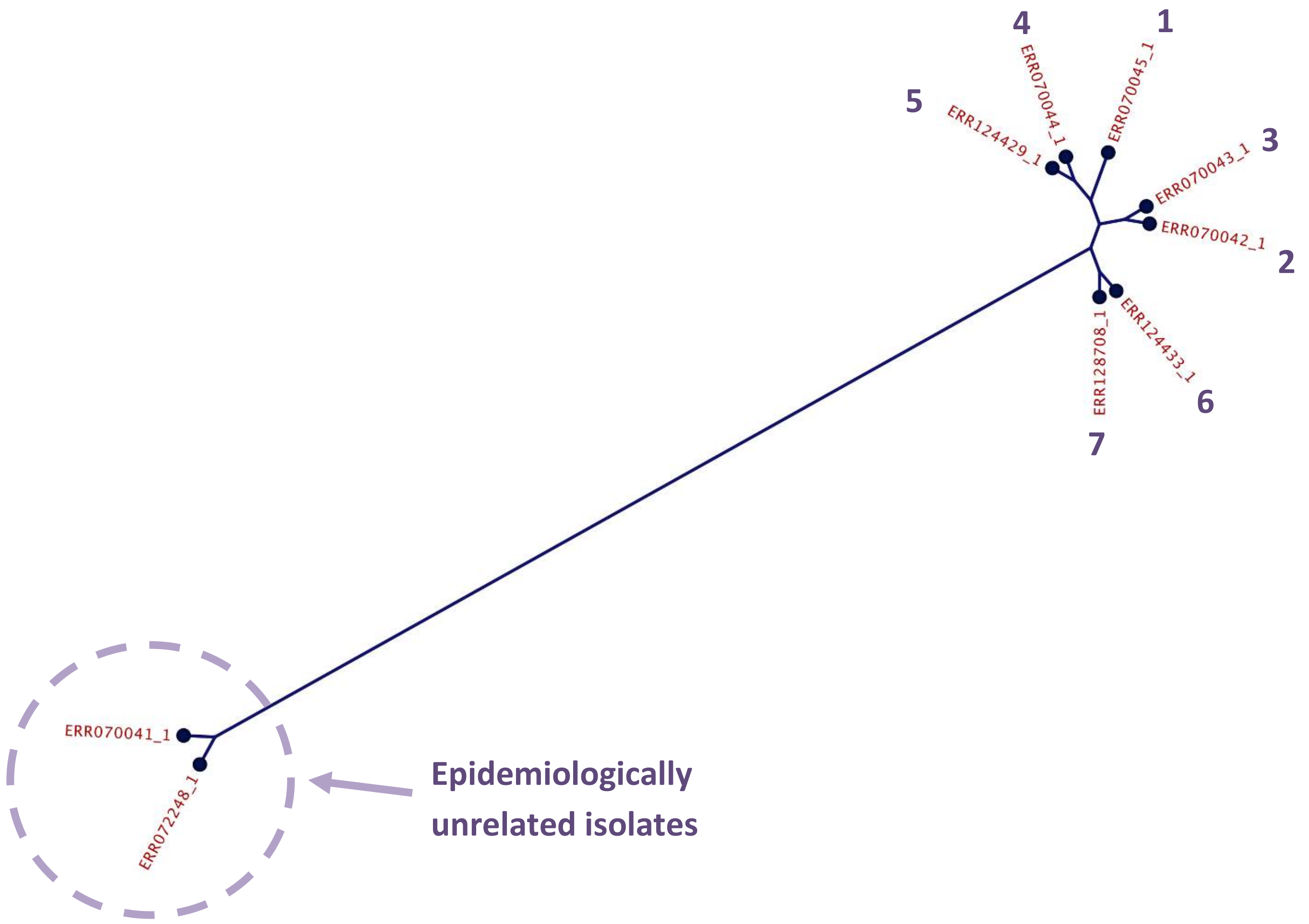

Clustering reproduced for Study 2

Phylogenetic tree combining epidemiologically related and nonrelated isolates published in the study 2 by Leekitcharoenphon et al. [PMID: 24505344] is represented below as “Study 2 tree”:

Study 2 tree (Leekitcharoenphon et al., PMID: 24505344):

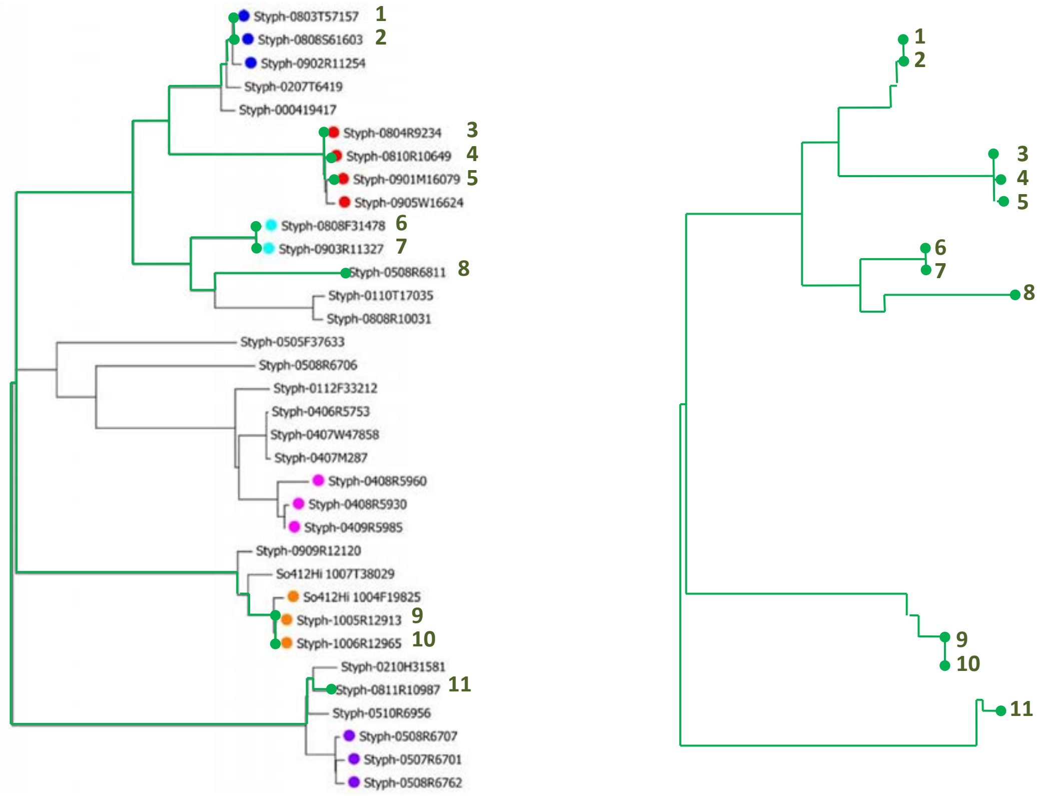

Validation tree 2:

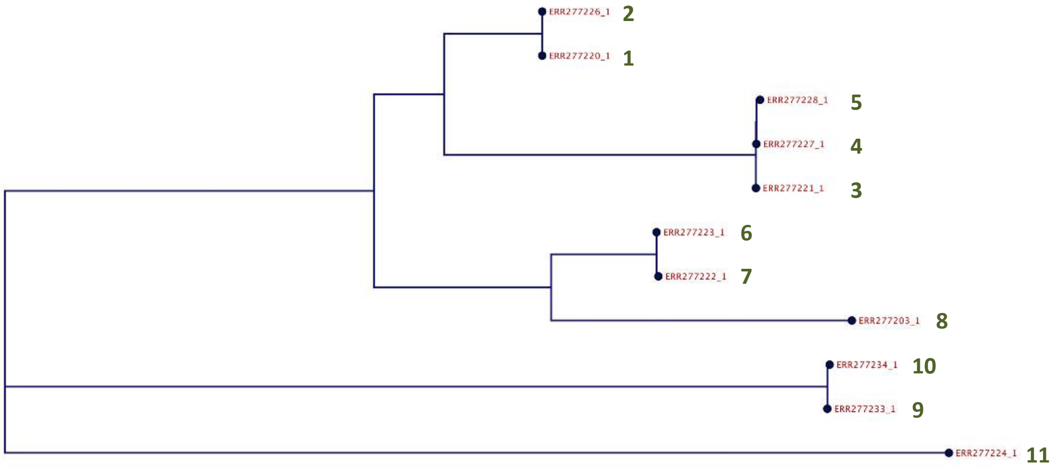

The isolates from the study 2 which were picked for validation marked with green node circles and had numbers 1 through 11 assigned for purposes of validation. Tree to the right from the Study 2 tree is a copy of phylogenetic connections between chosen isolates from original study tree from publication by Leekitcharoenphon et al. Nine of the selected for validation isolates are representative of 4 independent outbreaks and two isolates are epidemiologically unrelated controls:

Outbreak A: isolates 1, 2
Outbreak B: isolates 3, 4, 5
Outbreak C: isolates 6, 7
Outbreak D: isolates 9, 10
Isolate 8- epidemiologically unrelated
Isolate 11- epidemiologically unrelated

The phylogenetic tree generated using the Core lab bioinformatics pipeline is called “Validation tree 2”. The same isolates in the tree from Study 2 and in the validation tree are marked with the same numbers.

The clustering of validation tree is identical to the clustering of Study 2 tree. For example, isolates 6 and 7 were a part of the same outbreak, while isolate 8 is an epidemiologically unrelated control used in the study. In accordance with epidemiological data and Study 2 tree, the validation tree showed that isolates 6 and 7 do cluster together, but not with isolate 8 . All conclusions in regards to the genetic relatedness of the isolates which can be drawn from Study 2 tree by Leekitcharoenphon et al. can be also made from analysis of validation tree 2.

In summary, based on analysis of simulated data from both studies accuracy of the pipeline for phylogenetic analysis was 100%.

8.4. Inter- and Intra-assay Agreement

Inter- and intra-assay precision is also referred to as within and between- run precision, or repeatability and reproducibility, correspondingly.

Repeatability (precision within run) is established by sequencing the same samples multiple times under the same conditions and evaluating the concordance of the assay results and performance.

Reproducibility (precision between runs) is assessed as the consistency of the assay results and performance characteristics for the same sample sequenced under different conditions, such as between different runs and different sample preparations.

Thirty-four validation samples (as above) each were sequenced 3 times in the same run (for repeatability) and in 3 times in different runs (for reproducibility). For between run reproducibility assessment, all replicates were processed on different days, altering two operators, and generated starting from fresh culture (exception: replicates for *Mycobacterium tuberculosis* samples were generated starting from DNA), as suggested by the CLSI MM11A document [32]. For within run replicates one DNA extract was used, but independent library preparations were done, with final samples being included in one sequencing run. For each sample: Number of intra-assay replicates = 3, Number of inter-assay replicates = 3; Total number of repeated results= 5, as explained in the figure below:

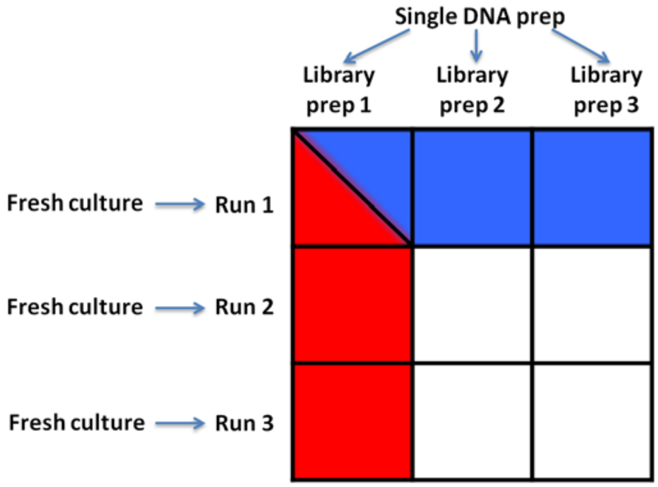

Red- between-run replicates, blue- within-run replicates.

Repeatability and reproducibility were assessed for 3 assays (Genotyping, MLST, and 16S rRNA gene ID). The precision in quality metrics among all replicates was evaluated as well. Concordance of performance was evaluated based on the following quality control metrics: depth of coverage, uniformity of coverage, and accuracy of base calling (Q score). All quality parameters remained relatively constant within and between runs. See all quality metrics of sequences generated for all replicates in the Appendix 8. Below see the ranges established for the corresponding data metrics for all replicates. For each metrics two graphs were plotted:

a) a distribution of samples by their value of given parameter;

b) a comparison of median value of the corresponding quality metric for all validation samples analyzed originally (see results in Chapter 8.3.1.1) vs. median value of quality metric for all replicates ("Original" vs. "Replicates"). Error bars reflect standard deviation.

Average read length with ≥ Q30 (= avg read length after trimming) = 115-191.1bp (median 142bp)

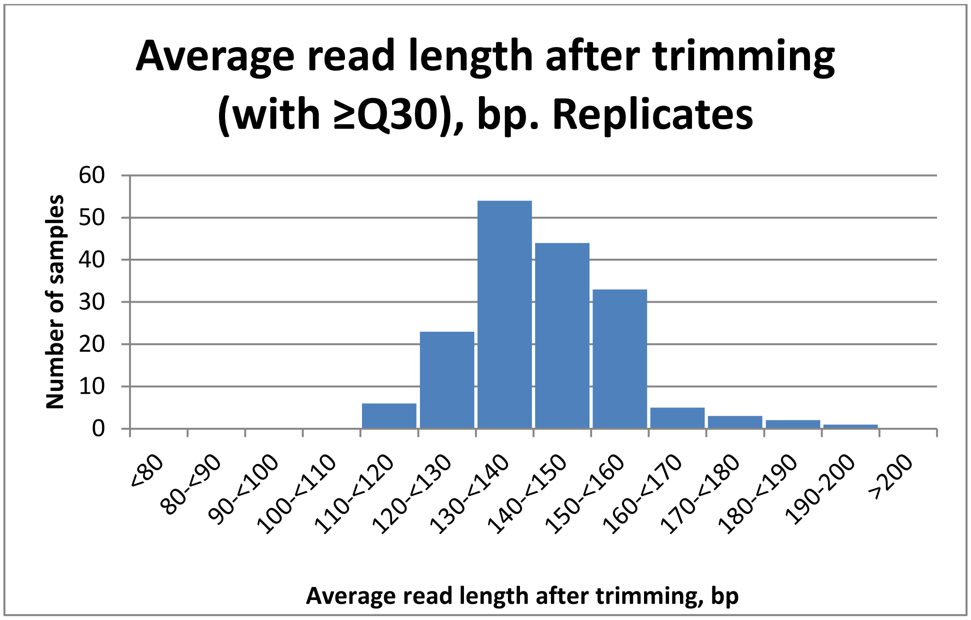

Comparison of parameter's median value for replicates with original median value:

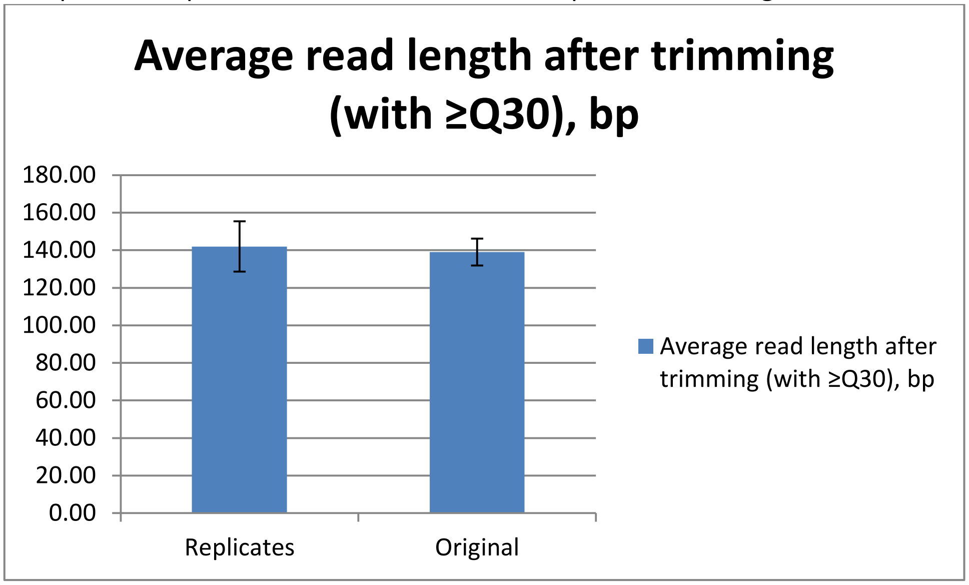

Read length at which 75% of bases have quality score ≥Q30 = 90-225bp (median 160bp)

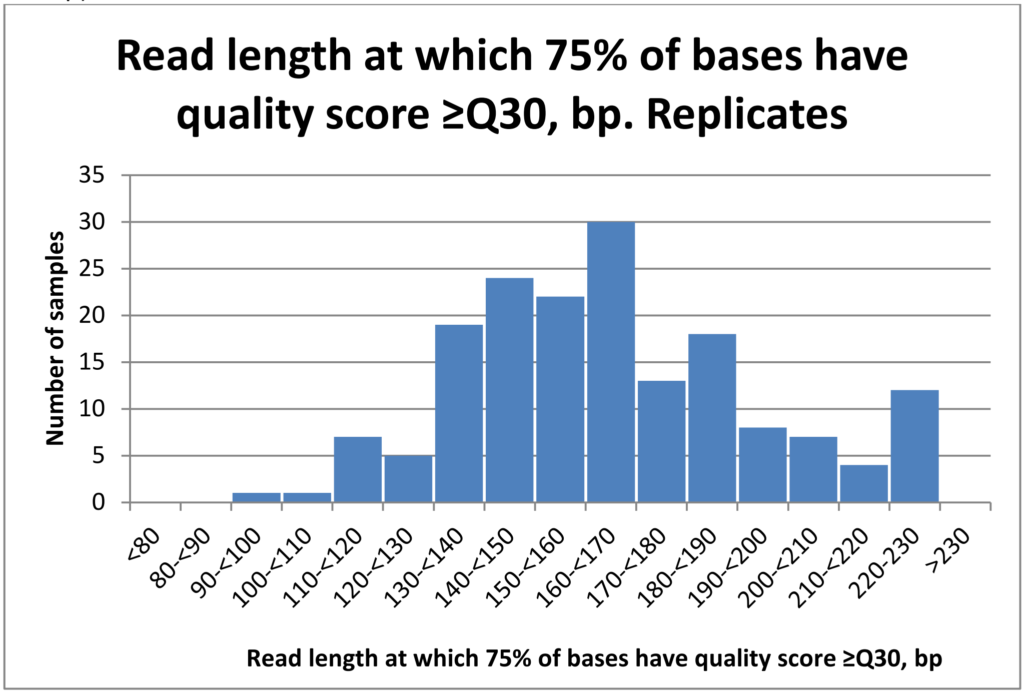

Comparison of parameter's median value for replicates with original median value:

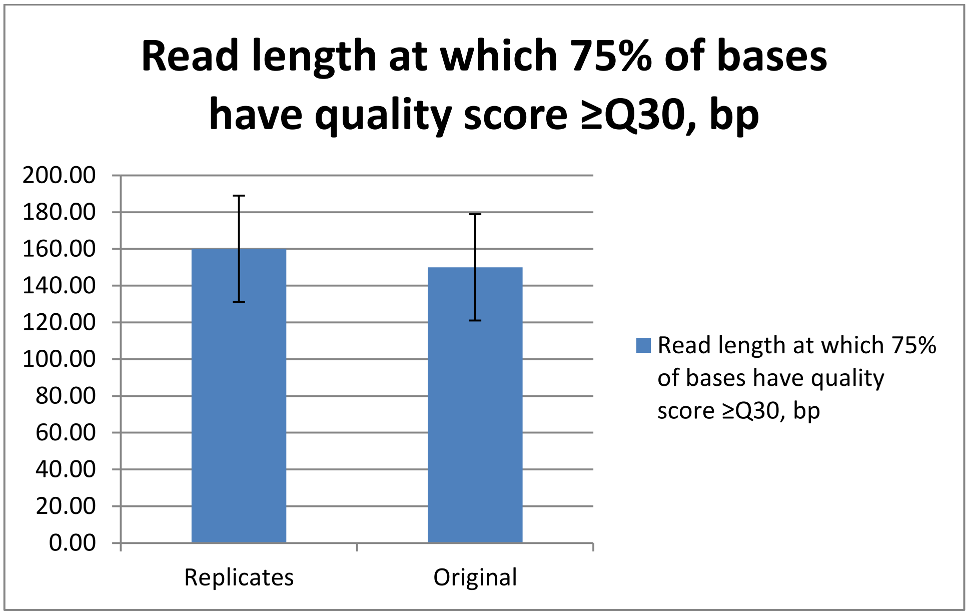

Average depth of coverage of genome= 15.71-216.4x (median 71.55x)

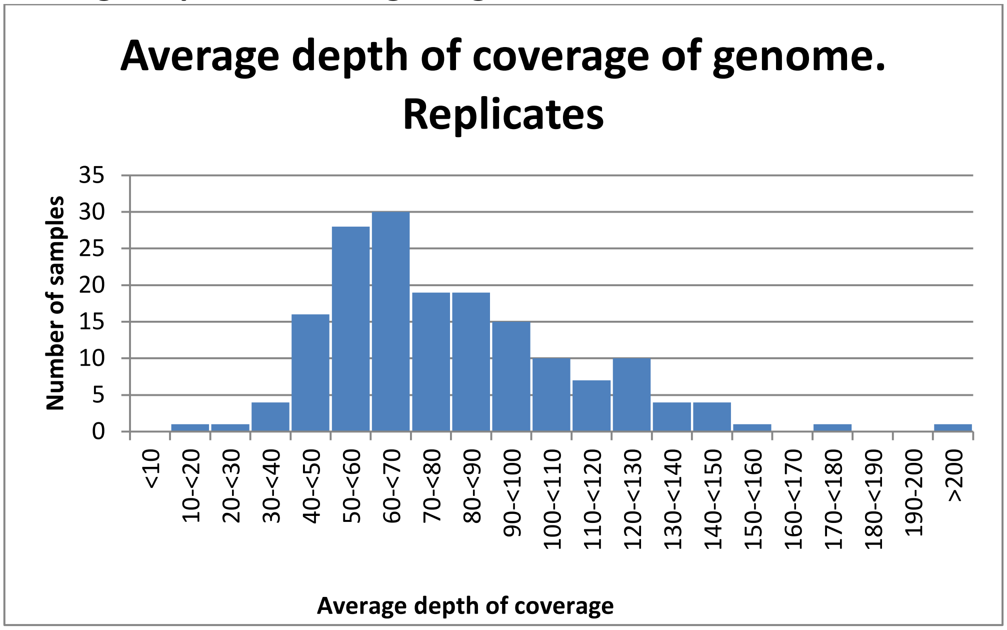

Comparison of parameter's median value for replicates with original median value:

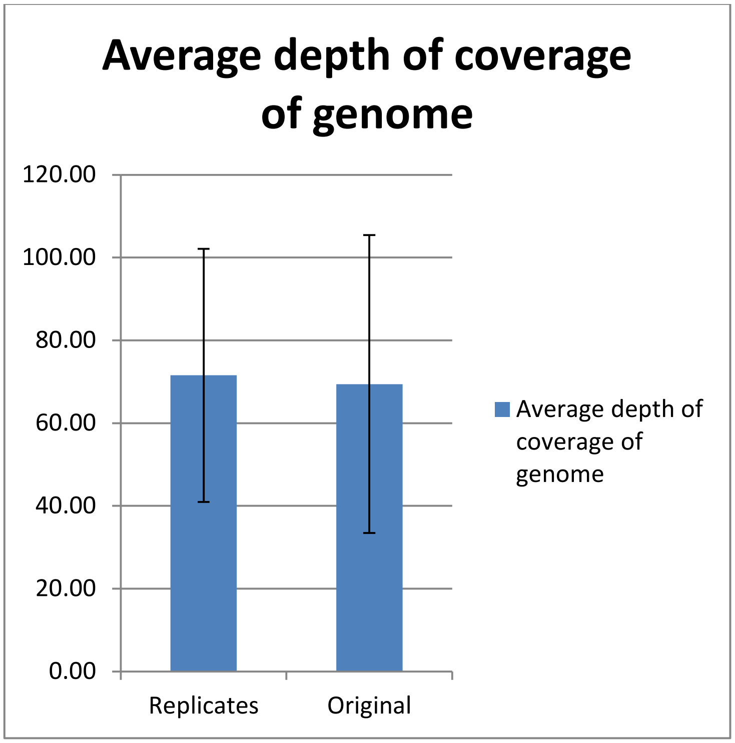

Percentage of genome covered (after mobile elements masking)= 85-100% (median 98%)

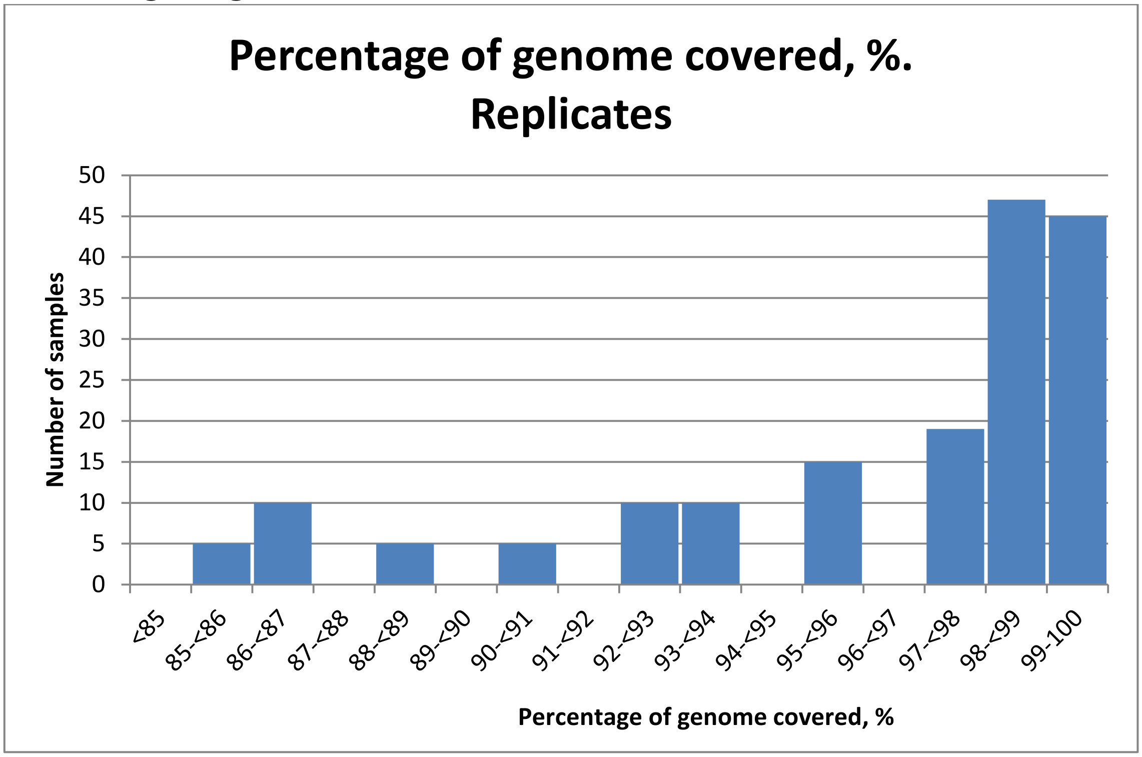

Comparison of parameter's median value for replicates with original median value:

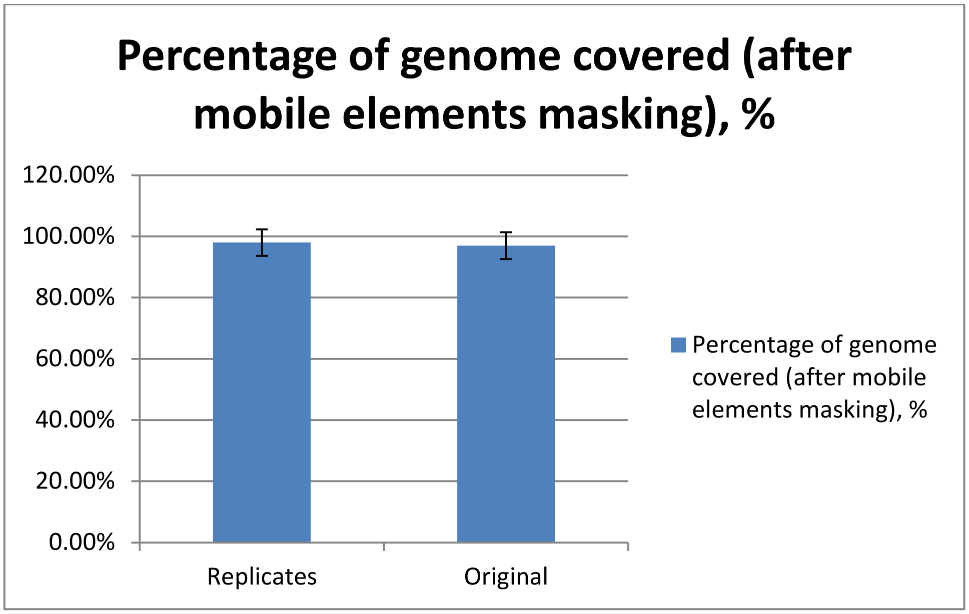

Uniformity of coverage at 10x (Percent of positions with coverage ≥10x)= 50.04-99.93% (median 95.71%)

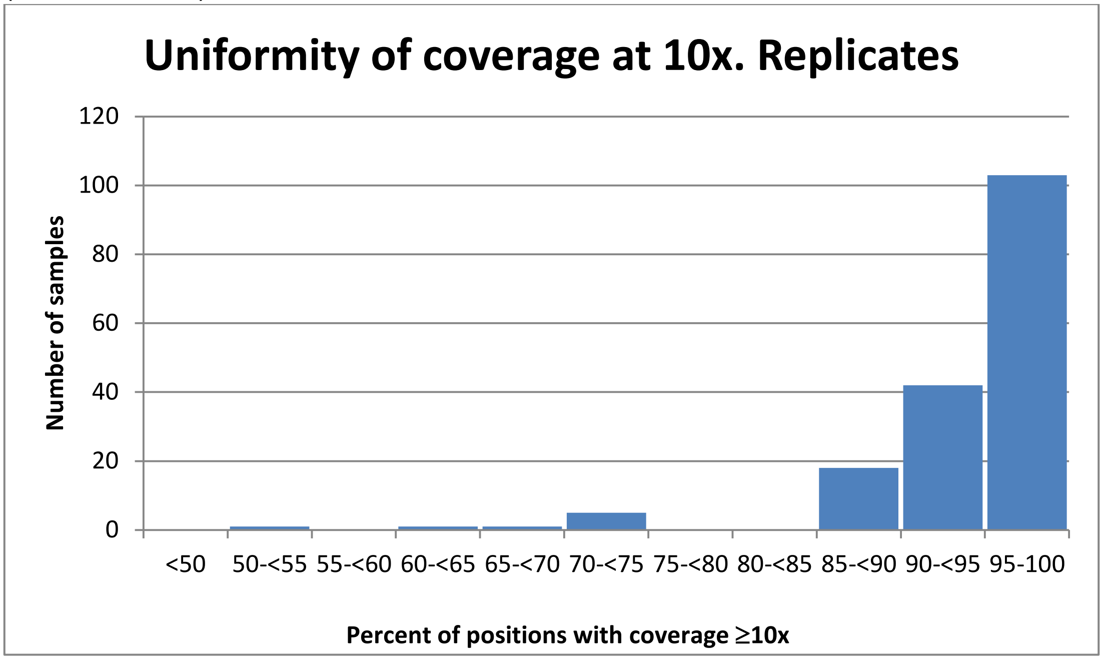

Uniformity of coverage at 5x (Percent of positions with coverage ≥5x)= 74.68-99.98% (median 96.55%)

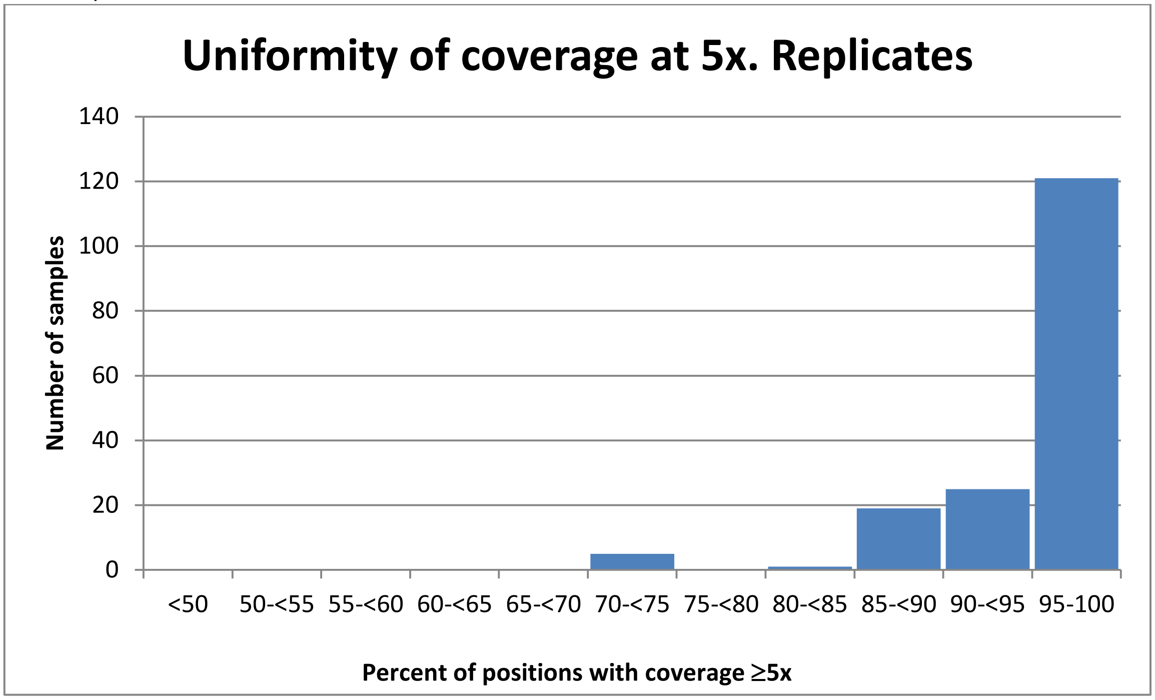

Comparison of parameter's median value for replicates with original median value.

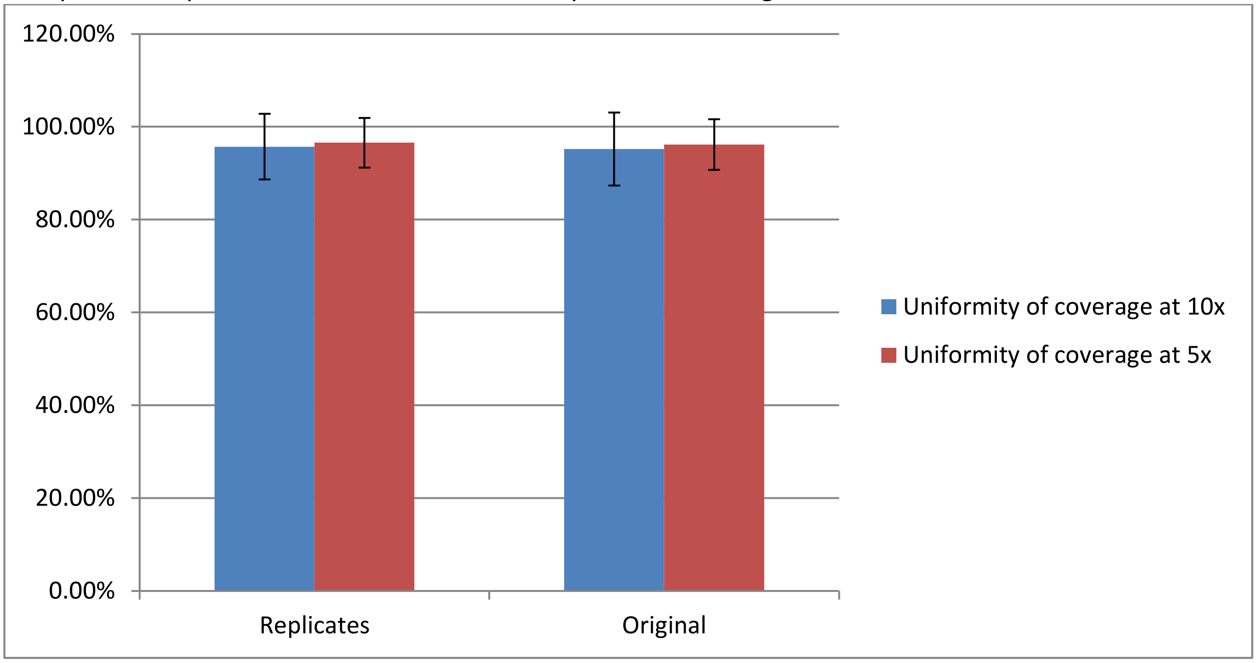

PhiX error rate =2.88-4.74% (median 3.43%)

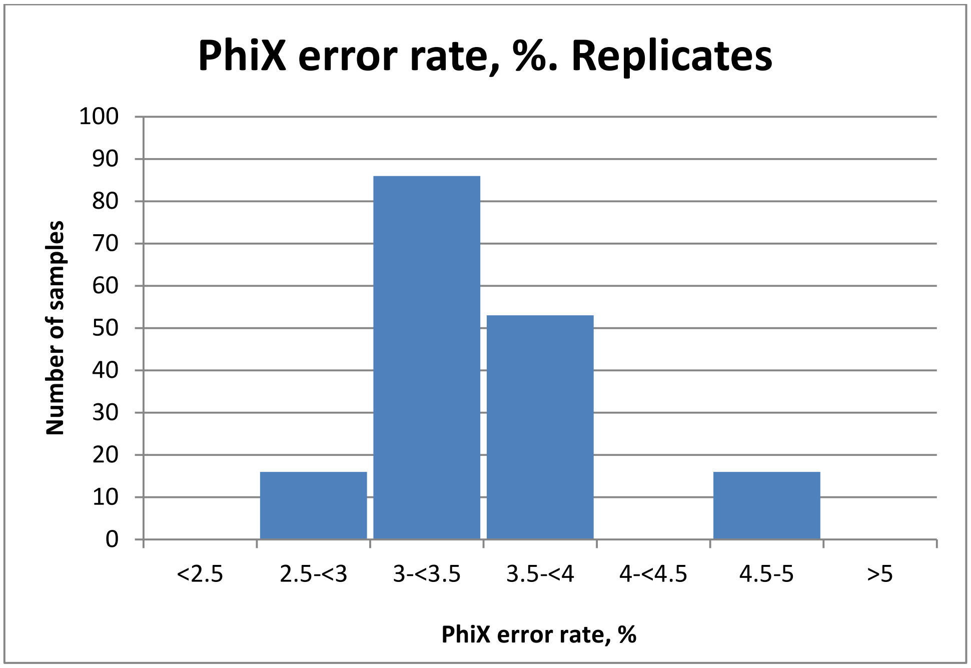

Comparison of parameter's median value for replicates with original median value:

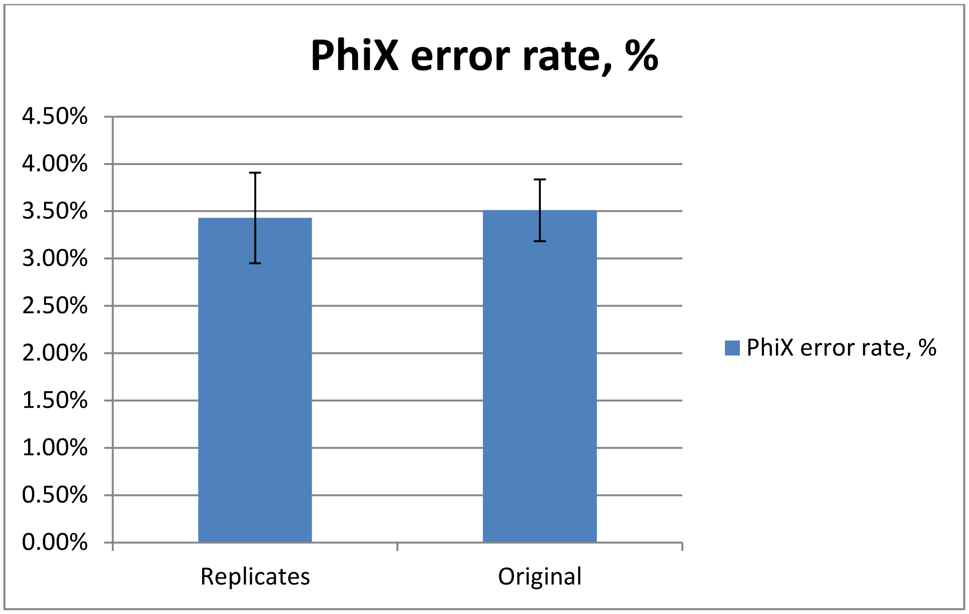

Percent of bases with quality score >Q30 for the run = 59.9-71% (median 62.3%)

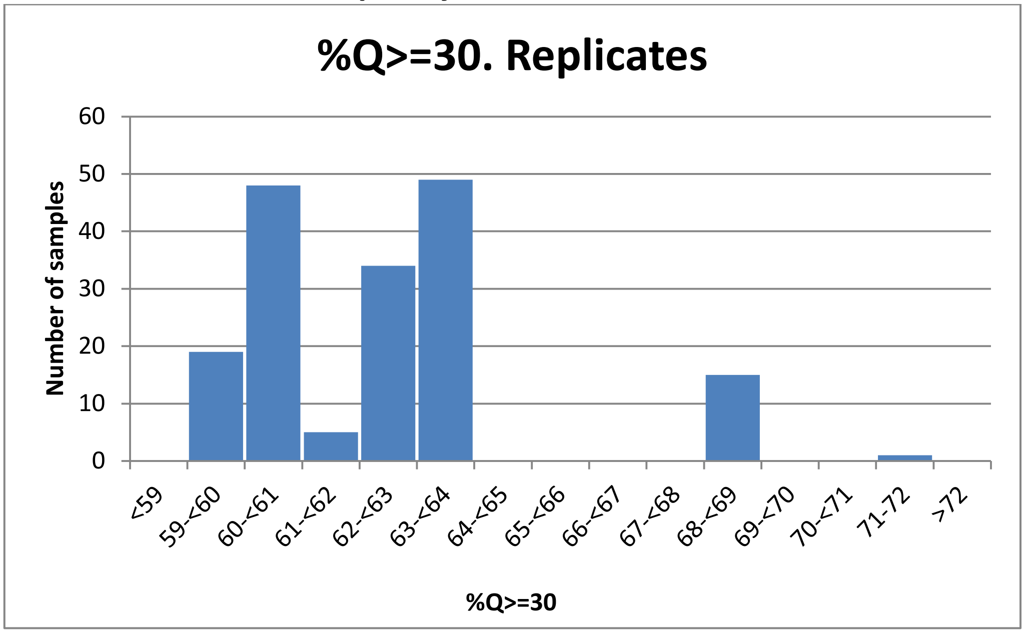

Comparison of parameter's median value for replicates with original median value:

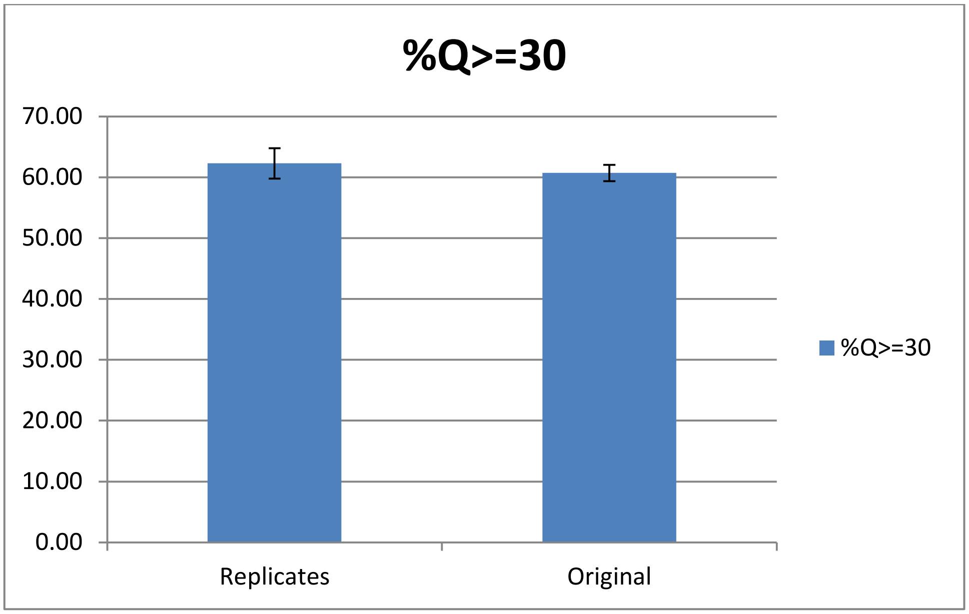

Cluster density for the run = 832-1693K/mm^2 (median 1272K/mm^2)

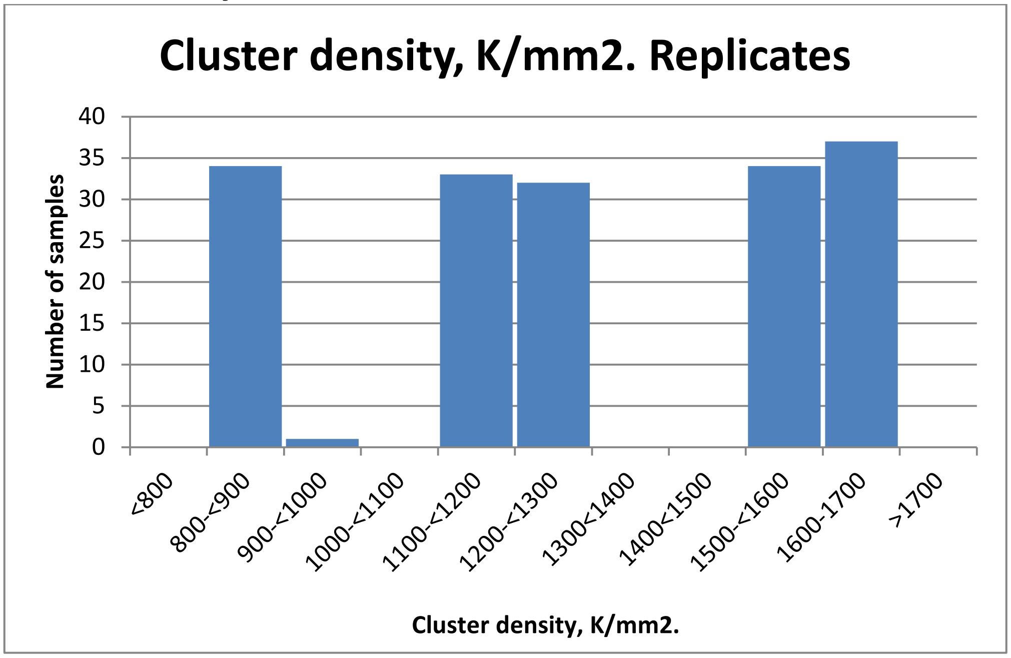

Comparison of parameter's median value for replicates with original median value:

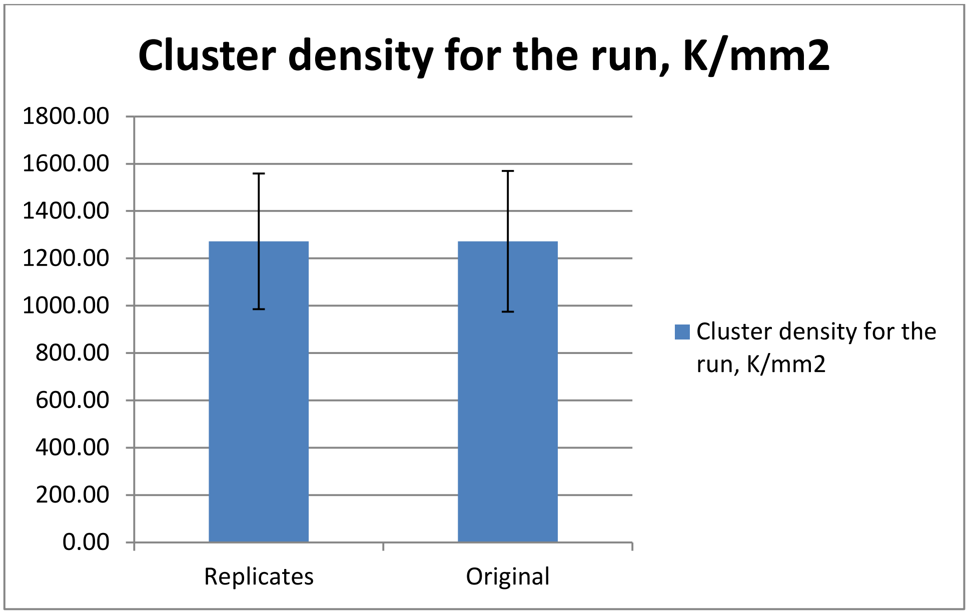

Cluster passing filter of the run = 76.14-95.6% (median 88.9%)

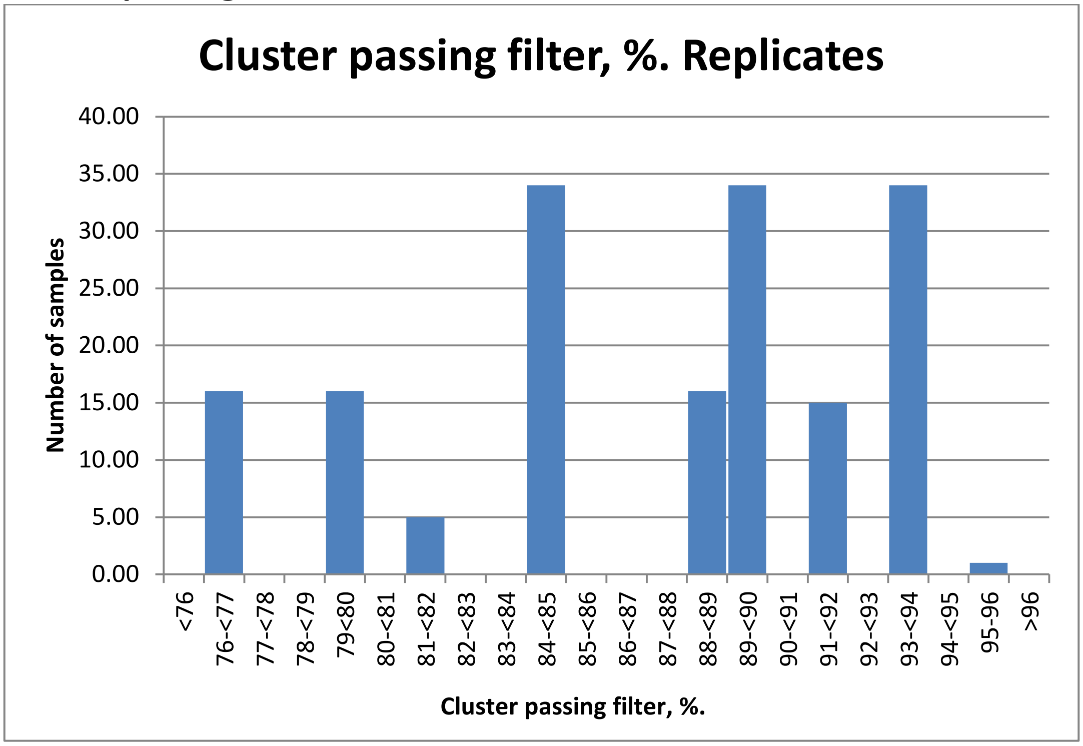

Comparison of parameter's median value for replicates with original median value:

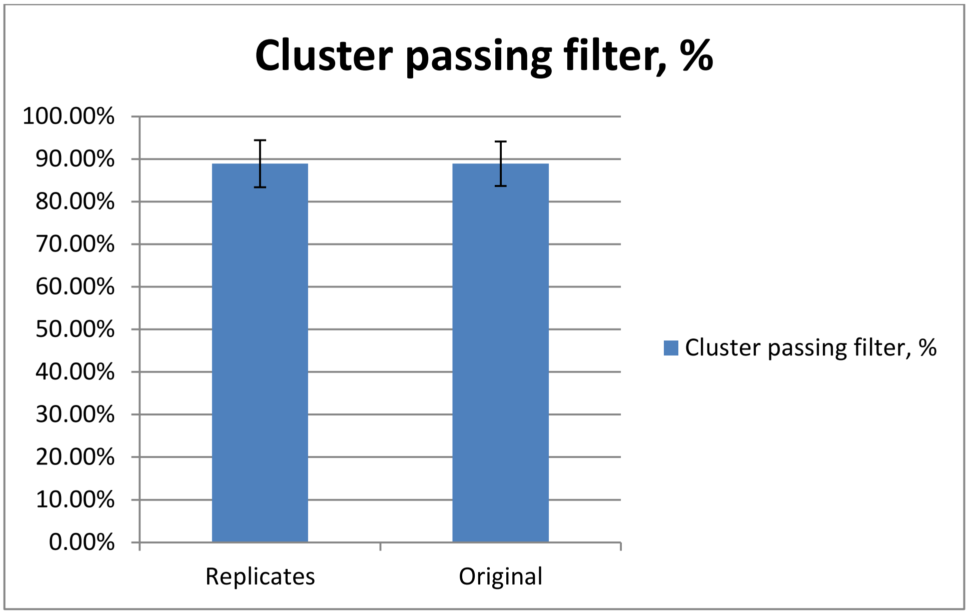

Addition of replicates to the analysis did not change the established median values and ranges for the quality parameters significantly as calculated P-values were >0.05 for all parameters (compared with data in Chapter 8.3.1.1). Below see the P values determined in two-tailed t-test for each parameter:

Q parameter	P value
Uniformity of coverage at 10x	0.691130514
Uniformity of coverage at 5x	0.79616002
Average depth of coverage	0.84418884
Average read length after trimming (with ≥Q30)	0.194259974
Read length at which 75% of bases have quality score ≥Q30	0.801947549
Percentage of genome covered	0.924893934
PhiX error rate (per run)	0.335568896
Percentage of Q score more Q30 (per run)	0.084560057
Cluster density (per run)	0.475706538
Cluster passing filter (per run)	0.38135653

The largest standard deviation within both original dataset and replicates' dataset was observed for two parameters: Depth of coverage of genome and Cluster density of the run. Most likely, the variations in coverage are caused by variability in cluster density. Even though sequences generated within established here ranges (15.71-216.4x Coverage, 832-1693K/mm^2 Cluster density) still provided accurate and reproducible assay results, especial attention should always be paid during final library concentration measurement and library pooling/lading steps, which are the critical steps affecting observed variations.

Repeatability and reproducibility were assessed for 3 assays:

- Genotyping
- *In silico* MLST
- 16S rRNA gene ID

Ultimately, in all 3 assays the repeatability and reproducibility of a single nucleotide variant detection are evaluated, since the precision of single nucleotide variant call determines the results of above-mentioned assays. In addition, quality metrics for all replicates were collected and analyzed.

8.4.1. Repeatability and Reproducibility of Genotyping assay

Repeatability and reproducibility for the genotyping assay can be assessed as inter- and intra-assay precision of single nucleotide variant detection. All validation replicates were mapped against the same reference sequence in order to identify SNPs deferring between the replicates.

Two methods of evaluating precision were used:

1) Evaluation of absolute inter- and intra-assay precision per replicate

2) Evaluation of precision relative to the genome size.

The first method considers the whole genome of one replicate as a single test, meaning that any number of single nucleotide changes in 1 out of 3 of the replicates is regarded as 33.3% disagreement for that validation sample.

The second method of precision estimation is more preferable for WGS validation. Each nucleotide call in the WGS should be considered as an independent test. For that reason, a precision of variant calling should be estimated in relation to the genome size of the sample. Meaning, that a single SNP in one of the replicates should be taken into account as a percentage of the number of base pairs called in the genome sequence of the validation sample.

Precision per replicate:

One out of 3 within-run replicates of isolate C50 *Pseudomonas aeruginosa* ATCC 27853 had a 1 SNP difference from other within-run replicates. All validation samples except C50 yielded identical whole genome sequences for all 3 within-run replicates. The inter-assay precision was 99.02% as per replicate.

$$\text{Inter} - \text{assay precision (Repeatability)} = \frac{\#\ \mathbf{within}\text{−run replicates in agreement}}{\text{Total \# of tests performed for } \mathbf{within}\text{−run replicates}} \times 100\% =$$

$$= \frac{101}{102} \times 100\% = 99.02\%$$

Three validation samples had one of the between-run replicates each differing from other between-run replicates. Sample C47 *Staphylococcus epidermidis* ATCC 12228 had one between-run replicate with 2 SNPs difference from other replicates. Samples C49 *Streptococcus pneumoniae* ATCC 6305 and C55 *Escherichia coli* ATCC 25922 each had one of the between-run replicates differing from other replicated sequences by 1 SNP. Intra-assay precision per replicate was 97.05%.

$$\text{Intra} - \text{assay precision (Reproducibility)} = \frac{\#\ \mathbf{between}\text{−run replicates in agreement}}{\text{Total \# of tests performed for } \mathbf{between}\text{−run replicates}} \times 100\% =$$

$$= \frac{99}{102} \times 100\% = 97.05\%$$

Precision per base pair (*calculated for each sample in the table below****):***

The precision per base pair was calculated for each sample and then the average value was calculated (see below). Estimated precision per base pair (in relation to the covered genome size), both within- and between-run replicates was > 99.99999%.

$$\text{Inter} - \text{assay precision (Repeatability)} =$$

$$\frac{(\text{\# of } \textbf{within}-\text{run replicates x Avg covered genome size}) - (\text{Total \# of SNP difference for } \textbf{within}-\text{run replicates})}{\text{\# of } \textbf{within}-\text{run replicates x Avg covered genome size}} \text{ x } 100\%$$

$$\text{Intra} - \text{assay precision (Reproducibility)} =$$

$$\frac{(\text{\# of } \textbf{between}-\text{run replicates x Avg covered genome size}) - (\text{Total \# of SNP difference for } \textbf{between}-\text{run replicates})}{\text{\# of } \textbf{between}-\text{run replicates x Avg covered genome size}} \text{ x } 100\%$$

Sample	Total # of SNP difference for within-run replicates	Total # of SNP difference for between-run replicated	# of within-run replicates in agreement	# of between-run replicates in agreement	Precision per replicate	Genome size of reference, bp	Length of covered genome, bp	Precision per base pair	
								Inter-assay precision	Intra-assay precision
C1	0	0	3	3	**Inter-assay precision = 99.02% Intra-assay precision =97.05%**	5639400	5244642	100	100
C2	0	0	3	3		4744448	4744448	100	100
C3	0	0	3	3		4746220	4698758	100	100
C4	0	0	3	3		5598800	5486824	100	100
C5	0	0	3	3		2806340	2750213	100	100
C6	0	0	3	3		4964100	4914459	100	100
C46	0	0	3	3		2939973	2939973	100	100
C47	0	2	3	2		2499279	2499279	100	99.99997
C48	0	0	3	3		2516575	2516575	100	100
C49	0	1	3	2		2221315	1954757	100	99.99998
C50	1	0	2	3		6712339	6175352	99.99999	100
C51	0	0	3	3		4989312	4989312	100	100
C52	0	0	3	3		3359001	3359001	100	100
C53	0	0	3	3		1941566	1844488	100	100
C54	0	0	3	3		4233806	3641073	100	100
C55	0	1	3	2		5203440	5047337	100	99.99999
C72	0	0	3	3		5273097	4534863	100	100
C73	0	0	3	3		4685848	4638990	100	100
C74	0	0	3	3		4710675	4569355	100	100
C75	0	0	3	3		4685848	4310980	100	100
C76	0	0	3	3		4685848	4357839	100	100
C103	0	0	3	3		5373121	4567153	100	100
C104	0	0	3	3		1856176	1670558	100	100
C105	0	0	3	3		2492821	2368180	100	100
C106	0	0	3	3		2233640	2121958	100	100
C56	0	0	3	3		4411532	4279186	100	100
C57	0	0	3	3		4411532	4279186	100	100
C58	0	0	3	3		4411532	4279186	100	100
C59	0	0	3	3		4411532	4323301	100	100
C61	0	0	3	3		4411532	4279186	100	100
C65	0	0	3	3		4411532	4323301	100	100
C67	0	0	3	3		4411532	4323301	100	100
C68	0	0	3	3		4411532	4323301	100	100
C69	0	0	3	3		4411532	4323301	100	100
Total	1	4	101	99			Average:	**99.99999**	**99.99999**

The discrepancies between replicates in this table are highlighted with orange.

P.S.: To retrieve Genome size information from NCBI Genome site- see instructions in Appendix 3. See Appendix 5 chapter data interpretation for instructions for collecting data on Total # of SNP difference for within-run replicates, Total # of SNP difference for

between-run replicated, # of within-run replicates in agreement, # of between-run replicates in agreement. See instructions in Appendix 4 to calculate average covered genome size.

8.4.2. Repeatability and Reproducibility for *in silico* MLST assay

Repeatability and Reproducibility of nucleotide base calling for *in silico* MLST assay were assessed. For that sequences of replicates for 21 samples were *de novo* assembled and used for MLST typing using CGE MLST online tool (as described above). A single change in nucleotide sequence of any of the housekeeping genes used in MLST scheme leads to change in allele number and corresponding Sequence type (ST) change. Within- and between run replicates must have reproducible sequence of all housekeeping genes resulting in a reproducible ST assignment.

Detection and correct identification of each of the MLST alleles in the typing scheme represents an independent test. For each replicate sample 7 tests of allele identification were performed (except C2 sample which had 6 tests and C53 which had 8 tests due to typing schemes difference). For MLST total number of alleles analyzed for either within- or between-run replicates was 441. Repeatability here reflects MLST allele detection precision for **within**-run replicates. Reproducibility reflects the precision of MLST-alleles detection for **between**-run replicates.

Inter-assay precision (Repeatability)=

Total # MLST alleles analyzed for **within**-run replicates - # of alleles difference for **within**-run replicates
____ x 100%=
Total # MLST alleles analyzed for **within**-run replicates

$= \frac{441-0}{441}$ x 100% = 100%

Intra-assay precision (Reproducibility) =

Total # MLST alleles analyzed for **between**-run replicates - # of alleles difference for **between**-run replicates
____ x 100%=
Total # MLST alleles analyzed for **between**-run replicates

$= \frac{441-0}{441}$ x 100% = 100%

Sample	Consensus ST	Alternative ST detected or unidentified ST	# of alleles per replicate	Total # alleles analyzed for within-run replicates	# of alleles difference for within-run replicates	Total # alleles analyzed for between-run replicates	# of alleles difference for between-run replicates	Repeatability	Reproducibility
C1	ST-11	no	7	21	0	21	0	100%	100%
C2	ST-1	no	6	18	0	18	0		
C3	ST-3021	no	7	21	0	21	0		
C4	ST-1	no	7	21	0	21	0		
C5	ST-243	no	7	21	0	21	0		
C6	ST-19	no	7	21	0	21	0		
C46	ST-30	no	7	21	0	21	0		
C47	ST-8	no	7	21	0	21	0		
C49	ST-4840	no	7	21	0	21	0		
C50	ST-155	no	7	21	0	21	0		
C51	ST-14	no	7	21	0	21	0		
C53	ST-98	no	8	24	0	24	0		
C54	ST-836	no	7	21	0	21	0		
C55	ST-73	no	7	21	0	21	0		
C73	ST-11	no	7	21	0	21	0		
C74	ST-32	no	7	21	0	21	0		
C75	ST-440	no	7	21	0	21	0		
C76	ST-592	no	7	21	0	21	0		
C72	ST-655	no	7	21	0	21	0		
C104	ST-44	no	7	21	0	21	0		
C106	ST-11075	no	7	21	0	21	0		
			Total:	441	0	441	0		

See results of *in silico* MLST typing for all replicates in the Appendix 10.

Each single allele in all validation samples was identified consistently among within- and between-run replicates. Within and between run precisions of allele detection were 100%.

8.4.3. Repeatability and Reproducibility in 16S rRNA gene ID assay

Repeatability and Reproducibility of for 16S rRNA gene identification assay were assessed. 16S rRNA gene sequences were extracted from genomes of validation replicates for 34 samples and identified using RDP database as mentioned above. Repeatability here reflects 16S rRNA gene identifications precision for **within**-run replicates. Reproducibility reflects the precision of 16S rRNA gene identifications for **between**-run replicates.

$$\text{Inter} - \text{assay precision (Repeatability)} = \frac{\#\ \textbf{within}-\text{run replicates in agreement for 16S ID assay}}{\text{Total \# of 16S ID tests performed for } \textbf{within}-\text{run replicates}} \text{ x } 100\% =$$

$$= \frac{102}{102} \text{ x } 100\% = 100\%$$

$$\text{Intra} - \text{assay precision (Reproducibility)} = \frac{\#\ \textbf{between}-\text{run replicates in agreement for 16S ID assay}}{\text{Total \# of 16S ID tests performed for } \textbf{between}-\text{run replicates}} \text{ x } 100\% =$$

$$= \frac{102}{102} \text{ x } 100\% = 100\%$$

Sample	Consensus ID	# of within-run replicates in agreement	# of differing ID results for within-run replicates	# of between-run replicates in agreement	# of differing ID results for between-run replicates	Alternative ID results or unidentified	Repeatability	Reproducibility
C1	*Escherichia coli*	3	0	3	0	none	100%	100%
C2	*Aeromonas hydrophila*	3	0	3	0	none		
C3	*Escherichia coli*	3	0	3	0	none		
C4	*Enterobacter cloacae*	3	0	3	0	none		
C5	*Staphylococcus aureus*	3	0	3	0	none		
C6	*Salmonella enterica*	3	0	3	0	none		
C46	*Enterococcus faecalis*	3	0	3	0	none		
C47	*Staphylococcus epidermidis*	3	0	3	0	none		
C48	*Staphylococcus saprophyticus*	3	0	3	0	none		
C49	*Streptococcus pneumoniae*	3	0	3	0	none		
C50	*Pseudomonas aeruginosa*	3	0	3	0	none		
C51	*Stenotrophomonas maltophilia*	3	0	3	0	none		
C52	*Legionella pneumophila*	3	0	3	0	none		
C53	*Moraxella catarrhalis*	3	0	3	0	none		
C54	*Acinetobacter baumannii*	3	0	3	0	none		
C55	*Escherichia coli*	3	0	3	0	none		
C72	*Escherichia coli*	3	0	3	0	none		
C73	*Salmonella enterica*	3	0	3	0	none		
C74	*Salmonella enterica*	3	0	3	0	none		
C75	*Salmonella enterica*	3	0	3	0	none		
C76	*Salmonella enterica*	3	0	3	0	none		
C103	*Bacteroides fragilis*	3	0	3	0	none		
C104	*Haemophilus influenzae*	3	0	3	0	none		
C105	*Corynebacterium jeikeium*	3	0	3	0	none		
C106	*Neisseria gonorrhoeae*	3	0	3	0	none		
C56	*Mycobacterium tuberculosis*	3	0	3	0	none		
C57	*Mycobacterium tuberculosis*	3	0	3	0	none		
C58	*Mycobacterium tuberculosis*	3	0	3	0	none		
C59	*Mycobacterium tuberculosis*	3	0	3	0	none		
C61	*Mycobacterium tuberculosis*	3	0	3	0	none		
C65	*Mycobacterium tuberculosis*	3	0	3	0	none		
C67	*Mycobacterium tuberculosis*	3	0	3	0	none		
C68	*Mycobacterium tuberculosis*	3	0	3	0	none		
C69	*Mycobacterium tuberculosis*	3	0	3	0	none		
Total:		102	0	102	0			

See results of 16S rRNA ID for all replicates in the Appendix 11.

Within- and between run replicates had repeatable/reproducible sequences of 16S rRNA gene and resulted in repeatable/reproducible species identification. Within and between run precisions of species identification were 100%.

8.5. Analytical sensitivity and Analytical specificity

8.5.1. Analytical sensitivity of SNP detection

8.5.1.1. Approach

Analytical sensitivity (Limit of Detection; LOD) of SNP calling was estimated by modeling different mapping coverages and estimating the minimum coverage which allows for accurate SNP calling (LOD_{SNP}). Mapping bam files were downsampled in order to achieve different coverage values (60x, 50x, 40x, 30x, 20x, 15x, 10x, 5x) for each of the 9 samples representative of different species. The original sequence mapping coverage was estimated from bam file using the following command:

```
samtools depth  bamfile_sorted.bam  |  awk '{sum+=$3} END { print "Average = ",sum/NR}'
```

The bam files were downsampled to a desired coverage using the following command:

```
samtools view -h -s F bamfile_sorted.bam >bamfile_sorted_30x.bam
```

where F is a fraction of desired coverage in relation to the original coverage.

Number of SNPs detected between the reference sequence and downsampled bam file was compared to the number of SNPs detected between reference and sample at its original coverage.

8.5.1.2. Limit of SNP detection

Below the SNPs detected in the downsampled and original samples are presented. The sequences subjected to downsampling are marked with corresponding coverage values in front of the sequence ID.

Sample C3_2b

		Number of SNPs between reference and sample
5x_C3dup_S8_L001_R1_001	-GA GGT AGA -AC AC- -TC C-- -	15
10x_C3dup_S8_L001_R1_001	-GA GGT AGA -AC AC- -TC CGT -	17
15x_C3dup_S8_L001_R1_001	-GA GGT AGA -AC AC- -TC CGT -	17
20x_C3dup_S8_L001_R1_001	-GA GGT AGA -AC AC- -TC CCG T	18
30x_C3dup_S8_L001_R1_001	-GA GGT AGA CAC AC- -TC CCG T	19
40x_C3dup_S8_L001_R1_001	-GA GGT AGA CAC ACG -TC CCG T	20
50x_C3dup_S8_L001_R1_001	-GA GGT AGA CAC ACG GTC CCG T	21
60x_C3dup_S8_L001_R1_001	AGA GGT AGA CAC ACG GTC CCG T	22
Originial_C3dup_S8_L001_R1_001	AGA GGT AGA CAC ACG GTC CCG T	22

For the rest of the samples only the results for lowest "accurate" coverage and highest "inaccurate" coverage are shown in the Appendix 16.

Below is the summary of the SNP detection at different coverage:

Sample ID	Species	Original coverage	Number of SNP detected between the validation sample and reference								
			At original coverage	60x	50x	40x	30x	20x	15x	10x	5x
C1_3b	*Escherichia coli* O157:H7	69.3x	5	5	5	5	5	5	4	4	4
C2_2a	*Aeromonas hydrophilia*	116.5x	1	1	1	1	1	1	1	1	1
C3_2b	*Escherichia coli*	85.2x	22	22	21	20	19	18	17	17	15
C5_1	*Staphylococcus aureus*	145x	0	0	0	0	0	0	0	0	0
C6_3a	*Salmonella enterica* ser Typhimurium	86.6x	12	12	12	8	8	7	7	7	7
C46_3	*Enterococcus faecalis*	58x	3	-	3	3	3	3	3	3	4
C52_2	*Legionella pneumophila*	99.8x	2	2	2	2	2	2	2	2	2
C72_2	*Escherichia coli* O121:H19	53x	0	-	0	0	0	0	0	0	0
C73_1c	*Salmonella enterica* ser Enteritidis	76.8x	0	0	0	0	0	0	0	0	0

The LOD_{SNP} was established at 60x as it was the lowest coverage which yielded accurate SNP detection in all samples.

8.5.2. Analytical specificity of SNP detection

8.5.2.1. Approach

To determine the analytical specificity, we *in silico* generated the sequencing files containing mixture of the reads from 2 different samples thus mimicking a contamination. The effect of potentially interfering sequencing reads on mapping metrics (% of reads mapped/not mapped, % of reference covered, etc.) and SNP detection was estimated. Sample C3 *Escherichia coli* ATCC 8739 was selected as a base not-contaminated sample and equal parts of reads from different species were merged with it to generate mixed fastq files. "Contaminated" and "not-contaminated" reads were mapped to the same reference genome of *E. coli* ATCC 8739 from NCBI. Further SNP calling analysis was performed using the standard pipeline under validation.

8.5.2.2. Mapping metrics

Following mapping quality parameters were estimated for original not-contaminated sample *Escherichia coli* C3 and different combinations of *E.coli* C3 with contaminating samples:

**Samples**	C3 *E.coli*	C3 *E.coli* + C75 *Salmonella enterica*	C3 *E.coli* + C1 *E.coli*	C3 *E.coli* + C54 *Acinetobacter baumannii*	C3 *E.coli* + C57 *Mycobacterium tuberculosis*	C3 *E.coli* + C5 *Staphylococcus aureus*
**% of Contamination reads**	no contamination	50%	50%	50%	50%	50%
**% of Mapped reads**	99%	68%	91%	51%	67%	49%
**% of Not mapped reads**	1%	32%	9%	49%	33%	51%
**% of Reads in pairs**	90%	59.60%	81%	48.30%	60%	46.60%
**% of Broken paired reads**	9%	8%	10%	3%	6%	3%
**% of reference covered**	99%	99%	99%	99%	99%	99%
**Average coverage**	86.54x	124.96x	115.15x	152.59x	150.73x	152.04x

As expected, contaminating reads led to decrease in percentage of mapped reads and increase in portion of unmapped reads. Percent of reads in pairs has decreased in samples containing contaminating reads. Mentioned parameters should be monitored in future routine samples to detect potential contamination.

8.5.2.3. Specificity of SNP calling

Specificity of SNP calling in the samples containing potentially interfering reads was estimated by comparing:

a) number of SNPs detected between "contaminated" sequence and reference

b) number of SNPs detected between "contaminated" and "not-contaminated" sequence included into the same SNP calling analysis.

**Samples**	C3 *E.coli*	C3 *E.coli* + C75 *Salmonella enterica*	C3 *E.coli* + C1 *E.coli*	C3 *E.coli* + C54 *Acinetobacter baumannii*	C3 *E.coli* + C57 *Mycobacterium tuberculosis*	C3 *E.coli* + C5 *Staphylococcus aureus*
SNPs between sequence and reference	22	24	22	40	22	36
Number of missed SNPs	NA	1	0	0	0	0
Number of nonspecific SNPs	NA	1	0	18	0	14
SNPs between "contaminated" and "not-contaminated" sequence	NA	2	0	18	0	14

Difference between original not-contaminated C3 *E. coli* sequences (all 5 replicates) and the reference NCBI genome was 22 SNPs. C1 *E. coli* and C57 *M. tuberculosis* contaminating reads didn't cause any change in called SNPs. Contamination with any of the other reads led to additional SNPs called both between the compared samples and with the reference. In sample contaminated with C75 *S. enterica,* in addition to nonspecific SNPs, one of the SNP detected previously was missed. The bioinformatics pipeline had certain tolerance to contaminating reads depending of the nature of contamination. Monitoring mapping metrics will be the best way to track contamination.

8.6. Diagnostic sensitivity and Diagnostic specificity

Diagnostic sensitivity- likelihood that an assay will detect a sequence variation when present within the analyzed genomic region (this value reflects a false negative rate of the assay) [21].

Diagnostic specificity- the probability that a NGS assay will not detect sequence variation(s) when none are present within the analyzed genomic region (this value reflects a false positive rate of the assay) [21].

Diagnostic sensitivity is also referred to as "Limit of detection", which is normally for molecular tests designates the lowest amount of nucleic acid that can be detected by the assay. Limit of detection in this sense is not applicable to WGS applications, which utilize pure bacterial culture as starting material, since the amount of DNA which goes into the reaction is strictly standardized and DNA concentration of each sample is measured by fluorometric method before each assay. Starting DNA input for all validation samples in this protocol was 1ng, at input DNA extract concentration of 0.2ng/μl. Samples with a concentration less than 1ng/μl shouldn't be processed for library preparation.

Diagnostic sensitivity and specificity of WGS were estimated in two assays: MLST and Genotyping.

8.6.1. Diagnostic sensitivity and specificity of *in silico* MLST assay

As described above, using organism-specific MLST databases sequence type of validation sequences and their reference sequences was determined. Identities of all alleles defining sequence type (ST) were confirmed.

For MLST number of the true positive results corresponds to the number of alleles correctly identified in the validation samples. For the true negative results we performed a comparison of validation sequences against MLST databases for not-matching species, e.g. search of alleles for C1 *Escherichia coli* validation sample against MLST database for *Salmonella enterica*. In the latter case, the MLST assay is not supposed to be able to identify any alleles, and definitively not to assign the sequence type, otherwise it would be counted as a false positive result.

True positive results (TP) = Number of correctly identified alleles in all validation samples combined = 104
True negative results (TN) = Number of unidentified alleles in negative control samples = 35
False positive (FP) = Number of identified alleles in negative control samples = 0
False negative (FP) = Number of unidentified or misidentified alleles validation samples= 0

Sensitivity = $\frac{\mathbf{TP}}{\mathbf{TP+FN}}$ **x 100%** = $\frac{104}{104+0}$ x 100% = 100%

Specificity = $\frac{\mathbf{TN}}{\mathbf{TN+FP}}$ **x 100%** = $\frac{35}{35+0}$ x 100% = 100%

All alleles in positive validation samples were identified correctly:

Validation Sample	Reference ST	Validation sample ST	# of allele total	# of identified correctly	# of identified incorrectly	Results
C1	ST-11	ST-11	7	7	0	pos
C2	ST-1	ST-1	6	6	0	pos
C3	ST-3021	ST-3021	7	7	0	pos
C4	ST-1	ST-1	7	7	0	pos
C5	ST-243	ST-243	7	7	0	pos
C6	ST-19	ST-19	7	7	0	pos
C46	ST-30	ST-30	7	7	0	pos
C47	ST-8	ST-8	7	7	0	pos
C51	ST-14	ST-14	7	7	0	pos
C55	ST-73	ST-73	7	7	0	pos
C73	ST-11	ST-11	7	7	0	pos
C74	ST-32	ST-32	7	7	0	pos
C75	ST-440	ST-440	7	7	0	pos
C76	ST-592	ST-592	7	7	0	pos
C72	ST-655	ST-655	7	7	0	pos

None of the negative controls were assigned to any ST. None of the alleles in negative controls were identified:

Negative control

		Negative control					
	**MLST database**	**Sample ID**	**Species**	**# of allele total**	**# of alleles matching negative control MLST profile**	**Results**	
1	*Salmonella enterica*	C1	*Escherichia coli*	7	0	unknown ST	neg
2	*Staphylococcus aureus*	C46	*Enterococcus faecalis*	7	0	unknown ST	neg
3	*Pseudomonas aeruginosa*	C51	*Stenotrophomonas maltophilia*	7	0	unknown ST	neg
4	*Escherichia coli #1*	C73	*Salmonella enterica*	7	0	unknown ST	neg
5	*Staphylococcus epidermidis*	C5	*Staphylococcus aureus*	7	0	unknown ST	neg

Both diagnostic sensitivity and diagnostic specificity were 100%.

8.6.2. Diagnostic sensitivity and specificity of Genotyping assay

In order to estimate diagnostic sensitivity and specificity of WGS-based genotyping, the hqSNPs difference between the strains was established to build a phylogenetic tree. Trees generated from the validation sequences were compared to the trees generated from the reference sequences for the same strain. See Appendix 1 for the results. Genotyping using WGS-derived hqSNPs was performed as described above in chapter “Accuracy of Genotyping assay”.

Diagnostic sensitivity in genotyping assay. Diagnostic sensitivity for genotyping test is the likelihood that all the SNPs differing between the isolates will be detected. In case of WGS-based genotyping, each sequence variation (SNP) which wasn’t detected represents a *false negative* result. Missed sequence variations manifest as a decrease in genetic distance between tested strains and would make samples appear to be closer to each other in comparison with the same samples clustering in reference tree. See the figure below:

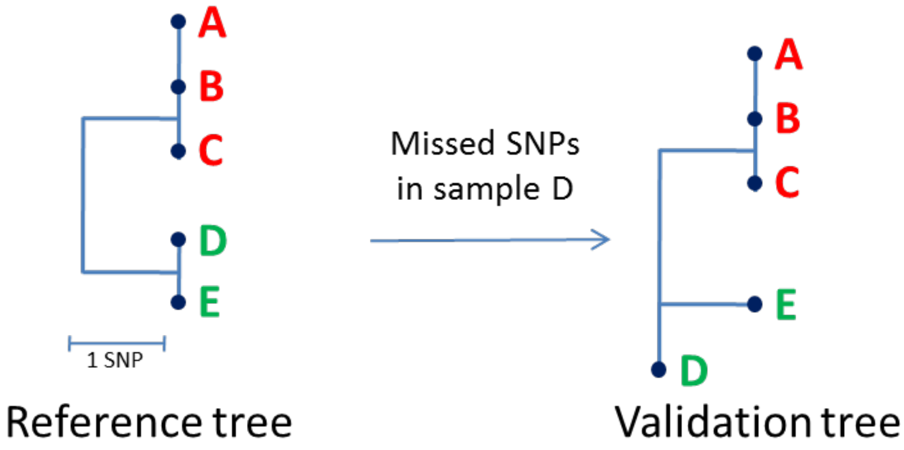

Explanation:

<u>Effect of false negative SNP on genotyping results.</u> Example: samples A-B-C are identical to each other and samples D and E are identical to each other, while these two groups differ by a certain number of SNPs (e.g. 1 SNP, for simplicity) and form two clusters (red and green). If sample D has false negative SNPs, meaning that SNPs which distinguished it from the "red cluster" A-B-C were missed, the distance between Sample D to A-B-C cluster would by artificially reduced. In a case of an outbreak situation, the false negative SNP call may lead to erroneous inclusion of the sample into the outbreak cluster.

For the genotyping test performed here, the correct result would be when the clustering of whole genome sequences generated by the Core lab result in an analogous phylogenetic tree as one generated from reference sequences available for these samples. Samples which clustered in separate groups must replicate their cluster division and genetic distance, indicating that all the SNPs were detected and none were missed.

For example, Saur_sample4 on the figure below is *Staphylococcus aureus* ATCC 25923 strain:

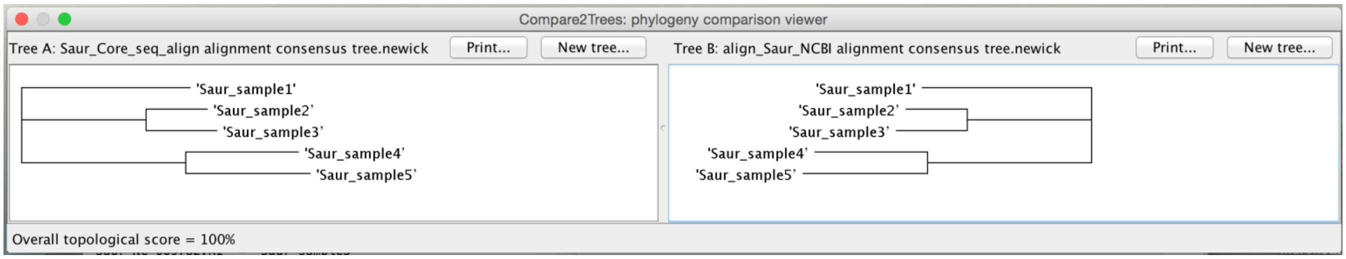

In tree on the left Saur_sample4 is represented by a sequence generated in Core laboratory as a part of validation. In the tree on the right Saur_sample4 is represented by reference sequence from NCBI. Other samples in both trees are NCBI sequences. False negative SNP calling results in the Saur_sample4 sequenced by

Core lab would lead to under-detection of SNPs in validation sample. This in turn would reduce the number of SNPs which are different between Saur_sample4 and Saur_sample5 or other samples. Reduction in SNP difference will result in Saur_sample4 in Core lab tree appearing closer to other isolates than in NCBI reference tree. From the comparison below we can see that clustering between Saur_sample4 and other samples is identical in both trees, suggesting that no false negative SNP calls affected genotyping results. Moreover, in a case of false negative SNP calling, a topological score of validation tree would be changed. According to validation vs reference tree comparison using Compare2Trees software tool all validation trees 100% matched reference trees, indicating absence false negative results in genotyping test.

Diagnostic specificity in genotyping assay. Diagnostic specificity for genotyping test is the likelihood that variation between the isolates (SNP) will not be detected when none are present. In the case of whole genome sequencing based genotyping, changes introduced in DNA sequence during library prep/sequencing, or data analysis errors can result in *false positive* sequence variations.

False positive SNP calls will lead to an increase in genetic distance between validation strains, in comparison with the distance between reference genomes. Consequently, samples would appear to be further apart from each other in comparison with the reference tree:

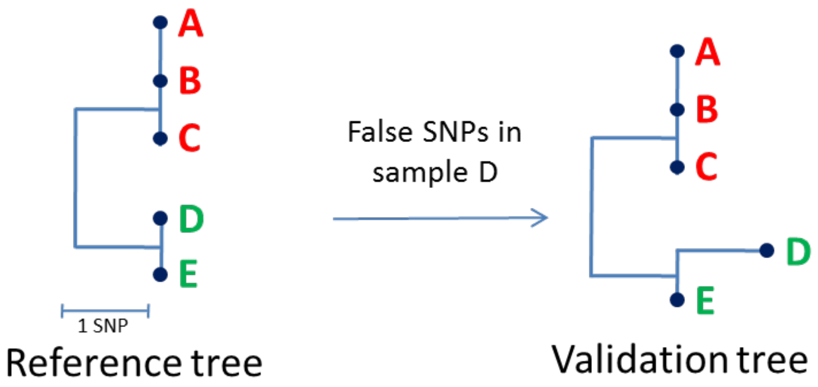

Explanation:

<u>*Effect of false positive SNP on genotyping results.*</u> Example: identical samples A-B-C form the red cluster, another group of identical isolates E & D form another cluster (green). If sample D has false positive SNPs, it will make it look more distant from sample E and other samples, in comparison with reference tree. In a case of an outbreak situation, the false positive SNP call may lead to the incorrect exclusion of the sample from the outbreak cluster.

For the genotyping test, clustering of sequences generated during validation should repeat clustering results for reference sequences. In other words, several samples clustered as a single group according to reference sequences must fall into one group after clustering of validation sequences. If one or more samples appear to be excluded from the group upon resequencing this indicates presence of false positive SNPs

For example, Smalt_sample4 on the figure below is *Stenotrophomonas maltophilia* ATCC 13637 strain:

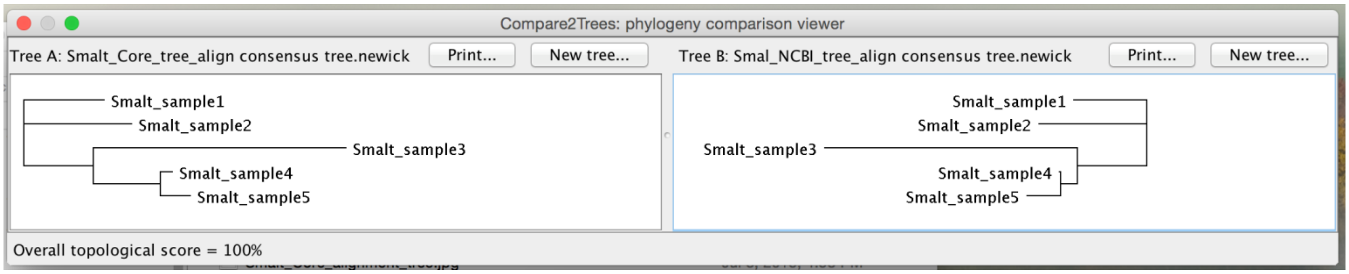

In the tree on the left Smalt_sample4 is represented by a sequence generated in Core laboratory as part of validation. In the tree on the right Smalt_sample4 is represented by reference sequence from NCBI. Other samples in both trees are NCBI sequences. False positive SNP calling results in the Smalt_sample4 sequenced by Core lab would likely lead to increase in the number of SNPs which are different between Smalt_sample4 and Smalt_sample5. False positive SNP will result in Smalt_sample4 in Core lab tree appearing further away from Smalt_sample5 than in NCBI reference tree, or Smalt_sample4 will not cluster together with Smalt_sample5. From the comparison below we can see that clustering between Saur_sample4 and other samples is identical in both trees, particularly Saur_sample4 remains clustered together Saur_sample5. Thus, no false positive SNP calls affected genotyping results. In a case of false positive SNP calling, a topological score of validation tree would be changed. According to validation vs reference tree comparison using Compare2Trees software tool all validation trees 100% matched reference trees, indicating absence false positive results in genotyping test.

<u>Formulas for calculation of the diagnostic sensitivity and specificity:</u>

True positive results (TP) = **True negative results (TN)** = Number of validation samples with clustering results matching reference = 11

False positive (FP) = Number of validation samples which failed to clustered together with samples, genetically similar according to the reference tree= 0

False negative (FP) = Number of validation samples which clustered together with samples, genetically distant according to the reference tree= 0

Sensitivity = $\frac{\mathbf{TP}}{\mathbf{TP+FN}}$ x 100% = $\frac{11}{11+0}$ x 100% = 100%

Specificity = $\frac{\mathbf{TN}}{\mathbf{TN+FP}}$ x 100% = $\frac{11}{11+0}$ x 100% = 100%

All generated validation trees repeated clustering and had 100% of topological similarity with corresponding reference trees. Both diagnostic sensitivity and diagnostic specificity of the hqSNP-based genotyping assay were 100%.

8.7. Reportable range for WGS

CLIA defines the reportable range as "the span of test result values over which the laboratory can establish or verify the accuracy of the instrument or test system measurement response". For NGS used for human genetic disease diagnosis, the reportable range has been previously defined as the portion of the genome for which sequence information can be reliably derived for a defined test system [21]. Presented here validation evaluated following portions of the genome which can be included into reportable range:

- Genome-wide hq SNPs
- Housekeeping genes used in MLST schemes
- 16S rRNA gene
- Antibiotic resistance genes in included ResFinder database

Reference range is not applicable to WGS since the method is not quantitative.

8.8. Summary of Performance Specifications of the assay

Performance Specification			Expected	Observed	Outcome
**Accuracy**	Accuracy of platform (per base)		≥ 90%	99.9993781%	Passed
	Accuracy of assay	MLST		100%	Passed
		16S rRNA ID		100%	Passed
		Genotyping		100%	Passed
		Antibiotic resistance genes detection		100%	Passed
	Accuracy of bioinformatics pipeline			100%	Passed
Reproducibility (precision between runs)		per base	≥ 90%	99.99999%	Passed
		MLST		100%	Passed
		16S rRNA ID		100%	Passed
Repeatability (precision within run)		per base	≥ 90%	99.99999%	Passed
		MLST		100%	Passed
		16S rRNA ID		100%	Passed
Specificity		MLST	≥ 90%	100%	Passed
		Genotyping		100%	Passed
Sensitivity		MLST	≥ 90%	100%	Passed
		Genotyping		100%	Passed

Conclusion: Whole genome sequencing assay was validated by Core laboratory. Following applications were chosen to evaluate the ability of WGS assay to perform accurate, reproducible, specific and sensitive base calling: hqSNP-based genotyping, in silico MLST, 16S rRNA ID, and antibiotic resistance genes detection. LOD was established at 60x coverage. Contaminating reads may cause reduction in analytical specificity of SNPs calling therefore mapping quality metrics must be monitored. WGS assay was shown to have >99.9% accuracy, >99.9% reproducibility/repeatability, and 100% specificity and sensitivity, which meets CLIA requirements for laboratory-developed tests. WGS method can be implemented by the MDL the laboratory for tested applications.

Discrepancies in SNPs detected during the validation between generated sequences and NCBI genomes were selectively confirmed by Sanger sequencing. No discrepancies were detected for the 16S rRNA ID, MLST, or resistance genes detection assays; therefore, confirmatory testing was not required.

9. Summary of quality assurance (QA) and quality control (QC) measures developed during validation

The quality assurance (QA) and quality control (QC) measures were developed as the results of valuation to ensure high quality and consistency of further routine testing using MiSeq Illumina platform. QC must be performed during both pre-analytical (DNA isolation, library preparation), analytical (quality metrics of sequencing run) and post-analytical (data analysis) steps of the WGS. On the stage of data analysis QC includes 3 steps: raw read QC, mapping quality QC (or/and *de novo* assembly QC), variant calling QC.

The laboratory should use the WGS validation to establish the thresholds of quality parameters, which can be used in following routine testing to filter out poor quality samples and data and this way minimize a chance of false results.

We suggest using spiked-in positive and negative controls for routine testing as well as more comprehensive monthly positive and negative controls. Since traditional CLIA rules require the positive and negative control to pass through all the pre-analytical steps, including DNA isolation, laboratory may choose to follow this guidance and perform DNA isolation and sequencing of positive and negative control in each run, or alternatively, implement Individualized Quality Control Plan (IQCP) [as per 42CFR493.1250] and use more economical spiked-in control instead. Type and complexity of positive and negative controls should be determined by each laboratory individually based on specifics of their workflow (most probable source of contamination), type of microorganisms and assays which are most commonly used.

The passing quality thresholds for the controls, particularly the acceptable level of contamination in negative controls (which is unavoidable), would have to be established as well. Our analytical specificity analysis showed that the SNP calling bioinformatics pipeline could be tolerant to a certain degree of contamination and still produce accurate assay results. As per CLIA, failure of either positive or negative control to meet minimum quality thresholds requires rejection and repeat of the entire sequencing run. For the laboratories processing large volumes of samples, especially when libraries are processed in 96-well plate format, it would be a good idea to consider including mix-up control for correct positioning of a known sample.

Here are the highlights of the proposed QA&QC procedures:

1. Every run QC should be performed by monitoring following quality metrics:
    a. Quality of the input DNA for all tested samples.
    b. Quantity of the input DNA for all tested samples.
    c. DNA library size distribution for representative samples.
    d. DNA library concentration for all tested samples.
    e. Run metrics:
        i. Percent of bases with quality score >Q30 for the run.
        ii. Cluster density for the run.
        iii. Cluster passing filter of the run.
    f. Quality of the sequences for all tested samples:

i. If submitter's ID is available, match the 16s rRNA ID with the submitter's information in order to check for the potential sample switch-over. If the WGS-based 16S ID doesn't match submitter's ID investigate prior to reporting the results of WGS.
ii. Average depth of the genome coverage.
iii. Accuracy of base calling (Read length with quality score ≥Q30).
iv. Mapping QC: Uniformity of coverage, Percentage of genome covered.
v. Quality of *de novo* assemblies: Min contig coverage, Min contig length .
vi. Variant calling QC: Min SNP coverage, Min SNP quality, SNP support. It is a good practice to view the vcf alignment and see if there are samples which have multiple SNPs localized to a single area. Double-check if those SNP differences are not caused by bacteriophage insertion or recombination event.

g. Quality of spiked in positive PhiX control sequences.
h. Quality of negative sequencing process/analysis control:
   i. To control for contamination during the library preparation and sequencing process, index combination which doesn't correspond to any sample in the current sequencing run can be added to the indexes demultiplexing step. Index combination for negative control should correspond to one of the index combinations used in the previous sequencing run, this way it would capture carry over contamination with the library fragments generated in the previous run. If the negative control doesn't meet established quality parameters, this indicates a possibility of carry over contamination with the library fragments generated in the previous run.
   ii. In a case of genotyping, epidemiologically unrelated strain of the same species as a pathogen caused potential outbreak should be included in the analysis as a negative control. Epidemiologically unrelated negative control should not cluster with tested samples on the phylogenetic tree.

2. Monthly QC should be performed by using following positive and negative controls, which have to be included into the WGS process starting from the DNA extraction step all the way to the data analysis:
   a. Select a well-characterized strain, e.g. *Escherichia coli* ATCC 25922, as a monthly positive control. If different DNA extraction protocols are used, it is recommended to include additional positive controls which would control for different procedures (e.g. *Listeria monocytogenes*– for Gram-positive DNA extraction protocol, *Mycobacterium tuberculosis*– for TB-specific DNA-extraction protocol). Additional DNA-extraction controls don't have to be sequenced if the DNA quality is assessed. Monitor all the template DNA and library quantity/quality parameters, sequencing quality parameters applied to the routinely tested samples and ensure that the sequencing analysis results match the true value. In the case of *E.coli* ATCC 25922:
      i. MLST allelic profile acquired during the run must correspond to known ST73 profile described for this strain.
      ii. 16S rRNA sequence must be identified as *Escherichia coli.*
      iii. No antibiotic resistance genes should be found with ResFinder analysis.

iv. Following virulence genes should be found using VirulenceFinder analysis with 100% ID and 100% query length coverage: *mchB, mchC, iss, mchF, mcmA, iha, sat, vat,* and *iroN*.

b. Include water/elution buffer as a no-template monthly control for detection of reagents contamination. Monitor DNA and library quantity/quality parameters, as well as sequencing quality parameters to ensure that they stay within the established thresholds.

3. Implement established quality thresholds as test rejection criteria.
4. Establish policies for tracking isolates throughout DNA isolation, library preparation, and data analysis:
    a. Develop a requisition form for the submitters.
    b. Database connecting received bacterial cultures to the DNA extracts and purified libraries (in the case if a repeat of the run is required), as well as sequencing run dates, analysis files and final reports.
    c. Implement templates for DNA dilutions and Indexing for better tracking of the samples. Use of Tracking form/worksheet is advisable.
    d. If possible, store the received bacterial cultures as the glycerol stocks.
5. Contamination prevention:
    a. It is a good practice to check low coverage (<15x) contigs which are ≥200 nt for the contamination using BLAST search and looking for DNA fragments belonging to other bacterial species.
    b. Perform irradiation of the biosafety cabinets/hoods used for DNA and library preparation with UV for 20-30 min after each time of use.
    c. Perform weekly cleaning of the pre- and post-PCR biosafety cabinets/laminar flow cabinets with their contents and mop lab floors using a 10% bleach solution. Monthly clean all lab working areas with 10% bleach.
    d. Perform line wash on MiSeq instrument with 0.01% sodium hypochlorite at least monthly.
    e. Good pipetting and sample handling practices to prevent carry-over should be followed.
    f. Separation of pre-PCR and post-PCR areas is beneficial. Ensure unidirectional sample flow.
    g. Index rotation schedule between the runs.
6. Establish semiannual proficiency testing for the laboratory and competency testing for the lab staff on an annual basis (semiannual in the first year from the training):
    a. It is recommended that the testing set (selected from previously characterized isolates) contains at least 4 isolates of the same species for phylogenetic analysis.
    b. Both Gram-negative and Gram-positive isolates should be included in the PT, or rotated periodically.
    c. Samples IDs should be blinded by the supervisor/personnel from outside of the laboratory prior to the testing, except for the species names (to allow appropriate DNA isolation method choices by the staff).
    d. One operator should start from DNA isolation and proceed through all the steps of library preparation, sequencing, and data analysis (unless the lab workflow is routinely divided among different staff members, then each person should be evaluated based on the procedures he/she performs).

e. All test samples should be assembled *de novo*. Species identification by 16S rRNA, MLST typing, ABR genes detection, or any other test done routinely by the lab should be performed for all samples.
f. Four or more samples belonging to the same species should be subjected to phylogenetic analysis.

7. Develop a documented quality assurance plan:
   a. SOP describing the testing procedure including sample receipt, DNA isolation, library preparation, operating sequencer, data analysis, and reporting. To supplement procedure SOP, develop worksheets covering all above-mentioned steps of WGS.
   b. Specific QC/QA SOPs, describing procedures for reagents preparation and QC, instruments maintenance and calibration, personnel training and competency assessment, proficiency testing, quarterly audit (traceback), instrument correlation (if multiple machines are used for the same assay), etc.
   c. Develop forms/logs/worksheets for reagents QC, instrument calibration/maintenance, storage temperature monitoring, software version control ,
   d. Have a monthly supervisory QC checklist. Implement corrective actions and follow up.
8. Sample and document retention policy needs to be developed.
9. Develop reporting language to be able to convey the WGS-based assay results to the public without the extensive knowledge of WGS- see Chapter 10 of this report for more details.
10. Perform re-validation of assay changes.

Regular and monthly QC practices are summarized in the figure below:

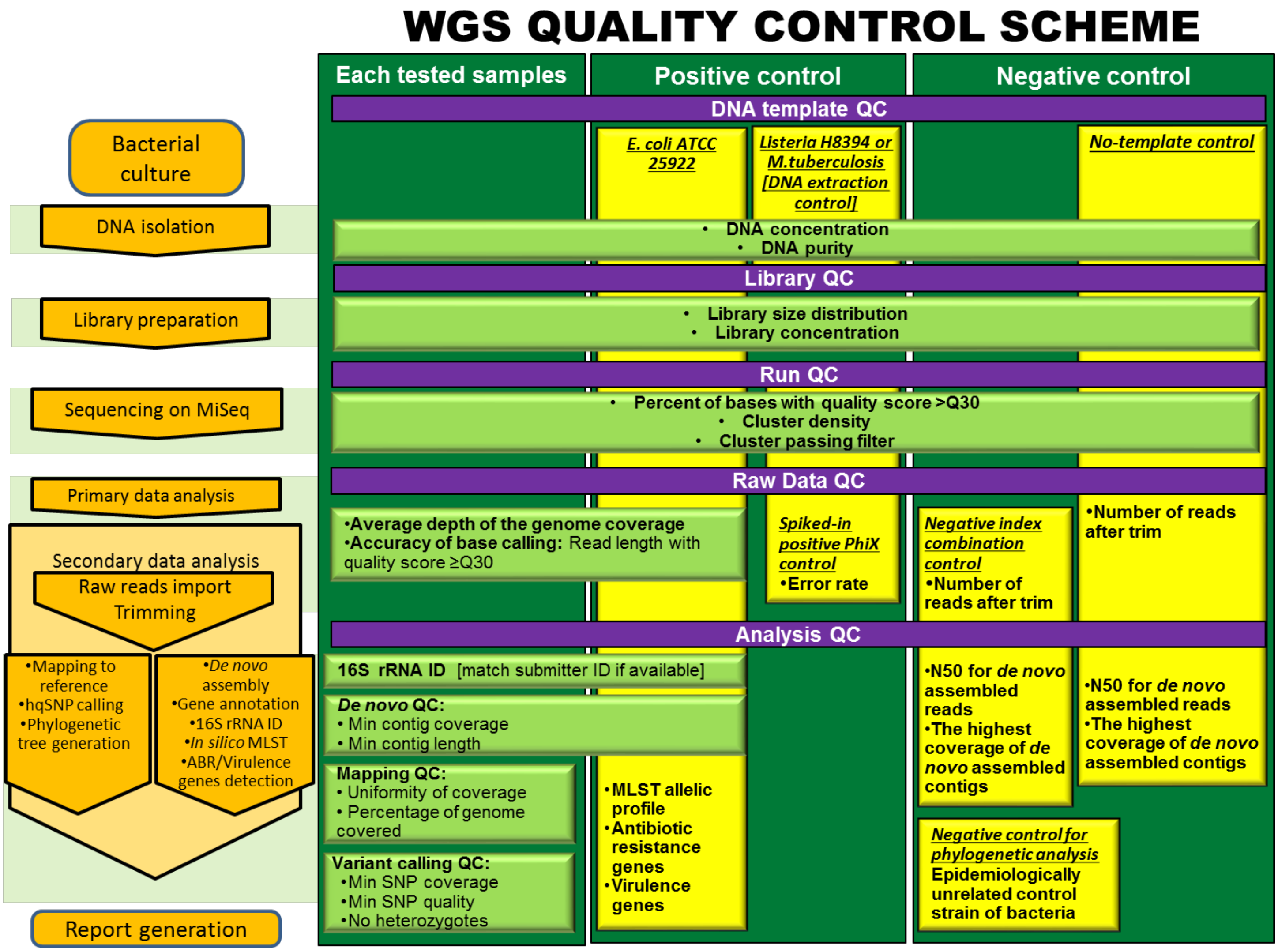

10. Results reporting

The following statement will accompany all WGS reports:

Disclaimer:

DISCLAIMER

The report

WGS results are provides based on a laboratory developed test (LDT) using Illumina MiSeq Sequencer and chemistry. The test has not been validated for clinical use and therefore, it is intended for investigational use only (IUO) or research use only (RUO). Additional investigation is necessary to establish epidemiological links.

Coverage and Gene Identification

The results represent ___*average depth of coverage X (min coverage X – max coverage X)*___ coverage of the bacterial genomes, which allows identification of genes and intergenic regions in over __ *% of genome coverage*____% of the __*species*____ genome. Any ambiguities in sequencing results should be resolved by Sanger sequencing.

High quality (hq) Single Nucleotide Polymorphisms (SNPs) Genotyping

Phylogenetic tree was generated by identification of hqSNPs in each isolate and genome wide comparison of hqSNPs between test isolates (___*tree building algorithm used*___). Individual __*species*___ isolates are represented as nodes on the phylogenetic tree. Group of isolates closely related to each other (connected by shorter branches) form a clade in phylogenetic tree, suggesting a possibility of epidemiological link between the isolates. The point where the branches split, called internal node, designates most recent common ancestor and potentially indicates common transmission event. The phylogenetic tree data is supplemented with the distance matrix table showing pairwise comparison of all isolates. Values in intersection show the number of SNPs differences between two isolates with smaller differences indicating close relatedness.

In silico **Multilocus Sequence Typing (MLST)**

In silico MLST results show genetic relatedness of the isolates based on sequence variations (alleles) in target housekeeping genes (MLST scheme for __*species/genus*___ via MLST 1.8 web tool at Center for Genomic Epidemiology (CGE)). Combination of known alleles of target genes allowed assignment of sequence type number, e.g. ST9. Overall, MLST provided significantly lower resolution among test isolates than WGS.

In silico **Antimicrobial Resistance Gene Detection**

The antimicrobial resistance genes were identified by database search of known antibiotic resistance determinants (ResFinder 2.1 web tool at CGE). This approach does not include chromosomal mutations conferring antibiotic resistance (like high-level Ciprofloxacin resistance, etc.). Phenotypic susceptibility tests are required to confirm antimicrobial resistance.

In silico **Detection of Virulence genes**

The virulence genes were identified by the database search for known virulence determinants (VirulenceFinder 1.4 web tool at CGE). Unknown or novel virulence element cannot be detected by this approach.

Data Release

The nucleotide sequence data from this analysis has not been released or deposited in any database.

Test results must be reported as presented in following form template:

Template form

Header: **Microbial Disease Laboratory / Core laboratory/ Whole Genome Sequencing report**

Note for lab personnel preparing the report: See instructions for filling the form in red color. Remove all records in red color before submitting the report. The information in

green is subject to change, substitute with correct value and change font to black before submitting the report.

The Whole Genome Sequencing of _# of isolates_ __*species*__ isolates was performed by MDL Core laboratory using Illumina sequencing chemistry, 300bp x 2 paired-end reads, on Illumina MiSeq sequencer. Phylogenetic analysis, 16S rRNA identification, *in silico* MLST typing, antibiotic resistance and virulence genes detection results are included in this report. Please find the report summary on page X.

Isolate Details

Use one of two following tables for isolate details recording depending on available metadata:
Isolate details table 1: Copy and paste this table multiple times if more than one isolate was analyzed

<table>
<tr><td colspan="4">MDL isolate ID number</td><td></td></tr>
<tr><td colspan="4">Patient's info</td><td>Description of submitted specimen</td></tr>
<tr><td colspan="2">Patient's ID:</td><td>Age:</td><td>Sex:</td><td>Date collected:</td></tr>
<tr><td colspan="4">Address:</td><td>Date submitted:</td></tr>
<tr><td colspan="4">Clinical condition or suspected disease:</td><td>Check source: ☐ Human ☐ Animal- species:_________________
☐ Other (specify):</td></tr>
<tr><td colspan="2">Date of onset:</td><td colspan="2">Physician's name:</td><td rowspan="2">Origin of specimen:
☐ Blood ☐ Serum ☐ Sputum ☐ CSF
☐ Throat ☐ Urine ☐ Feces ☐ Skin</td></tr>
<tr><td colspan="4">☐ Case ☐ Epidemic ☐ Sporadic ☐ Contact ☐ Carrier</td></tr>
<tr><td colspan="4" rowspan="5">Return report to:
Name:
Address:

ZIP code:</td><td>Tissue, type:</td></tr>
<tr><td>Pus, source:</td></tr>
<tr><td>Exudate, source:</td></tr>
<tr><td>Wound, location:</td></tr>
<tr><td rowspan="2">Other (specify):</td></tr>
<tr><td colspan="4">Antimicrobial agents: ☐ None</td></tr>
<tr><td>Types</td><td>Dosage</td><td>Date Begun</td><td>Date completed</td><td rowspan="4">Submitter's identification of organism:</td></tr>
<tr><td></td><td></td><td></td><td></td></tr>
<tr><td></td><td></td><td></td><td></td></tr>
<tr><td></td><td></td><td></td><td></td></tr>
<tr><td colspan="5">Brief but complete case history, therapy, outcome (print or type):</td></tr>
</table>

or Isolate details form 2: in the field heading "PFGE pattern" insert used restriction enzyme name, e.g. Xba I

	Submitter's information						MDL Core Lab information		
MDL Core lab Sample ID	Submitter	Submitter's ID number	County	Date collected	PFGE pattern (Xba I)	Outbreak code	Date received	Date of DNA isolation	Date of sequencing run

Species identity confirmation

☐ Species identity was confirmed

☐ Species identity is different from original identification. New ID ___________

List genes used for identification and percent identity:

☐ 16S rRNA _________

☐ cpn60 ___________

☐ Other:

____

Results of genotyping based on high quality (hq) Single Nucleotide Polymorphisms (SNPs)

Phylogenetic tree

Algorithm used for phylogenetic tree building: ☐ Neighbor Joining

☐ Maximum Likelihood

☐ Other. Specify: ____________

Tree goes here

Distance matrix (pairwise comparison):

Distance matrix goes here

More similar More different

Explanation: Value in intersection shows the number of SNPs difference between two isolates.

Comments:

***In silico* MLST results:**

**Isolate ID**	**MLST type (sequence type, ST)**

Comments:

Antibiotic resistance genes found:

Keep the background coloring for wells with %ID and Query each resistance/isolate combination as in ResFinder output.

**Type of antibiotic resistance**	**Aminoglycoside**						**Beta-Lactam**		**Sulphonamide**		**Tetracycline**		**Trimethoprim**	
**Resistance gene**	***aadA1***		***strA (aph(3'')-Ib)***		***strB (aph(6)-Id)***		***blaTEM-1B***		***sul2***		***tet(A)***		***dfrA1***	
**Gene accession number in NCBI**	JQ480156		AF321551		M96392		JF910132		GQ421466		AJ517790		X00926	
Isolate ID	% ID	Query/aligned length	% ID	Query/aligned length	% ID	Query/aligned length	% ID	Query/aligned length	% ID	Query/aligned length	% ID	Query/aligned length	% ID	Query/aligned length

1) The dark green color indicates a perfect match for a given gene. The %Identity is 100 and the sequence in the genome covers the entire length of the resistance gene in the database.
2) The grey color indicates a warning due to a non-perfect match, length of aligned sequence is shorter than resistance gene length, %ID can be 100% or less.
3) The light green color indicates a warning due to a non-perfect match, %ID < 100%, while length of aligned sequence = resistance gene length.

Comments:

Virulence genes found:

Keep the background coloring for wells with %ID and Query each resistance/isolate combination as in VirulenceFinder output.

	Virulence gene									
	celb		***gad***		***lpfA***		***senB***		***sigA***	
**Gene accession number in NCBI**	HF570109		U00096		AY646923		CP000038		CP000038	
Isolate ID	% ID	Query/aligned length	% ID	Query/aligned length	% ID	Query/aligned length	% ID	Query/aligned length	% ID	Query/aligned length

Virulence factors encoded by the present genes:

celb - Endonuclease colicin E2
gad - Glutamate decarboxylase
lpfA - Long polar fimbriae
senB - Plasmid-encoded enterotoxin
sigA- Shigella IgA-like protease homologue

1) The dark green color indicates a perfect match for a given gene. The %Identity is 100 and the sequence in the genome covers the entire length of the virulence gene in the database.
2) The grey color indicates a warning due to a non-perfect match, length of aligned sequence is shorter than virulence gene length, %ID can be 100% or less.
3) The light green color indicates a warning due to a non-perfect match, %ID < 100%, while length of aligned sequence = virulence gene length.

Comments:

Summary

1.
2.

References

Date of analysis __________

Report status: ☐ Preliminary

☐ Final

Reported by   Date_________

Supervisor   Date_________

DISCLAIMER

The report

WGS results are provides based on a laboratory developed test (LDT) using Illumina MiSeq Sequencer and chemistry. The test has not been validated for clinical use and therefore, it is intended for investigational use only (IUO) or research use only (RUO). Additional investigation is necessary to establish epidemiological links.

Coverage and Gene Identification

The results represent ___*average depth of coverage X (min coverage X – max coverage X)*___ coverage of the bacterial genomes, which allows identification of genes and intergenic regions in over __ *% of genome coverage*____% of the __*species*____ genome. Any ambiguities in sequencing results should be resolved by Sanger sequencing.

High quality (hq) Single Nucleotide Polymorphisms (SNPs) Genotyping

Phylogenetic tree was generated by identification of hqSNPs in each isolate and genome wide comparison of hqSNPs between test isolates (___*tree building algorithm used*___). Individual __*species*___ isolates are represented as nodes on the phylogenetic tree. Group of isolates closely related to each other (connected by shorter branches) form a clade in phylogenetic tree, suggesting a <u>possibility</u> of epidemiological link between the isolates. The point where the branches split, called internal node, designates most recent common ancestor and potentially indicates common transmission event. The phylogenetic tree data is supplemented with the distance matrix table showing pairwise comparison of all isolates. Values in intersection show the number of SNPs differences between two isolates with smaller differences indicating close relatedness.

***In silico* Multilocus Sequence Typing (MLST)**

In silico MLST results show genetic relatedness of the isolates based on sequence variations (alleles) in target housekeeping genes (MLST scheme for __*species/genus*___ via MLST 1.8 web tool at Center for Genomic Epidemiology (CGE)). Combination of known alleles of target genes allowed assignment of sequence type number, e.g. ST9. Overall, MLST provided significantly lower resolution among test isolates than WGS.

***In silico* Antimicrobial Resistance Gene Detection**

The antimicrobial resistance genes were identified by database search of known antibiotic resistance determinants (ResFinder 2.1 web tool at CGE). This approach does not include chromosomal mutations conferring antibiotic resistance (like high-level Ciprofloxacin resistance, etc.). Phenotypic susceptibility tests are required to confirm antimicrobial resistance.

In silico **Detection of Virulence genes**

The virulence genes were identified by the database search for known virulence determinants (VirulenceFinder 1.4 web tool at CGE). Unknown or novel virulence element cannot be detected by this approach.

Data Release

The nucleotide sequence data from this analysis has not been released or deposited in any database.

Example of the report

Header: **Microbial Disease Laboratory / Core laboratory/ Whole Genome Sequencing report**

The Whole Genome Sequencing of 54 *Shigella sonnei* isolates was performed by MDL Core laboratory using Illumina sequencing chemistry, 300bp x 2 paired-end reads, on Illumina MiSeq sequencer. Phylogenetic analysis, 16S rRNA identification, and *in silico* MLST typing results are included in this report. Please find the report summary on page 5.

Isolate Details

	MDL Core lab Sample ID	Submitter's information						MDL Core Lab information		
		Submitter	Submitter's ID number	County	Date collected	Xba1 pattern	Outbreak code	Date received	Date of DNA isolation	Date of sequencing run
1	C157	X Regional	M15X0xxxx	Santa Clara	10/17/2015	J16X01.0728	1510CASCJ16-1	11/10/2015	11/10/2015	11/13/2015
2	C158	X Regional	M15X0yyyy	Santa Clara	10/17/2015	J16X01.1244	1510CASCJ16-1	11/10/2015	11/10/2015	11/13/2015
3	C163	Z Co PHL	M15X0zzzz	Alameda	10/18/2015	J16X01.0283	1510CASCJ16-1	11/10/2015	11/10/2015	11/13/2015
4	…									
5										
…										
54										

Comments: PFGE profiles for samples submitted by the X Regional Laboratory were generated by MDL Foodborne & Waterborne Disease Detection Unit. For samples from Z Co PHL the PFGE pattern information was provided by the submitter.

Species identity confirmation

☒ Species identity was confirmed

☐ Species identity is different from original identification. New ID __________

List genes used for identification and percent identity:

☒ 16S rRNA 100% ID with *Escherichia coli*/*Shigella* spp ___

☐ cpn60 __________

☐ Other:

____

Comments: *Escherichia coli* and *Shigella* species cannot be distinguished based on 16S rRNA sequence[1].

Results of genotyping based on high quality (hq) Single Nucleotide Polymorphisms (SNPs)

Phylogenetic tree

Algorithm used for phylogenetic tree building: ☐ Neighbor Joining

☒ Maximum Likelihood

☐ Other. Specify: ____________

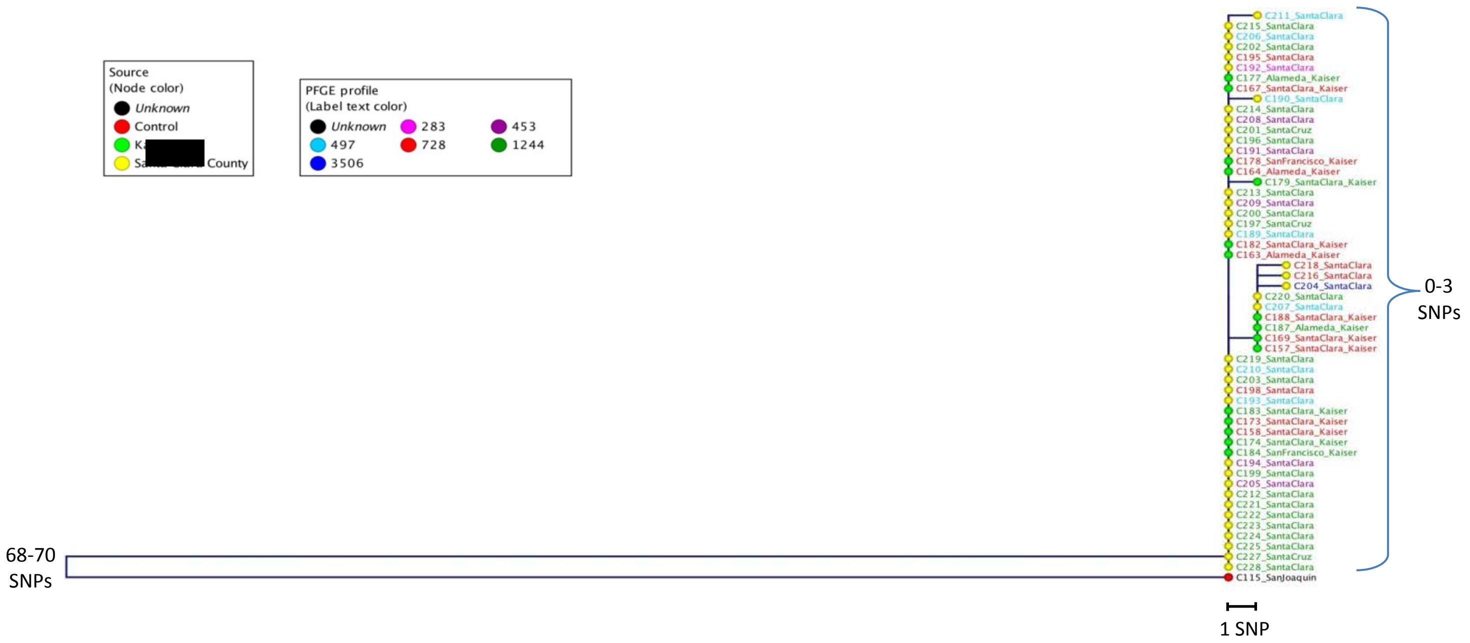

Distance matrix (pairwise comparison):

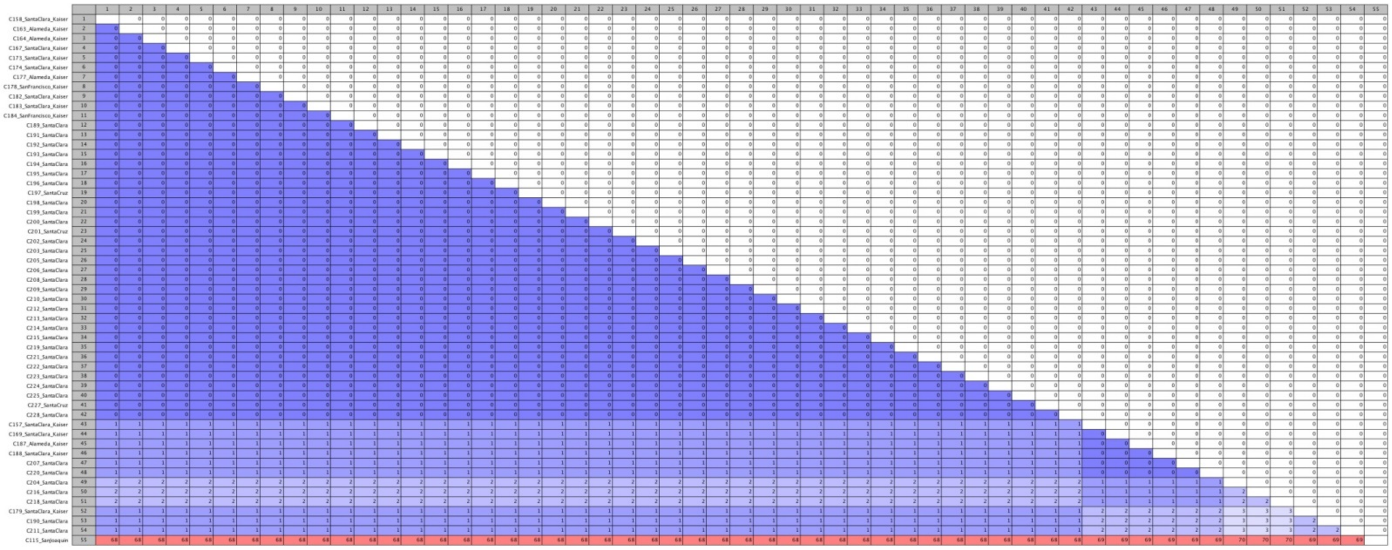

More similar — More different

Explanation: Value in intersection shows the number of SNPs difference between two isolates.

Comments: The isolate C115 is included in the analysis for comparison; it represents a recent *Shigella sonnei* strain with no established epidemiological connection to the tested samples.

***In silico* MLST results:**

**Isolate ID**	**MLST type (sequence type, ST)**
C157	ST-152
C158	ST-152
C163	ST-152
…	…

Antibiotic resistance genes found:

Type of antibiotic resistance	Aminoglycoside						Beta-Lactam		Sulphonamide		Tetracycline		Trimethoprim	
Resistance gene	*aadA1*		*strA (aph(3'')-Ib)*		*strB (aph(6)-Id)*		*blaTEM-1B*		*sul2*		*tet(A)*		*dfrA1*	
Gene accession number in NCBI	JQ480156		AF321551		M96392		JF910132		GQ421466		AJ517790		X00926	
Isolate ID	% ID	Query/aligned length	% ID	Query/aligned length	% ID	Query/aligned length	% ID	Query/aligned length	% ID	Query/aligned length	% ID	Query/aligned length	% ID	Query/aligned length
C157	100	789 / 789	100	804 / 804	100	837 / 837	100	861 / 861			100	1172 / 1200	100	474 / 474
C158	100	789 / 789	100	804 / 804	100	765 / 837	100	861 / 861	100	816 / 816	100	1172 / 1200	100	474 / 474
C163	100	789 / 789	100	804 / 804	100	837 / 837			100	816 / 816	100	1172 / 1200	100	474 / 474
…														

1) The dark green color indicates a perfect match for a given gene. The %Identity is 100 and the sequence in the genome covers the entire length of the resistance gene in the database.
2) The grey color indicates a warning due to a non-perfect match, length of aligned sequence is shorter than resistance gene length, %ID can be 100% or less.
3) The light green color indicates a warning due to a non-perfect match, %ID < 100%, while length of aligned sequence = resistance gene length.

Comments:

Virulence genes found:

Virulence gene	celb		gad		lpfA		senB		sigA	
Gene accession number in NCBI	HF570109		U00096		AY646923		CP000038		CP000038	
Isolate ID	% ID	Query/aligned length	% ID	Query/aligned length	% ID	Query/aligned length	% ID	Query/aligned length	% ID	Query/aligned length
C157	100	144 / 144	99.83	1401 / 1158	100	573 / 573	100	1176 / 1176	100	3858 / 3858
C158	100	144 / 144			100	573 / 573	100	1176 / 1176	100	3858 / 3858
C163	100	144 / 144	99.65	1401 / 1158	100	573 / 573	100	1176 / 1176	100	3858 / 3858
…										

Virulence factors encoded by the present genes:

celb - Endonuclease colicin E2
gad - Glutamate decarboxylase
lpfA - Long polar fimbriae
senB - Plasmid-encoded enterotoxin
sigA- Shigella IgA-like protease homologue

Summary

1. All *Shigella sonnei* isolates from XXX County were assigned to a single cluster based on hqSNP calling. These isolates differed only by 0-3 SNPs. The control isolate is unrelated to the described cluster, as expected, and differs from closest member of the cluster by 68 SNPs.
2. According to MLST typing, all isolates belong to the same sequence type ST-152.
3. All isolates possess genes known to mediate aminoglycoside (streptomycin) resistance. Ampicillin resistance-conferring gene *blaTEM-1* was found in genome sequences of all tested isolates. Combination of genes *sul2* and *dfrA1*, connected to trimethoprim/sulfamethoxazole resistance, was found in all isolates. A *tetA* gene is truncated in all the isolates, and is most likely not expressed.
4. Tested isolates encode characteristic for *Shigella sonnei* virulence factors. No Shiga-toxin genes were found.

References

1. CLSI 2008. Interpretive criteria for identification of bacteria and fungi by DNA target sequencing; approved guideline, CLSI document MM18-A. Clinical and Laboratory Standards Institute, Wayne, PA

Date of analysis ______12/22/2015

Report status: ☐ Preliminary

☒ Final

Reported by …   Date____12/22/2015

Supervisor …   Date____12/23/2015

DISCLAIMER

The report

WGS results were generated based upon a laboratory developed test (LDT) using Illumina MiSeq Sequencer and chemistry. The test has not been validated for clinical use and therefore, it is intended for investigational use only (IUO) or research use only (RUO). Additional investigation is necessary to establish epidemiological links.

Coverage and Gene Identification

The results represent 56.37X (33.29 - 92.18X) coverage of the bacterial genomes, which allows identification of genes and intergenic regions in over 93% of the *Shigella sonnei* genome. Any ambiguities in sequencing results are to be resolved by Sanger sequencing.

High quality (hq) Single Nucleotide Polymorphisms (SNPs) Genotyping

Phylogenetic tree was generated by identification of hqSNPs in each isolate and genome wide comparison of hqSNPs between test isolates Maximum Likelihood tree building algorithm). Individual *Shigella sonnei* isolates are represented as nodes on the phylogenetic tree. Group of isolates closely related to each other (connected by shorter branches) form a clade in phylogenetic tree, suggesting the possibility of epidemiological link between the isolates. The point where the branches split, called internal node, designates most recent common ancestor and potentially indicates common transmission event. The phylogenetic tree data is supplemented with the distance matrix table showing pairwise comparison of all isolates. Values in intersection show the number of SNPs differences between two isolates with smaller differences indicating close relatedness.

***In silico* Multilocus Sequence Typing (MLST)**

In silico MLST results show genetic relatedness of the isolates based on sequence variations (alleles) in target housekeeping genes (MLST scheme for *Escherichia coli/Shigella* via MLST 1.8 web tool at Center for Genomic Epidemiology (CGE)). Combination of known alleles of target genes allowed assignment of sequence type number, e.g. ST9. Overall, MLST provided significantly lower resolution among test isolates than WGS.

***In silico* Antimicrobial Resistance Gene Detection**

The antimicrobial resistance genes were identified by database search of known antibiotic resistance determinants (ResFinder 2.1 web tool at CGE). This approach does not include chromosomal mutations conferring antibiotic resistance (like high-level Ciprofloxacin resistance, etc.). Phenotypic susceptibility tests are required to confirm antimicrobial resistance.

***In silico* Detection of Virulence genes**

The virulence genes were identified by the database search for known virulence determinants (VirulenceFinder 1.4 web tool at CGE). Unknown or novel virulence element cannot be detected by this approach.

Data Release

The nucleotide sequence data from this analysis has not been released or deposited in any database.

References

1. Gilchrist CA, Turner SD, Riley MF, Petri WA, Jr., Hewlett EL (2015) Whole-genome sequencing in outbreak analysis. Clin Microbiol Rev 28: 541-563.
2. Rohde H, Qin J, Cui Y, Li D, Loman NJ, et al. (2011) Open-source genomic analysis of Shiga-toxin-producing E. coli O104:H4. N Engl J Med 365: 718-724.
3. Snitkin ES, Zelazny AM, Thomas PJ, Stock F, Henderson DK, et al. (2012) Tracking a hospital outbreak of carbapenem-resistant Klebsiella pneumoniae with whole-genome sequencing. Sci Transl Med 4: 148ra116.
4. Sintchenko V, Holmes EC (2015) The role of pathogen genomics in assessing disease transmission. BMJ 350: h1314.
5. Leopold SR, Goering RV, Witten A, Harmsen D, Mellmann A (2014) Bacterial whole-genome sequencing revisited: portable, scalable, and standardized analysis for typing and detection of virulence and antibiotic resistance genes. J Clin Microbiol 52: 2365-2370.
6. Mellmann A, Harmsen D, Cummings CA, Zentz EB, Leopold SR, et al. (2011) Prospective genomic characterization of the German enterohemorrhagic Escherichia coli O104:H4 outbreak by rapid next generation sequencing technology. PLoS One 6: e22751.
7. Snyder LA, Loman NJ, Faraj LA, Levi K, Weinstock G, et al. (2013) Epidemiological investigation of Pseudomonas aeruginosa isolates from a six-year-long hospital outbreak using high-throughput whole genome sequencing. Euro Surveill 18.
8. Leekitcharoenphon P, Nielsen EM, Kaas RS, Lund O, Aarestrup FM (2014) Evaluation of whole genome sequencing for outbreak detection of Salmonella enterica. PLoS One 9: e87991.
9. Salipante SJ, SenGupta DJ, Cummings LA, Land TA, Hoogestraat DR, et al. (2015) Application of whole-genome sequencing for bacterial strain typing in molecular epidemiology. J Clin Microbiol 53: 1072-1079.
10. Reuter S, Ellington MJ, Cartwright EJ, Koser CU, Torok ME, et al. (2013) Rapid bacterial whole-genome sequencing to enhance diagnostic and public health microbiology. JAMA Intern Med 173: 1397-1404.
11. Koser CU, Holden MT, Ellington MJ, Cartwright EJ, Brown NM, et al. (2012) Rapid whole-genome sequencing for investigation of a neonatal MRSA outbreak. N Engl J Med 366: 2267-2275.
12. Harris SR, Cartwright EJ, Torok ME, Holden MT, Brown NM, et al. (2013) Whole-genome sequencing for analysis of an outbreak of meticillin-resistant Staphylococcus aureus: a descriptive study. Lancet Infect Dis 13: 130-136.
13. Zankari E, Hasman H, Kaas RS, Seyfarth AM, Agerso Y, et al. (2013) Genotyping using whole-genome sequencing is a realistic alternative to surveillance based on phenotypic antimicrobial susceptibility testing. J Antimicrob Chemother 68: 771-777.
14. Stoesser N, Batty EM, Eyre DW, Morgan M, Wyllie DH, et al. (2013) Predicting antimicrobial susceptibilities for Escherichia coli and Klebsiella pneumoniae isolates using whole genomic sequence data. J Antimicrob Chemother 68: 2234-2244.
15. Gordon NC, Price JR, Cole K, Everitt R, Morgan M, et al. (2014) Prediction of Staphylococcus aureus antimicrobial resistance by whole-genome sequencing. J Clin Microbiol 52: 1182-1191.
16. Joensen KG, Scheutz F, Lund O, Hasman H, Kaas RS, et al. (2014) Real-time whole-genome sequencing for routine typing, surveillance, and outbreak detection of verotoxigenic Escherichia coli. J Clin Microbiol 52: 1501-1510.
17. Ranjan R, Rani A, Metwally A, McGee HS, Perkins DL (2015) Analysis of the microbiome: Advantages of whole genome shotgun versus 16S amplicon sequencing. Biochem Biophys Res Commun.
18. Lazarevic V, Whiteson K, Gaia N, Gizard Y, Hernandez D, et al. (2012) Analysis of the salivary microbiome using culture-independent techniques. J Clin Bioinforma 2: 4.

19. Li H, Handsaker B, Wysoker A, Fennell T, Ruan J, et al. (2009) The Sequence Alignment/Map format and SAMtools. Bioinformatics 25: 2078-2079.
20. CLIA regulations 42CFR § 493.1253.
21. Gargis AS, Kalman L, Berry MW, Bick DP, Dimmock DP, et al. (2012) Assuring the quality of next-generation sequencing in clinical laboratory practice. Nat Biotechnol 30: 1033-1036.
22. CLSI (2014) Nucleic Acid Sequencing Methods in Diagnostic Laboratory Medicine: Approved Guideline- 2d edition. MM09-A2.
23. Moran-Gilad J, Sintchenko V, Pedersen SK, Wolfgang WJ, Pettengill J, et al. (2015) Proficiency testing for bacterial whole genome sequencing: an end-user survey of current capabilities, requirements and priorities. BMC Infect Dis 15: 174.
24. Chosewood LC, Wilson DE, Centers for Disease Control and Prevention (U.S.), National Institutes of Health (U.S.) (2009) Biosafety in microbiological and biomedical laboratories. Washington, D.C.: U.S. Dept. of Health and Human Services, Public Health Service, Centers for Disease Control and Prevention, National Institutes of Health. xxii, 415 p. p.
25. Sims D, Sudbery I, Ilott NE, Heger A, Ponting CP (2014) Sequencing depth and coverage: key considerations in genomic analyses. Nat Rev Genet 15: 121-132.
26. MiSeq Specifications.
27. Schirmer M, Ijaz UZ, D'Amore R, Hall N, Sloan WT, et al. (2015) Insight into biases and sequencing errors for amplicon sequencing with the Illumina MiSeq platform. Nucleic Acids Res 43: e37.
28. Rihtman B, Meaden S, Clokie MR, Koskella B, Millard AD (2016) Assessing Illumina technology for the high-throughput sequencing of bacteriophage genomes. PeerJ 4: e2055.
29. Kwong JC, McCallum N, Sintchenko V, Howden BP (2015) Whole genome sequencing in clinical and public health microbiology. Pathology 47: 199-210.
30. Carrillo CD, Kruczkiewicz P, Mutschall S, Tudor A, Clark C, et al. (2012) A framework for assessing the concordance of molecular typing methods and the true strain phylogeny of Campylobacter jejuni and C. coli using draft genome sequence data. Front Cell Infect Microbiol 2: 57.
31. Gargis AS, Kalman L, Bick DP, da Silva C, Dimmock DP, et al. (2015) Good laboratory practice for clinical next-generation sequencing informatics pipelines. Nat Biotechnol 33: 689-693.
32. CLSI (2007) Molecular Methods for Bacterial Strain Typing; Approved Guideline, MM11-A.

Appendix 1. Reference and Validation tree comparison method

Comparison with NCBI reference tree

Validation sequences generated by the Core lab were clustered together with additional comparison sequences of the same species from NCBI database. Total number of clustered samples =5. Reference sequences available from NCBI for validation samples were clustered in an analogous way with comparison sequences. Tree comprised of only NCBI sequences ("NCBI tree") matched tree comprised of combination of validation Core lab sequences and comparison NCBI sequences ("Core tree") in all cases, meaning that sequences which were generated by Core lab for the validation strains were concordant with NCBI reference sequences for the same strains.

In order to cluster NCBI reference sequences, full genomes were downloaded and converted into the paired reads format. Following command-line tool was used for mock reads generation:

```
wgsim -N1000000 -r 0 -R 0 -X 0 -e 0 NCBI_sequence.fasta
Mock_reads_NCBI_sequences.R1.fastq Mock_reads_NCBI_sequences.R2.fastq
```

Afterwards NCBI mock reads and Core lab generated reads were processed in identical ways as described in SOP.

Comparison with CDC reference tree

Similarly, strains previously sequenced by CDC were used for the tree comparison. Clustering of sequences generated by CDC was compared with clustering of Core lab validation sequences for the same strains. Tree generated from CDC sequences ("CDC tree") matched tree comprised of validation sequences generated by the Core lab ("Core tree").

CDC sequences were obtained as raw reads from SRA database and processed alongside with Core lab sequences.

Comparison trees were built for 2 species of Enterobacteriaceae family, 2 species of Gram-positive bacteria, and one species on Non-fermenting bacteria. Below see the list of sequenced isolates and references used for comparison:

Escherichia coli

Core lab ID	MDL ID	Species	NCBI Strain	NCBI Acc#	Comments
C1	O157:H7 CDC EDL 933	*Escherichia coli*	O157:H7 CDC EDL 933	NZ_CP008957.1	
C3	ATCC 8739	*Escherichia coli*	ATCC 8739	NC_010468.1	
C55	ATCC 25922	*Escherichia coli*	ATCC 25922	NZ_CP009072.1	Reference for the tree
N/A	N/A	*Escherichia coli*	K-12 substr. MG1655	NC_000913.3	
N/A	N/A	*Escherichia coli*	O157:H7 str. Sakai	NC_002695.1	

Salmonella enterica

Core lab ID	MDL ID	Species	NCBI Strain	NCBI Acc#	Comments
C73	M10X01956	*Salmonella* Enteritidis	M10X01956/CDC_2010K-1543	SRR518749	SRA
C74	M14X00933	*Salmonella* Infantis	M14X00933/2014K-0434	SRR1616809	SRA
C75	M14X04729	*Salmonella* Adelaide	M14X04729/2014K-0941	SRR1686419	SRA
C76	M12X03253	*Salmonella* Worthington	M12X03253/2012K-1219	SRR1614868	SRA
C77	M14X04723	Salmonella Saintpaul	M14X04723/2014K-0875	SRR1640105	SRA
N/A	N/A	*Salmonella enterica* ser Typhimurium	14028S	NC_016856	Reference for the tree

Staphylococcus aureus

Core lab ID	MDL ID	Species	NCBI Strain	NCBI Acc#	Comments
C5	ATCC 25923	*Staphylococcus aureus*	ATCC 25923	NZ_CP009361	Reference for the tree
N/A	N/A	*Staphylococcus aureus*	RF122	NC_007622	
N/A	N/A	*Staphylococcus aureus*	NCTC 8325	NC_007795	
N/A	N/A	*Staphylococcus aureus*	Mu3	NC_009782	
N/A	N/A	*Staphylococcus aureus*	ST398	NC_017333	

Enterococcus faecalis

Core lab ID	MDL ID	Species	NCBI Strain	NCBI Acc#	Comments
C46	ATCC 29212	*Enterococcus faecalis*	ATCC 29212	NZ_CP008816	Reference for the tree
N/A	N/A	*Enterococcus faecalis*	D32	NC_018221	
N/A	N/A	*Enterococcus faecalis*	Symbioflor 1	NC_019770	
N/A	N/A	*Enterococcus faecalis*	DENG1	NZ_CP004081	
N/A	N/A	*Enterococcus faecalis*	V583	NC_004668	

Stenotrophomonas maltophilia

Core lab ID	MDL ID	Species	NCBI Strain	NCBI Acc#	Comments
C51	ATCC 13637	*Stenotrophomonas maltophilia*	ATCC 13637	NZ_CP008838	Reference for the tree
N/A	N/A	*Stenotrophomonas maltophilia*	R551-3	NC_011071	
N/A	N/A	*Stenotrophomonas maltophilia*	JV3	NC_015947	
N/A	N/A	*Stenotrophomonas maltophilia*	D457	NC_017671	
N/A	N/A	*Stenotrophomonas maltophilia*	K279a	NC_010943	

Green color highlights validation strains sequenced by Core lab and possessing matching reference sequence in Genome NCBI or SRA databases.

Analysis protocol

SNP alignments for NCBI tree and Core tree were imported into Geneious software:

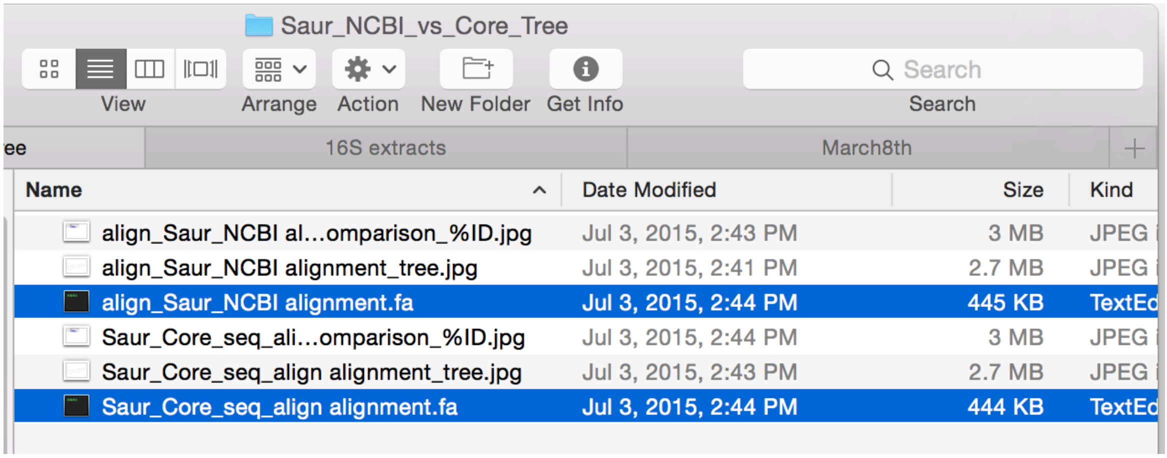

Import as Alignment:

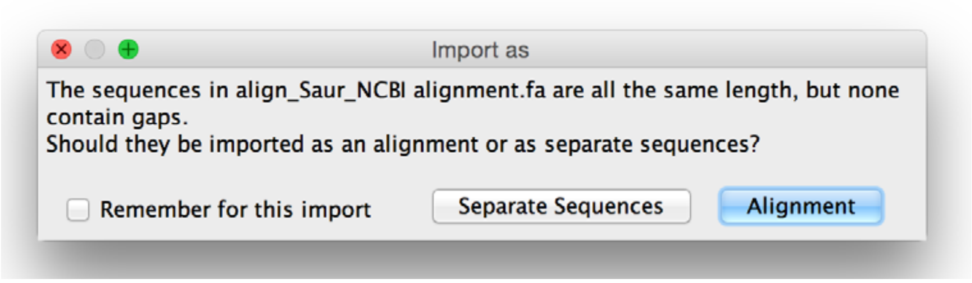

Select one alignment at a time and Build a tree:

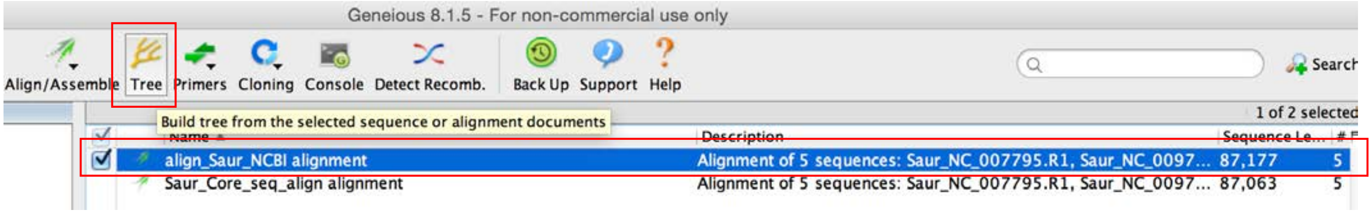

Used parameters:

(pick the same outgroup for both NCBI and Core tree)

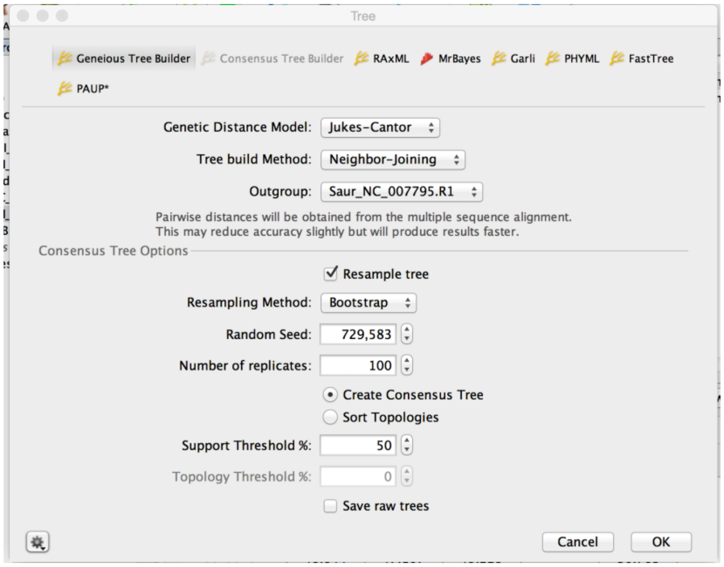

Export tree in newick format:

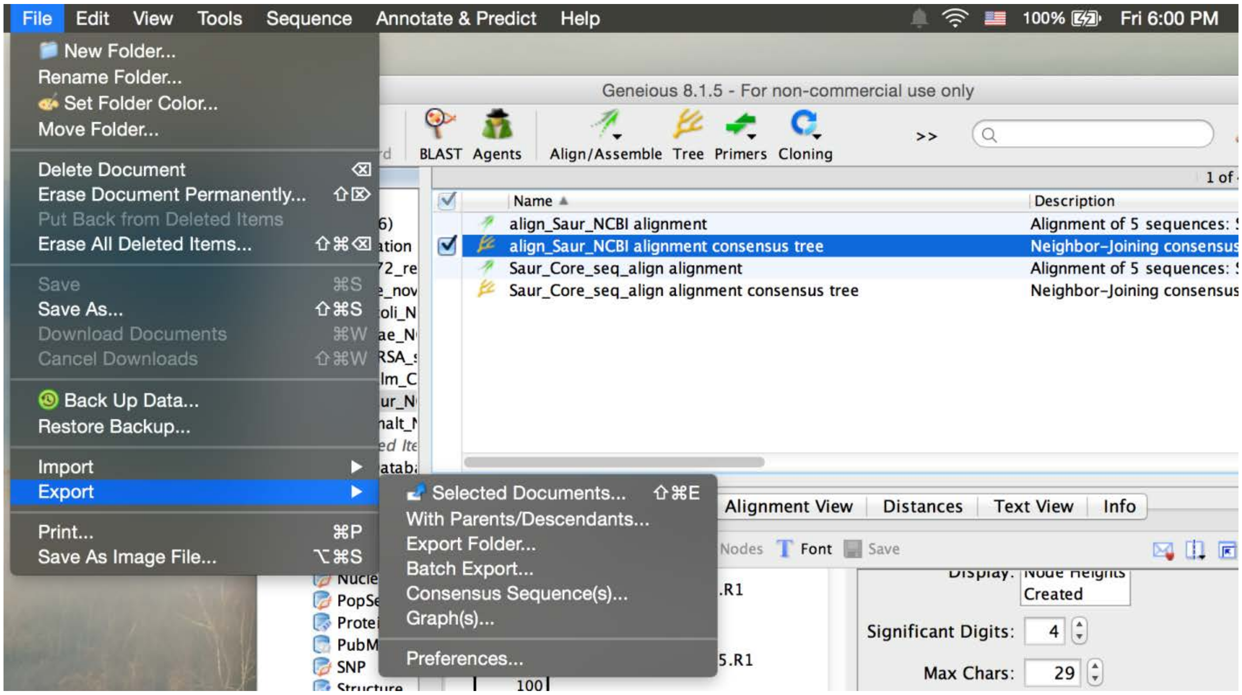

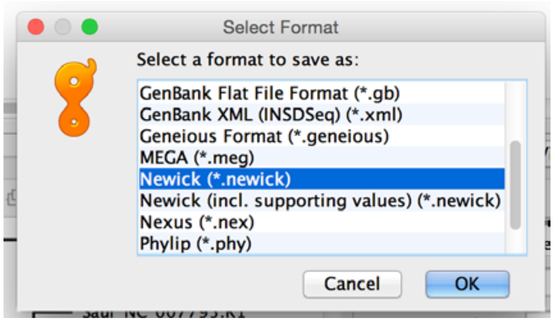

Open newick file with Text editor:

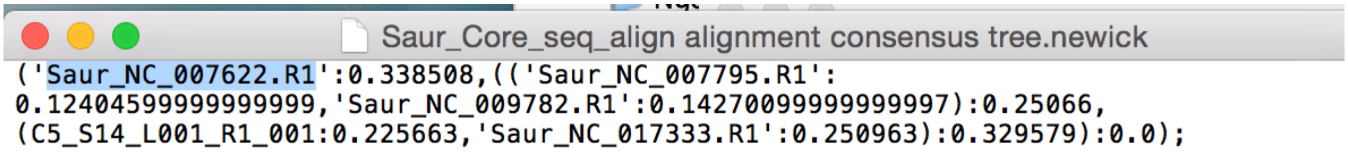

Copy newick record from both NCBI and Core tree:

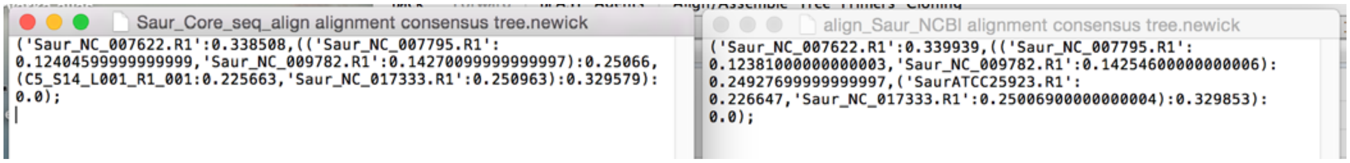

Substitute names of the same strains in NCBI and Core trees with generic name.

Example:

```
'Saur_NC_007622.R1' = ‘Saur_sample1'
'Saur_NC_007795.R1' = 'Saur_sample2’
'Saur_NC_009782.R1' = 'Saur_sample3’
C5_S14_L001_R1_001 or 'SaurATCC25923.R1' = 'Saur_sample4’
'Saur_NC_017333.R1' = 'Saur_sample5’
```

```
Core seq:
('Saur_NC_007622.R1':0.338508,(('Saur_NC_007795.R1':0.12404599999999999,'Saur_N
C_009782.R1':0.14270099999999997):0.25066,(C5_S14_L001_R1_001:0.225663,'Saur_NC
_017333.R1':0.250963):0.329579):0.0);
```

```
Substituted names for Core seq:
('Saur_sample1':0.338508,(('Saur_sample2’:0.12404599999999999,'Saur_sample3’:0.
14270099999999997):0.25066,('Saur_sample4’:0.225663,'Saur_sample5’:0.250963):0.
329579):0.0);
```

```
NCBI seq:
('Saur_NC_007622.R1':0.339939,(('Saur_NC_007795.R1':0.12381000000000003,'Saur_N
C_009782.R1':0.14254600000000006):0.24927699999999997,('SaurATCC25923.R1':0.226
647,'Saur_NC_017333.R1':0.25006900000000004):0.329853):0.0);

Substituted names for NCBI seq:
('Saur_sample1':0.339939,(('Saur_sample2':0.12381000000000003,'Saur_sample3':0.
14254600000000006):0.24927699999999997,('Saur_sample4':0.226647,'Saur_sample5':
0.25006900000000004):0.329853):0.0);
```

Modify and save files in newick format

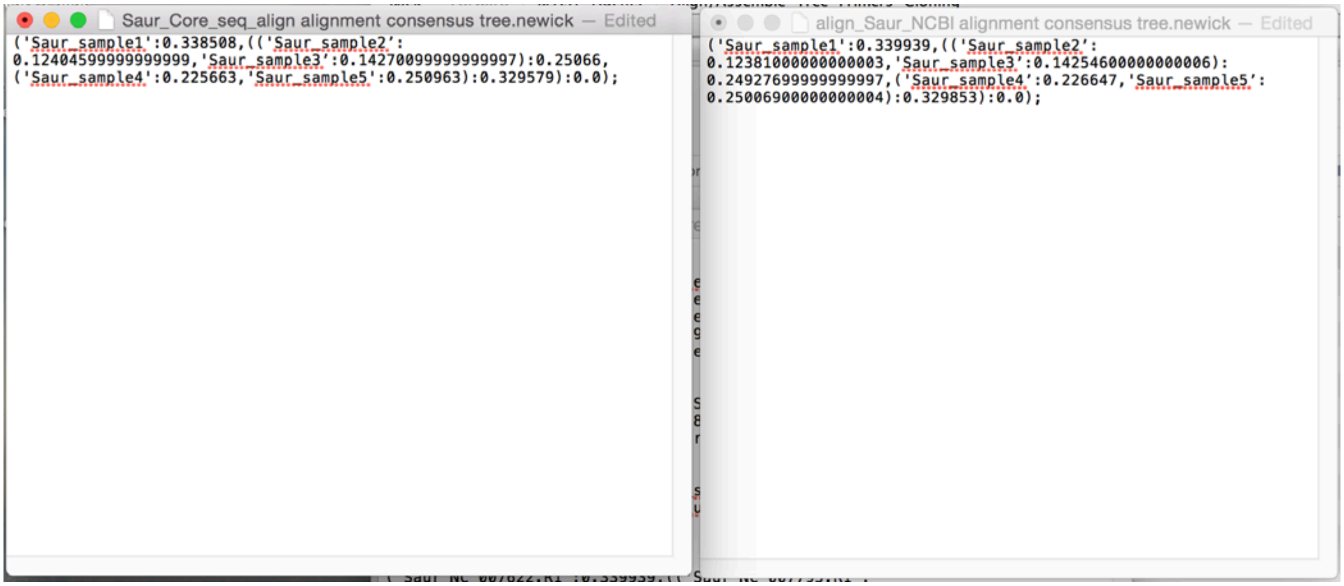

Open Compare2Trees app using command line:

Go to the directory with Compare2Trees app

```
cd ~/Compare2Trees
```

Type command to open the software:

```
java -classpath PhyloCore.jar:parallelcolt-0.9.4.jar
treecomparison.Compare2Trees
```

Click button "New tree.."

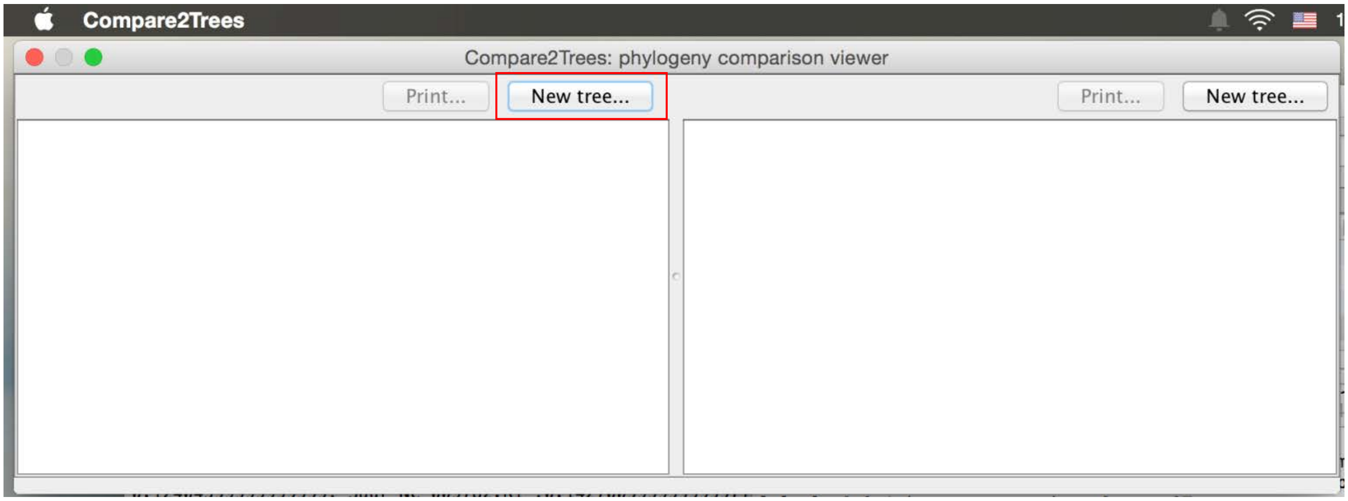

Open Core tree in newick format:

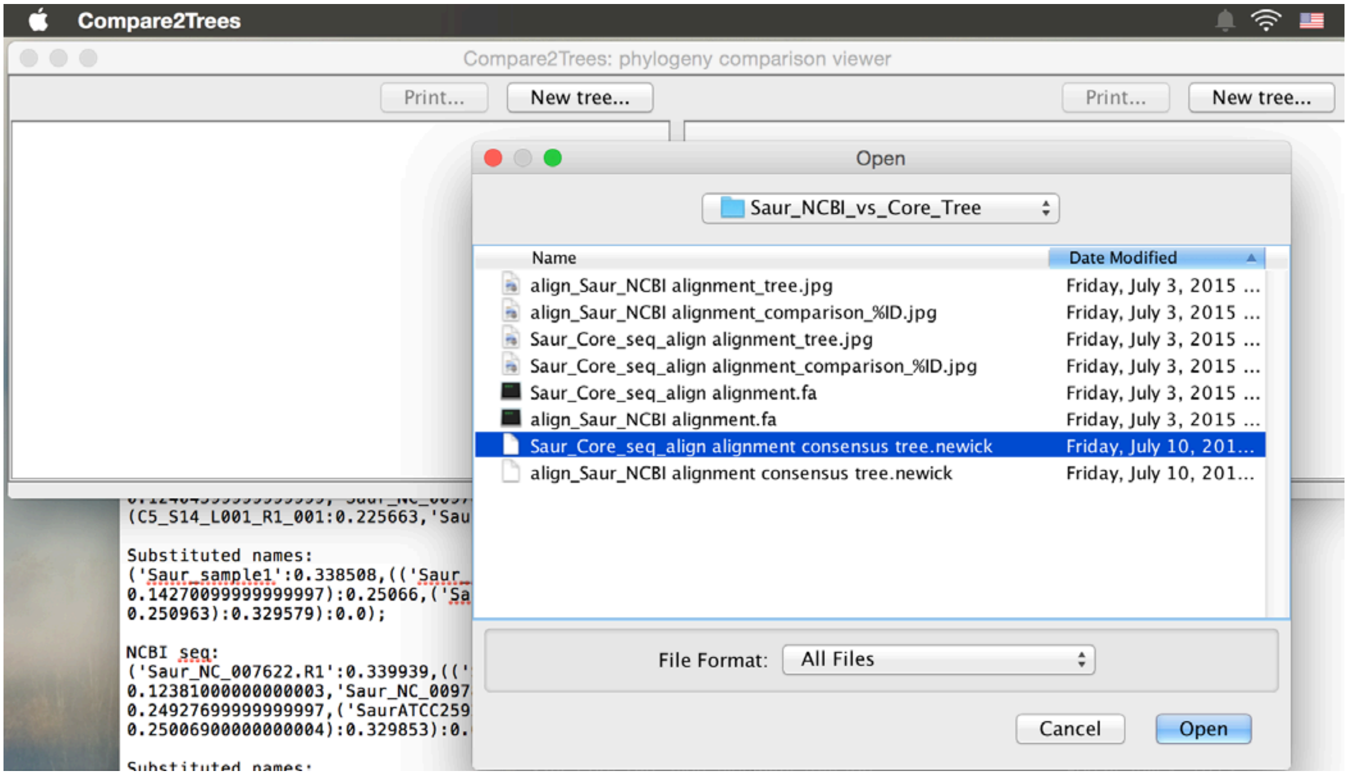

Open NCBI tree in newick format:

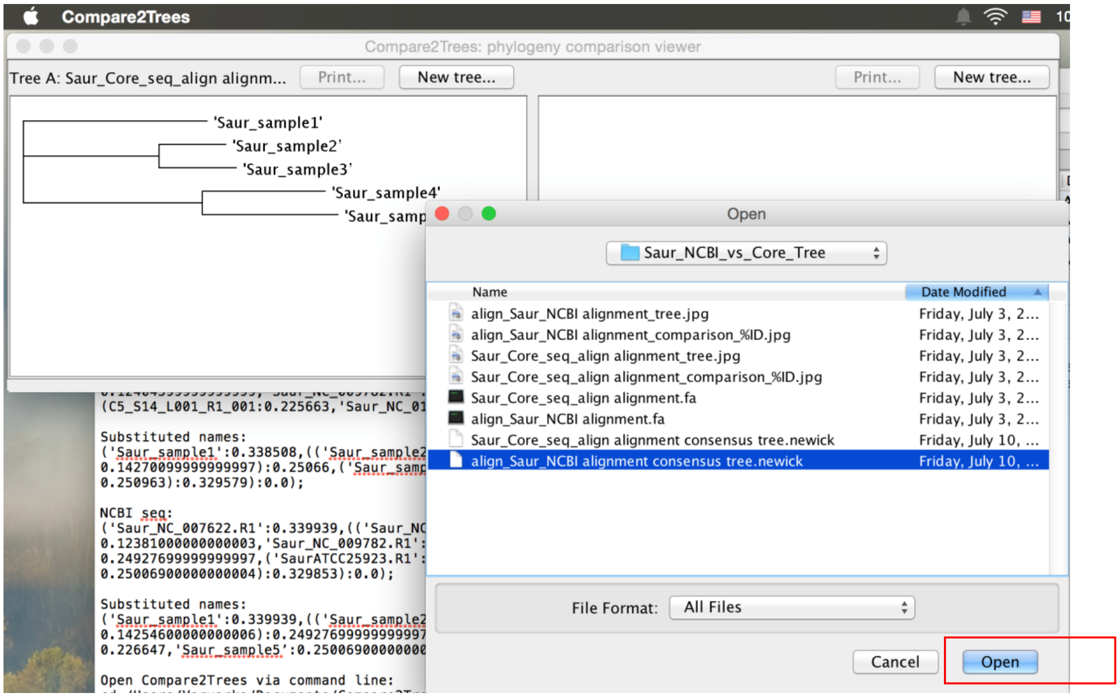

Comparison view:

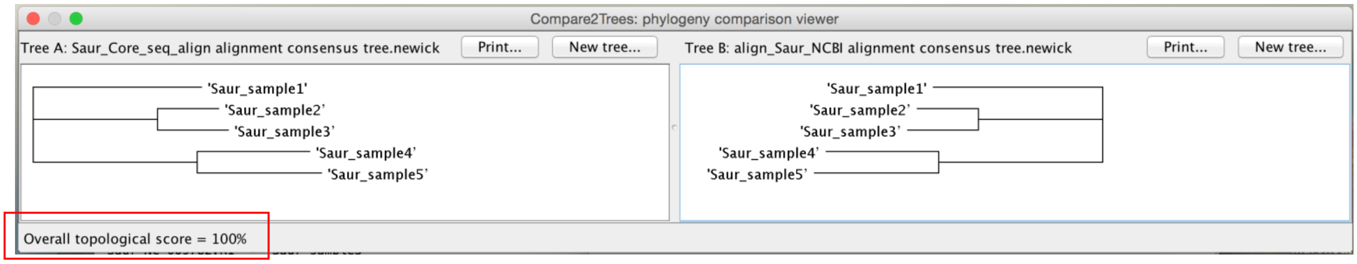

Record overall topological score.

Log of the analysis for each tree comparison

Staphylococcus aureus

```
*******************
Samples coding
'Saur_NC_007622.R1' = ‘Saur_sample1'
'Saur_NC_007795.R1' = 'Saur_sample2’
'Saur_NC_009782.R1' = 'Saur_sample3’
C5_S14_L001_R1_001 or 'SaurATCC25923.R1' = 'Saur_sample4’
'Saur_NC_017333.R1' = 'Saur_sample5’
```

Core seq:
('Saur_NC_007622.R1':0.338508,(('Saur_NC_007795.R1':0.12404599999999999,'Saur_NC_009782.R1':0.14270099999999997):0.25066,(C5_S14_L001_R1_001:0.225663,'Saur_NC_017333.R1':0.250963):0.329579):0.0);

Substituted names for Core seq:
('Saur_sample1':0.338508,(('Saur_sample2':0.12404599999999999,'Saur_sample3':0.14270099999999997):0.25066,('Saur_sample4':0.225663,'Saur_sample5':0.250963):0.329579):0.0);

NCBI seq:
('Saur_NC_007622.R1':0.339939,(('Saur_NC_007795.R1':0.12381000000000003,'Saur_NC_009782.R1':0.14254600000000006):0.24927699999999997,('SaurATCC25923.R1':0.226647,'Saur_NC_017333.R1':0.25006900000000004):0.329853):0.0);

Substituted names for NCBI seq:
('Saur_sample1':0.339939,(('Saur_sample2':0.12381000000000003,'Saur_sample3':0.14254600000000006):0.24927699999999997,('Saur_sample4':0.226647,'Saur_sample5':0.25006900000000004):0.329853):0.0);

Results:

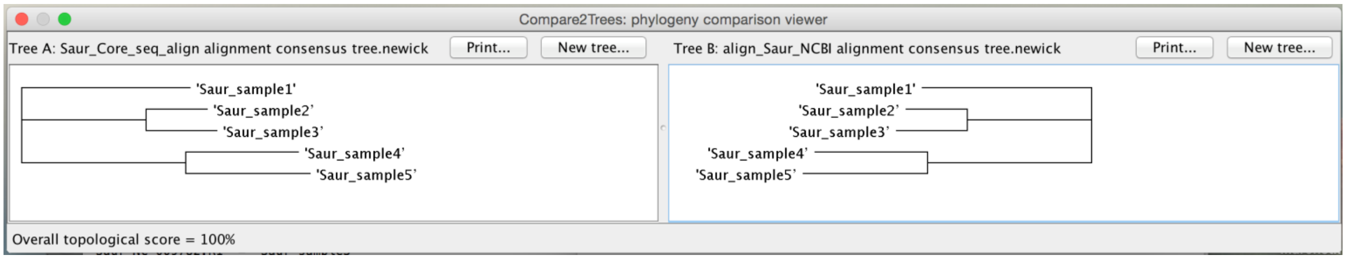

Enterococcus faecalis

****

Samples coding
'EfaeV583.R1' = 'Efae_sample1'
'Efae_NC_018221.R1' = 'Efae_sample2'
'Efae_NC_019770.R1' = 'Efae_sample3'
C46_S1_L001_R1_001 or 'EfaeATCC29212.R1' = 'Efae_sample4'
'Efae_NZ_CP004081.R1' = 'Efae_sample5'

Core seq:
('EfaeV583.R1':0.522708,('Efae_NC_018221.R1':0.363811,('Efae_NC_019770.R1':0.412639,(C46_S1_L001_R1_001:0.18541000000000002,'Efae_NZ_CP004081.R1':0.18834):0.06539399999999995):0.05600300000000025):0.0);

Substituted names for Core seq:
('Efae_sample1':0.522708,('Efae_sample2':0.363811,('Efae_sample3':0.412639,('Efae_sample4':0.18541000000000002,'Efae_sample5':0.18834):0.06539399999999995):0.05600300000000025):0.0);

NCBI seq:
('EfaeV583.R1':0.522938,('Efae_NC_018221.R1':0.36459,('Efae_NC_019770.R1':0.414211,('EfaeATCC29212.R1':0.184602,'Efae_NZ_CP004081.R1':0.188635):0.06522300000000003):0.05485099999999998):0.0);

Substituted names for NCBI seq:
('Efae_sample2':0.522938,('Efae_sample1':0.36459,('Efae_sample3':0.414211,('Efae_sample4':0.184602,'Efae_sample5':0.188635):0.06522300000000003):0.05485099999999998):0.0);

Results:

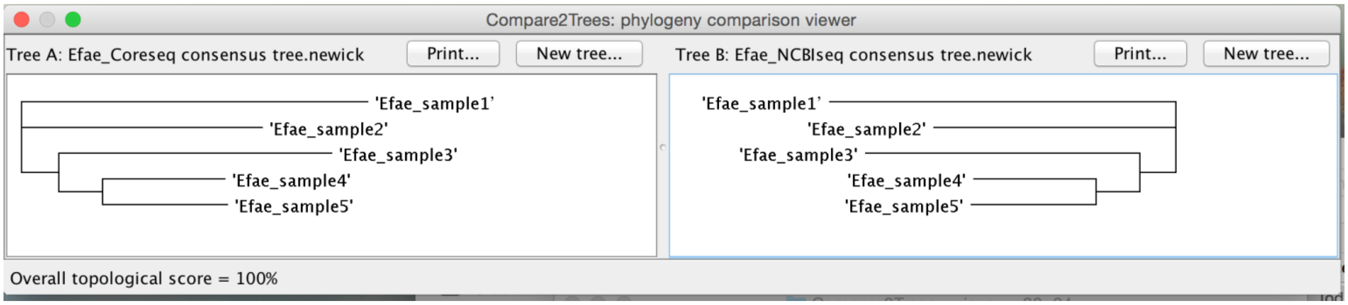

Negative control for Efae: switch Sample 1 and 2 places in NCBI file:
('Efae_sample2':0.522938,('Efae_sample1':0.36459,('Efae_sample3':0.414211,('Efae_sample4':0.184602,'Efae_sample5':0.188635):0.06522300000000003):0.05485099999999998):0.0);

Results in topological score = 75%

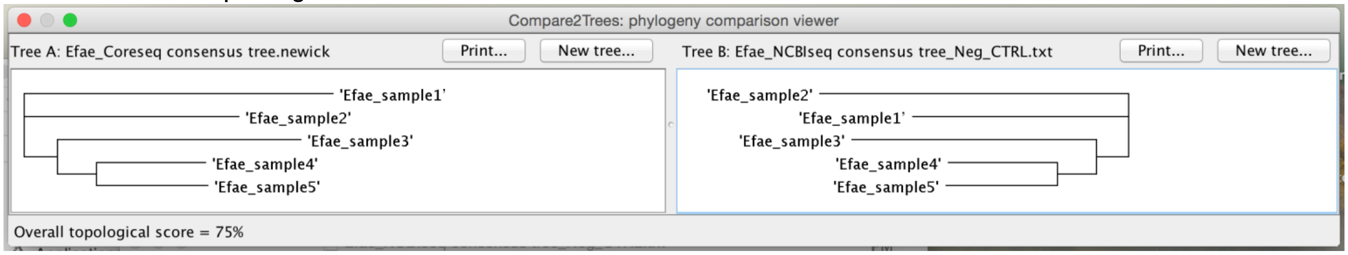

Escherichia coli

****

Samples coding
EcoK-12_NC_000913.R1 = Eco_sample1
C3_S2_L001_R1_001 or EcoATCC8739_NC_010468.R1 = Eco_sample2
EcoATCC25922_S16_L001_R1_001 or EcoATCC25922_NZ_CP009072.R1= Eco_sample3
C1_S1_L001_R1_001 or Eco_O157H7_EDL933_NZ_CP008957.R1 = Eco_sample4
EcoO157H7Sakai_NC_002695.R1 = Eco_sample5

Core seq:
('EcoK-
12_NC_000913.R1':0.10630300000000004,(C3_S2_L001_R1_001:0.16415899999999994,(Ec
oATCC25922_S16_L001_R1_001:1.524866,(C1_S1_L001_R1_001:0.006474000000000091,'Ec
oO157H7Sakai_NC_002695.R1':0.0):0.42080799999999985):0.1423270000000001):0.0);

Substituted names for Core seq:
(Eco_sample1:0.10630300000000004,(Eco_sample2:0.16415899999999994,(Eco_sample3:
1.524866,(Eco_sample4:0.006474000000000091,Eco_sample5:0.0):0.42080799999999985
):0.1423270000000001):0.0);

NCBI seq:
('EcoK-
12_NC_000913.R1':0.18175699999999995,('EcoATCC8739_NC_010468.R1':0.208916,('Eco
ATCC25922_NZ_CP009072.R1':0.6590369999999999,('EcoO157H7Sakai_NC_002695.R1':9.6
09999999999896E-4,'Eco_O157H7_EDL933_NZ_CP008957.R1':8.310000000000262E-
4):0.483762):0.34979800000000005):0.0);

Substituted names for NCBI seq:
(Eco_sample1:0.18175699999999995,(Eco_sample2:0.208916,(Eco_sample3:0.659036999
9999999,(Eco_sample5:9.609999999999896E-4,Eco_sample4:8.310000000000262E-
4):0.483762):0.34979800000000005):0.0);

Results:

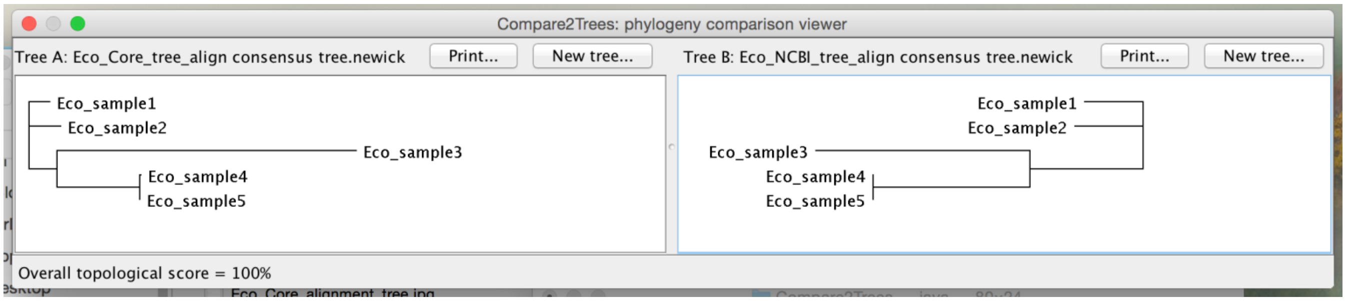

Stenotrophomonas maltophilia

****
Samples coding
Smal_JV3_NC_015947.R1 = Smalt_sample1
Smal_D457_NC_017671.R1 = Smalt_sample2
Smal_R551_3_NC_011071.R1 = Smalt_sample3
C51_S5_L001_R1_001 or Smal_ATCC13637_NZ_CP008838.R1 = Smalt_sample4
Smal_K279a_NC_010943.R1 = Smalt_sample5

Core seq:
('Smal_JV3_NC_015947.R1':0.23491700000000004,('Smal_D457_NC_017671.R1':0.313368
,('Smal_R551_3_NC_011071.R1':0.737567,(C51_S5_L001_R1_001:0.03381299999999998,'
Smal_K279a_NC_010943.R1':0.08788299999999999):0.19598300000000002):0.2023869999
9999998):0.0);

Substituted names for Core seq:
(Smalt_sample1:0.23491700000000004,(Smalt_sample2:0.313368,(Smalt_sample3:0.737567,(Smalt_sample4:0.03381299999999998,Smalt_sample5:0.08788299999999999):0.19598300000000002):0.20238699999999998):0.0);

NCBI seq:
('Smal_JV3_NC_015947.R1':0.242444,('Smal_D457_NC_017671.R1':0.35339300000000007,('Smal_R551_3_NC_011071.R1':0.8331699999999999,('Smal_ATCC13637_NZ_CP008838.R1':0.0023869999999999917,'Smal_K279a_NC_010943.R1':0.11181799999999997):0.056333999999999995):0.228198):0.0);

Substituted names for NCBI seq:
(Smalt_sample1:0.242444,(Smalt_sample2:0.35339300000000007,(Smalt_sample3:0.8331699999999999,(Smalt_sample4:0.0023869999999999917,Smalt_sample5:0.11181799999999997):0.056333999999999995):0.228198):0.0);

Results:

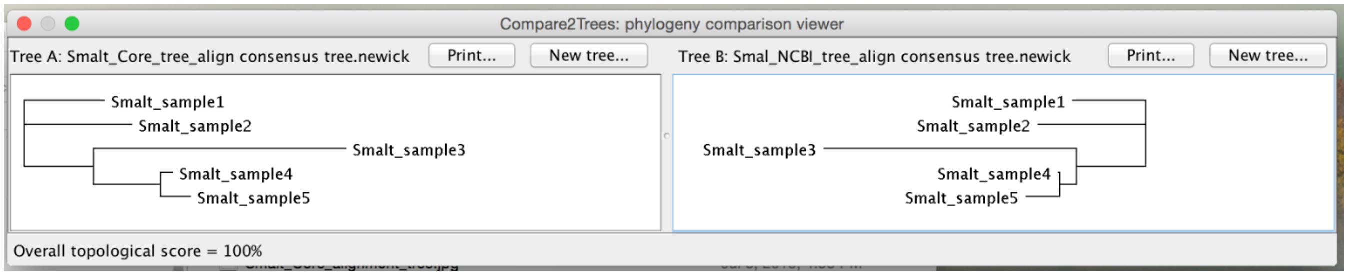

Salmonella enterica

****

Samples coding
C74-1_S3_L001_R1_001 or C74_M14X00933_Infantis = Salm_sample1
C76-1_S5_L001_R1_001 or C76_M12X03253_Worthington = Salm_sample2
C75-1_S4_L001_R1_001 or C75_M14X04729_Adelaide = Salm_sample3
C73-1_S2_L001_R1_001 or SRX157800sra_data = Salm_sample4
C77_S6_L001_R1_001 or C77_M14X04723_Saintpaul = Salm_sample5

Core seq:
(C74-1_S3_L001_R1_001:0.282951,C76-1_S5_L001_R1_001:0.409031,(C75-1_S4_L001_R1_001:0.406701,(C73-1_S2_L001_R1_001:0.25101999999999997,C77_S6_L001_R1_001:0.24577499999999997):0.037896000000000004):0.015641999999999999);

Substituted names for Core seq:
(Salm_sample1:0.282951,Salm_sample2:0.409031,(Salm_sample3:0.406701,(Salm_sample4:0.25101999999999997,Salm_sample5:0.24577499999999997):0.037896000000000004):0.015641999999999999);

```
CDC seq:
(C74_M14X00933_Infantis:0.27533,C76_M12X03253_Worthington:0.460218,(C75_M14X047
29_Adelaide:0.422506,(SRX157800sra_data:0.270891,C77_M14X04723_Saintpaul:0.2280
3300000000004):0.023168999999999995):0.016614999999999999);

Substituted names for CDC seq:
(Salm_sample1:0.27533,Salm_sample2:0.460218,(Salm_sample3:0.422506,(Salm_sample
4:0.270891,Salm_sample5:0.22803300000000004):0.023168999999999995):0.0166149999
9999999);
```

Results:

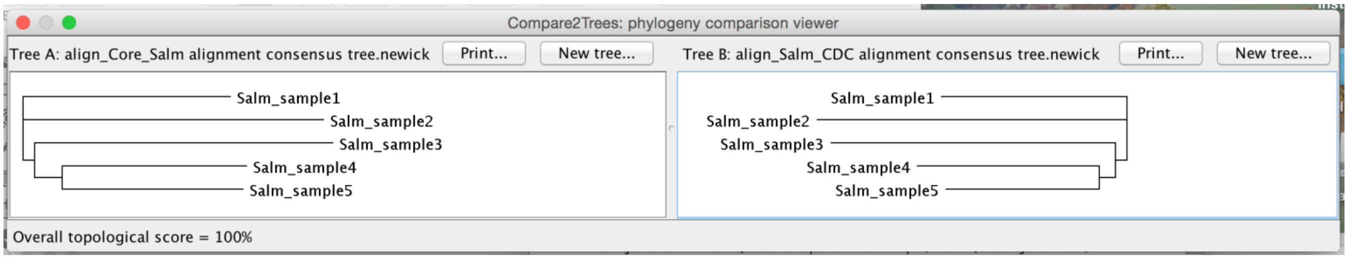

Appendix 2. Calculation of platform performance parameters

Parameter 1: *Average read length with ≥Q30 (= avg read length after trimming), (bp)*

Method of calculation:

Choose option to create Trim report in CLCbio when performing quality trimming.

Pick to save trimmed files and to create report:

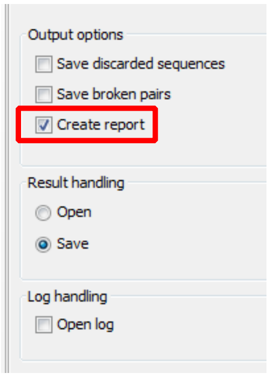

Open Trim report by double clicking on the corresponding file in the navigation area.

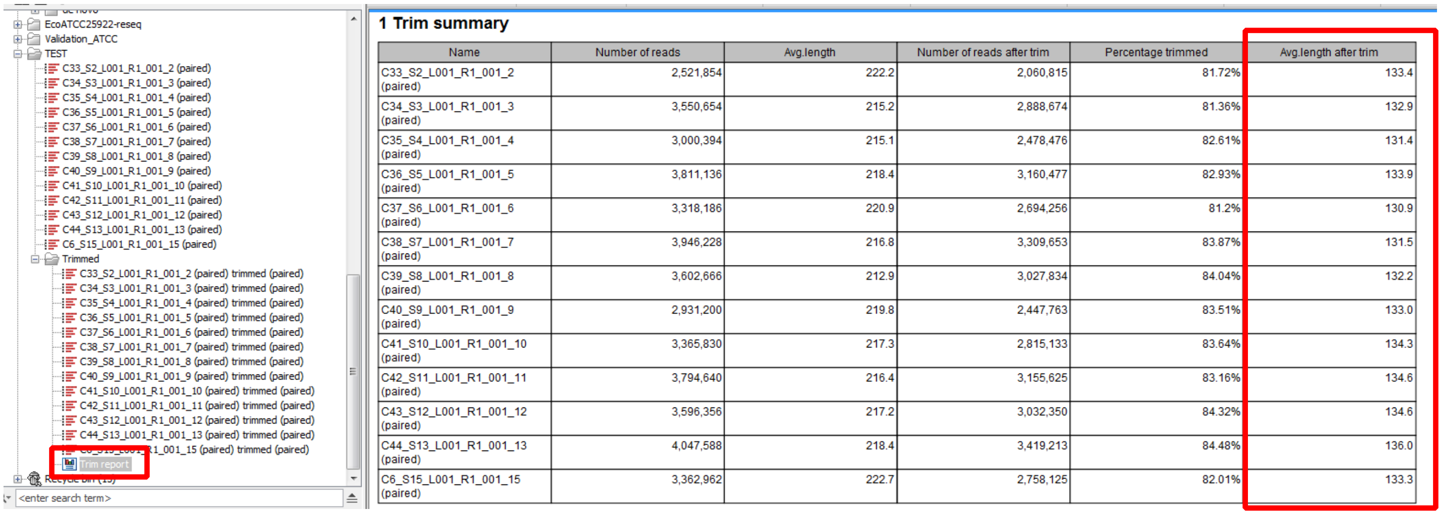

1 Trim summary

Name	Number of reads	Avg.length	Number of reads after trim	Percentage trimmed	Avg.length after trim
C33_S2_L001_R1_001_2 (paired)	2,521,854	222.2	2,060,815	81.72%	133.4
C34_S3_L001_R1_001_3 (paired)	3,550,654	215.2	2,888,674	81.36%	132.9
C35_S4_L001_R1_001_4 (paired)	3,000,394	215.1	2,478,476	82.61%	131.4
C36_S5_L001_R1_001_5 (paired)	3,811,136	218.4	3,160,477	82.93%	133.9
C37_S6_L001_R1_001_6 (paired)	3,318,186	220.9	2,694,256	81.2%	130.9
C38_S7_L001_R1_001_7 (paired)	3,946,228	216.8	3,309,653	83.87%	131.5
C39_S8_L001_R1_001_8 (paired)	3,602,666	212.9	3,027,834	84.04%	132.2
C40_S9_L001_R1_001_9 (paired)	2,931,200	219.8	2,447,763	83.51%	133.0
C41_S10_L001_R1_001_10 (paired)	3,365,830	217.3	2,815,133	83.64%	134.3
C42_S11_L001_R1_001_11 (paired)	3,794,640	216.4	3,155,625	83.16%	134.6
C43_S12_L001_R1_001_12 (paired)	3,596,356	217.2	3,032,350	84.32%	134.6
C44_S13_L001_R1_001_13 (paired)	4,047,588	218.4	3,419,213	84.48%	136.0
C6_S15_L001_R1_001_15 (paired)	3,362,962	222.7	2,758,125	82.01%	133.3

In the example above Average read length after trim (with quality ≥Q30) is 133.4bp. Average length after trim should be > 109bp.

Parameter 2: *Read length at which 75% of bases have quality score ≥Q30, (bp)*

Method of calculation:

Perform quality analysis of raw reads in CLCbio. In generated report find Chapter 3. Per-base analysis – 3.5 Quality Distribution:

3.5 Quality distribution

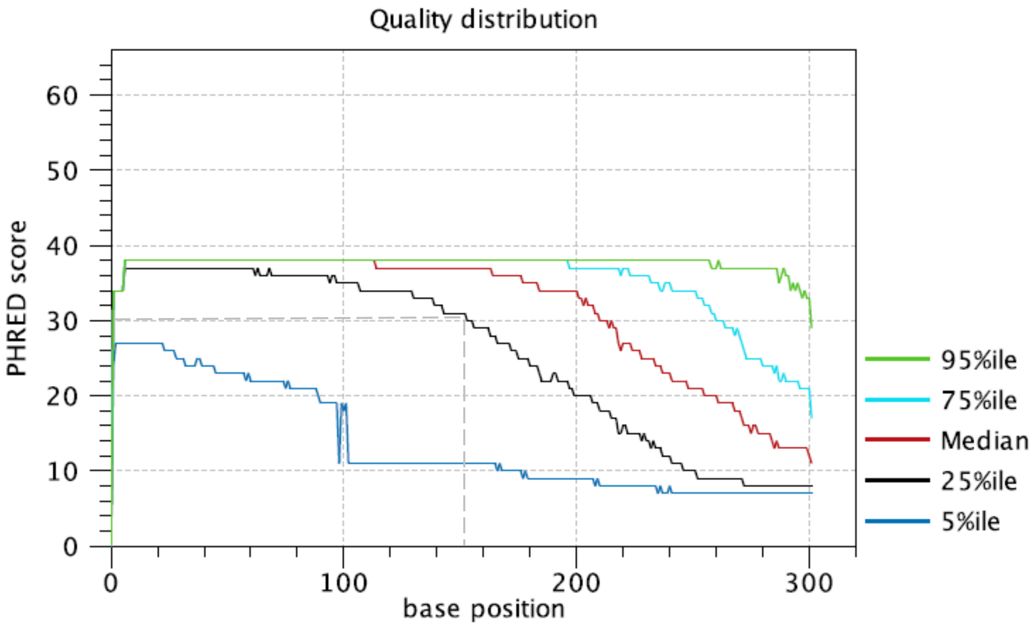

Base-quality distribution along the base positions.
x: base position
y: median & percentiles of quality scores observed at that base position

On the Per-base quality distribution diagram mark at what base position does the 25%ile graph (black line) fall below PHRED score 30. In example above the read length at which 75% of bases have quality score ≥Q30 equals ≈ 150bp. This parameter should be > 86 base position.

Parameter 3: *Average depth of coverage of genome* and **Parameter 4:** *Percentage of genome covered (after mobile elements masking)*

Method of calculation:

Create a detailed report of mapping when it is done:

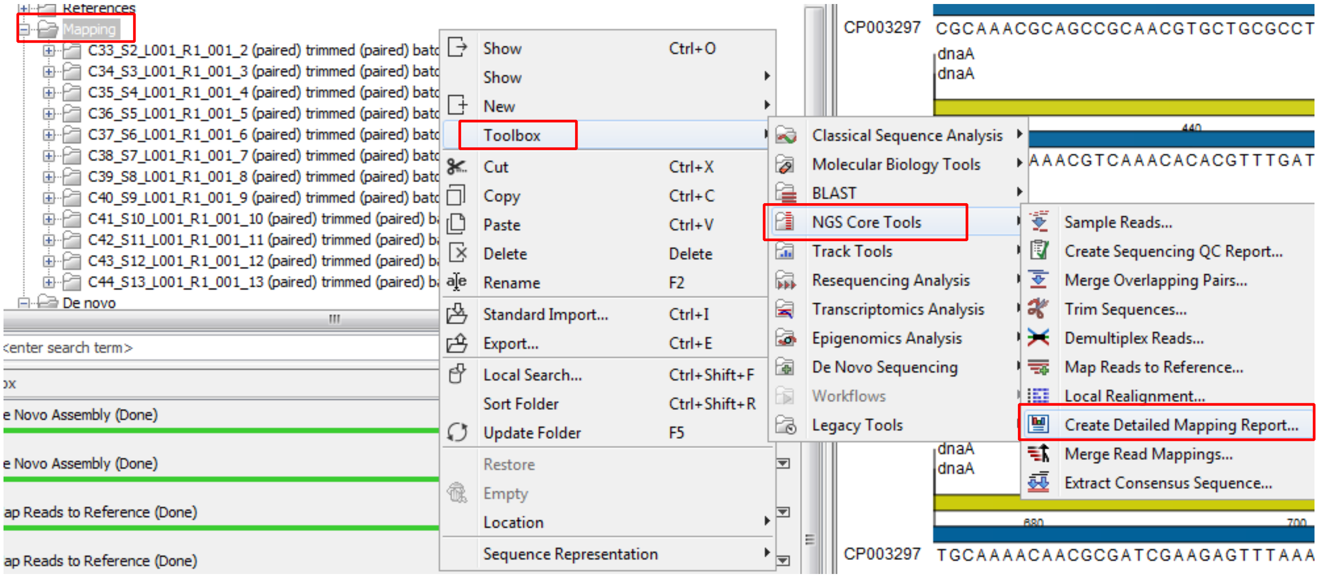

Do batch analysis of the folder “Mapping”:

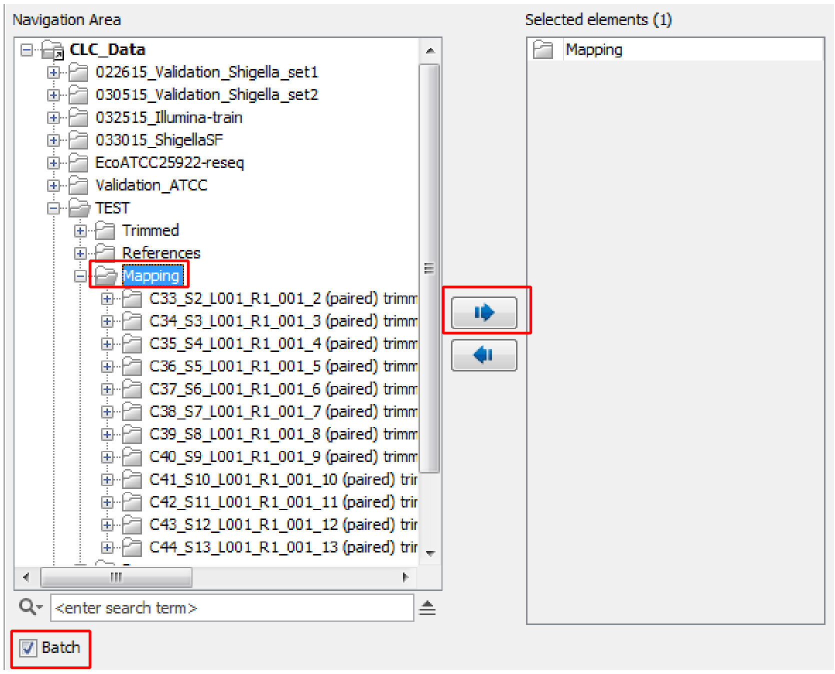

Leave all mappings in the analysis list:

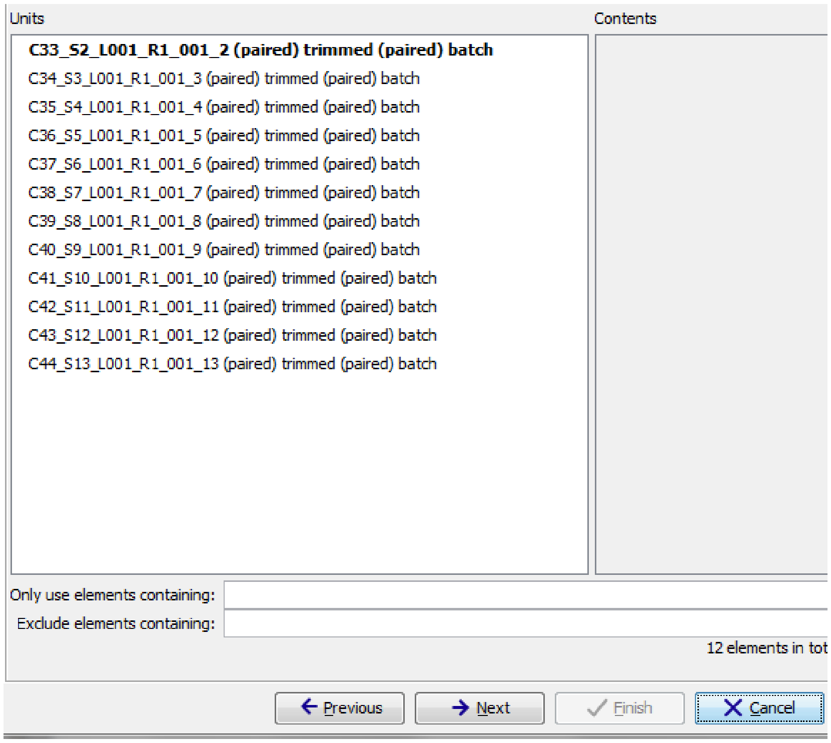

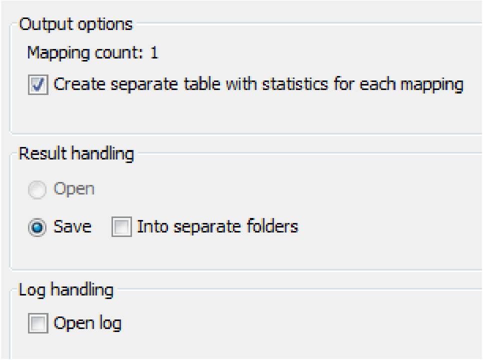

Finish

Open detailed mapping report for each sample one by one. Record following metrics: fraction of reference covered and average coverage:

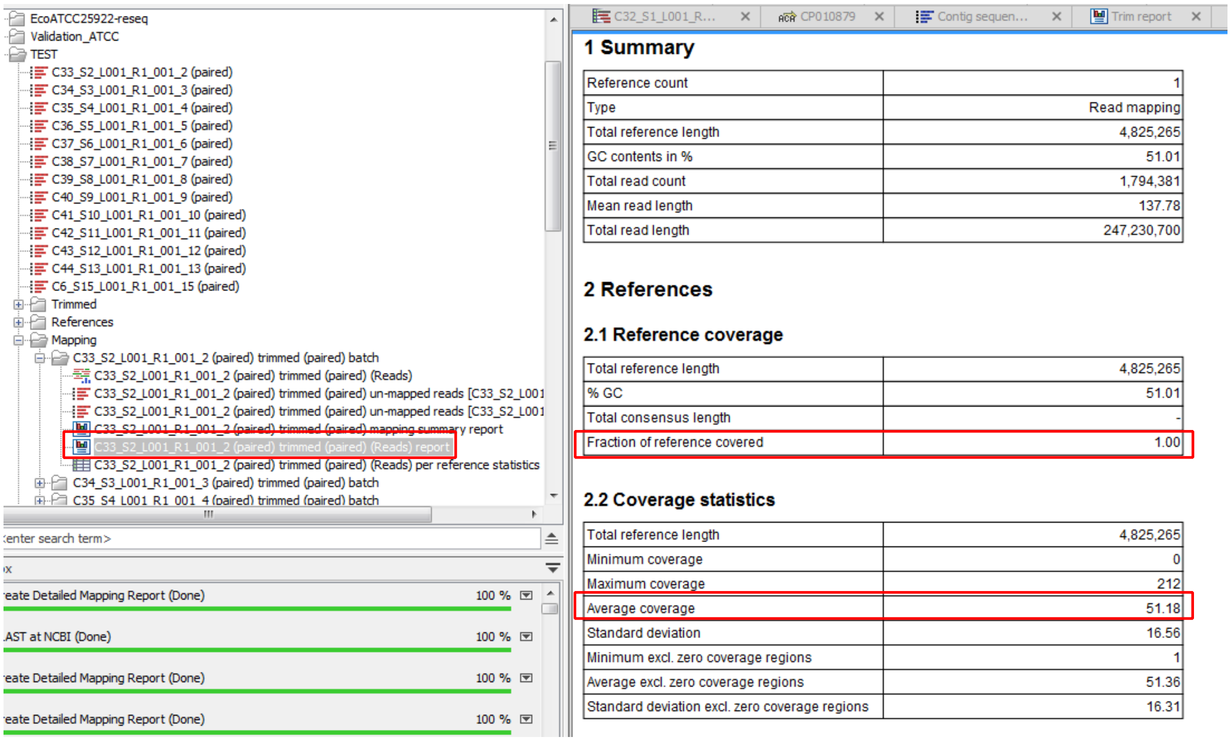

In the example above average coverage = 51.18X, Fraction of reference covered= 1.00 (=100%).

Average coverage should be ≥ 15X. Fraction of reference covered should be > 0.8 (80%).

Parameter 5: *Uniformity of coverage at 10x/5x*

Method of calculation:

Create annotation track from the reference sequence which was used for mapping:

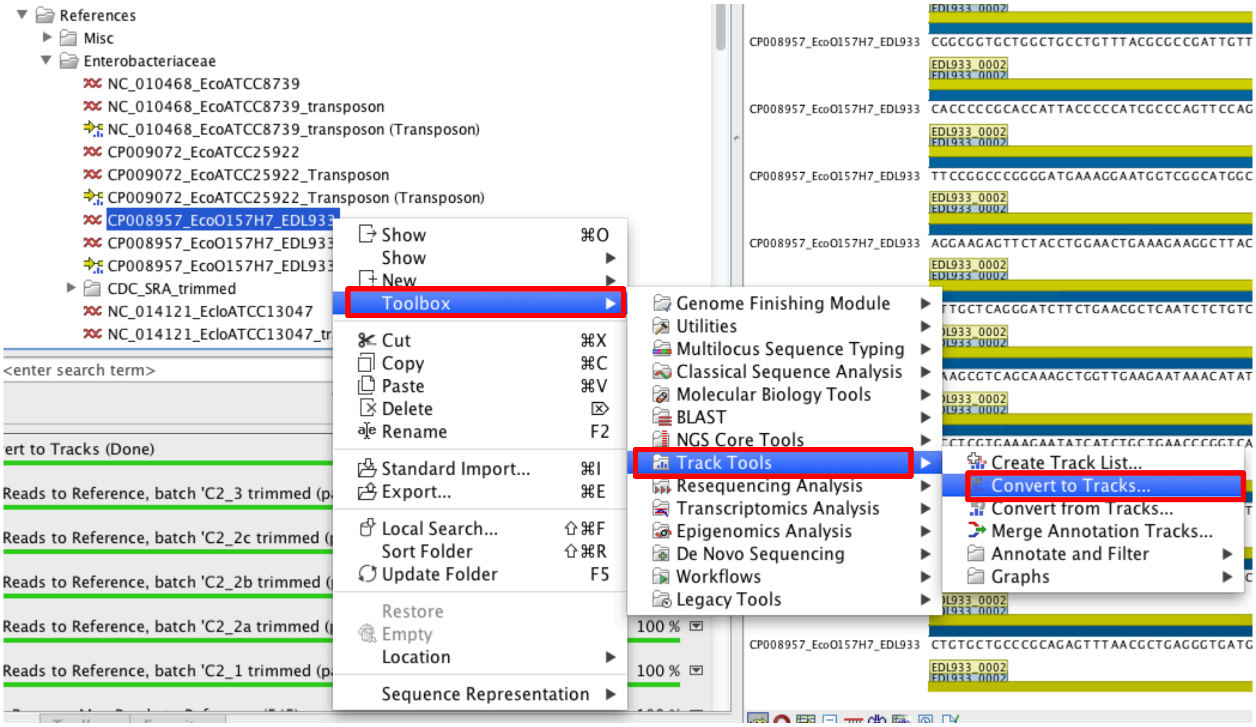

To choose annotation type click green plus button:

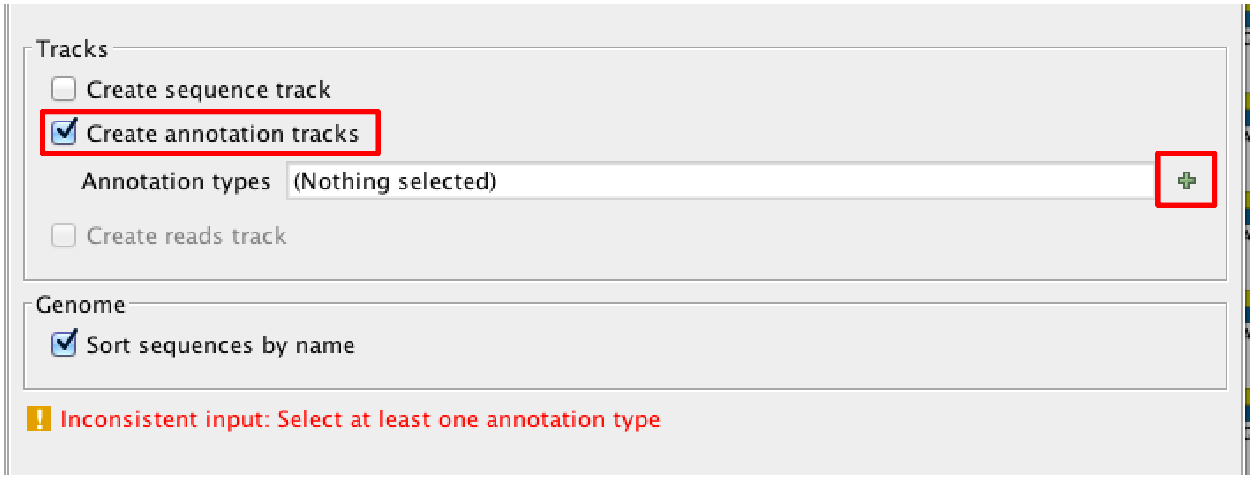

Pick only annotation type "CDS":

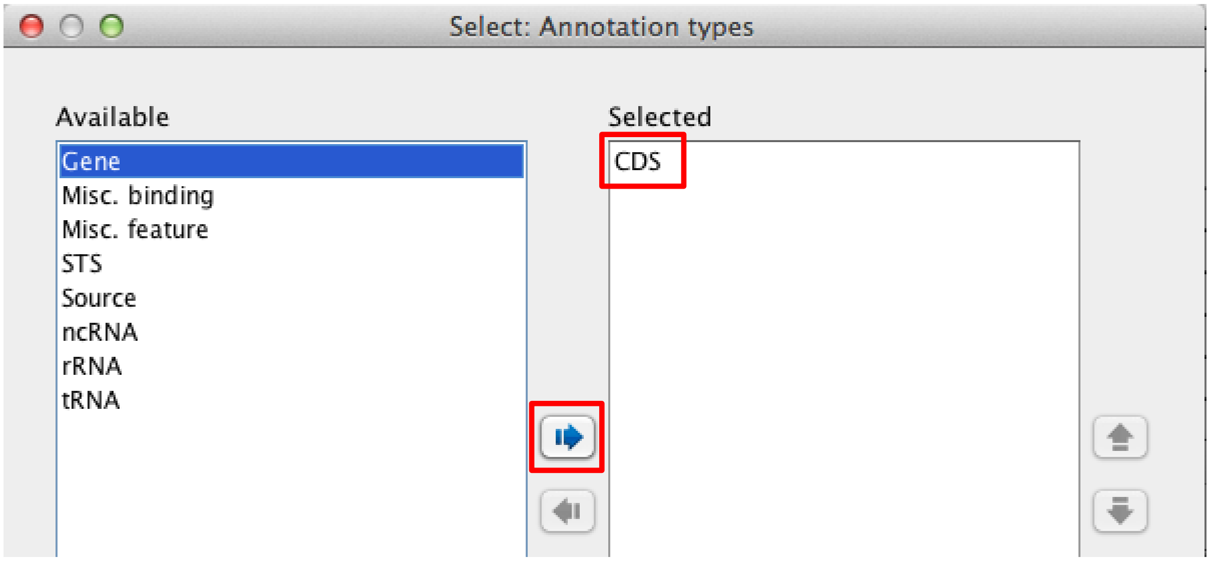

Save CDS annotation track in the same folder with reference file:

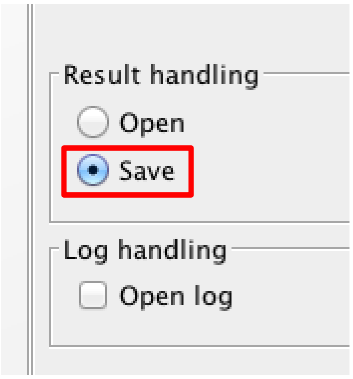

Select mapping file and perform analysis: Toolbox → Resequencing Analysis → Create Statistics for Target Regions…

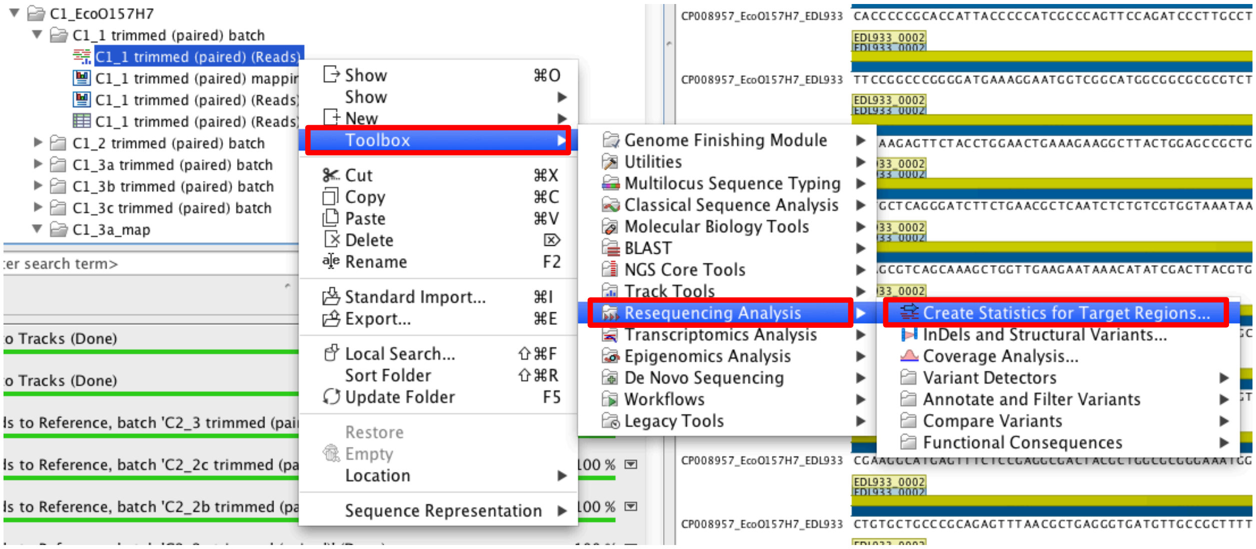

Select one file at a time or batch

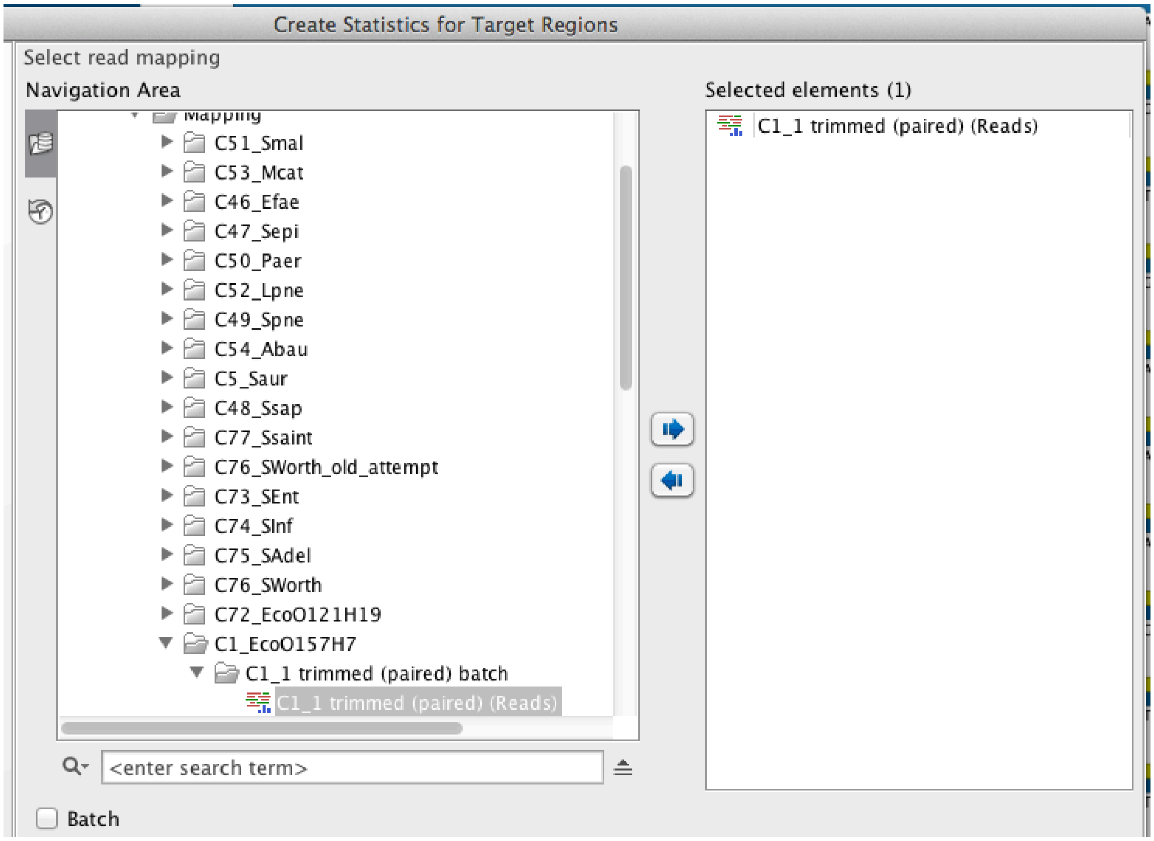

As Track of Target Regions select CDS annotation track created from the reference file. Minimum coverage= 10.

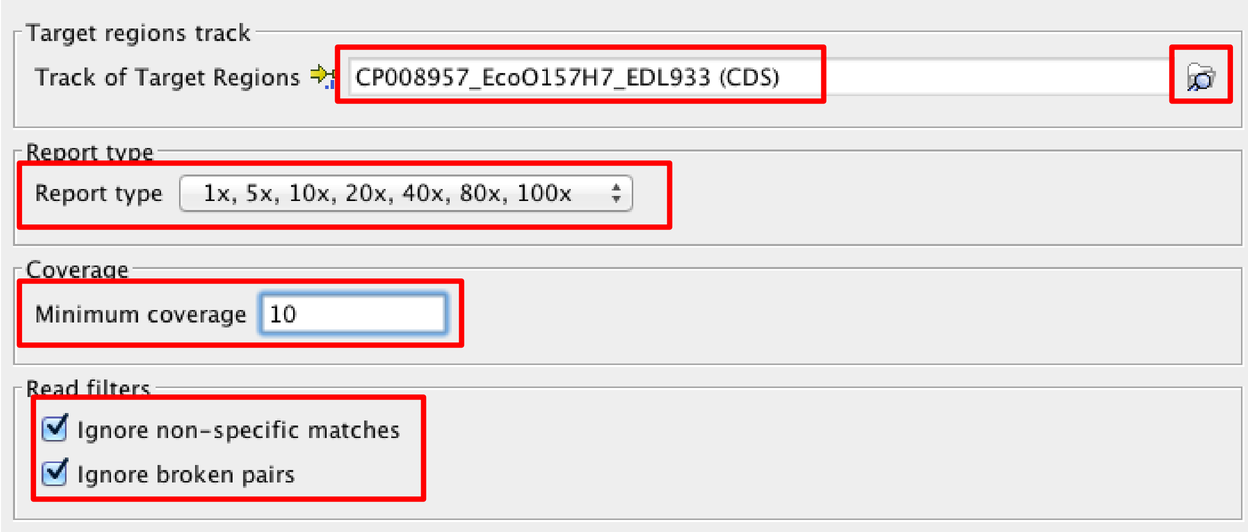

Create report:

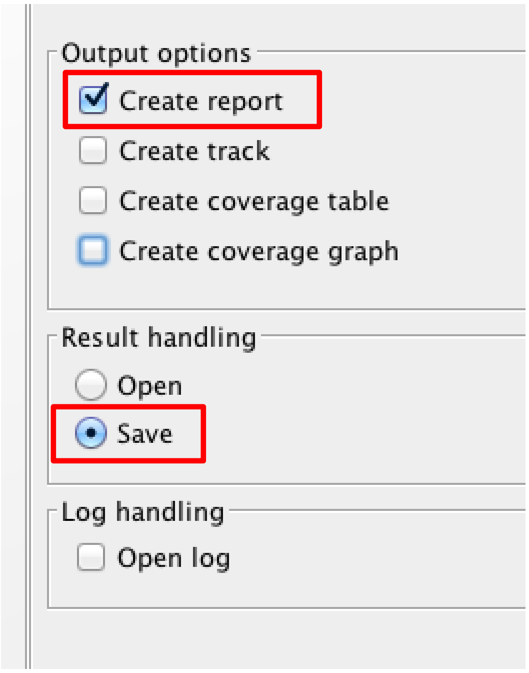

Open report file

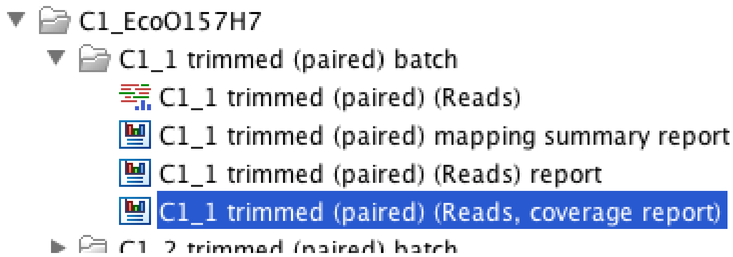

Find table “1.5 Minimum coverage of target regions”

Record value for 10x and 5x coverage.

Example of the table:

1.5 Minimum coverage of target regions

Coverage	
1 x	89.36%
5 x	89.07%
10 x	88.09%
20 x	69.28%
40 x	6.10%
80 x	0.06%
100 x	0.03%

Sample in the example above has 88.09% of the positions on the target covered by at least 10 bases (10x coverage) and 89.07% of the positions covered by at least 5 bases (5x coverage).

>50% of positions on the target should have coverage ≥10x and >70% of positions should have coverage ≥5x

Parameter 6: *N50 for De Novo assembly*

Method of calculation:

Open *De novo* assembly report:

- C1_2 trimmed (paired) batch
  - C1_2 trimmed (paired) assembly
  - C1_2 trimmed (paired) assembly summary report
  - Contig sequences from C1_2 trimmed (paired) assembly

Record N50 value from table "1.2 Contig measurements (including scaffolded regions)"

1 C1_2 trimmed (paired) assembly summary report

1.1 Nucleotide distribution

Nucleotide	Count	Frequency
Adenine (A)	1,340,047	25.0%
Cytosine (C)	1,352,000	25.2%
Guanine (G)	1,347,377	25.1%
Thymine (T)	1,330,569	24.8%
Any nucleotide (N)	597	0.0%

1.2 Contig measurements (including scaffolded regions)

	Length
N75	43,146
N50	96,173
N25	157,754
Minimum	119
Maximum	313,894
Average	10,191
Count	527
Total	5,370,590

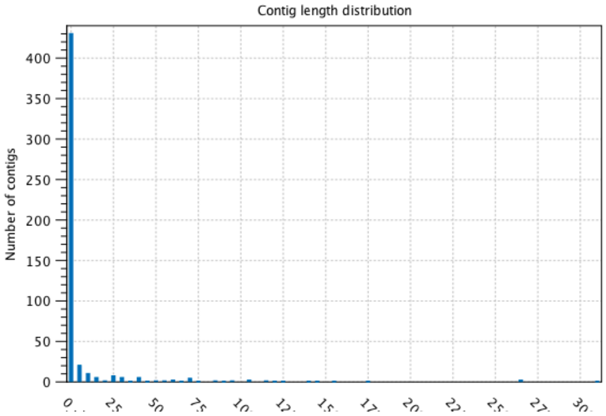

Parameter 7: *PhiX error rate,* **Parameter 8:** *Percent of bases with quality score >Q30 for the run*, **Parameter 9:** *Cluster density for the run, (K/mm2),* and **Parameter 9:** *Cluster passing filter of the run*

Method of calculation:

Record information about Percent of bases with quality score >Q30 for the run and Cluster density for the run, (K/mm2) from Illumina Sequencing Analysis Viewer (SAV)

Open folder with MiSeq output in SAV. Open tab "Summary":

Record total %Q>=30 and Error Rate (%). Record Density (K/mm2) and Cluster PF (%) values from any of the reads:

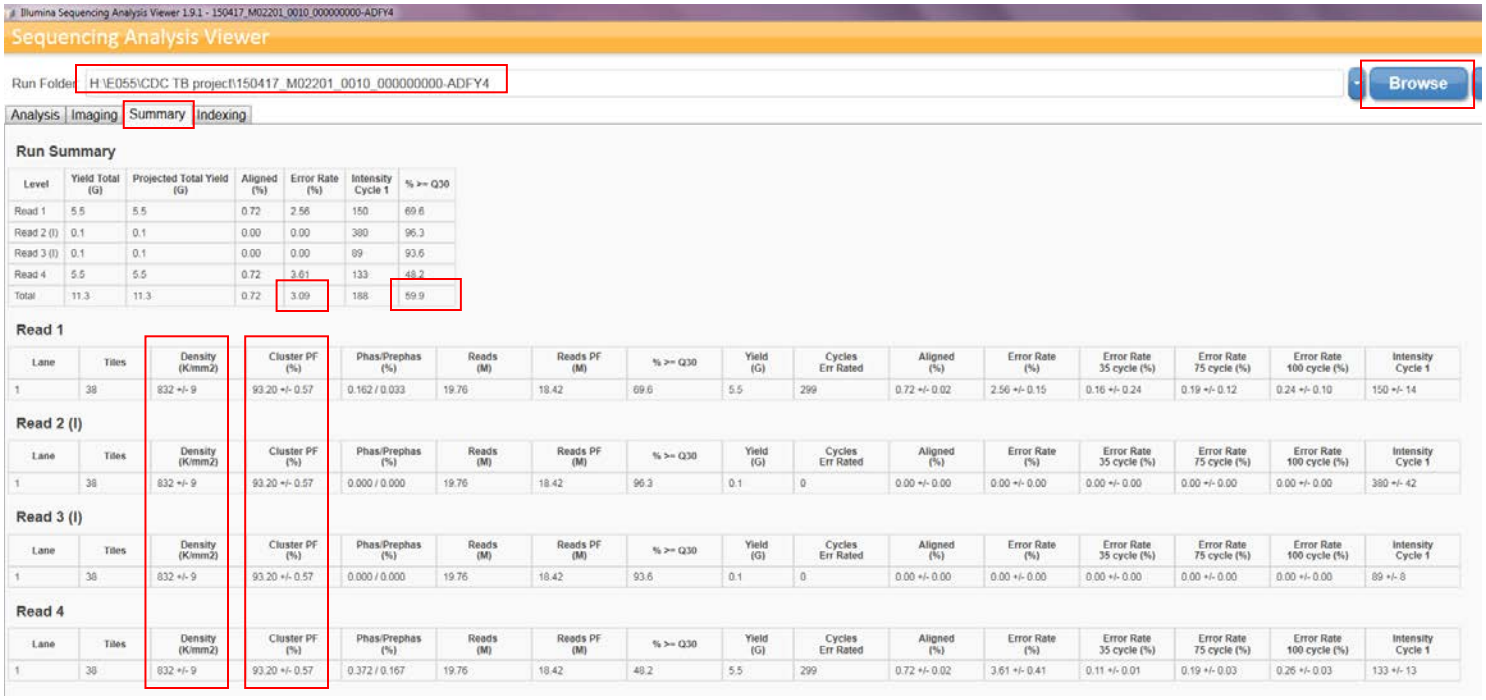

Run Summary

Level	Yield Total (G)	Projected Total Yield (G)	Aligned (%)	Error Rate (%)	Intensity Cycle 1	% >= Q30
Read 1	5.5	5.5	0.72	2.56	150	69.6
Read 2 (I)	0.1	0.1	0.00	0.00	380	96.3
Read 3 (I)	0.1	0.1	0.00	0.00	89	93.6
Read 4	5.5	5.5	0.72	3.61	133	48.2
Total	11.3	11.3	0.72	3.09	188	59.9

Read 1

Lane	Tiles	Density (K/mm2)	Cluster PF (%)	Phas/Prephas (%)	Reads (M)	Reads PF (M)	% >= Q30	Yield (G)	Cycles Err Rated	Aligned (%)	Error Rate (%)	Error Rate 35 cycle (%)	Error Rate 75 cycle (%)	Error Rate 100 cycle (%)	Intensity Cycle 1
1	38	832 +/- 9	93.20 +/- 0.57	0.162 / 0.033	19.76	18.42	69.6	5.5	299	0.72 +/- 0.02	2.56 +/- 0.15	0.16 +/- 0.24	0.19 +/- 0.12	0.24 +/- 0.10	150 +/- 14

Read 2 (I)

Lane	Tiles	Density (K/mm2)	Cluster PF (%)	Phas/Prephas (%)	Reads (M)	Reads PF (M)	% >= Q30	Yield (G)	Cycles Err Rated	Aligned (%)	Error Rate (%)	Error Rate 35 cycle (%)	Error Rate 75 cycle (%)	Error Rate 100 cycle (%)	Intensity Cycle 1
1	38	832 +/- 9	93.20 +/- 0.57	0.000 / 0.000	19.76	18.42	96.3	0.1	0	0.00 +/- 0.00	0.00 +/- 0.00	0.00 +/- 0.00	0.00 +/- 0.00	0.00 +/- 0.00	380 +/- 42

Read 3 (I)

Lane	Tiles	Density (K/mm2)	Cluster PF (%)	Phas/Prephas (%)	Reads (M)	Reads PF (M)	% >= Q30	Yield (G)	Cycles Err Rated	Aligned (%)	Error Rate (%)	Error Rate 35 cycle (%)	Error Rate 75 cycle (%)	Error Rate 100 cycle (%)	Intensity Cycle 1
1	38	832 +/- 9	93.20 +/- 0.57	0.000 / 0.000	19.76	18.42	93.6	0.1	0	0.00 +/- 0.00	0.00 +/- 0.00	0.00 +/- 0.00	0.00 +/- 0.00	0.00 +/- 0.00	89 +/- 8

Read 4

Lane	Tiles	Density (K/mm2)	Cluster PF (%)	Phas/Prephas (%)	Reads (M)	Reads PF (M)	% >= Q30	Yield (G)	Cycles Err Rated	Aligned (%)	Error Rate (%)	Error Rate 35 cycle (%)	Error Rate 75 cycle (%)	Error Rate 100 cycle (%)	Intensity Cycle 1
1	38	832 +/- 9	93.20 +/- 0.57	0.372 / 0.167	19.76	18.42	48.2	5.5	299	0.72 +/- 0.02	3.61 +/- 0.41	0.11 +/- 0.01	0.19 +/- 0.03	0.26 +/- 0.03	133 +/- 13

In the example above Percent of bases with quality score >Q30 for the run is 59.9%, and Cluster density for the run is 832 K/mm^2. Cluster passing filter of the run is 93.20%. PhiX error rate is 3.09%

Appendix 3. Genome size information

In order to look up genome size of the reference strain use accession number to search for it's genome at the NCBI Genome site: http://www.ncbi.nlm.nih.gov/genome/ . Type in species of the reference:

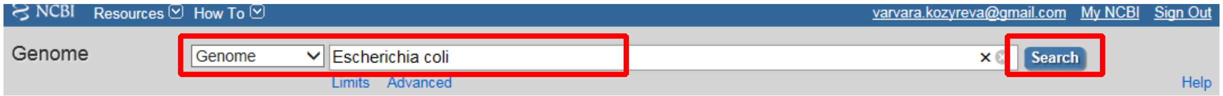

Click on a link "Genome Assembly and Annotation report":

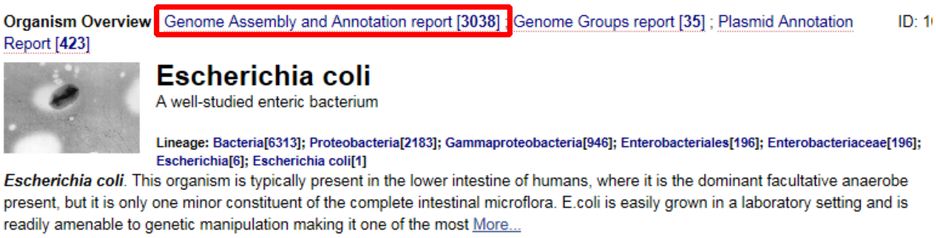

Sort genomes by the level of completion:

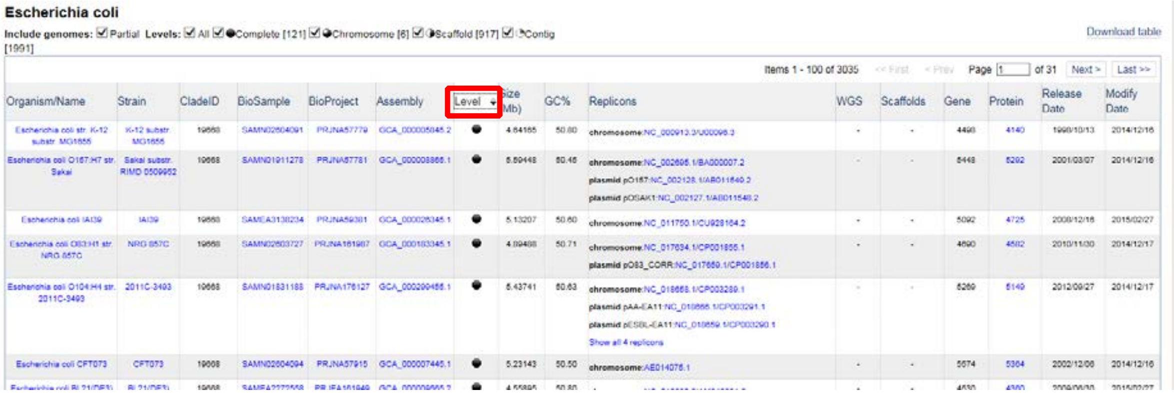

Find reference strain by it's accession number, e.g. NC_002695 (use Ctrl+F function in browser). Record Genome size shown in the table in megabases (Mb):

Escherichia coli

Include genomes: ☑ Partial **Levels:** ☑ All ☑ ●Complete [121] ☑ ◕Chromosome [6] ☑ ◑Scaffold [917] ☑ ◔Contig [1991]

Download table

Items 1 - 100 of 3035 << First < Prev Page 1 of 31 Next > Last >>

Organism/Name	Strain	CladeID	BioSample	BioProject	Assembly	Level	Size (Mb)	GC%	Replicons	WGS	Scaffolds	Gene	Protein	Release Date	Modify Date
Escherichia coli str. K-12 substr. MG1655	K-12 substr. MG1655	19668	SAMN02604091	PRJNA57779	GCA_000005845.2	●	4.64165	50.80	**chromosome**:NC_000913.3/U00096.3	-	-	4498	4140	1998/10/13	2014/12/16
Escherichia coli O157:H7 str. Sakai	Sakai substr. RIMD 0509952	19668	SAMN01911278	PRJNA57781	GCA_000008865.1	●	5.59448	50.45	**chromosome**:NC_002695.1/BA000007.2<br>**plasmid** pO157:NC_002128.1/AB011549.2<br>**plasmid** pOSAK1:NC_002127.1/AB011548.2	-	-	5448	5292	2001/03/07	2014/12/16
Escherichia coli IAI39	IAI39	19668	SAMEA3138234	PRJNA59381	GCA_000026345.1	●	5.13207	50.60	**chromosome**:NC_011750.1/CU928164.2	-	-	5092	4725	2008/12/16	2015/02/27

Appendix 4. Average covered genome size

To calculate average covered genome size:

1) Determine genome size as described in Appendix 3.

2) Determine Fraction of reference covered (after mobile elements masking) as described in Appendix 2 for Parameter 4.

3) Average covered genome size = (Genome size) x (Fraction of reference covered)

Appendix 5. Data analysis for test validation

Trimming of the reads

Open CLCbio by clicking the icon on the desktop:

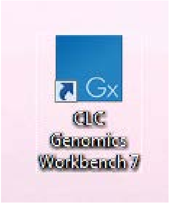

Create new folder for the run or set of samples which you are going to analyze by clicking folder icon in the Navigation area (left right corner of the window):

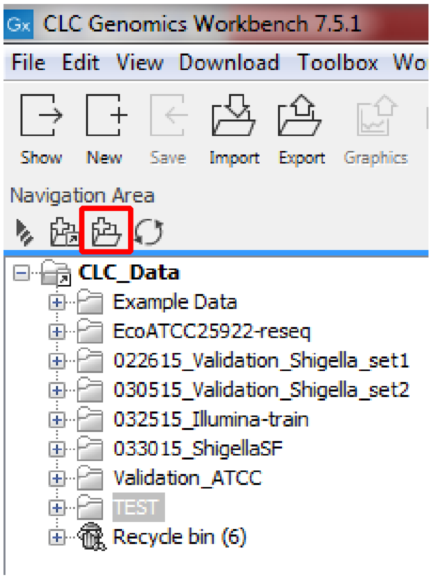

Import FASTQ files (two files for each sample corresponding to both paired-end reads) for all sample which you are going to analyze.

Select new folder. Then click on the button “Import” in the tools panel at a top of the screen and select “Illumina…”:

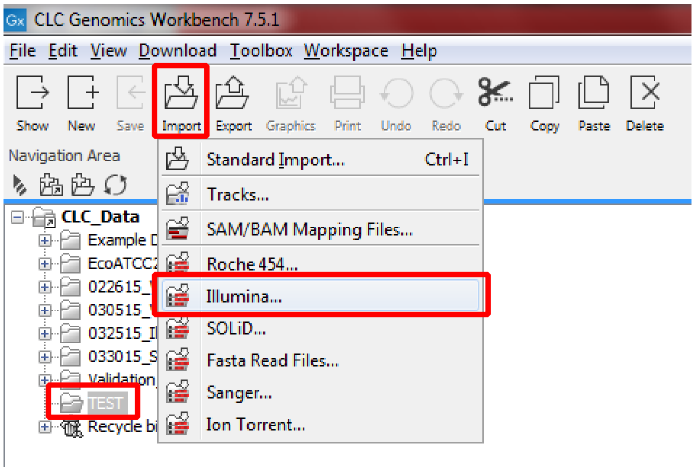

Browse to the location of the FASTQ files inside of the run folder: e.g., 150410_M02201_0008_000000000-AAMFL\Data\Intensities\BaseCalls\

Leave default import parameters. Select two files for each sample corresponding to both paired-end reads (R1- read 1, R2- read 2), e.g.:

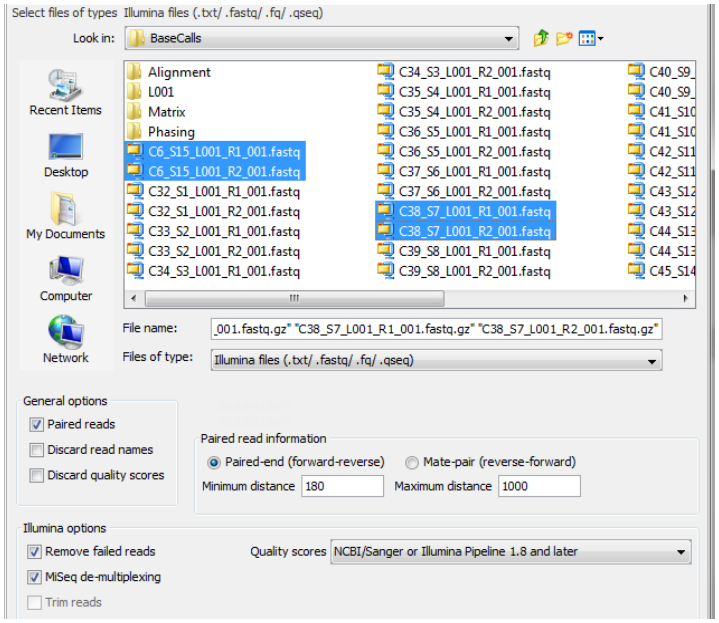

Next

Chose to save imported files:

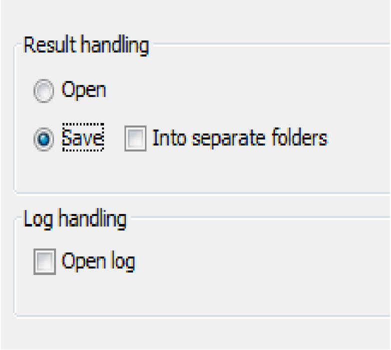

Chose newly created folder as the directory to import files to:

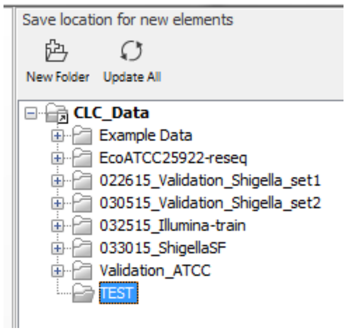

Progress of all the processes is reflected in the field in the left bottom of the screen under the tab “Processes”:

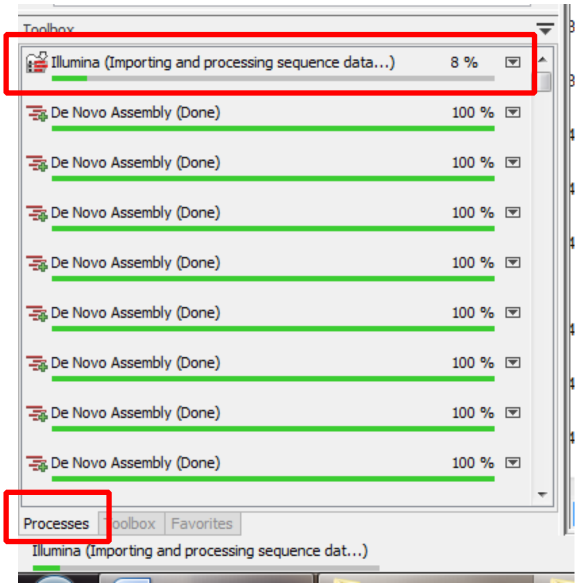

Imported files will appear inside of the selected folder:

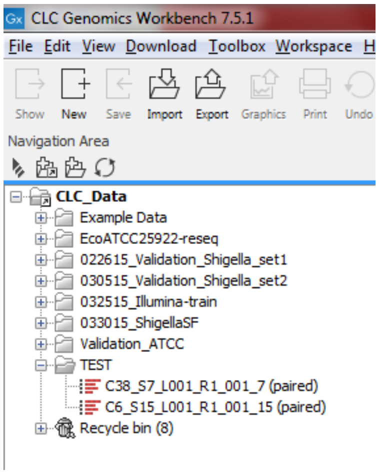

Later you can import more files pertaining to the same project into the same folder.

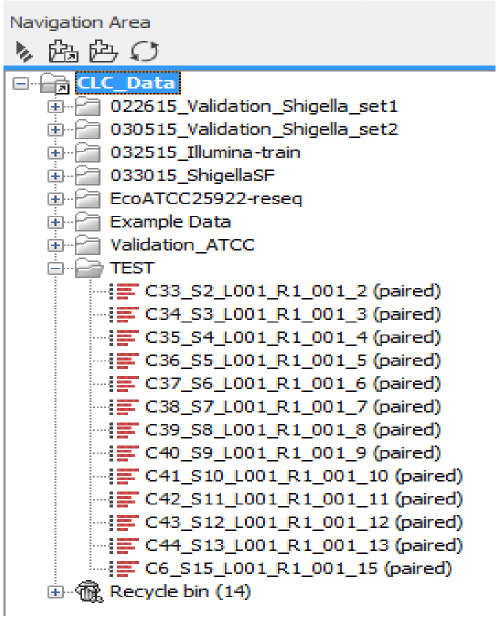

Perform quality trimming. For that: Select all files. Click with right mouse button on the files, Pick: Toolbox →NGS Core Tools → Trim Sequences…

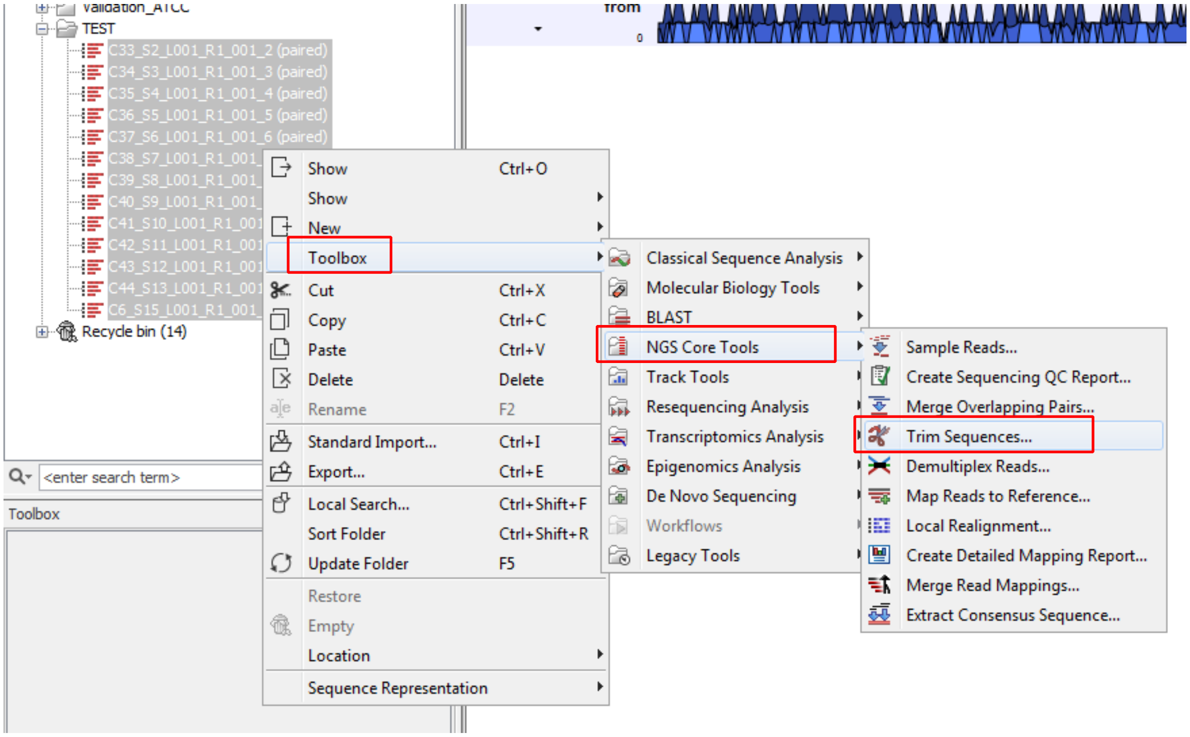

Move files you want to perform operation with to the left part of the screen by having them selected and clicking arrow to the left:

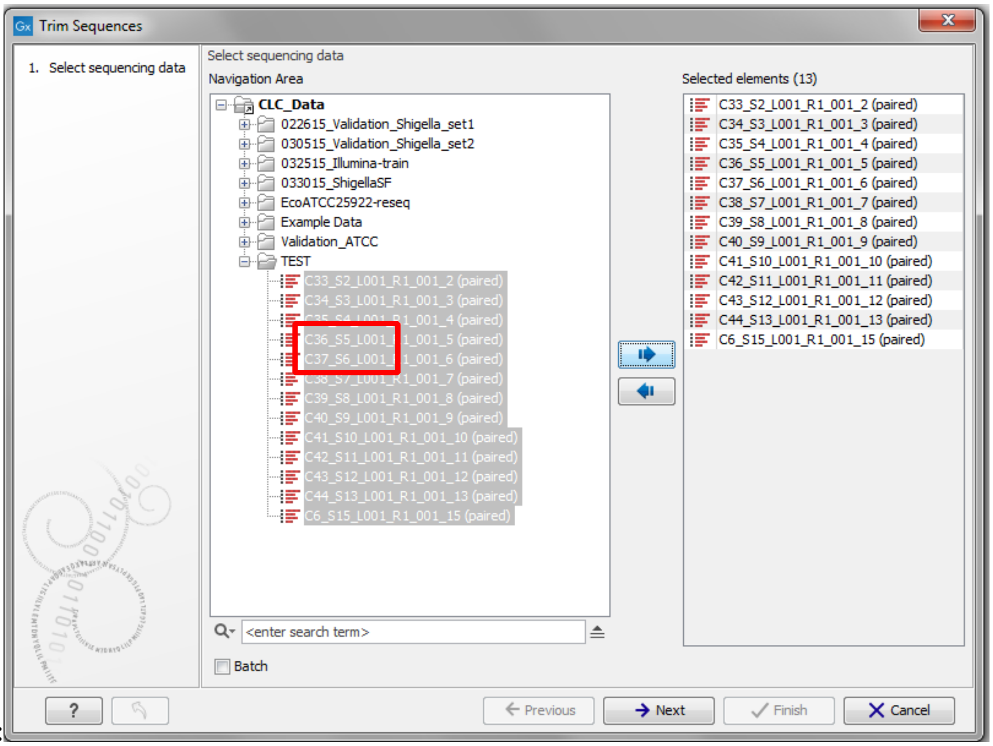

Next

Choose following parameters:

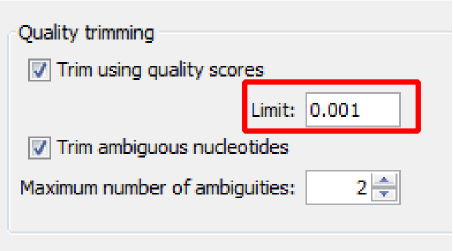

Trim using quality scores with limit 0.001 corresponds to Q>30

Next.

Adapter trimming: Do not perform adapter trimming, it was accomplished by MiSeq primary analysis software.

Next

Discard reads below 40bp:

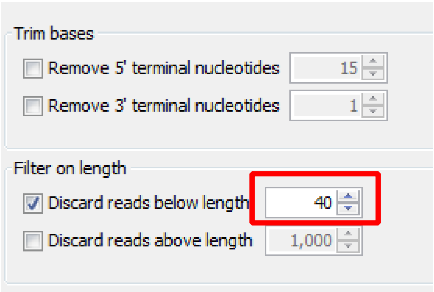

Next

Pick to save trimmed files and to create report:

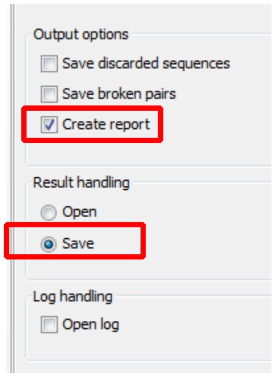

Create new folder inside of the current analysis folder "Trimmed" to save all trimmed reads there:

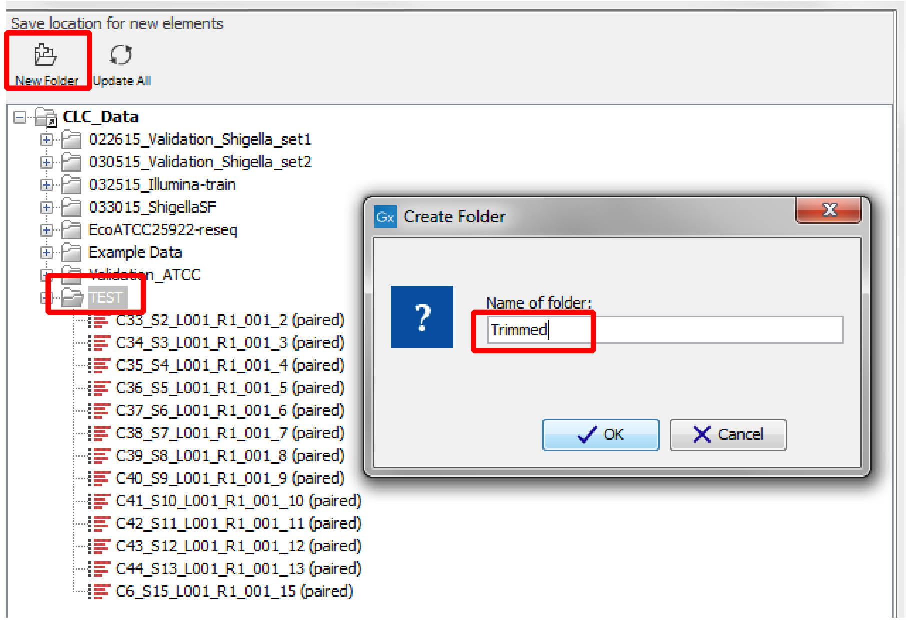

Finish

Reads quality check

Open Trim report by double clicking on the corresponding file in the navigation area. Check average length after trim. It should be > 80bp

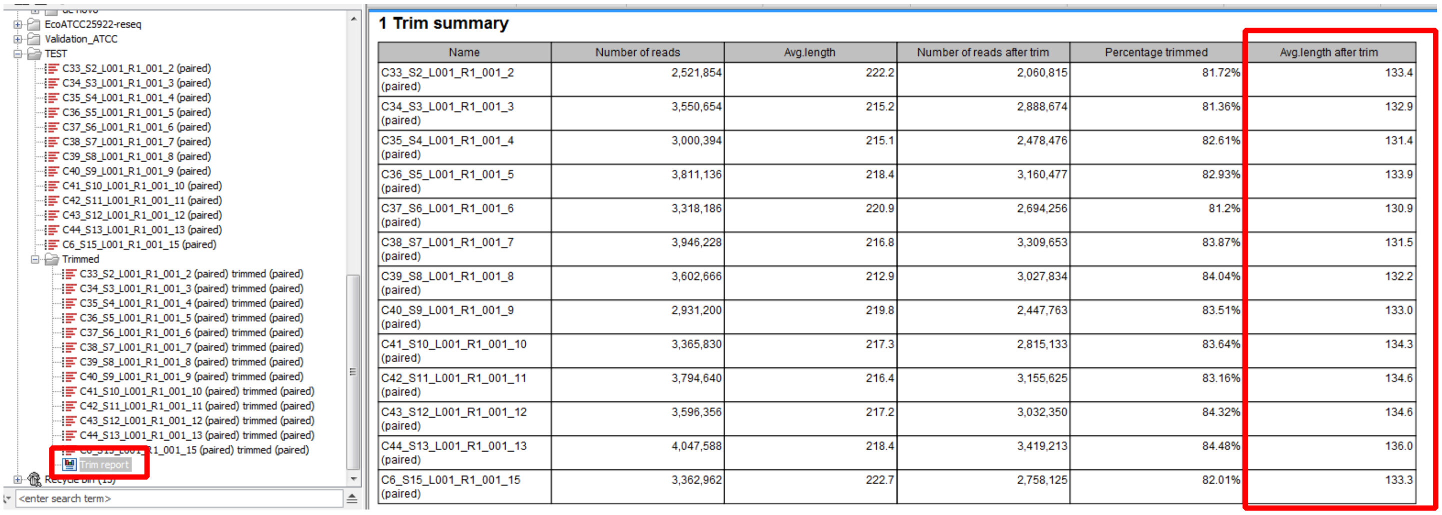

1 Trim summary

Name	Number of reads	Avg.length	Number of reads after trim	Percentage trimmed	Avg.length after trim
C33_S2_L001_R1_001_2 (paired)	2,521,854	222.2	2,060,815	81.72%	133.4
C34_S3_L001_R1_001_3 (paired)	3,550,654	215.2	2,888,674	81.36%	132.9
C35_S4_L001_R1_001_4 (paired)	3,000,394	215.1	2,478,476	82.61%	131.4
C36_S5_L001_R1_001_5 (paired)	3,811,136	218.4	3,160,477	82.93%	133.9
C37_S6_L001_R1_001_6 (paired)	3,318,186	220.9	2,694,256	81.2%	130.9
C38_S7_L001_R1_001_7 (paired)	3,946,228	216.8	3,309,653	83.87%	131.5
C39_S8_L001_R1_001_8 (paired)	3,602,666	212.9	3,027,834	84.04%	132.2
C40_S9_L001_R1_001_9 (paired)	2,931,200	219.8	2,447,763	83.51%	133.0
C41_S10_L001_R1_001_10 (paired)	3,365,830	217.3	2,815,133	83.64%	134.3
C42_S11_L001_R1_001_11 (paired)	3,794,640	216.4	3,155,625	83.16%	134.6
C43_S12_L001_R1_001_12 (paired)	3,596,356	217.2	3,032,350	84.32%	134.6
C44_S13_L001_R1_001_13 (paired)	4,047,588	218.4	3,419,213	84.48%	136.0
C6_S15_L001_R1_001_15 (paired)	3,362,962	222.7	2,758,125	82.01%	133.3

The average coverage after trimming must be >10x. To calculate coverage for each sample= (Avg.length after trim x Number of reads after trim) / genome size in bp

e.g., for the first E.coli sample in the table below coverage= (133.4 x 2,060,815)/ 5,000,000 = 54.98x

1 Trim summary

Name	Number of reads	Avg.length	Number of reads after trim	Percentage trimmed	Avg.length after trim
C33_S2_L001_R1_001_2 (paired)	2,521,854	222.2	2,060,815	81.72%	133.4
C34_S3_L001_R1_001_3 (paired)	3,550,654	215.2	2,888,674	81.36%	132.9
C35_S4_L001_R1_001_4 (paired)	3,000,394	215.1	2,478,476	82.61%	131.4
C36_S5_L001_R1_001_5 (paired)	3,811,136	218.4	3,160,477	82.93%	133.9
C37_S6_L001_R1_001_6 (paired)	3,318,186	220.9	2,694,256	81.2%	130.9
C38_S7_L001_R1_001_7 (paired)	3,946,228	216.8	3,309,653	83.87%	131.5
C39_S8_L001_R1_001_8 (paired)	3,602,666	212.9	3,027,834	84.04%	132.2
C40_S9_L001_R1_001_9 (paired)	2,931,200	219.8	2,447,763	83.51%	133.0
C41_S10_L001_R1_001_10 (paired)	3,365,830	217.3	2,815,133	83.64%	134.3
C42_S11_L001_R1_001_11 (paired)	3,794,640	216.4	3,155,625	83.16%	134.6
C43_S12_L001_R1_001_12 (paired)	3,596,356	217.2	3,032,350	84.32%	134.6
C44_S13_L001_R1_001_13 (paired)	4,047,588	218.4	3,419,213	84.48%	136.0
C6_S15_L001_R1_001_15 (paired)	3,362,962	222.7	2,758,125	82.01%	133.3

Preparing reference sequence for mapping

Using NCBI genome as a reference

Download reference sequence for mapping

Create folder “References” inside of the current analysis folder as described previously.

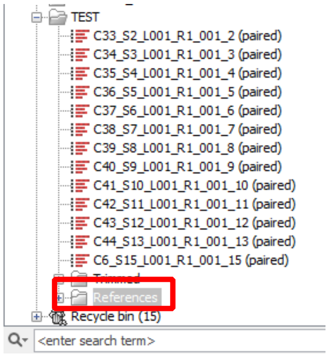

Import references from fasta file saved on PC or download from NCBI (preferably).

Import through NCBI in CLC (preferable way):

Select Download at the top of the screen →Search for Sequences at NCBI

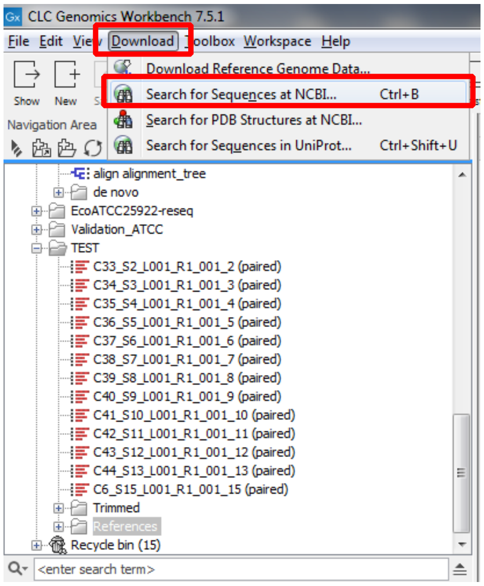

In newly opened window type GenBank accession number. In order to get accession number look for available genomes for the species of interest on the site http://www.ncbi.nlm.nih.gov/genome/ .

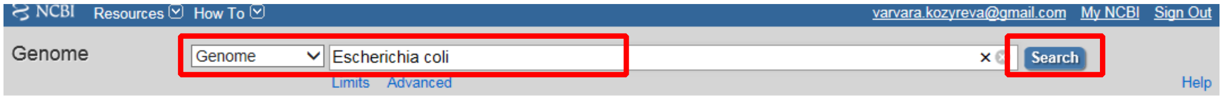

Click on a link “Genome Assembly and Annotation report”:

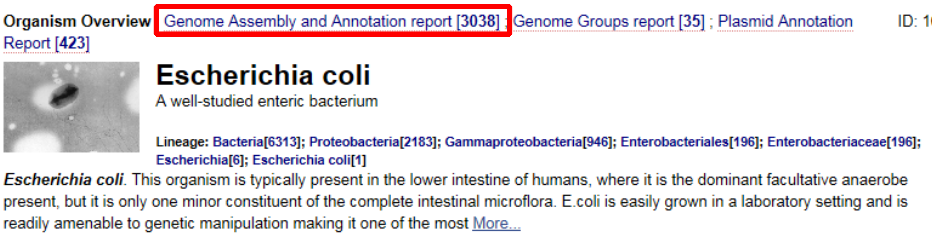

Sort genomes by the level of completion:

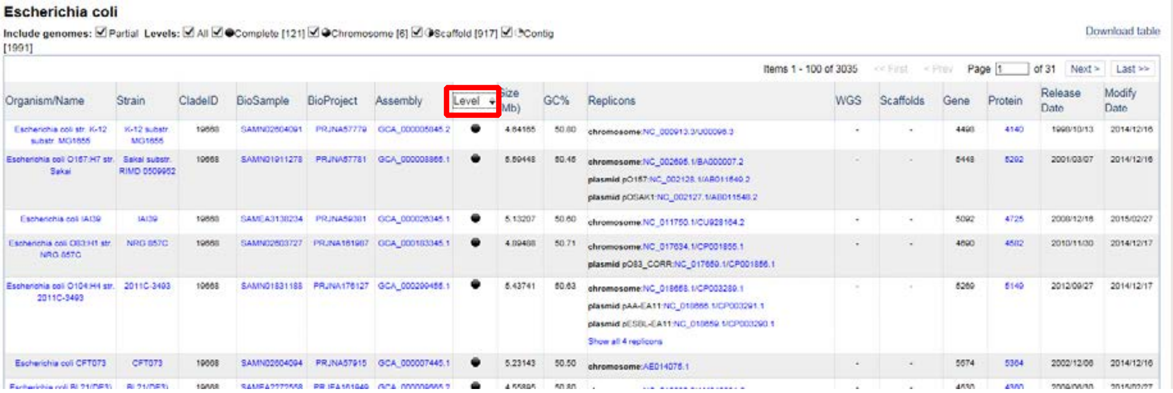

Find suitable reference on NCBI/ Genome site: most preferable to use as the reference is the full genome sequence. Alternatively it is possible to use scaffold sequence. Try to find well annotated genome. Write down accession number for the genome which you would like to use as a reference.

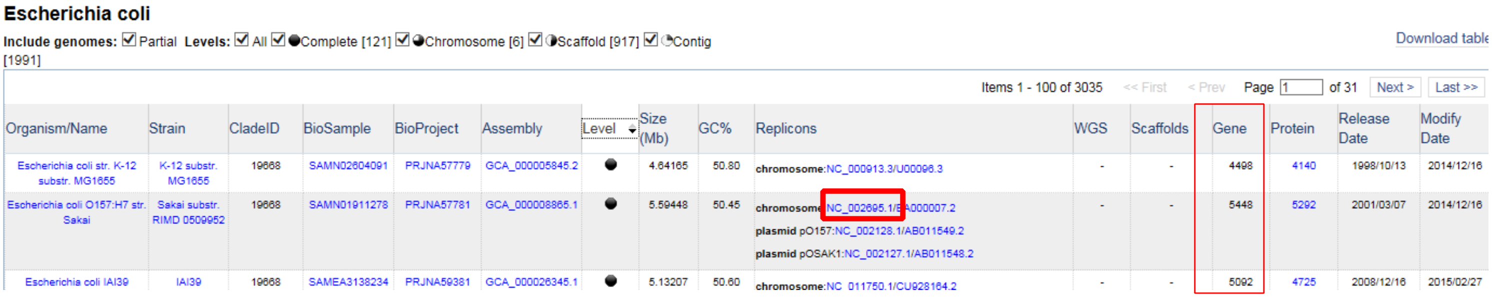

Use it in search in NCBI CLC tool: Type accession number in the field "all fields". Click button "Start search":

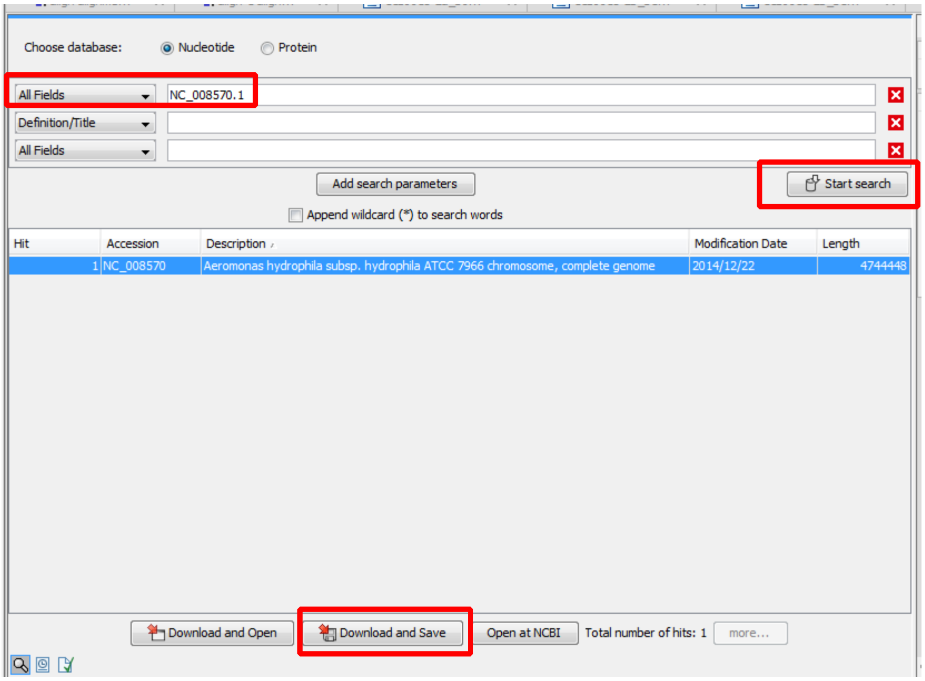

Pick the most suitable hit if more than one available. Click button "Download and save". Chose to save in "References" folder:

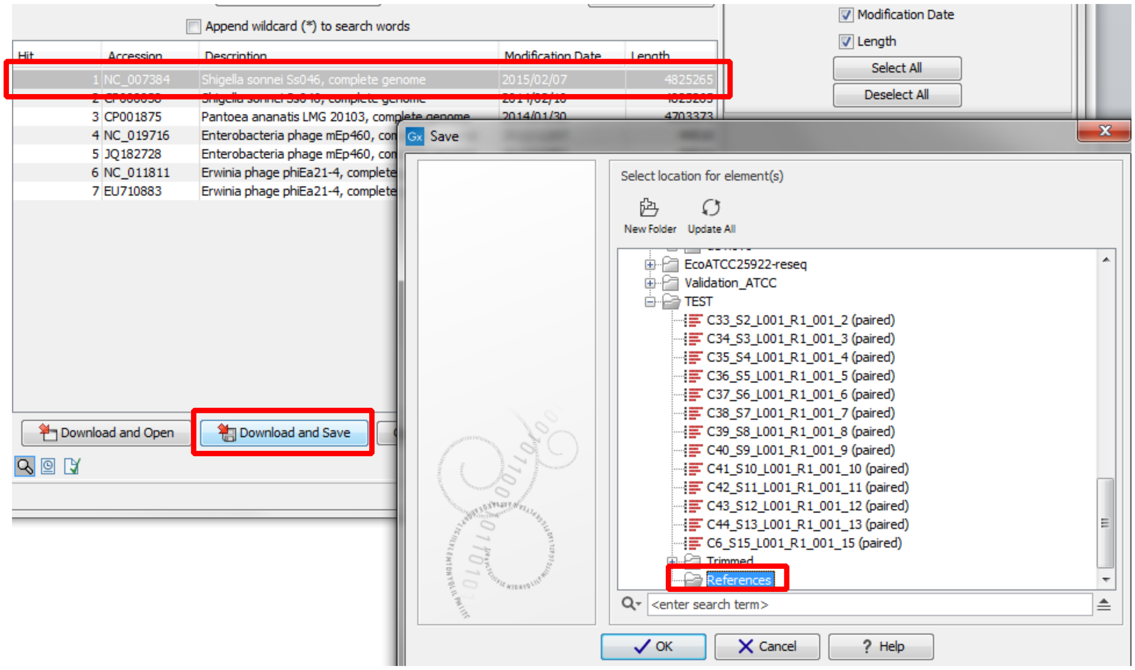

Alternatively:

Import fasta files with reference sequences: Import → Standard import:

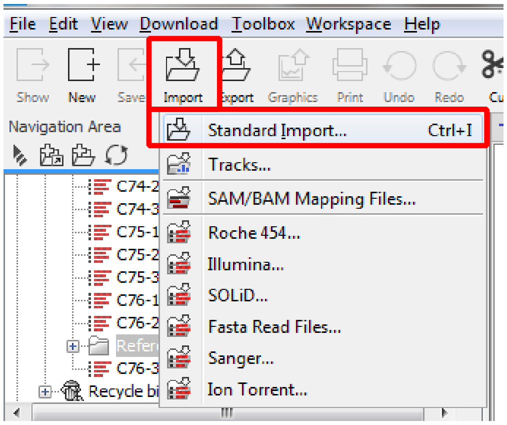

Pick one or more reference files stored on computer:

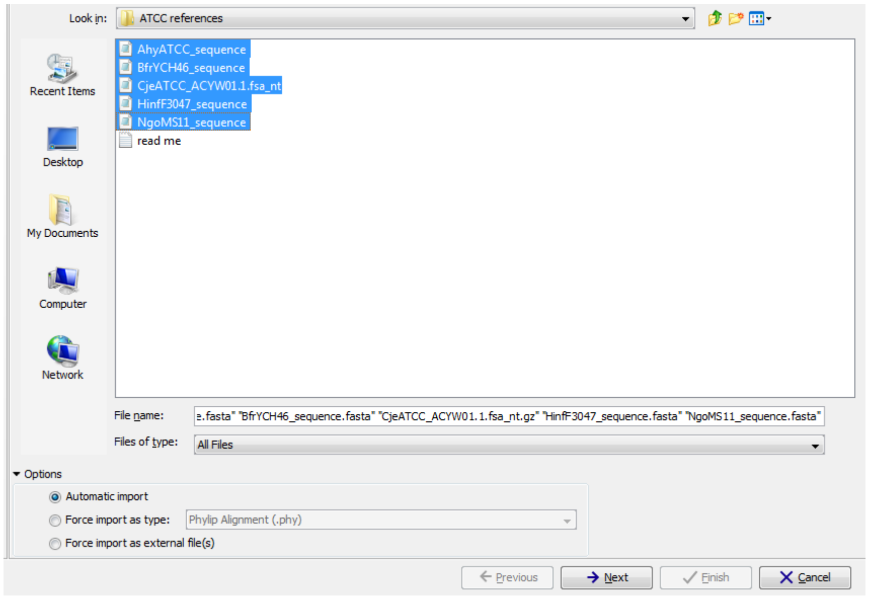

Chose to import into the new folder "References":

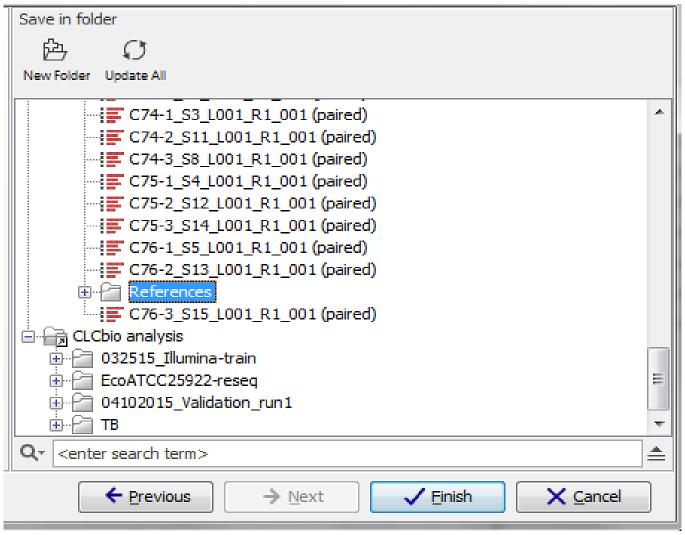

Reference can be imported from whole genome or from scaffold:

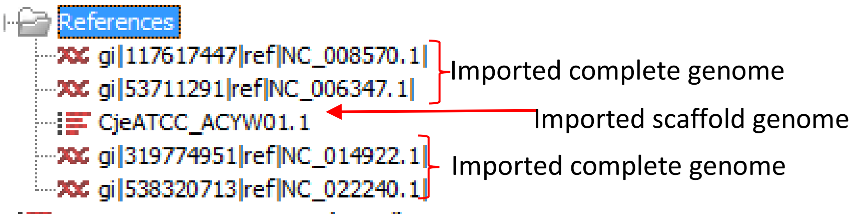

Scaffold might have several contigs. Concatenate contigs in scaffold genome as described below in chapter "Using one of the outbreak strains genome as a reference"

For validation of sequencing results agains CDC sequences, raw reads were downloaded from SRA NCBI site: http://www.ncbi.nlm.nih.gov/sra/

SAMN numbers provided by CDC were used to locate reads corresponding to the Salmonella and E.coli samples stored in MDL collection.

Command-line based SRA tools software was used to generate paired-end reads from SRA file:

```
Fastq-dump -I --split-files C74_M14X00933_Infantis.sra >
C74_M14X00933_Infantis.log 2>&1
```

Generated fastq files were compressed to match input Illumina reads using following command:

```
gzip -v *.fastq
```

Import CDC fastq.gz file to CLCbio using Illumina import.

CDC reads were trimmed and mapped alongside with Corelab sequences to the alternative complete genome of the same species/serotype. Alternative complete genome was downloaded from NCBI as described above.

Important: Make sure that name of the reference file in CLCbio doesn't contain spaces or any special symbols, e.g. !,.-@#$%^()&*[ ]. Use underscore (_) to separate words if necesasary.

Using one of the outbreak strains genome as a reference

When no good reference is available use genome of one of the tested samples as a reference for mapping of the other samples. It is also recommended to perform mapping to one of the outbreak samples in parallel with mapping to NCBI reference to compare results of two methods.

Perform *De novo* assembly of the original trimmed reads of one of the tested isolates- as described in chapter "*De novo* assembly"

Then proceed to annotation of assembled genome as described in chapter "Annotation with PROKKA"

Export annotated .gbk file into the CLCbio

Concatenate annotated contigs: Toolbox → Classical Sequencing Analysis → General Sequencing Analysis → Join Sequences

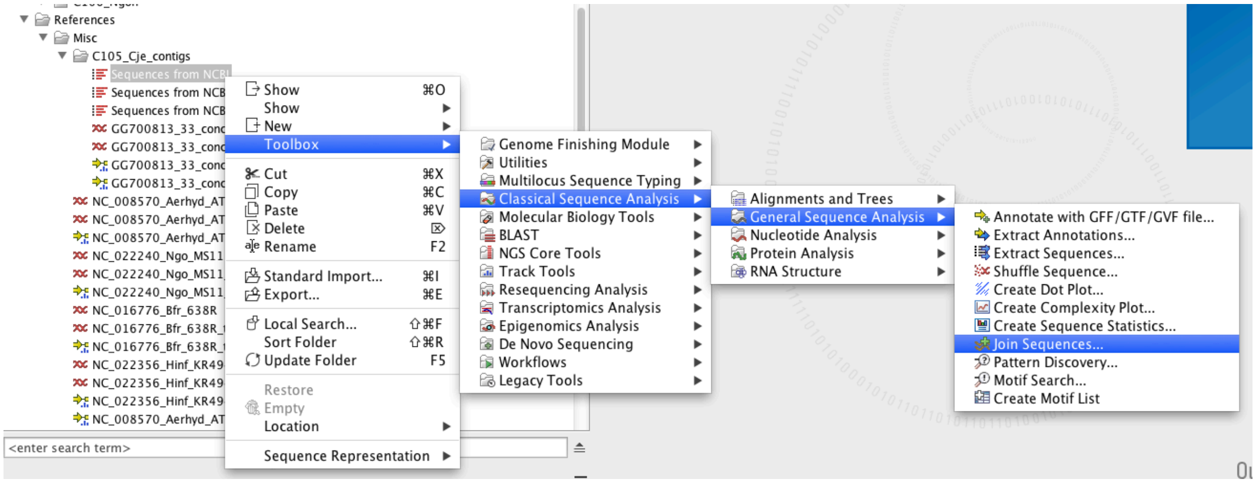

Select one or more files which you would like to join:

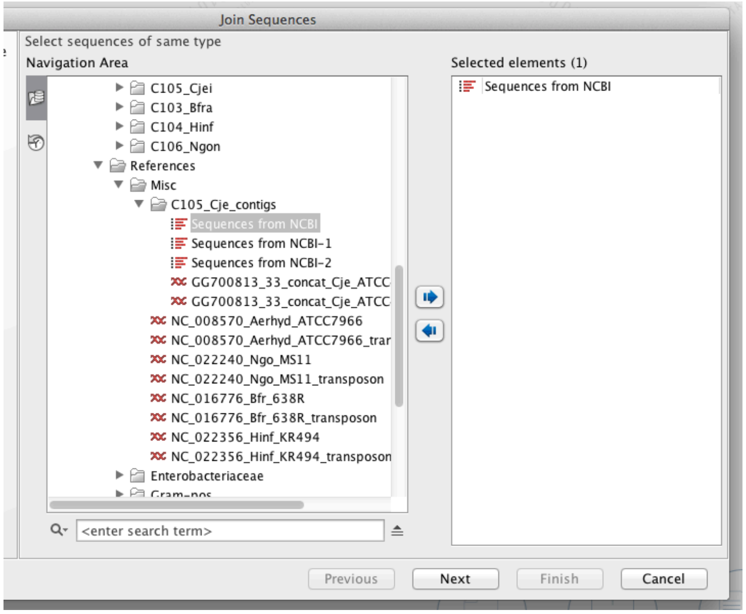

Leave all contigs in the file

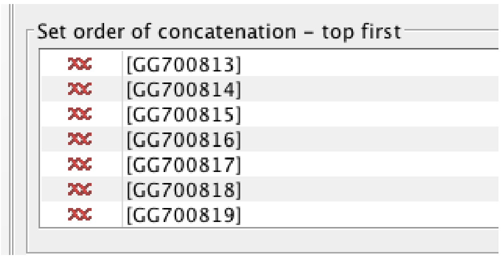

Save

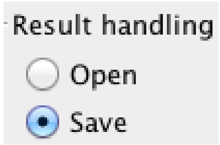

Important: Make sure that name of the reference file in CLCbio doesn't contain spaces or any special symbols, e.g. !,.-@#$%^()&*[ ]. Use underscore (_) to separate words if necesasary.

Use concatenated sequence to mask mobile elements and for mapping as described below.

Mapping

Mobile elements masking

Mobile elements can be masked from the reference genome in order not to interfere with phylogenetic tree. Masking is optional.

Open annotated reference genome file in CLCbio by double clicking on the name. In left bottom corner choose table view.

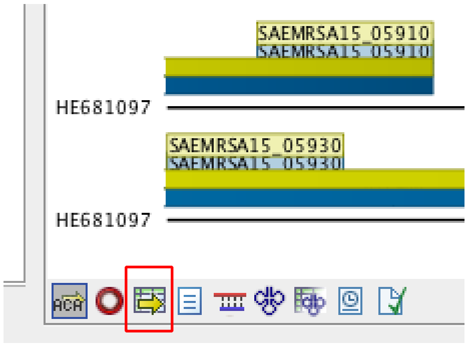

In search field of opened table with annotations input type of the annotation to sort for it. E.g. annotations containing word "phage" or "transposase":

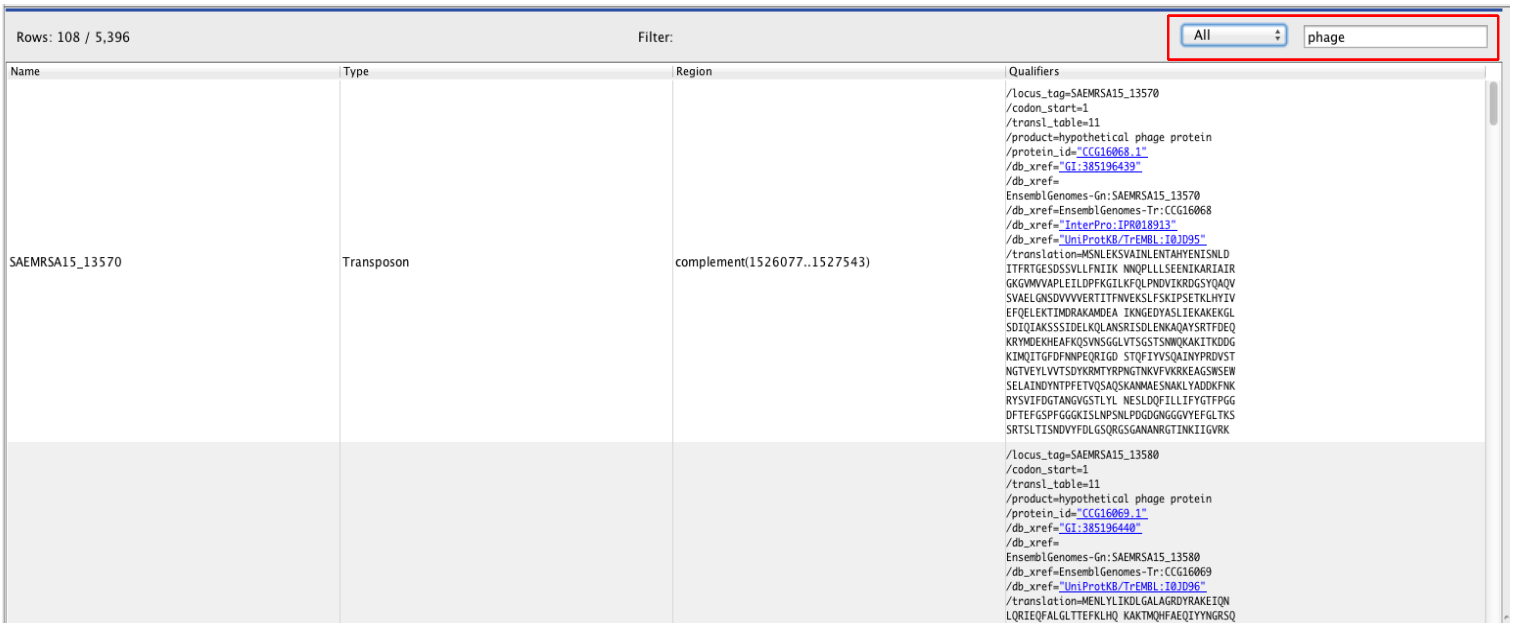

Press Enter

Make sure that in sorted table all entries in the column "Qualifiers" for the "product" have a mobile element.

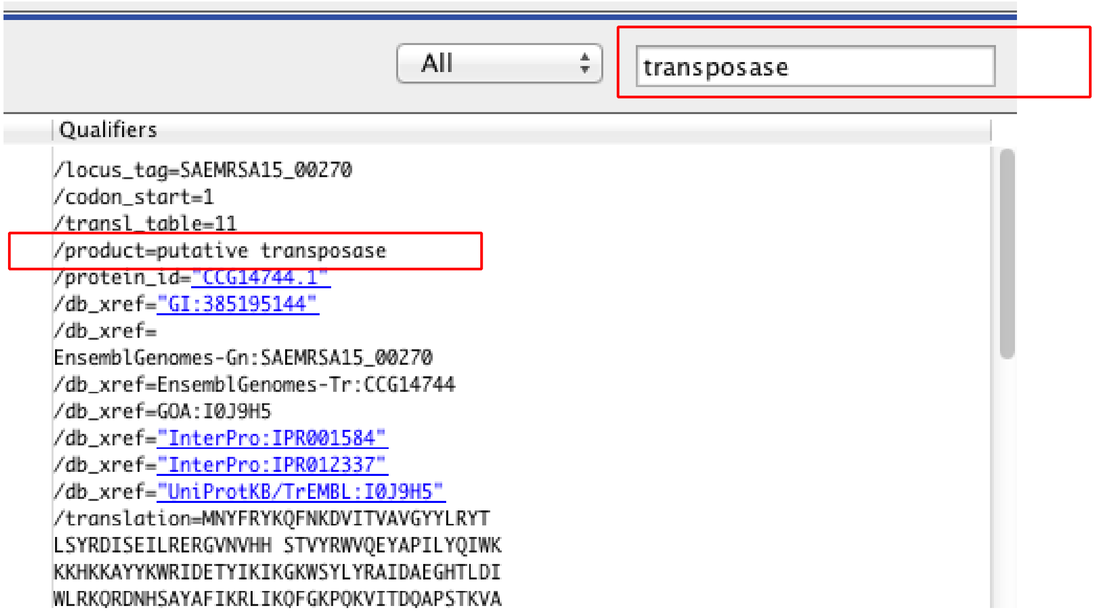

Select all entries annotated as mobile elements.

By left click with mouse open menu. Pick “Advanced Retype…”

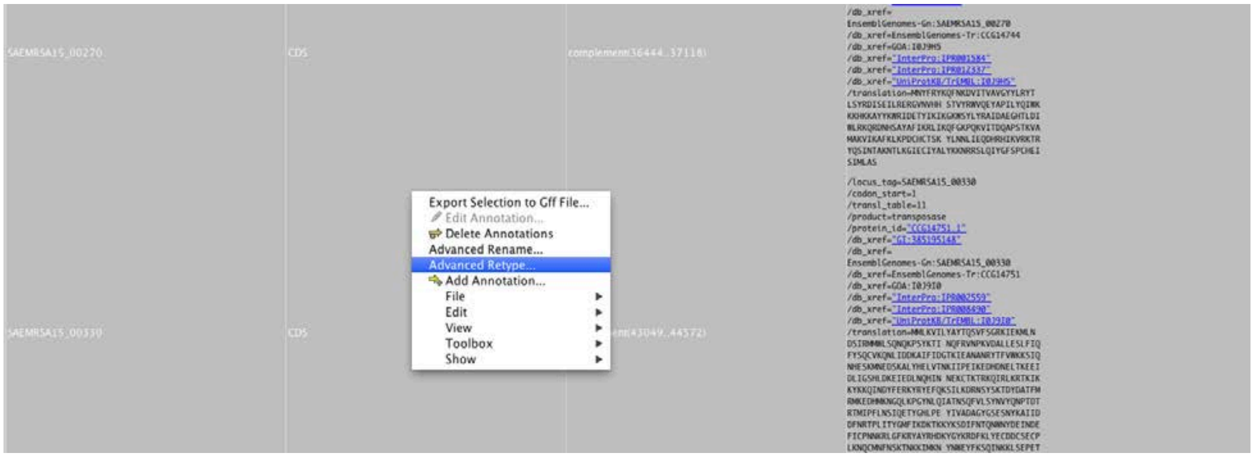

Choose “New type”. From drop down list pick “Transposon” (for all types of mobile elements)

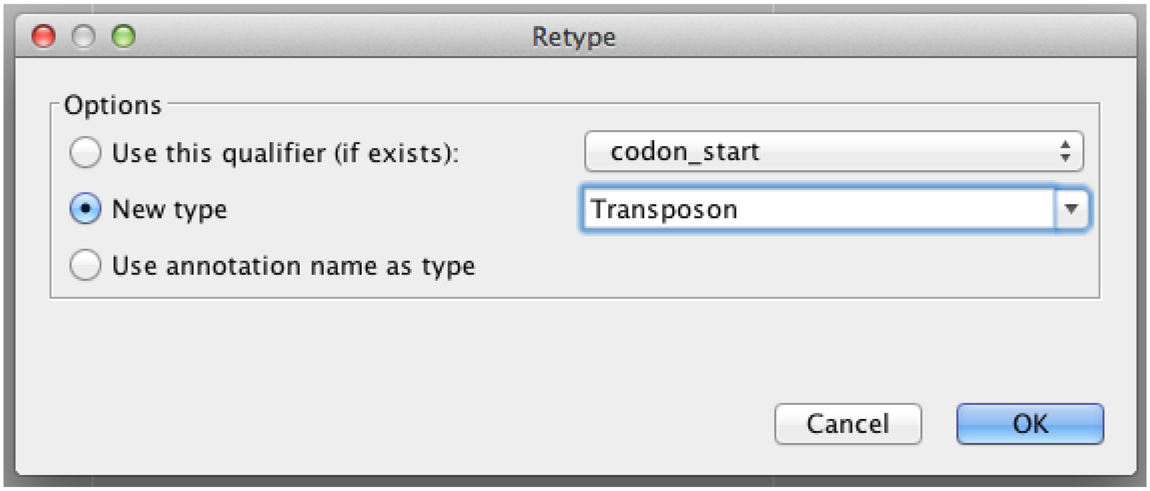

Repeat the search and reassigning of the annotation type for other mobile elements. Perform search for following words: “bacteriophage”, “phage”, “transposon”, “transposase”, “resolvase”, “integron”, “integrase”, “insertion”, “IS “, “mobile”, “element”, “island”. Every time double check that that non-related to mobile elements annotation are not selected. E.g. annotation containing word “macrophage” shouldn’t be included into the selection upon search for the word “phage”.

When renamed all annotations containing mobile elements from “CDS” to “Transposon”, save new reference with modified annotations with modified file name by adding “_Transposon” in the end of the name.

Convert reference file with added Transposon annotations to tracks:

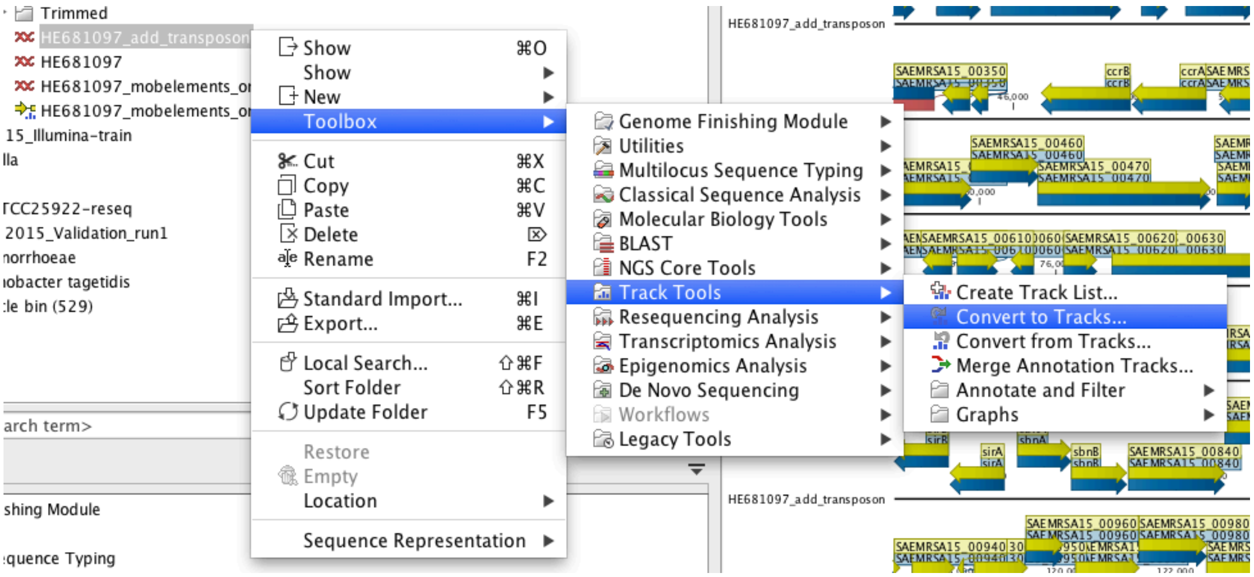

Create annotation tracks only for annotation type “Transposon”. To select annotation type click on green plus sign:

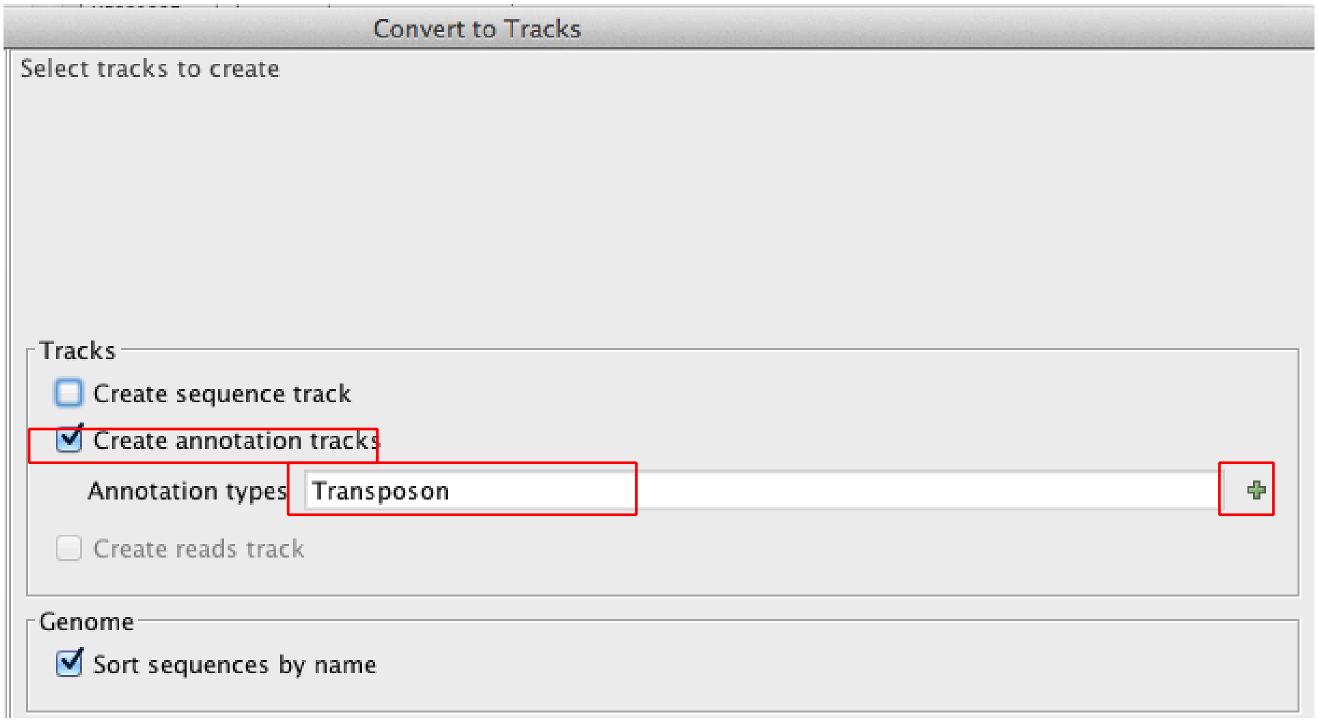

Save annotation track with “Transposon” annotations:

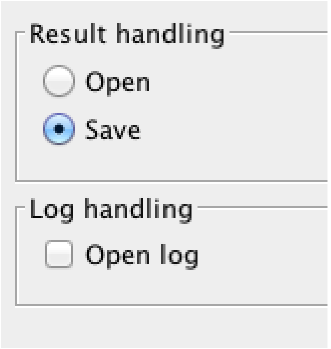

Mapping

Select trimmed reads to use for mapping to the corresponding reference sequence: Toolbox →NGS Core Tools → Map Reads to Reference…

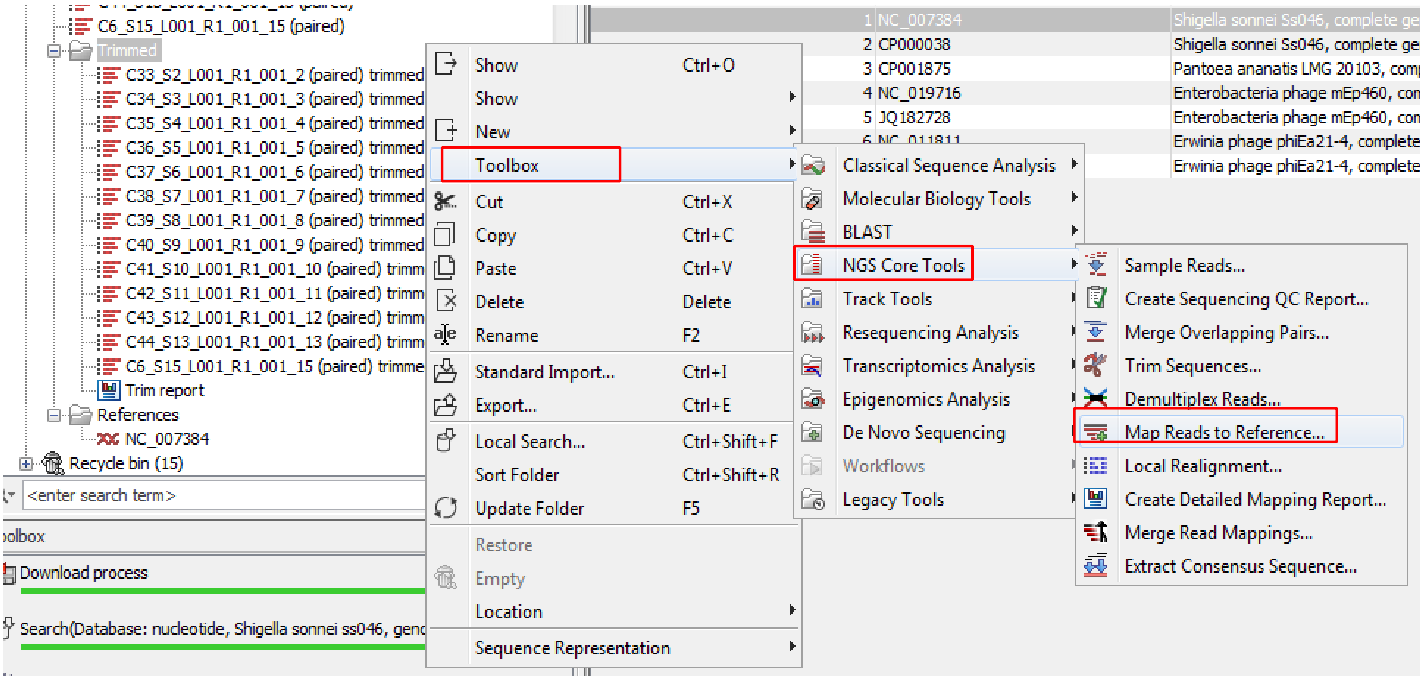

Select the whole folder “Trimmed”. Select “Batch” on the bottom of the window and add the folder to the “Selected elements” by clicking on right arrow. Batch only sequences which belong to the same species (so they will be mapped to the same reference sequence).

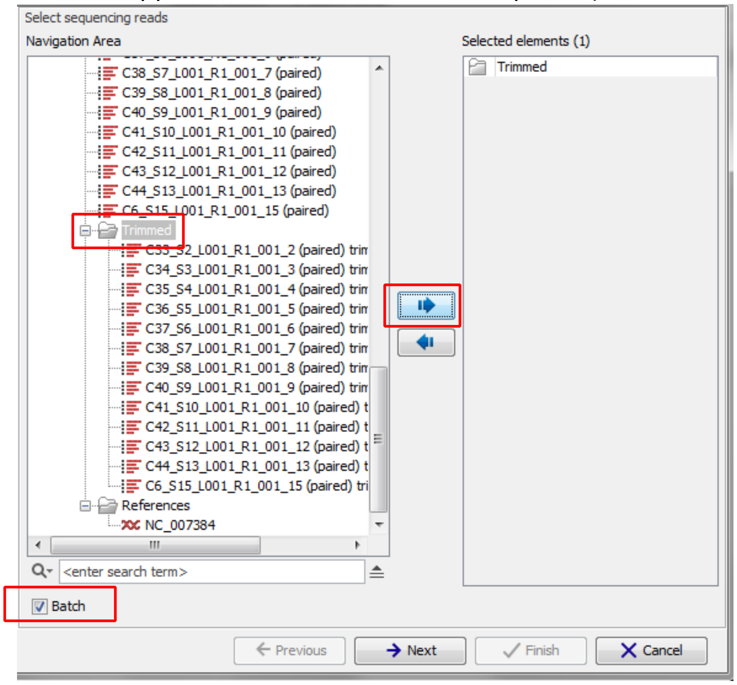

[Alternatively undo “Batch” mark and select file(s) from the folder “Trimmed” for analysis one by one if have to map to different references. If several reads are selected together without batching these reads will be combined into the single mapping file]:

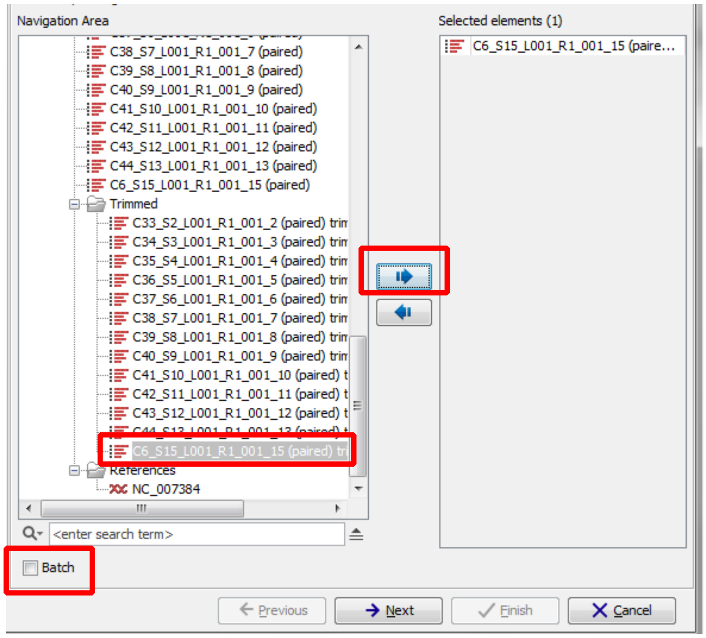

If using batching mode proceed to selecting files from the folder “Trimmed” for analysis by removing from the list files which you do not desire to map. For the right click with the mouse on the selected samples which you want to remove from the analyses and pick “Remove Batch Unit”. All remaining in the list files will be mapped to the same reference sequence.

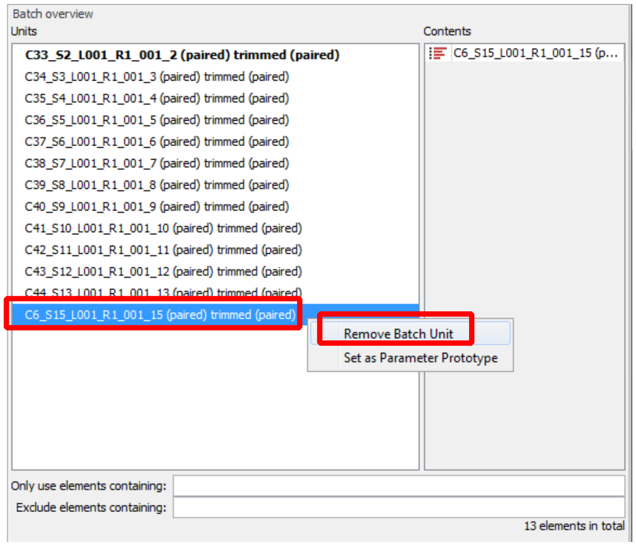

For validation map all replicates of one samples to the same reference in a batch. Reference in this validation study can be represented by:

1) *Complete genome of the same ATCC strain which was sequenced by the Core lab. Used when genome of the strain sequenced by Core lab is available from NCBI database.*

2) *Complete genome of the strain belonging to the same species as tested by Core lab isolates, however not identical to the isolate sequenced by the Core lab. Used when genome of the strain sequenced by Core lab is NOT available from NCBI database.*

3) *Raw reads generated by CDC for the same strains which were also sequenced in Core lab.*

In case 1 and 2 reference is downloaded from NCBI Genome database and used as a reference for mapping of the Core lab- generated sequences. SNP difference between Core lab replicates is shown in both cases. SNP difference between reference sequence and Core lab sequences is estimated only for the case 1.

In case 3 reference raw reads are trimmed and mapped alongside with Corelab sequences to the alternative complete genome of the same species/serotype to show number of SNP difference between Core lab replicates and CDC-generated sequences.

Next

Change previously used reference to a new one by clicking on browse button:

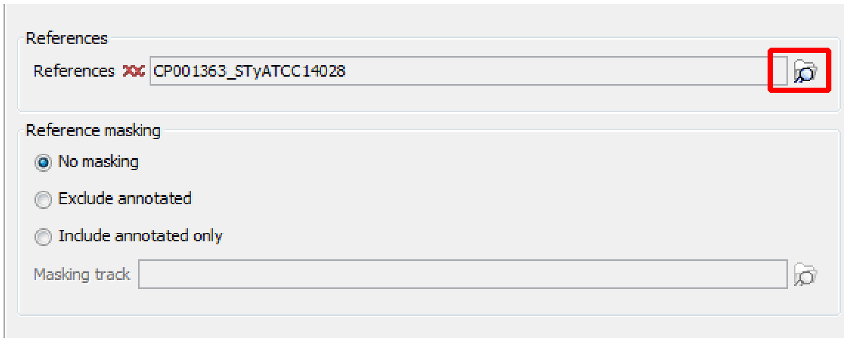

First remove previous reference sequence by clicking on it and using arrow to the left

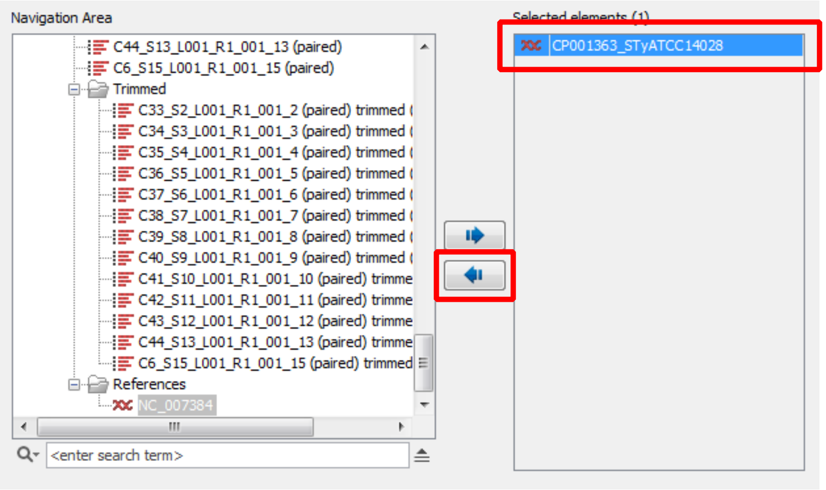

Then select sequence from the folder “References” and add it to the “Selected elements”:

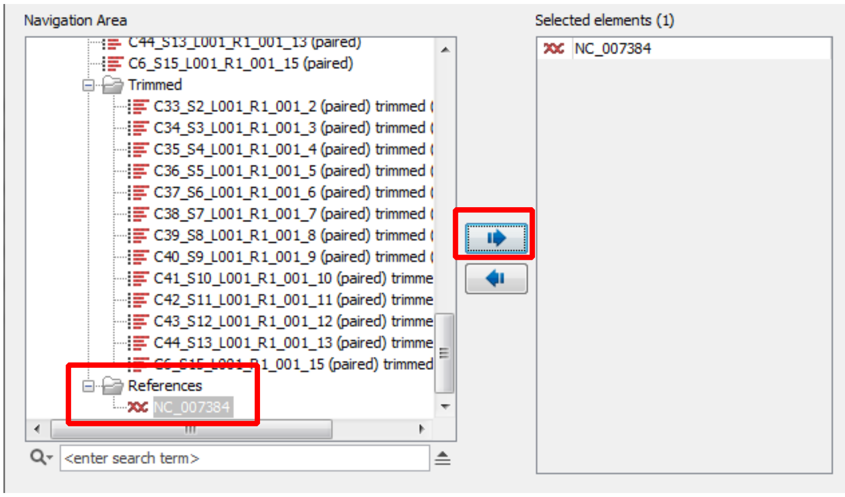

Next

Choose to “Exclude annotated” and pick the “Masking track” from track containing only “Transposon” annotations which was created above.

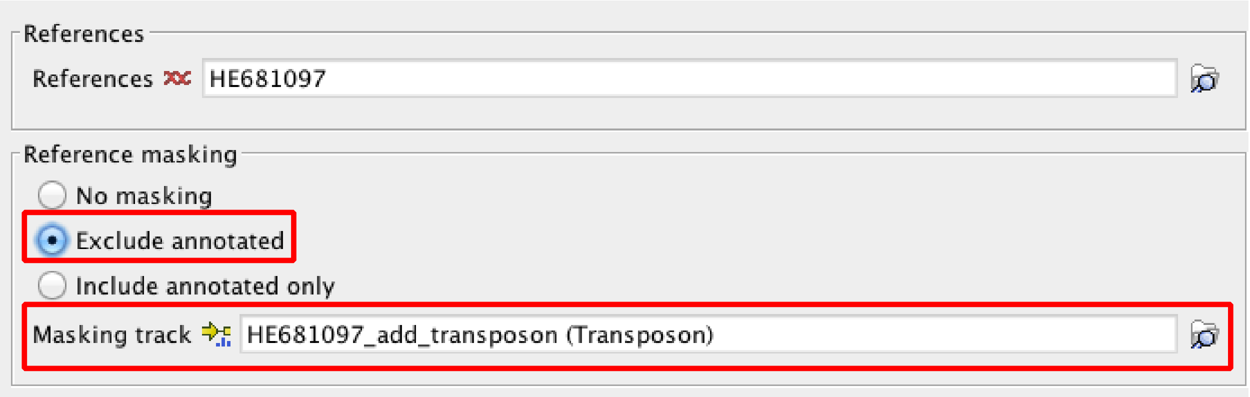

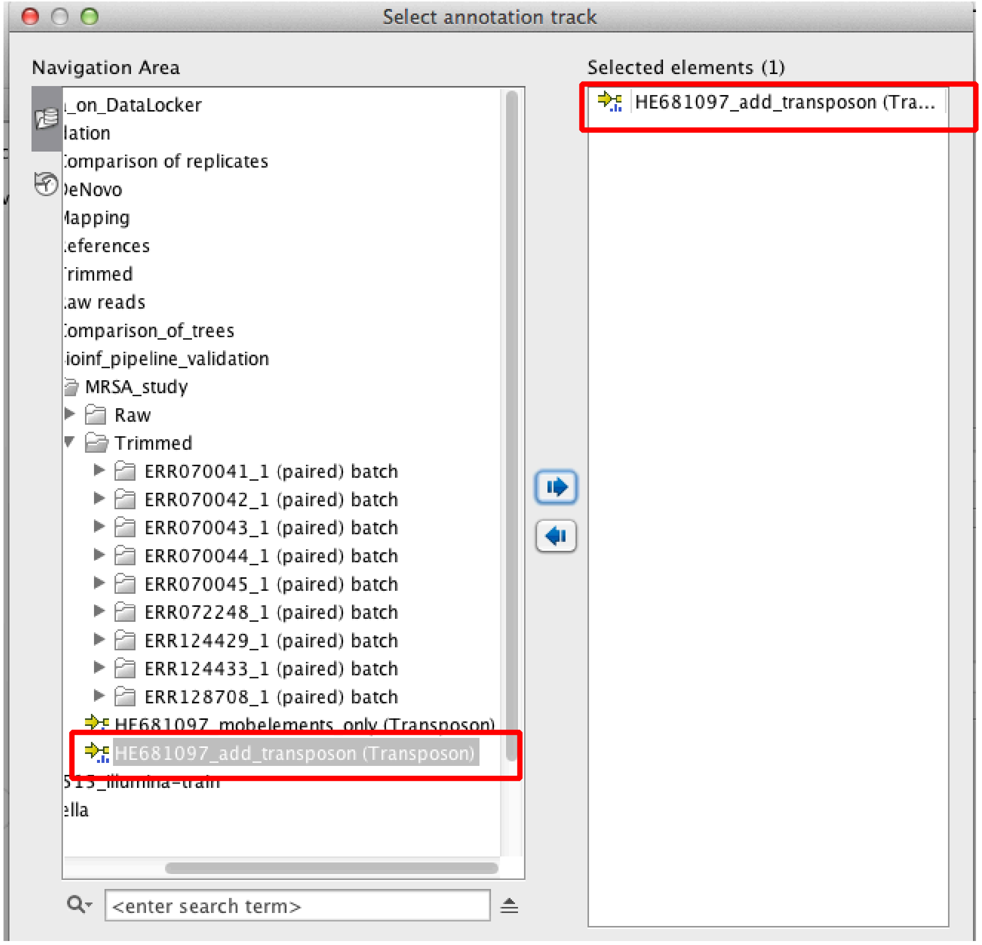

Select annotation track
Navigation Area
Selected elements (1)
HE681097_add_transposon (Tra...
_on_DataLocker
lation
omparison of replicates
eNovo
apping
eferences
rimmed
aw reads
omparison_of_trees
ioinf_pipeline_validation
MRSA_study
Raw
Trimmed
ERR070041_1 (paired) batch
ERR070042_1 (paired) batch
ERR070043_1 (paired) batch
ERR070044_1 (paired) batch
ERR070045_1 (paired) batch
ERR072248_1 (paired) batch
ERR124429_1 (paired) batch
ERR124433_1 (paired) batch
ERR128708_1 (paired) batch
HE681097_mobelements_only (Transposon)
HE681097_add_transposon (Transposon)
ella
<enter search term>

Use following default settings:

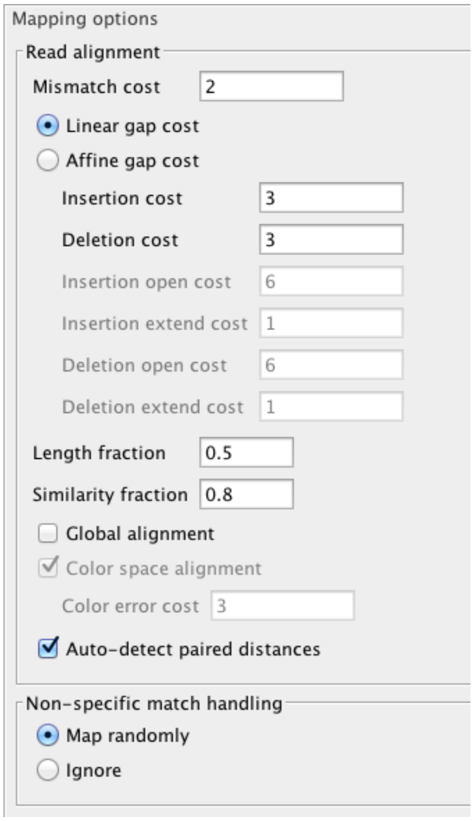

Next

Chose to Save mappings into separate folders, create report and to collect un-mapped reads:

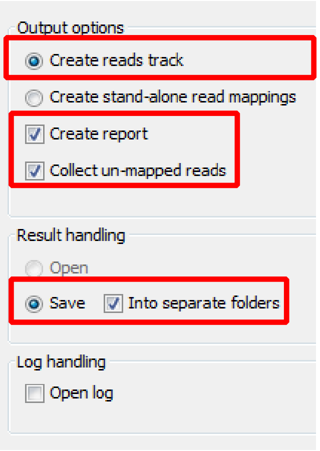

Create folder "Mapping" inside of the current analysis folder:

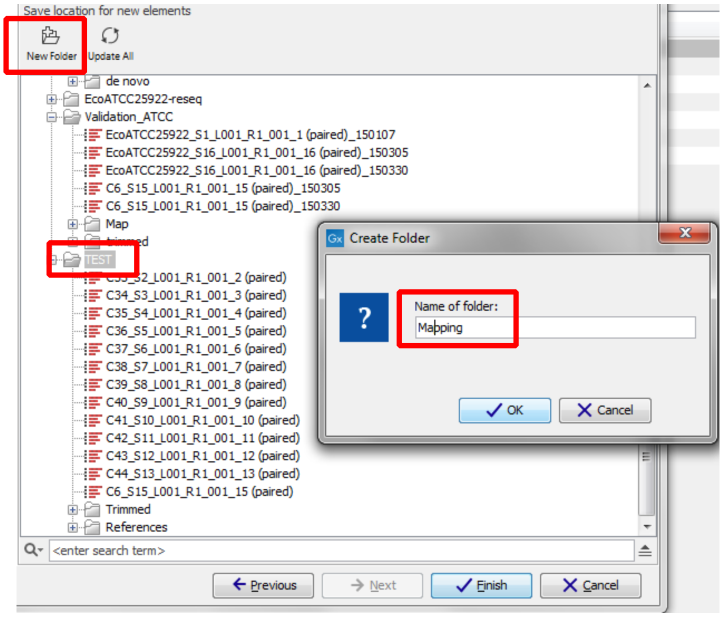

Finish

Open created mapping to check if no mapping was performed in the areas corresponding to mobile elements:

Open mapping file. Click on button "Create Track List"

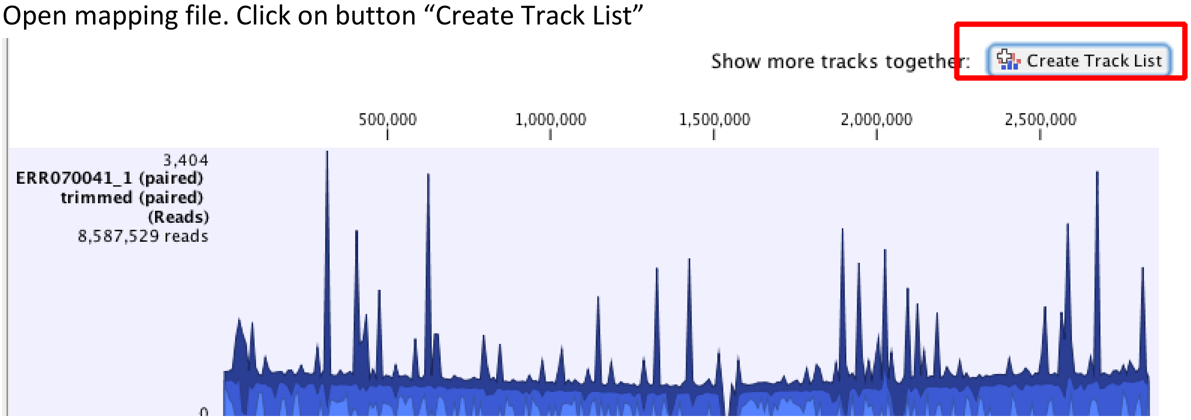

Add annotation track containing only "Transposon" annotations created previously:

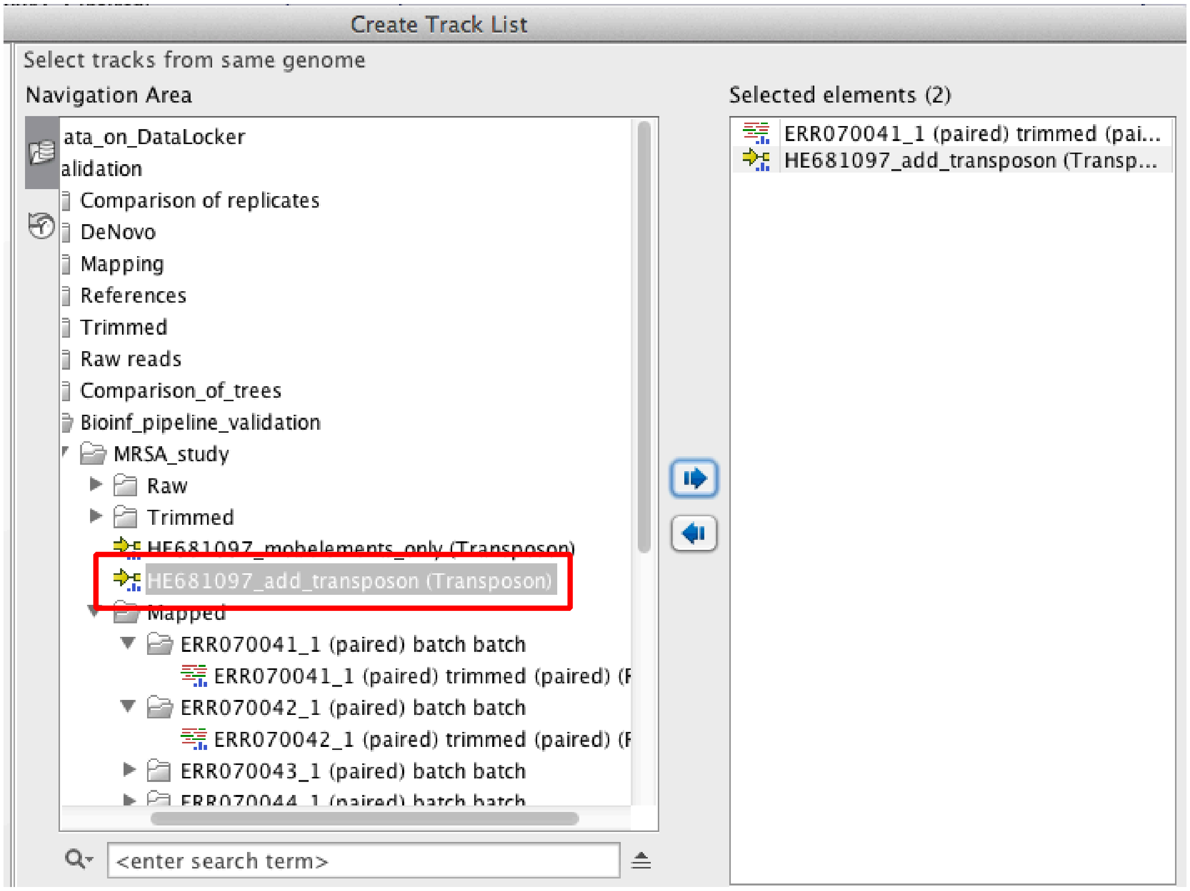

Zoom on one of the areas marked by red bar, e.g.:

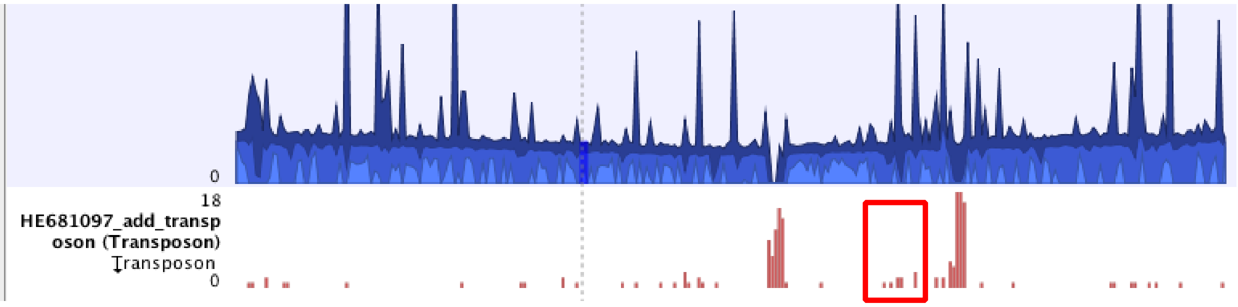

Zoom until arrow view will appear. Hover over the arrow to see the type of annotation. No mapping in the area of the annotation must be seen:

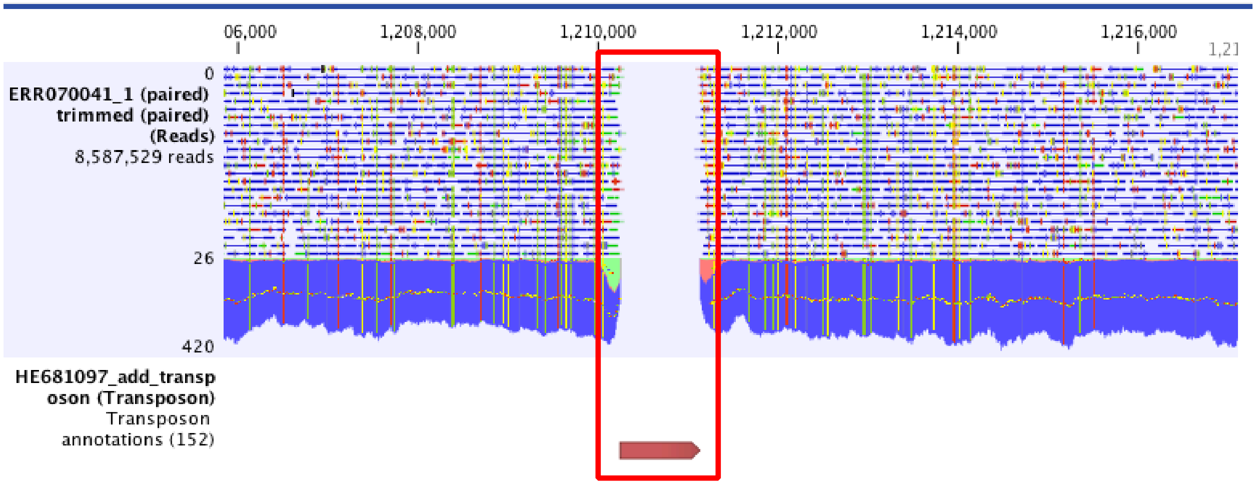

Detailed mapping report generation

Create a detailed report of mapping when it is done:

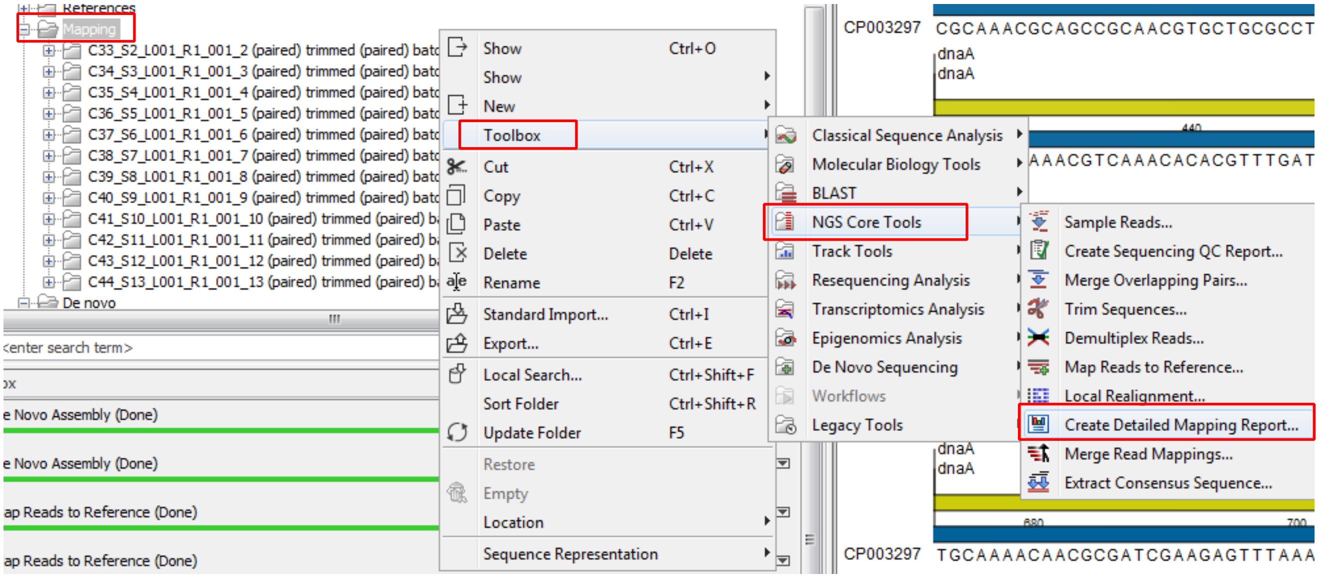

Do batch analysis of the folder "Mapping":

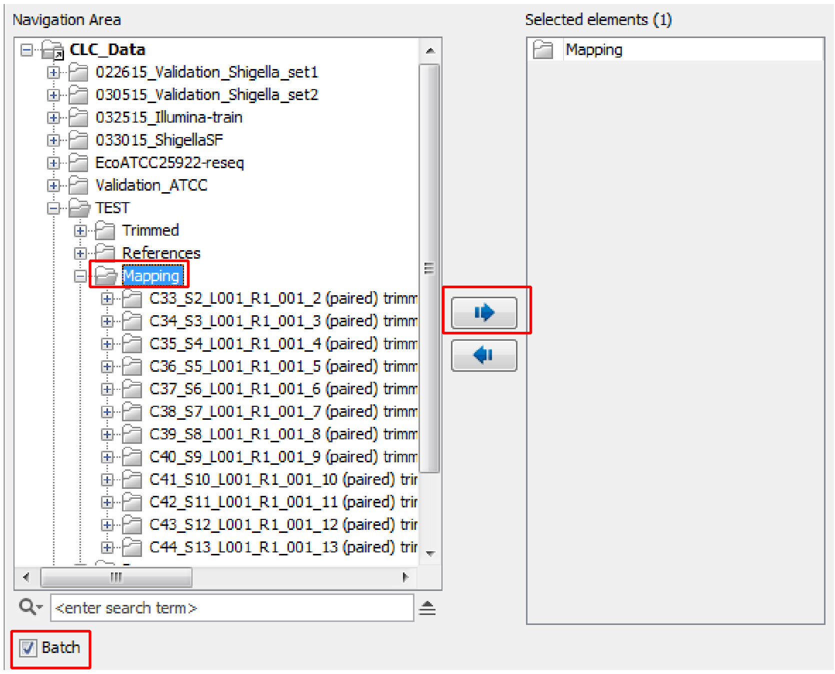

Leave all mappings in the analysis list:

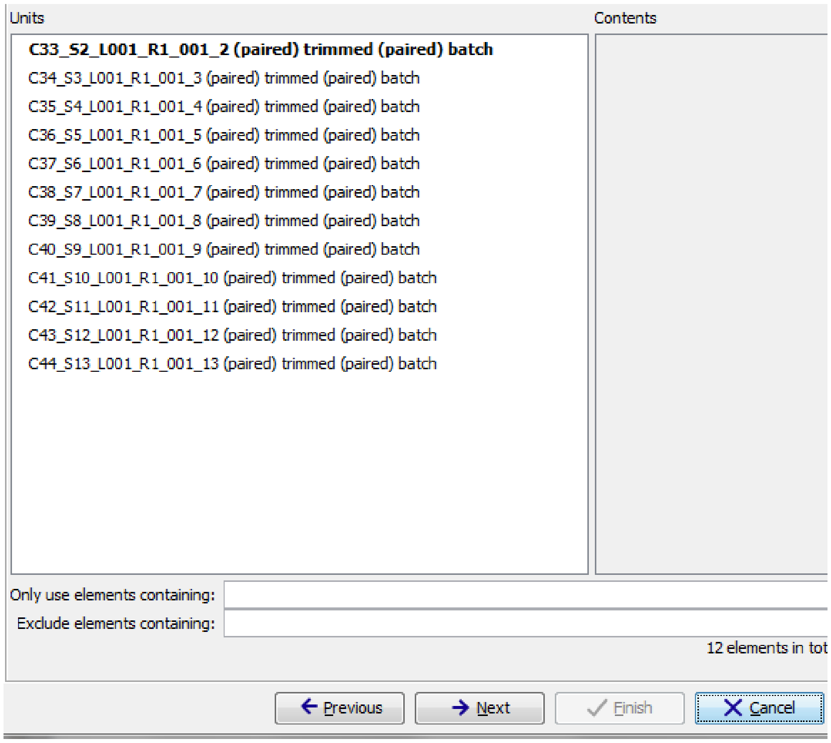

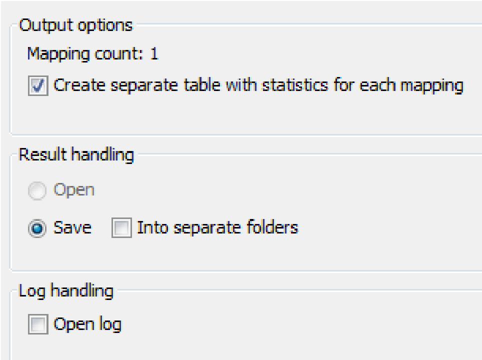

Finish

Open detailed mapping report for each sample one by one. Notice following metrics: mean read length should be >80bp, fraction of reference covered should be > 0.9, average coverage is preferable to be > 20x:

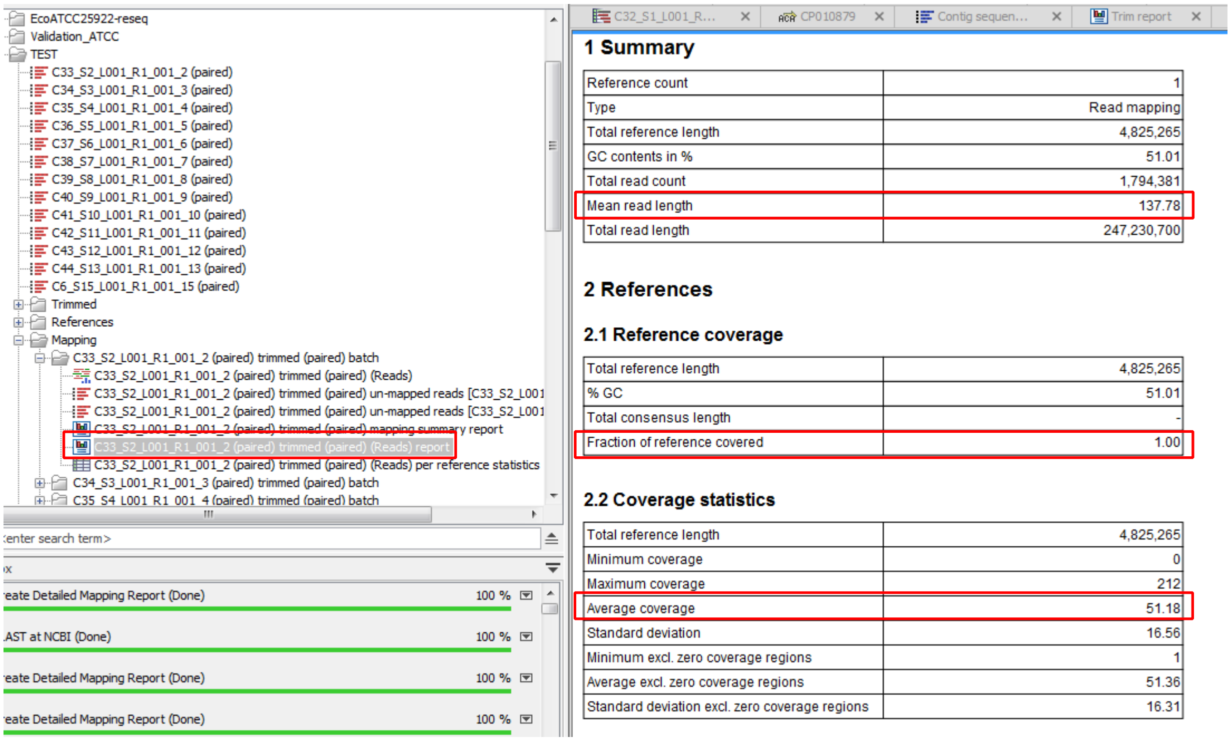

Export of mapping files

Export created maps for all samples one by one as .bam files:

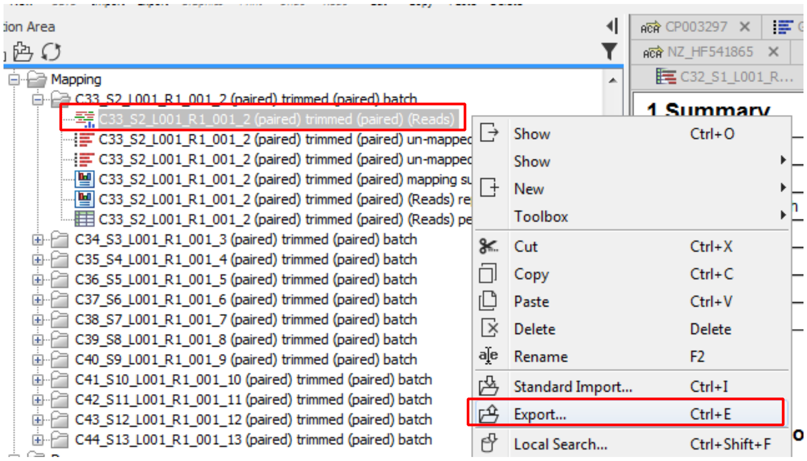

Pick BAM format for export:

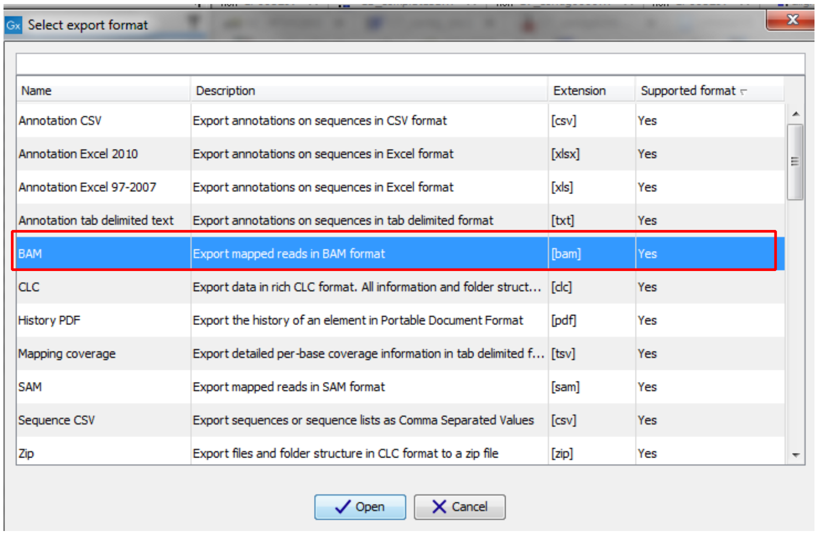

Export reference file which was used for the mapping in fasta format:

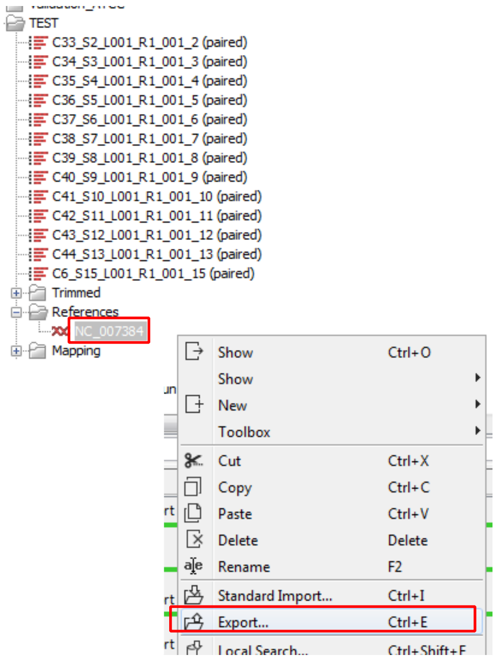

Pick FASTA format for export:

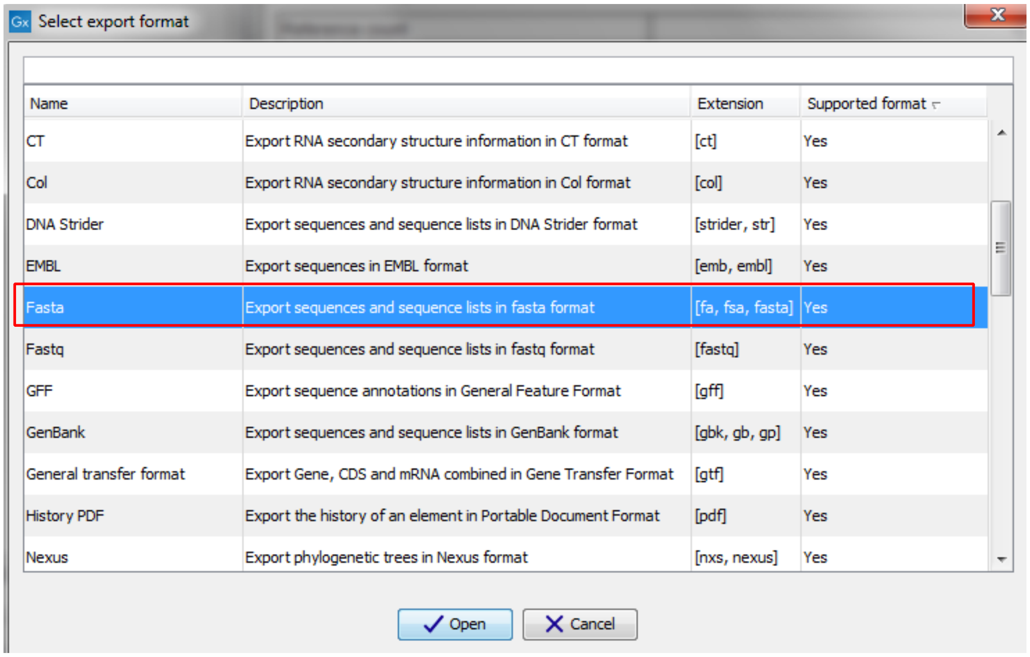

Use exported bam files to created sorted bam files and then use in samtools mpileup. Use exported reference fasta file as the reference in mpileup.

Also export Annotations from the Reference file, for that click on reference file with right mouse button →Export → GFF- Export sequence annotations in General Feature Format:

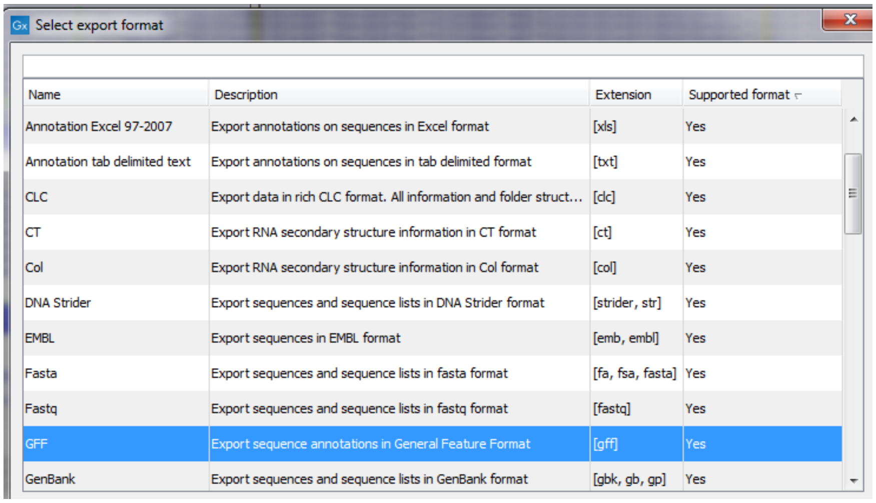
Select export format

Name	Description	Extension	Supported format
Annotation Excel 97-2007	Export annotations on sequences in Excel format	[xls]	Yes
Annotation tab delimited text	Export annotations on sequences in tab delimited format	[txt]	Yes
CLC	Export data in rich CLC format. All information and folder struct...	[clc]	Yes
CT	Export RNA secondary structure information in CT format	[ct]	Yes
Col	Export RNA secondary structure information in Col format	[col]	Yes
DNA Strider	Export sequences and sequence lists in DNA Strider format	[strider, str]	Yes
EMBL	Export sequences in EMBL format	[emb, embl]	Yes
Fasta	Export sequences and sequence lists in fasta format	[fa, fsa, fasta]	Yes
Fastq	Export sequences and sequence lists in fastq format	[fastq]	Yes
GFF	Export sequence annotations in General Feature Format	[gff]	Yes
GenBank	Export sequences and sequence lists in GenBank format	[gbk, gb, gp]	Yes

Sorting of exported bam files

Transfer the bam files and reference files to a folder in MacPro using Finder. Make sure that names of files and folders don't contain any spaces. Open Terminal on the MacPro and go into the directory where your exported your bam files.

Use following commands to navigate:

pwd – show the current directory

`ls –lrt` - show all files and folders in the current directory

`cd ../` -go outside of the current directory one folder up

`cd ./Name_of_Folder_here` - pick the folder inside of the current directory as a new location

Run the following command in Terminal for each exported bam file to create a sorted bam file. In this example, "C50.bam" is the name of the exported bam file. Here and further parts of a command which need to be changed according to the sample name and file location highlighted with green. Depending on the size of the file, this command should take less than 5 minutes to run.

```
samtools sort C50.bam C50.sorted
```

Now make an index file of each sorted bam file using the following command. Do this for each sorted `bam file.`

```
samtools index C50.sorted.bam
```

Parallel mpileup of sorted bam files

Index reference sequence. Use following command, where "NZ_CP010555_PaerFRD1.fa" name of reference file:

```
samtools faidx ./NZ_CP010555_PaerFRD1.fa
```

Next, count the number of nucleotides in your reference genome by looking at the fasta index file (.fai file) in the directory. It is the first number (second column) of the file.

```
cat NZ_CP010555_PaerFRD1.fa.fai
```

Take this number and divide it by 24 (assuming 24 is the number of cores for the MacPro) and then add 10 to the quotient so there'd be no remainder if divided by 24. Then run the following command. It splits the fasta index file and does 24 separate pileups in 24 separate regions of the reference genome, creating 24 tmp.vcf files that include all bases across the genome.

```
vcfutils.pl splitchr -l 279690 ./NZ_CP010555_PaerFRD1.fa.fai | xargs -I {} -
n 1 -P 24 sh -c "samtools mpileup -f ./NZ_CP010555_PaerFRD1.fa -r '{}' -D -g
*.sorted.bam | bcftools view - > tmp.{}.vcf"
```

Now we have to concatenate the different vcf files to make one. Use the follow command to make one sorted vcf file called res.vcf.

```
vcf-concat tmp.*.vcf | vcf-sort > res.vcf
```

All downstream bcftools or vcftools commands can be performed on the output file from the vcf-concat command. We call variants with the following command:

```
bcftools call -c -v res.vcf > res_variants.vcf
```

Filtering of High-quality SNPs

Parse the variants to include only high-quality SNPs (hqSNPs) with mean coverage > 30X, minimum quality > 200 with the following command:

```
vcftools --vcf res_variants.vcf --min-meanDP 30 --minQ 200 --remove-indels -
-recode --out C50_HQ_SNPonly
```

Now, remove all heterozygote calls from our haploid genomes with the following command.

```
grep -v "0/1" C50_HQ_SNPonly.recode.vcf > C50_HQ_SNP_nohet.recode.vcf
```

If generated `*_HQ_SNP_nohet.recode.vcf` file doesn't contain any SNPs, it means that reference 100% matched tested samples.

Alignment of SNPs in SVAMP

Open SVAMP software: Desktop →Outbreak → SVAMP

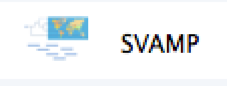

File → Load

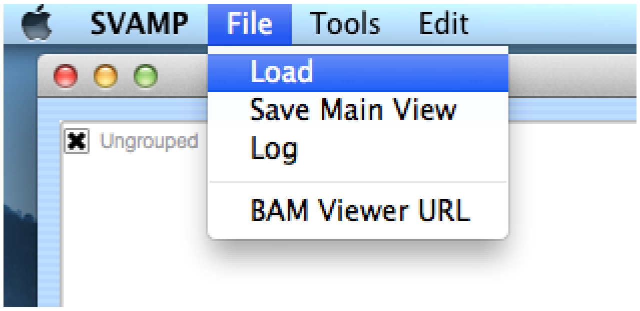

Reference (fasta): Chose original reference file exported from CLCbio (fasta or fa format).
Annotation (gff): chose annotation for the reference file exported from CLCbio in gff format
Variation (vcf): chose final vcf file with hqSNPs only and without heterozygotes (e.g. `C50_HQ_SNP_nohet.recode.vcf`)

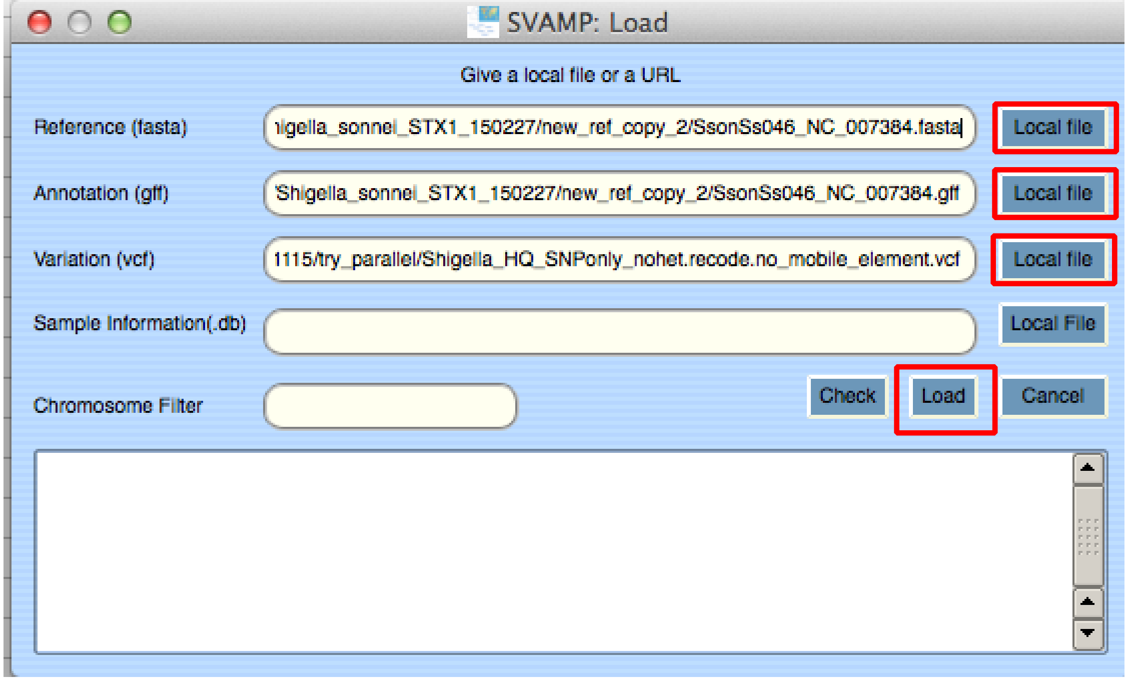

After sequences were loaded perform alignment for tree building:

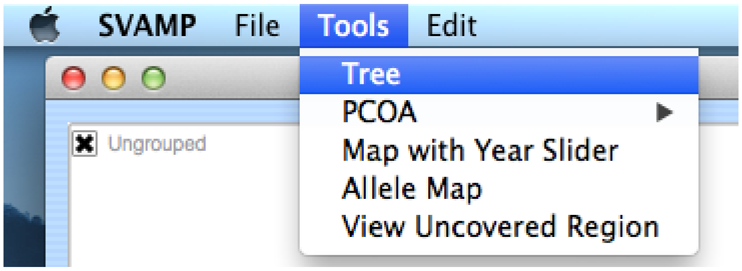

In newly open window: "Save alignment and compute tree using 3rd party phylogeny software" → Save alignment to directory → choose directory … → Save:

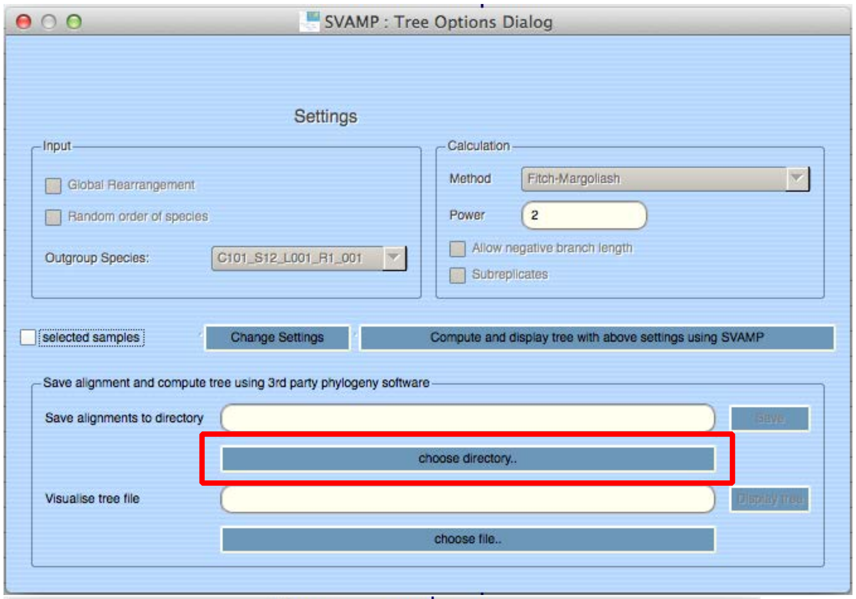

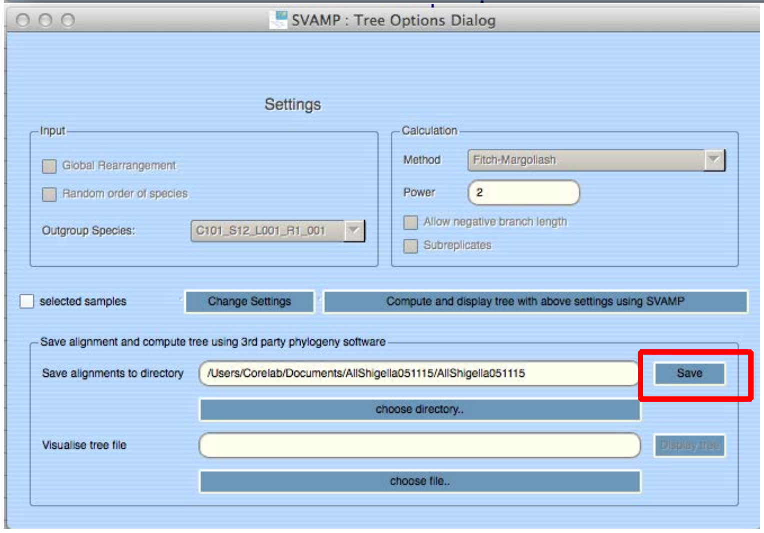

In next window select a region to perform alignment on. Choose “Chromosome”:

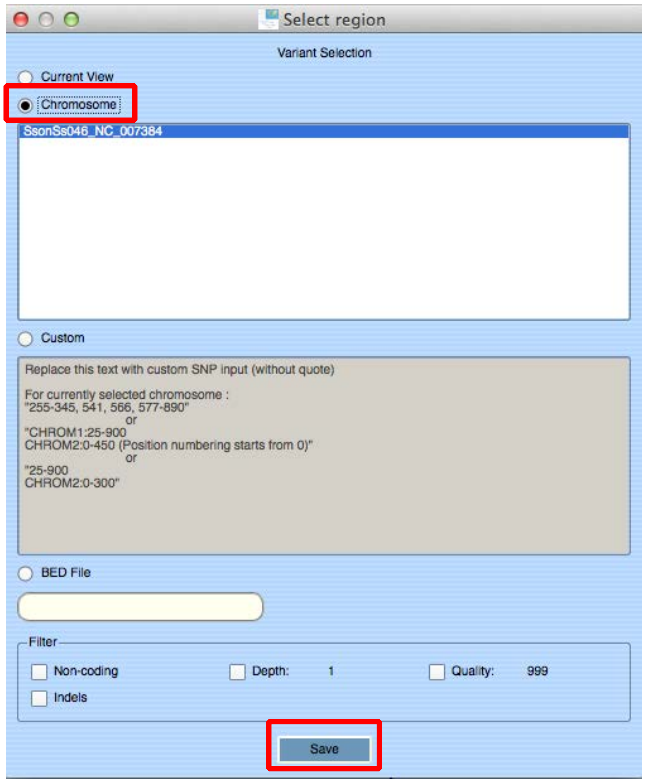

Go to the directory where you have saved the alignment and copy align.fasta file to import in into the CLCbio:

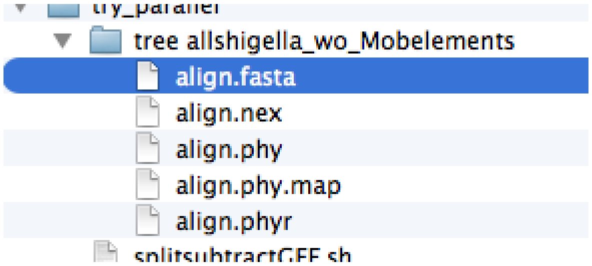

Alignment in CLCbio

Import align.fasta file into the CLCbio: Import → Standard Import…

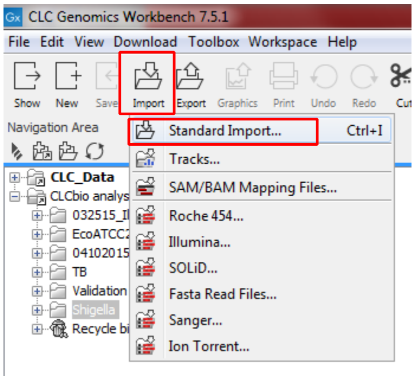

Pick the file:

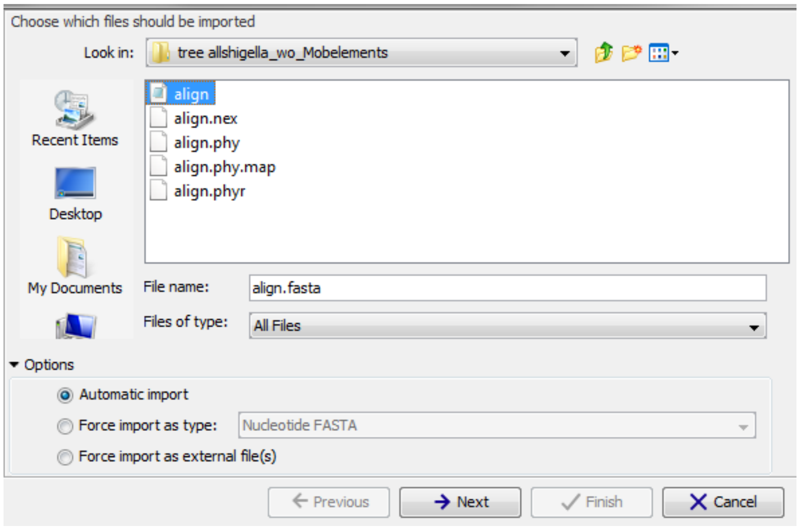

Choose the folder to save import to:

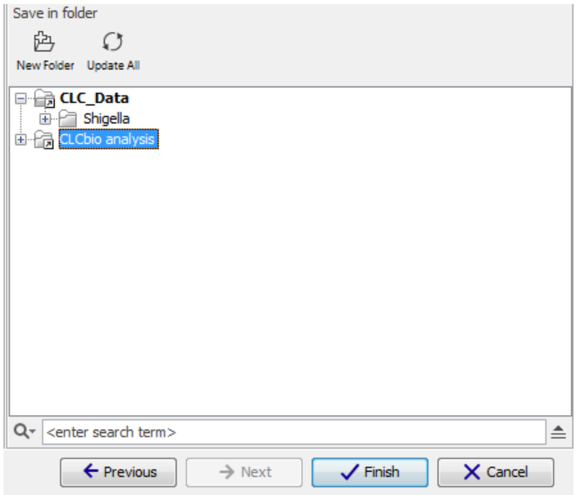

Create alignment:

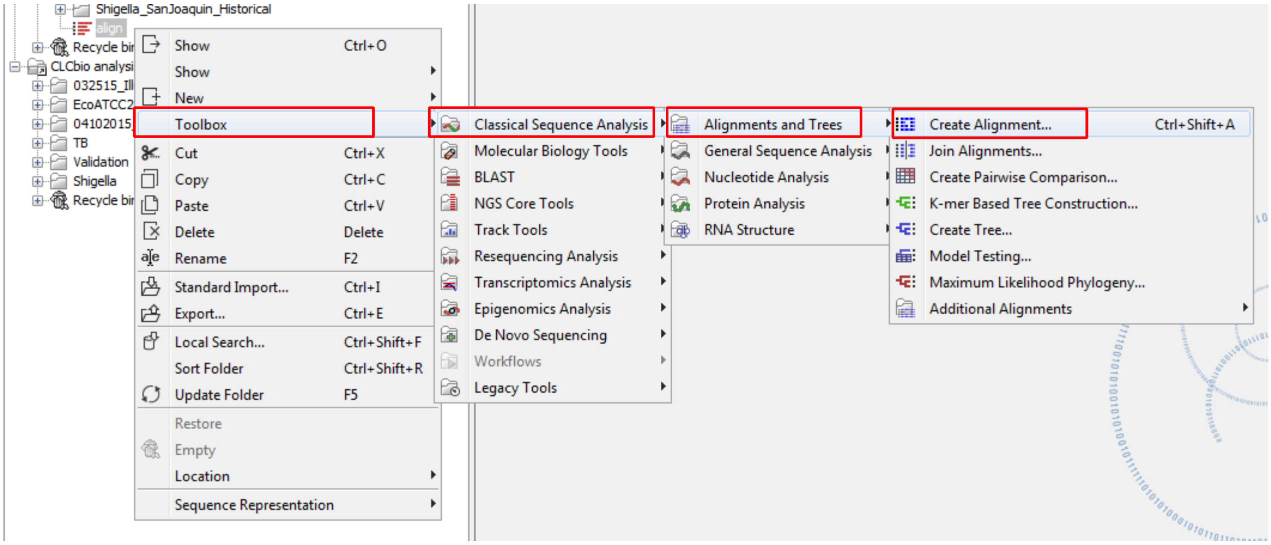

Use imported fasta file for alignment. Ignore note about time of analysis:

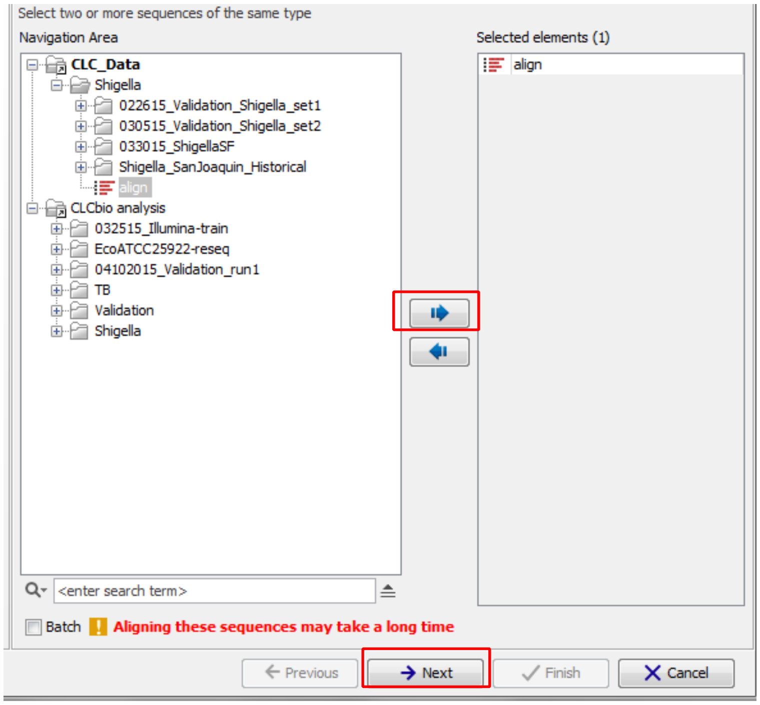

Use following parameters:

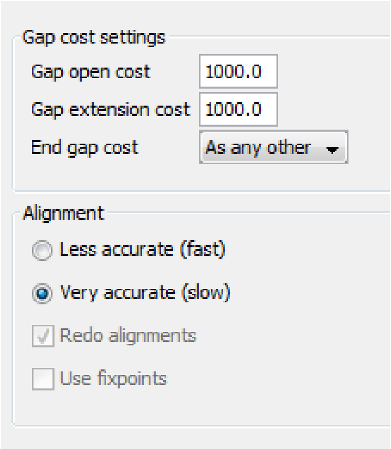

Save alignment:

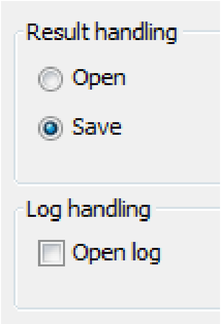

Comparison matrix

Create pairwise comparison table from alignment file:

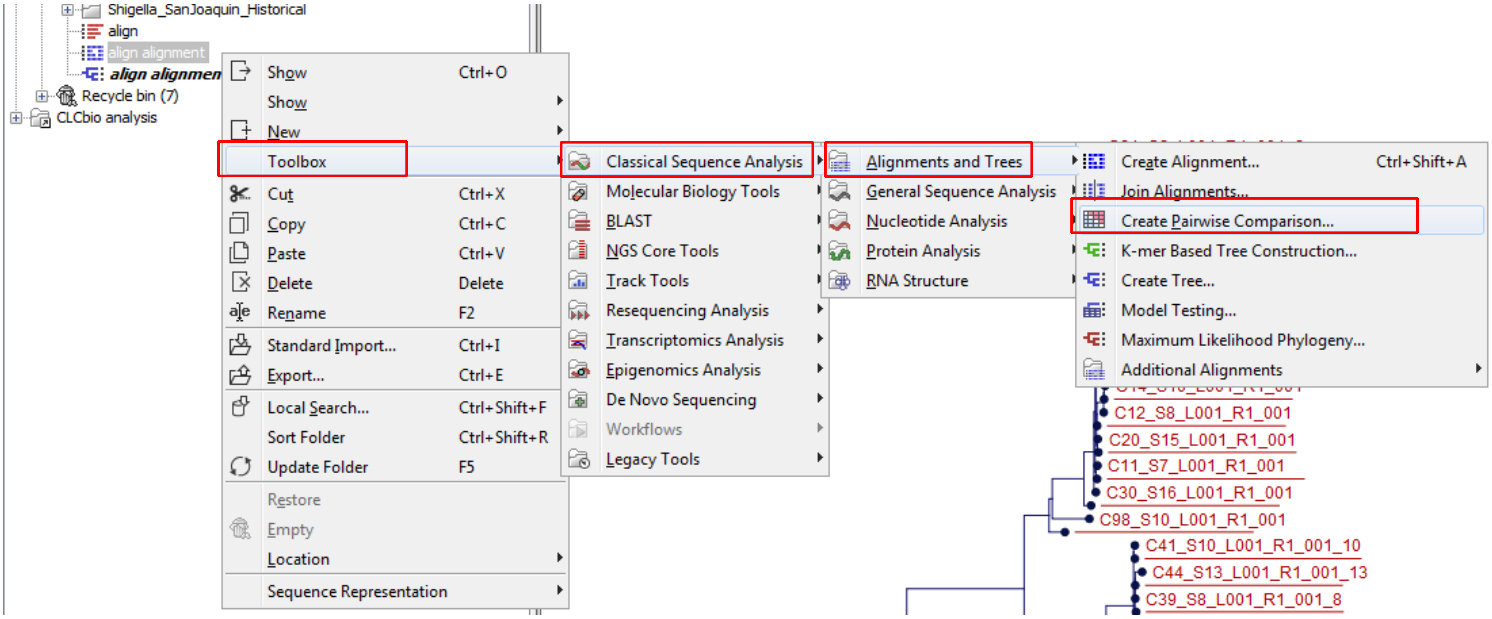

Use alignment file:

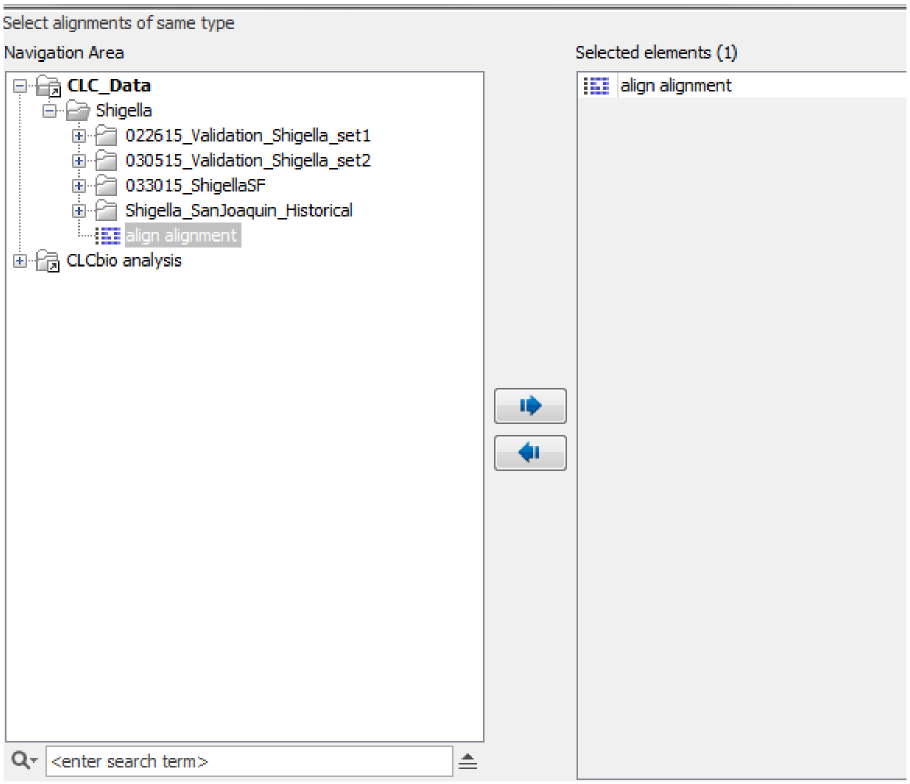

Set parameters:

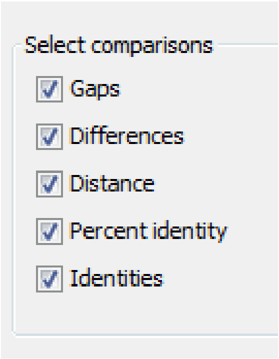

Save:

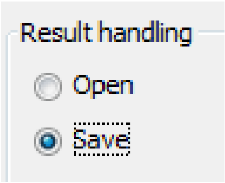

Save in the same directory as alignment file

To fit full table on a screen can reduce the font size:

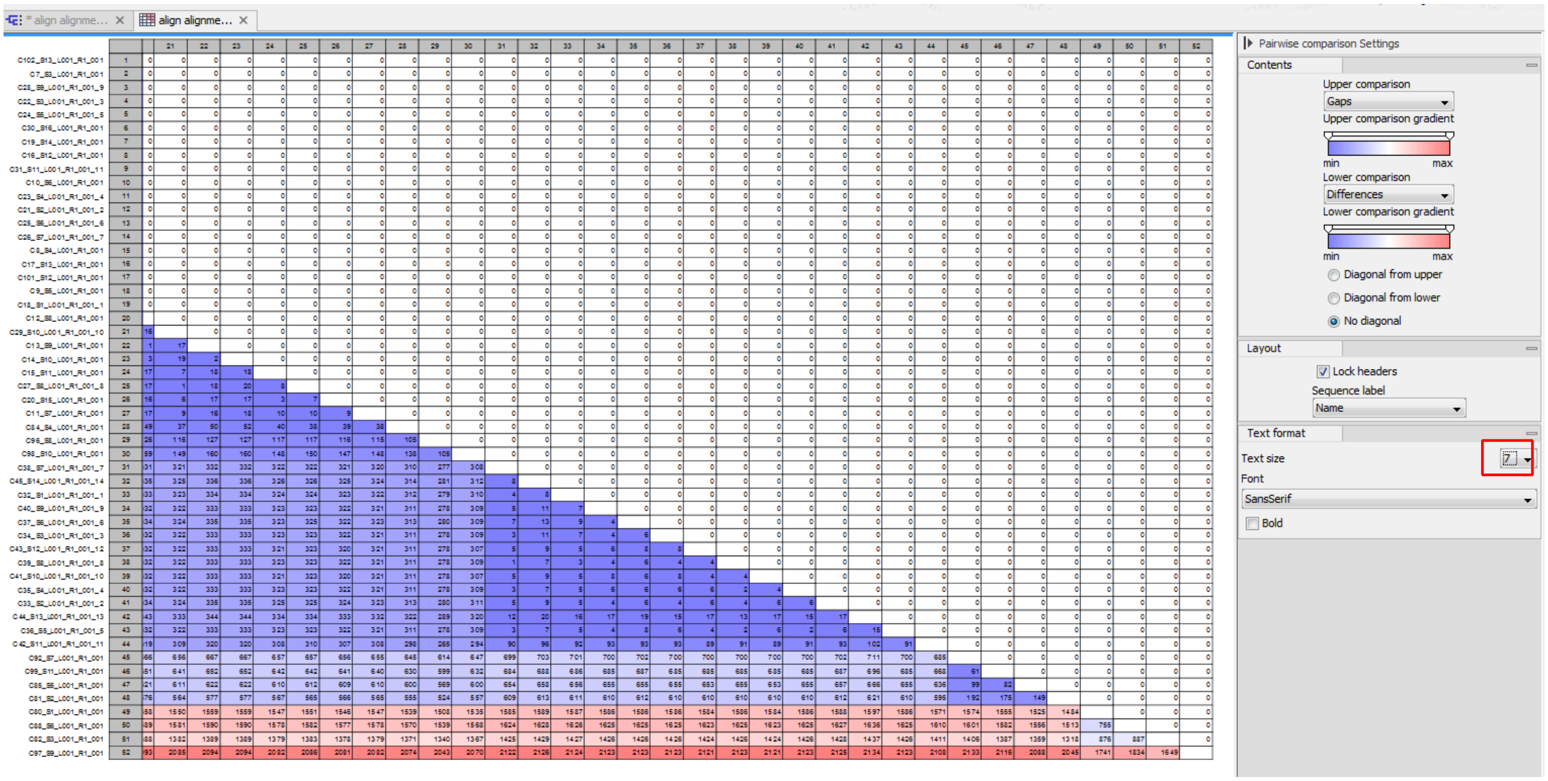

Data interpretation

I. Total # of SNP difference with the reference

Use alignment length to tell # of SNP difference between Core lab sequences and NCBI reference:

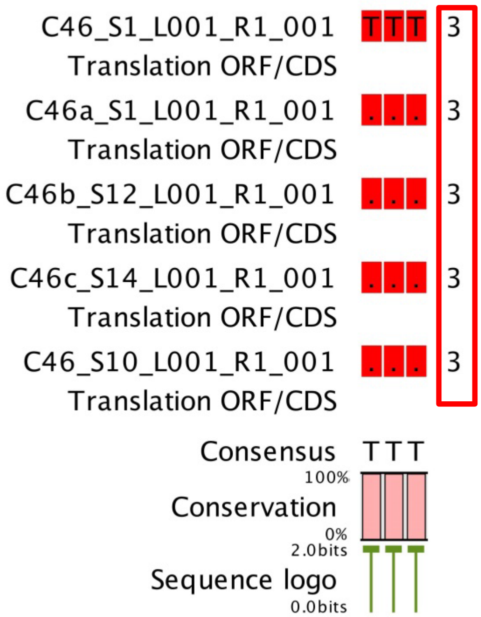

In the example above each of 5 replicates (within- and between-run) differ from the reference by 3 SNPs. Total # of SNP difference with the reference in this case is 3.

II. # of sequencing errors (SNP is supported only by 4 or less validation replicates). View alignment above. Since the 3 detected SNPs are identical in all 5 replicates the difference between validation sequencing results and the reference should be ignored. Number of sequencing errors in this case is 0. The test is considered to have 100% accuracy. The difference with the reference sequence in such case is obviously caused by mutations acquisition by ATCC strain but not by false base pair call. The SNP difference with the reference is considered to be real only in those cases when one or more of the replicates have different SNPs in this position.

III. Reproducibility and repeatability (Total # of SNP difference for within-run replicates, Total # of SNP difference for between-run replicated, # of within-run replicates in agreement, # of between-run replicates in agreement**).**

Use Pairwise comparison matrix to record difference between the replicates

Example 1:

		1	2	3	4	5
C46_S1_L001_R1_001	1		0	0	0	0
C46a_S1_L001_R1_001	2	0		0	0	0
C46b_S12_L001_R1_001	3	0	0		0	0
C46c_S14_L001_R1_001	4	0	0	0		0
C46_S10_L001_R1_001	5	0	0	0	0	

E.g. in example above samples C46a, C46b, C46c (positions in table ## 2,3,4) designate within run replicates. Samples C46_S1, C46_S10, and (C46a, C46b, C46c) are between run replicates. There is 0 SNP difference between all the replicates, meaning that for given sample reproducibility and repeatability is 100%. Total # of SNP difference for within-run replicates = 0, Total # of SNP difference for between-run replicated = 0, # of within-run replicates in agreement = 3, # of between-run replicates in agreement = 3

Example 2:

		1	2	3	4	5
C50-1_S4_L001_R1_001	1		0	0	0	0
C50-2_S11_L001_R1_001	2	0		0	0	0
C50_S14_L001_R1_001	3	0	0		0	0
C50_S2_L001_R1_001	4	0	0	0		0
C50-3_S15_L001_R1_001	5	1	1	1	1	

C50-1_S4, C50-2_S11, C50-3_S15 here are within-run replicates. C50_S14, C5_S2, and (C50-1_S4, C50-2_S11, C50-3_S15) are between-run replicates. One within run replicate C50-3_S15 has one SNP difference from other within run replicates, which are otherwise are identical and don't have any SNP difference with between run replicates. In this example: Total # of SNP difference for within-run replicates = 1, Total # of SNP difference for between-run replicated = 0, # of within-run replicates in agreement =2, # of between-run replicates in agreement = 3

Example 3:

		1	2	3	4	5
C49_S13_L001_R1_001	1		0	0	0	0
C49a_S4_L001_R1_001	2	0		0	0	0
C49b_S11_L001_R1_001	3	0	0		0	0
C49c_S14_L001_R1_001	4	0	0	0		0
C49_S4_L001_R1_001	5	1	1	1	1	

C49a_S4, C49b_S11, C49c_S14 here are within-run replicates. C49_S13, C49_S4, and (C49a_S4, C49b_S11, C49c_S14) are between-run replicates. All within run replicates in this case are identical. One between run replicate C49_S4 has one SNP difference from other between run replicates. In this example: Total # of SNP difference for within-run replicates = 0, Total # of SNP difference for between-run replicated = 1, # of within-run replicates in agreement =3, # of between-run replicates in agreement = 2.

Appendix 6. MLST and 16s rRNA analysis protocol and results

Protocol for 16S rRNA gene analysis

Open annotated *de novo* assembled genome of validation sample or reference ATCC sequence in GenBank format (with annotation). Search for annotation “16S”. Copy and paste DNA sequence for 16S RNA gene in text file. Change txt extension to .fasta. Use RDP site to upload this text file.

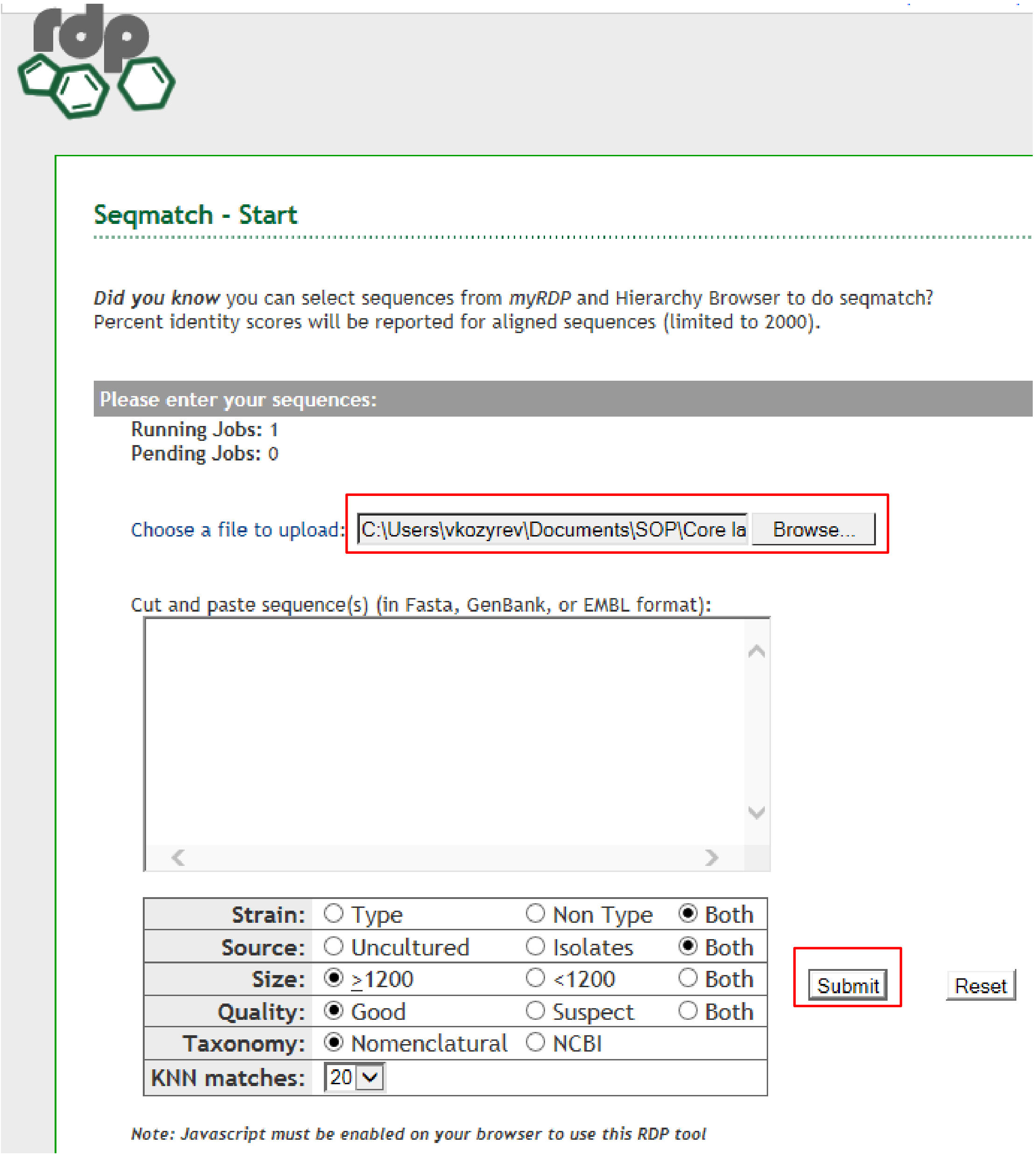

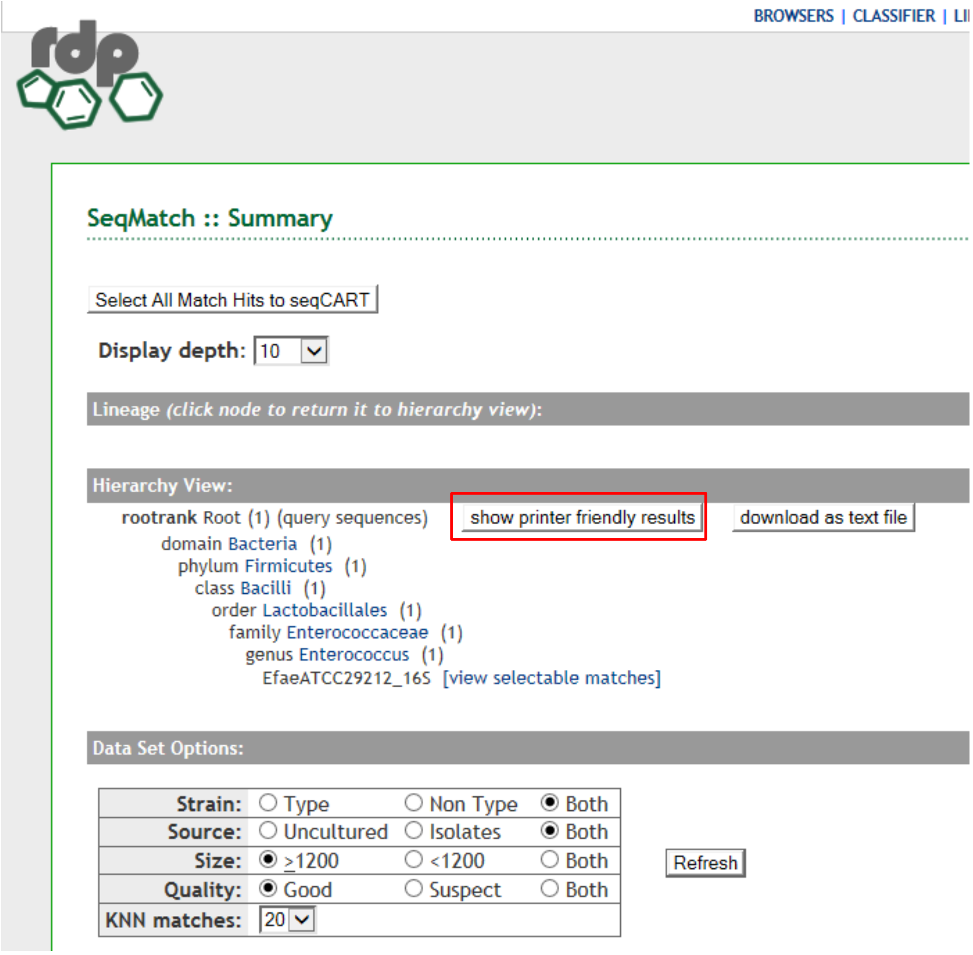

BROWSERS | CLASSIFIER | LI
rdp
SeqMatch :: Summary
Select All Match Hits to seqCART
Display depth: 10
Lineage (click node to return it to hierarchy view):
Hierarchy View:
rootrank Root (1) (query sequences)
show printer friendly results
download as text file
domain Bacteria (1)
phylum Firmicutes (1)
class Bacilli (1)
order Lactobacillales (1)
family Enterococcaceae (1)
genus Enterococcus (1)
EfaeATCC29212_16S [view selectable matches]
Data Set Options:
Strain: Type Non Type Both
Source: Uncultured Isolates Both
Size: >1200 <1200 Both
Quality: Good Suspect Both
KNN matches: 20
Refresh

Results:

SeqMatch :: Result

[new match | help]

Seqmatch: version 3
RDP Data: release11_4
Data Set: both type and non-type strains, both environmental (uncultured) sequences and isolates, near-full-length sequences (≥1200 bases), good quality sequences
Comments: 1504475 sequences were included in the search
The screening was based on 7-base oligomers
Query Submit Date: Wed Jul 01 15:33:44 EDT 2015

Match hit format: short ID, orientation, similarity score, S_ab score, unique common oligomers and sequence full name. More help is available.

Lineage:
Results for Query Sequence: EfaeATCC29212_16S, 1482 unique oligos

rootrank Root (20) (match sequences)
domain Bacteria (20)
phylum Firmicutes (20)
class Bacilli (20)
order Lactobacillales (20)
family Enterococcaceae (20)
genus Enterococcus (20)

S000009238 not_calculated 1.000 1376 Enterococcus faecalis; Y18293
S000012997 not_calculated 1.000 1417 Enterococcus faecalis; AF039902
S000468458 not_calculated 1.000 1470 Enterococcus faecalis; SFL; AY850358
S000485949 not_calculated 1.000 1402 Enterococcus faecalis; C13115; AY550919
S000487878 not_calculated 1.000 1394 uncultured Enterococcus sp.; 1-2; AY830398
S000487884 not_calculated 1.000 1398 uncultured Enterococcus sp.; 2-4; AY830404
S000487886 not_calculated 1.000 1395 uncultured Enterococcus sp.; 2-7; AY830406
S000495530 not_calculated 1.000 1435 Enterococcus faecalis; K-4; AB036835
S000651465 not_calculated 1.000 1407 Enterococcus faecalis; ATCC 19433; DQ411814
S000709541 not_calculated 1.000 1394 uncultured bacterium; aaa50g08; DQ817930
S000710171 not_calculated 1.000 1405 uncultured bacterium; aaa44e09; DQ818560
S000710930 not_calculated 1.000 1410 uncultured bacterium; aab28d06; DQ819319
S000826989 not_calculated 1.000 1435 uncultured bacterium; BF0001D065; AM697450
S000827002 not_calculated 1.000 1435 uncultured bacterium; BF0001D078; AM697463
S000838154 not_calculated 1.000 1419 uncultured bacterium; P2D1-555; EF510402
S000838194 not_calculated 1.000 1419 uncultured bacterium; P2D1-740; EF510442
S000969143 not_calculated 1.000 1407 uncultured bacterium; ca; AB291625
S001059411 not_calculated 1.000 1353 uncultured bacterium; PB1_aai26d05; EU460312
S001095541 not_calculated 1.000 1323 Enterococcus faecalis; 17A; EU168400
S001095542 not_calculated 1.000 1336 Enterococcus faecalis; 4B; EU168401

Protocol for *in silico* MLST analysis

Use MLST online tool from Center for Genomic Epidemiology:
https://cge.cbs.dtu.dk/services/MLST/
Chose the MLST configuration from drop down list according to the species.
Type of reads- “Assembled Genome/Contigs” or “Illumina – paired end reads” for validation samples
For NCBI reference use complete sequence in fasta format and pick Type of reads- “Assembled Genome/Contigs”

If chosen “Assembled Genome/Contigs” click buton “Isolate File” and load assembled *de novo* contigs, saved as single fasta file.

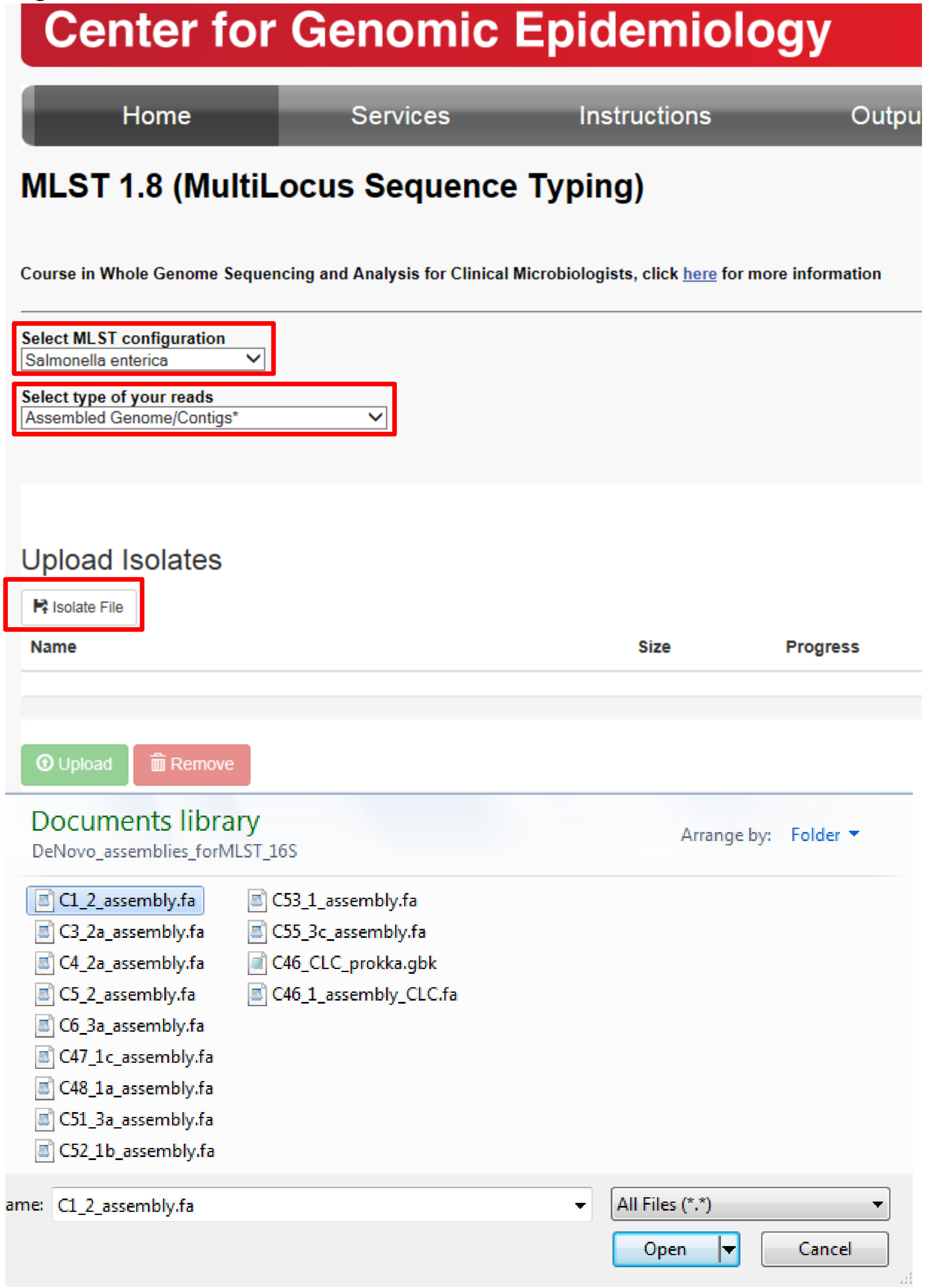

When name of the file appears in field “upload isolates”, click button “Upload”:

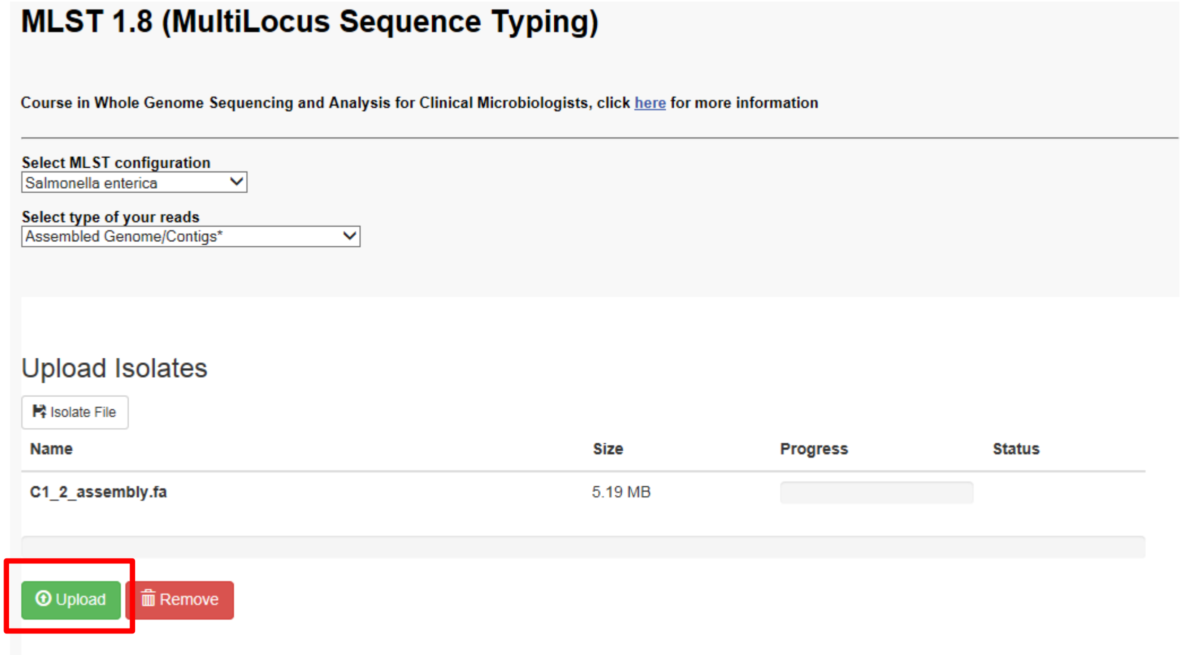

If chosen “Illumina – paired end reads” ” click buton “Isolate File” and load original fastq files of both paired reads for one sample at a time”

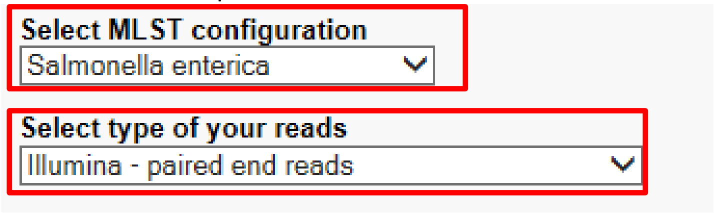

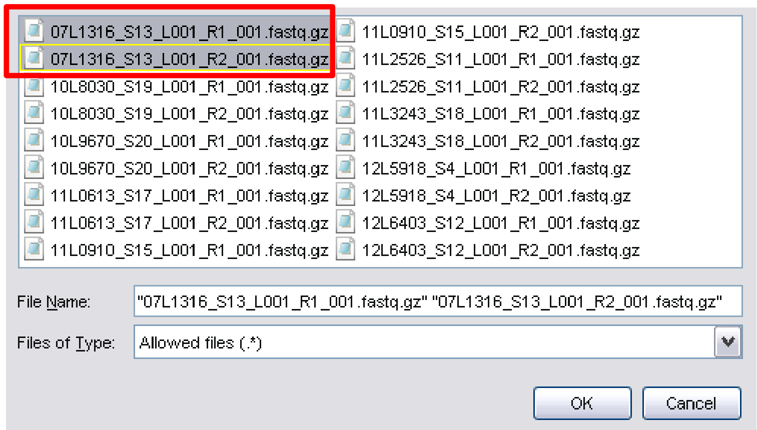

Click buton “Upload”

Allow server to process request. Don’t close the window.

Example of the result report of the CGE site:

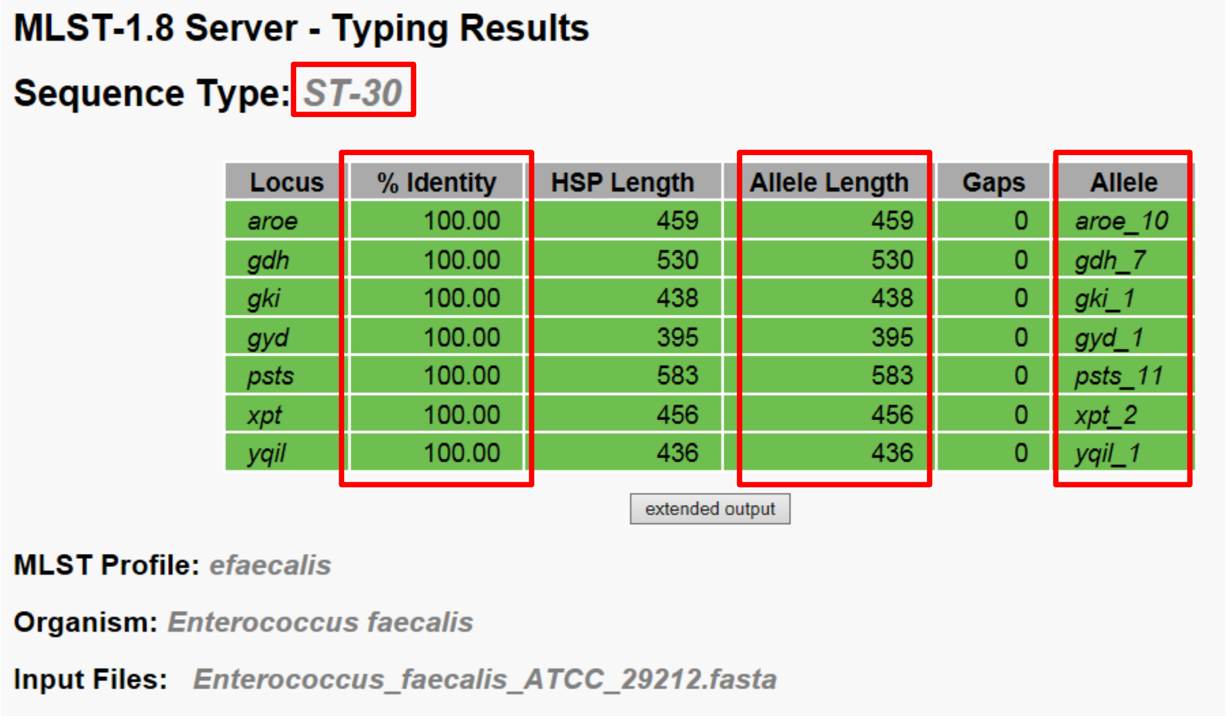

MLST-1.8 Server - Typing Results

Sequence Type: *ST-30*

Locus	% Identity	HSP Length	Allele Length	Gaps	Allele
aroe	100.00	459	459	0	*aroe_10*
gdh	100.00	530	530	0	*gdh_7*
gki	100.00	438	438	0	*gki_1*
gyd	100.00	395	395	0	*gyd_1*
psts	100.00	583	583	0	*psts_11*
xpt	100.00	456	456	0	*xpt_2*
yqil	100.00	436	436	0	*yqil_1*

extended output

MLST Profile: *efaecalis*

Organism: *Enterococcus faecalis*

Input Files: *Enterococcus_faecalis_ATCC_29212.fasta*

Record Sequence type number (ST). ST is considered as valid only when identity of all loci is 100% and allele length is matching HSP length.

When comparing several isolates record loci which are different between the isolates and corresponding allele numbers for those loci.

Click button "Extended output". Save results as text.

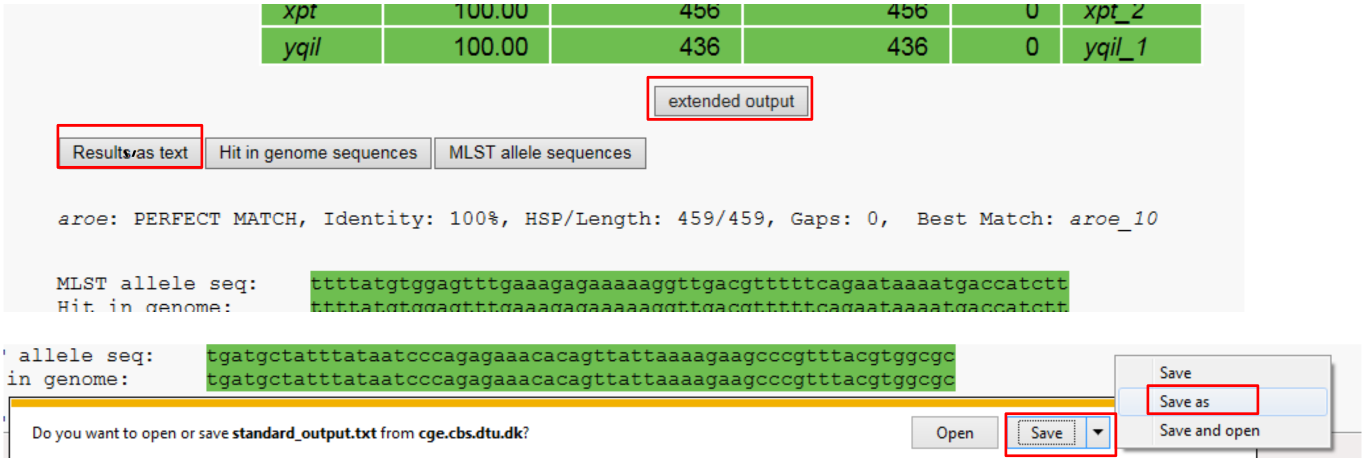

MLST negative controls

Negative control

		Negative control				
	**MLST database**	**Sample ID**	**Species**	**# of alleles matching negative control MLST profile**	**Results**	
1	Salmonella enterica	C1	Escherichia coli	0	unknown ST	neg
2	Staphylococcus aureus	C46	Enterococcus faecalis	0	unknown ST	neg
3	Pseudomonas aeruginosa	C51	Stenotrophomonas maltophilia	0	unknown ST	neg
4	Escherichia coli #1	C73	Salmonella enterica	0	unknown ST	neg
5	Staphylococcus epidermidis	C5	Staphylococcus aureus	0	unknown ST	neg

Control 1 results:

MLST-1.8 Server - Typing Results

Sequence Type: *Unknown ST*

Locus	% Identity	HSP Length	Allele Length	Gaps	Allele
aroc	85.74	491	501	0	*aroc-391*
dnan	87.03	501	501	0	*dnan-389*
hemd	99.30	429	432	0	*hemd-343*
hisd	82.20	500	501	0	*hisd-11*
pure	97.73	397	399	0	*pure-487*
suca	98.00	501	501	0	*suca-281*
thra	81.56	488	501	0	*thra-208*

Please note that one or more loci do not match perfectly to any previously registered MLST allele.
We recommend verifying the results by traditional methods for MLST!

extended output

MLST Profile: *senterica*

Organism: *Salmonella enterica*

Input Files: *C1_2_assembly.fa*

Control 2 results:

MLST-1.8 Server - Typing Results

Sequence Type: *Unknown ST*

Locus	% Identity	HSP Length	Allele Length	Gaps	Allele
arcc	91.18	34	436	0	*arcc-77*
aroe	89.29	28	456	0	*aroe-365*
glpf	87.18	39	450	0	*glpf-205*
gmk	92.31	39	417	0	*gmk_-173*
pta	79.26	188	474	0	*pta_-94*
tpi	80.00	90	402	0	*tpi_-227*
yqil	94.74	19	467	0	*yqil-277*

Please note that one or more loci do not match perfectly to any previously registered MLST allele.
We recommend verifying the results by traditional methods for MLST!

extended output

MLST Profile: *saureus*

Organism: *Staphylococcus aureus*

Input Files: *C46_1_assembly_CLC.fa*

Control 3 results:

MLST-1.8 Server - Typing Results

Sequence Type: *Unknown ST*

Locus	% Identity	HSP Length	Allele Length	Gaps	Allele
acs	85.58	312	390	0	*acs_151*
aro	90.24	41	498	0	*aro_136*
gua	97.05	373	373	0	*gua_121*
mut	82.30	113	442	2	*mut_110*
nuo	92.31	26	366	0	*nuo_102*
pps	84.70	183	370	0	*pps_123*
trp	87.65	81	443	0	*trp_104*

Please note that one or more loci do not match perfectly to any previously registered MLST allele.
We recommend verifying the results by traditional methods for MLST!

extended output

MLST Profile: *paeruginosa*

Organism: *Pseudomonas aeruginosa*

Input Files: *C51_3a_assembly.fa*

Control 4 results:

MLST-1.8 Server - Typing Results

Sequence Type: *Unknown ST*

Locus	% Identity	HSP Length	Allele Length	Gaps	Allele
adk	99.07	536	536	0	*adk-44*
fumc	99.36	469	469	0	*fumc-624*
gyrb	98.70	460	460	0	*gyrb-212*
icd	100.00	518	518	0	*icd-40*
mdh	99.34	452	452	0	*mdh-392*
pura	98.95	478	478	0	*pura-29*
reca	99.02	510	510	0	*reca-338*

Please note that one or more loci do not match perfectly to any previously registered MLST allele.
We recommend verifying the results by traditional methods for MLST!

extended output

MLST Profile: *ecoli*

Organism: *Escherichia coli#1*

Input Files: *C73_1a_assembly.fa*

Control 5 results:

MLST-1.8 Server - Typing Results

Sequence Type: *Unknown ST*

Locus	% Identity	HSP Length	Allele Length	Gaps	Allele
arcc	80.25	314	465	0	*arcc-19*
aroe	84.62	65	420	0	*aroe-28*
gtr	79.72	434	438	0	*gtr-35*
muts	84.21	323	412	0	*muts-41*
pyr	83.96	106	428	0	*pyr-36*
tpi	82.09	374	424	0	*tpi-19*
yqil	80.29	274	416	0	*yqil-26*

Please note that one or more loci do not match perfectly to any previously registered MLST allele.
We recommend verifying the results by traditional methods for MLST!

extended output

MLST Profile: *sepidermidis*

Organism: *Staphylococcus epidermidis*

Input Files: *C5_2_assembly.fa*

Comparison of the results of 16S rRNA ID and *in silico* MLST for accuracy estimation for each sample can be found in a separate file.

Appendix 7. Validation testing plan

ID numbers of tested isolates, dates and names of operators for the validation runs:

	**Operator**	**Date**
Run 1	VK	2/23/2015

			Replicate 1	Replicate 2	Replicate 3
C1	O157:H7 CDC EDL 933	Escherichia coli	●		
C3	ATCC 8739	Escherichia coli	●		
Shared run with 14 *Shigella sonnei* genomes (Research Use Only)					

	**Operator**	**Date**
**Run 2**	VK	3/5/2015

			Replicate 1	Replicate 2	Replicate 3
C1	O157:H7 CDC EDL 933	*Escherichia coli*	●		
C4	ATCC 13047	*Enterobacter cloacae*	●		
C5	ATCC 25923	*Staphylococcus aureus*	●		
C6	ATCC 14028	*Salmonella enterica* ser Typhimurium	●		
C55	ATCC 25922	*Escherichia coli*	●		
Shared run with 11 *Shigella sonnei* genomes (Research Use Only)					

	**Operator**	**Date**
**Run 3**	VK	3/30/2015

			Replicate 1	Replicate 2	Replicate 3
C6	ATCC 14028	*Salmonella enterica* ser Typhimurium	●		
C55	ATCC 25922	*Escherichia coli*	●		
Shared run with 14 *Shigella sonnei* genomes (Research Use Only)					

	**Operator**	**Date**
**Run 4**	CLT	4/10/2015

			Replicate 1	Replicate 2	Replicate 3
C1	O157:H7 CDC EDL 933	*Escherichia coli*	●	●	●

C3	ATCC 8739	Escherichia coli	●	●	●
C4	ATCC 13047	*Enterobacter cloacae*	●	●	●
C5	ATCC 25923	*Staphylococcus aureus*	●		
C6	ATCC 14028	*Salmonella enterica* ser Typhimurium	●	●	●
C55	ATCC 25922	*Escherichia coli*	●	●	●

	**Operator**	**Date**
**Run 5**	VK	4/14/2015

			Replicate 1	Replicate 2	Replicate 3
C46	ATCC 29212	Enterococcus faecalis	●		
C47	96A-9665	Staphylococcus epidermidis	●	●	●
C48	83A-2517	Staphylococcus saprophyticus	●	●	●
C52	87A-512	Legionella pneumophila SG-12	●	●	●
C50	ATCC 27853	Pseudomonas aeruginosa	●	●	●
C53	87A-3084	Moraxella catarrhalis	●		
C54	90A-4973	Acinetobacter baumannii	●		
C51	ATCC 13637	Stenotrophomonas maltophilia	●		

	**Operator**	**Date**
**Run 6**	CLT	4/17/2015

			Replicate 1	Replicate 2	Replicate 3
C56	11L2526	Mycobacterium tuberculosis	●	●	●
C57	12L6403	Mycobacterium tuberculosis	●	●	●
C58	07L1316	Mycobacterium tuberculosis	●	●	●
C59	12L5918	Mycobacterium tuberculosis	●	●	●
C61	11L0910	Mycobacterium tuberculosis	●	●	●
C65	11L0613	Mycobacterium tuberculosis	●		
C67	11L3243	Mycobacterium tuberculosis	●		
C68	10L8030	Mycobacterium tuberculosis	●		
C69	10L9670	Mycobacterium tuberculosis	●		

	**Operator**	**Date**
**Run 7**	VK	4/21/2015

			Replicate 1	Replicate 2	Replicate 3
C72	M14X02267	*Escherichia coli*	●	●	●
C73	M10X01956	*Salmonella* Enteritidis	●	●	●
C74	M14X00933	*Salmonella* Infantis	●	●	●
C75	M14X04729	*Salmonella* Adelaide	●	●	●
C76	M12X03253	*Salmonella* Worthington	●	●	●

C4	ATCC 13047	*Enterobacter cloacae*	●		

Operator **Date**

Run 8 VK 4/28/2015

			Replicate 1	Replicate 2	Replicate 3
C72	M14X02267	*Escherichia coli*	●		
C73	M10X01956	*Salmonella* Enteritidis	●		
C74	M14X00933	*Salmonella* Infantis	●		
C75	M14X04729	*Salmonella* Adelaide	●		
C76	M12X03253	*Salmonella* Worthington	●		
C46	ATCC 29212	Enterococcus faecalis	●	●	●
C47	96A-9665	Staphylococcus epidermidis	●		
C48	83A-2517	Staphylococcus saprophyticus	●		
C49	87A-257	Streptococcus pneumoniae	●		
C52	87A-512	Legionella pneumophila SG-12	●		
C53	87A-3084	Moraxella catarrhalis	●	●	●
C54	90A-4973	Acinetobacter baumannii	●		

Operator **Date**

Run 9 CLT 5/1/2015

			Replicate 1	Replicate 2	Replicate 3
C72	M14X02267	*Escherichia coli*	●		
C73	M10X01956	*Salmonella* Enteritidis	●		
C74	M14X00933	*Salmonella* Infantis	●		
C75	M14X04729	*Salmonella* Adelaide	●		
C76	M12X03253	*Salmonella* Worthington	●		
C3	ATCC 8739	*Escherichia coli*	●		
C5	ATCC 25923	*Staphylococcus aureus*	●	●	●
C46	ATCC 29212	Enterococcus faecalis	●		
C47	96A-9665	Staphylococcus epidermidis	●		
C48	83A-2517	Staphylococcus saprophyticus	●		
C49	87A-257	Streptococcus pneumoniae	●		
C50	ATCC 27853	Pseudomonas aeruginosa	●		
C51	ATCC 13637	Stenotrophomonas maltophilia	●		
C52	87A-512	Legionella pneumophila SG-12	●		

Operator **Date**

Run 10 VK 5/12/2015

Replicate 1	Replicate 2	Replicate 3

C49	87A-257	Streptococcus pneumoniae	●	●	●
C50	ATCC 27853	Pseudomonas aeruginosa	●		
C51	ATCC 13637	Stenotrophomonas maltophilia	●	●	●
C53	87A-3084	Moraxella catarrhalis	●		
C54	90A-4973	Acinetobacter baumannii	●	●	●
C103	00A-04614	Bacteroides fragilis	●		
C104	89A-2867	Haemophilus influenzae type b	●		
C2	ATCC 7966	Aeromonas hydrophilia	●		
C105	94A-6422	Corynebacterium jeikeium	●		
C106	84A-5863	Neisseria gonorrhoeae	●		

	**Operator**	**Date**
**Run 11**	CLT	5/15/2015

			Replicate 1	Replicate 2	Replicate 3
C56	11L2526	Mycobacterium tuberculosis	●		
C57	12L6403	Mycobacterium tuberculosis	●		
C58	07L1316	Mycobacterium tuberculosis	●		
C59	12L5918	Mycobacterium tuberculosis	●		
C61	11L0910	Mycobacterium tuberculosis	●		
C65	11L0613	Mycobacterium tuberculosis	●		
C67	11L3243	Mycobacterium tuberculosis	●	●	●
C68	10L8030	Mycobacterium tuberculosis	●	●	●
C69	10L9670	Mycobacterium tuberculosis	●	●	●

	**Operator**	**Date**
**Run 12**	CLT	5/29/2015

			Replicate 1	Replicate 2	Replicate 3
C103	00A-04614	Bacteroides fragilis	●	●	●
C104	89A-2867	Haemophilus influenzae type b	●	●	●
C2	ATCC 7966	Aeromonas hydrophilia	●	●	●
C105	94A-6422	Corynebacterium jeikeium	●	●	●
C106	84A-5863	Neisseria gonorrhoeae	●	●	●
C77	M14X04723	Salmonella Saintpaul	●		

	**Operator**	**Date**
**Run 13**	CLT	7/13/2015

			Replicate 1	Replicate 2	Replicate 3
C56	11L2526	Mycobacterium tuberculosis	●		
C57	12L6403	Mycobacterium tuberculosis	●		
C58	07L1316	Mycobacterium tuberculosis	●		

C59	12L5918	Mycobacterium tuberculosis	●		
C61	11L0910	Mycobacterium tuberculosis	●		
C65	11L0613	Mycobacterium tuberculosis	●	●	●
C67	11L3243	Mycobacterium tuberculosis	●		
C68	10L8030	Mycobacterium tuberculosis	●		
C69	10L9670	Mycobacterium tuberculosis	●		
C103	00A-04614	Bacteroides fragilis	●		
C104	89A-2867	Haemophilus influenzae type b	●		
C2	ATCC 7966	Aeromonas hydrophilia	●		
C105	94A-6422	Corynebacterium jeikeium	●		
C106	84A-5863	Neisseria gonorrhoeae	●		

Appendix 8. Quality parameters for sequences of the replicates

Sample	Average read length with ≥ Q30 (= avg read length after trimming), (bp)	Read length at which 75% of bases have quality score ≥Q30, (bp)	Average depth of coverage of genome	Percentage of genome covered (after mobile elements masking)	Uniformity of Coverage		PhiX error rate	Percent of bases with quality score >Q30 for the run	Cluster density for the run, (K/mm^2)	Cluster passing filter of the run
					at 5x	at 10x				
**C1_1**	153.6	155	30.46	93	89.07%	88.09%	3.46%	63.8	1128	89.1%
**C1_2**	134.1	160	63.68	93	88.93%	88.75%	3.87%	61.1	1651	81.2%
**C1_3a**	138.5	170	67.19	93	96.93%	96.74%	3.57%	63.3	1505	84.3%
**C1_3b**	142	170	64.33	93	96.94%	96.76%	3.57%	63.3	1505	84.3%
**C1_3c**	131.6	155	54.56	93	96.87%	96.61%	3.57%	63.3	1505	84.3%
**C2_1**	132.3	140	85.69	100	99.80%	99.66%	3.67%	60.7	1272	88.9%
**C2_2a**	150.1	170	116.28	100	99.84%	99.75%	2.88%	68.7	1149	91.2%
**C2_2b**	156.9	165	99.33	100	99.83%	99.71%	2.88%	68.7	1149	91.2%
**C2_2c**	158.6	165	94.16	100	99.82%	99.68%	2.88%	68.7	1149	91.2%
**C2_3**	144.7	155	88.73	100	99.82%	99.66%	3.15%	62.3	1545	84.0%
**C3_1**	155.9	160	56.15	99	99.80%	99.71%	3.46%	63.8	1128	89.1%
**C3_2a**	139.6	170	86.54	99	99.76%	99.70%	3.57%	63.3	1505	84.3%
**C3_2b**	138.2	170	84.15	99	99.75%	99.70%	3.57%	63.3	1505	84.3%
**C3_2c**	134.1	150	65.11	99	99.73%	99.62%	3.57%	63.3	1505	84.3%
**C3_3**	133.7	165	103.01	99	99.74%	99.69%	3.43%	63.8	1172	89.7%
**C4_1**	126.7	140	70.58	98	99.47%	99.39%	3.87%	61.1	1651	81.2%
**C4_2a**	136.5	160	73.76	98	99.48%	99.41%	3.57%	63.3	1505	84.3%
**C4_2b**	127.5	140	40.43	98	99.38%	98.22%	3.57%	63.3	1505	84.3%
**C4_2c**	131.3	145	52.48	98	99.43%	99.18%	3.57%	63.3	1505	84.3%
**C4_3**	130.3	140	55.33	98	99.47%	99.34%	3.51%	60.4	1612	79.1%
**C5_1**	131.3	195	143.11	98	99.75%	99.70%	3.87%	61.1	1651	81.2%
**C5_2**	156	225	216.40	98	99.75%	99.75%	3.57%	63.3	1505	84.3%
**C5_3a**	154.3	225	154.26	98	99.76%	99.75%	3.43%	63.8	1172	89.7%
**C5_3b**	153.5	225	124.46	98	99.76%	99.75%	3.43%	63.8	1172	89.7%
**C5_3c**	184.1	220	76.67	98	99.79%	99.76%	3.43%	63.8	1172	89.7%
**C6_1**	128.2	145	84.45	99	98.80%	98.73%	3.87%	61.1	1651	81.2%
**C6_2**	133.3	150	68.37	99	98.79%	98.70%	3.42%	62.6	1507	84.6%

**C6_3a**	135.9	160	85.98	99	98.81%	98.76%	3.57%	63.3	1505	84.3%
**C6_3b**	129.8	165	66.77	99	98.77%	98.66%	3.57%	63.3	1505	84.3%
**C6_3c**	136.4	165	72.35	99	98.80%	98.72%	3.57%	63.3	1505	84.3%
**C46_1**	144.7	200	104.33	100	99.77%	99.69%	3.33%	62.8	1203	89.1%
**C46_2a**	137.2	185	101.62	100	99.80%	99.76%	4.74%	60.2	1693	76.1%
**C46_2b**	128.2	170	99.20	100	99.71%	99.62%	4.74%	60.2	1693	76.1%
**C46_2c**	136.6	185	146.75	100	99.83%	99.80%	4.74%	60.2	1693	76.1%
**C46_3**	174.9	200	58.05	100	99.41%	94.59%	3.43%	63.8	1172	89.7%
**C47_1a**	147.3	225	90.26	100	99.46%	97.91%	3.33%	62.8	1203	89.1%
**C47_1b**	147.8	225	94.63	100	99.37%	97.84%	3.33%	62.8	1203	89.1%
**C47_1c**	146.5	225	106.56	100	99.47%	98.35%	3.33%	62.8	1203	89.1%
**C47_2**	144.2	200	144.46	100	99.76%	99.71%	4.74%	60.2	1693	76.1%
**C47_3**	191.1	220	47.28	100	99.23%	94.89%	3.43%	63.8	1172	89.7%
**C48_1a**	150.1	220	134.57	100	99.94%	99.91%	3.33%	62.8	1203	89.1%
**C48_1b**	150	220	122.12	100	99.92%	99.87%	3.33%	62.8	1203	89.1%
**C48_1c**	146.4	220	127.84	100	99.93%	99.87%	3.33%	62.8	1203	89.1%
**C48_2**	143.2	200	132.38	100	99.94%	99.92%	4.74%	60.2	1693	76.1%
**C48_3**	183.9	215	60.88	100	99.96%	99.76%	3.43%	63.8	1172	89.7%
**C49_1**	132.8	185	105.38	88	86.56%	86.27%	4.74%	60.2	1693	76.1%
**C49_2**	175.7	195	51.54	88	86.45%	85.92%	3.43%	63.8	1172	89.7%
**C49_3a**	140.3	185	101.66	88	86.53%	86.13%	3.67%	60.7	1272	88.9%
**C49_3b**	135.2	180	58.25	88	86.35%	85.78%	3.67%	60.7	1272	88.9%
**C49_3c**	132.8	180	68.86	88	86.26%	85.51%	3.67%	60.7	1272	88.9%
**C50_1a**	121.5	120	47.50	92	92.38%	91.17%	3.33%	62.8	1203	89.1%
**C50_1b**	123.5	125	45.02	92	92.38%	91.09%	3.33%	62.8	1203	89.1%
**C50_1c**	119.2	110	41.87	92	92.23%	90.17%	3.33%	62.8	1203	89.1%
**C50_2**	132.3	90	15.71	92	88.27%	69.48%	3.43%	63.8	1172	89.7%
**C50_3**	131.5	130	64.56	92	92.59%	92.19%	3.67%	60.7	1272	88.9%
**C51_1**	122.4	120	57.61	100	99.52%	99.09%	3.33%	62.8	1203	89.1%
**C51_2**	135.7	115	23.88	100	98.35%	88.84%	3.43%	63.8	1172	89.7%
**C51_3a**	130.8	125	55.32	100	99.58%	99.43%	3.67%	60.7	1272	88.9%
**C51_3b**	126.1	135	69.32	100	99.56%	99.40%	3.67%	60.7	1272	88.9%
**C51_3c**	129.3	140	77.56	100	99.55%	99.37%	3.67%	60.7	1272	88.9%
**C52_1a**	144.5	200	110.8	100	99.79%	99.73%	3.33%	62.8	1203	89.1%
**C52_1b**	145.9	205	120.1	100	99.76%	99.71%	3.33%	62.8	1203	89.1%
**C52_1c**	139.5	190	110.14	100	99.78%	99.71%	3.33%	62.8	1203	89.1%

**C52_2**	134.4	185	99.81	100	99.75%	99.70%	4.74%	60.2	1693	76.1%
**C52_3**	168.1	190	51.81	100	99.74%	97.62%	3.43%	63.8	1172	89.7%
**C53_1**	137.4	180	105.68	95	95.18%	95.11%	3.33%	62.8	1203	89.1%
**C53_2a**	130.4	170	80.75	95	95.11%	95.09%	4.74%	60.2	1693	76.1%
**C53_2b**	131.7	175	119.56	95	95.19%	95.12%	4.74%	60.2	1693	76.1%
**C53_2c**	127.8	160	99.43	95	95.21%	95.14%	4.74%	60.2	1693	76.1%
**C53_3**	135.7	190	127.86	95	95.22%	95.17%	3.67%	60.7	1272	88.9%
**C54_1**	143.4	180	83.58	86	74.72%	74.48%	3.33%	62.8	1203	89.1%
**C54_2**	134.5	180	79.95	86	74.72%	74.52%	4.74%	60.2	1693	76.1%
**C54_3a**	137.8	180	83.44	86	74.68%	74.40%	3.67%	60.7	1272	88.9%
**C54_3b**	134.8	180	93.05	86	74.72%	74.44%	3.67%	60.7	1272	88.9%
**C54_3c**	137.3	190	97.74	86	74.70%	74.44%	3.67%	60.7	1272	88.9%
**C55_1**	126.3	160	66.20	97	99.64%	99.50%	3.87%	61.1	1651	81.2%
**C55_2**	133.1	155	80.92	97	99.73%	99.64%	3.42%	62.6	1507	84.6%
**C55_3a**	139.5	170	72.14	97	99.71%	99.62%	3.57%	63.3	1505	84.3%
**C55_3b**	132.3	155	56.52	97	99.67%	99.53%	3.57%	63.3	1505	84.3%
**C55_3c**	142.7	175	67.26	97	99.73%	99.62%	3.57%	63.3	1505	84.3%
**C56_1a**	143.9	135	46.48	97	95.53%	94.14%	3.09%	59.9	832	93.2%
**C56_1b**	145.2	135	48.44	97	95.92%	94.56%	3.09%	59.9	832	93.2%
**C56_1c**	147.4	140	55.57	98	96.27%	95.09%	3.09%	59.9	832	93.2%
**C56_2**	150.6	165	70.03	97	95.79%	94.68%	3.18%	63.7	897	93.1%
**C56_3**	153.0	140	73.62	97	96.16%	95.58%	3.15%	62.3	1545	84.0%
**C57_1a**	144.0	135	41.60	97	95.48%	93.23%	3.09%	59.9	832	93.2%
**C57_1b**	143.6	140	49.64	98	96.03%	94.40%	3.09%	59.9	832	93.2%
**C57_1c**	145.8	140	54.36	98	96.29%	94.92%	3.09%	59.9	832	93.2%
**C57_2**	153.6	165	63.98	98	95.96%	94.40%	3.18%	63.7	897	93.1%
**C57_3**	151.0	140	88.83	98	96.40%	95.84%	3.15%	62.3	1545	84.0%
**C58_1a**	147.3	135	57.07	97	95.99%	95.26%	3.09%	59.9	832	93.2%
**C58_1b**	145.8	140	56.00	98	96.12%	95.37%	3.09%	59.9	832	93.2%
**C58_1c**	147.3	145	59.00	98	96.18%	95.41%	3.09%	59.9	832	93.2%
**C58_2**	151.7	165	67.54	98	95.92%	95.07%	3.18%	63.7	897	93.1%
**C58_3**	150.5	150	78.02	98	96.14%	95.44%	3.15%	62.3	1545	84.0%
**C59_1a**	148.1	150	68.43	98	96.33%	95.71%	3.09%	59.9	832	93.2%
**C59_1b**	145.9	145	61.29	98	96.08%	95.39%	3.09%	59.9	832	93.2%
**C59_1c**	146.0	145	65.22	98	96.36%	95.68%	3.09%	59.9	832	93.2%
**C59_2**	149.5	170	89.89	98	96.23%	95.59%	3.18%	63.7	897	93.1%

**C59_3**	131.6	120	69.87	98	96.16%	95.39%	3.15%	62.3	1545	84.0%
**C61_1a**	145.4	135	43.38	97	95.21%	93.35%	3.09%	59.9	832	93.2%
**C61_1b**	144.2	135	39.37	97	94.92%	92.81%	3.09%	59.9	832	93.2%
**C61_1c**	140.6	130	46.32	97	95.45%	93.52%	3.09%	59.9	832	93.2%
**C61_2**	153.8	165	67.54	98	95.47%	93.80%	3.18%	63.7	897	93.1%
**C61_3**	142.0	140	98.13	98	96.46%	95.90%	3.15%	62.3	1545	84.0%
**C65_1**	142.0	135	49.72	98	89.63%	61.74%	3.09%	59.9	832	93.2%
**C65_2**	149.9	160	53.14	98	82.45%	50.04%	3.18%	63.7	897	93.1%
**C65_3a**	144.3	145	91.16	98	96.96%	96.62%	3.15%	62.3	1545	84.0%
**C65_3b**	129.4	135	83.08	98	96.87%	96.44%	3.15%	62.3	1545	84.0%
**C65_3c**	128.3	135	77.22	98	96.89%	96.43%	3.15%	62.3	1545	84.0%
**C67_1**	146.5	140	48.69	98	96.51%	95.78%	3.09%	59.9	832	93.2%
**C67_2a**	153.1	165	79.28	98	96.49%	95.90%	3.18%	63.7	897	93.1%
**C67_2b**	151.2	165	92.98	98	97.00%	96.63%	3.18%	63.7	897	93.1%
**C67_2c**	152.1	165	55.52	98	96.55%	95.69%	3.18%	63.7	897	93.1%
**C67_3**	146.5	150	102.98	98	96.72%	96.24%	3.15%	62.3	1545	84.0%
**C68_1**	144.7	135	52.37	98	95.90%	91.59%	3.09%	59.9	832	93.2%
**C68_2a**	148.8	165	73.50	98	95.94%	91.77%	3.18%	63.7	897	93.1%
**C68_2b**	154.0	165	82.35	98	96.63%	93.88%	3.18%	63.7	897	93.1%
**C68_2c**	152.6	160	59.00	98	95.37%	90.17%	3.18%	63.7	897	93.1%
**C68_3**	143.6	145	97.96	98	96.77%	96.33%	3.15%	62.3	1545	84.0%
**C69_1**	142.6	135	30.26	98	96.55%	94.81%	3.09%	59.9	832	93.2%
**C69_2a**	151.7	165	70.49	98	97.00%	96.56%	3.18%	63.7	897	93.1%
**C69_2b**	154.2	165	57.43	98	96.96%	96.35%	3.18%	63.7	897	93.1%
**C69_2c**	158.1	165	60.52	98	97.03%	96.50%	3.18%	63.7	897	93.1%
**C69_3**	147.2	140	63.40	98	96.87%	96.34%	3.15%	62.3	1545	84.0%
**C69_4**	169.6	170	95.89	98	97%	97%	2.95%	71.0	909	95.6%
**C72_1a**	136	155	54.32	86	89.54%	89.24%	3.51%	60.4	1612	79.1%
**C72_1b**	125.2	140	39.20	86	89.37%	88.93%	3.51%	60.4	1612	79.1%
**C72_1c**	133.3	155	54.21	86	89.52%	89.24%	3.51%	60.4	1612	79.1%
**C72_2**	116.1	115	45.34	86	89.45%	89.02%	4.74%	60.2	1693	76.1%
**C72_3**	124.4	170	71.23	86	89.62%	89.41%	3.43%	63.8	1172	89.7%
**C73_1a**	137.8	150	64.16	99	98.77%	98.74%	3.51%	60.4	1612	79.1%
**C73_1b**	124.8	135	65.68	99	98.77%	98.74%	3.51%	60.4	1612	79.1%
**C73_1c**	134.5	150	75.08	99	98.77%	98.74%	3.51%	60.4	1612	79.1%
**C73_2**	115	110	66.05	99	98.76%	98.70%	4.74%	60.2	1693	76.1%

**C73_3**	134.4	155	87.04	99	98.77%	98.74%	3.43%	63.8	1172	89.7%
**C74_1a**	138.4	145	62.01	97	98.31%	98.22%	3.51%	60.4	1612	79.1%
**C74_1b**	126.7	130	51.80	97	98.25%	98.00%	3.51%	60.4	1612	79.1%
**C74_1c**	135.6	150	80.72	97	98.33%	98.26%	3.51%	60.4	1612	79.1%
**C74_2**	116.3	105	59.14	97	98.27%	98.10%	4.74%	60.2	1693	76.1%
**C74_3**	134.5	155	115.80	97	98.34%	98.28%	3.43%	63.8	1172	89.7%
**C75_1a**	135.1	150	87.92	92	92.85%	92.78%	3.51%	60.4	1612	79.1%
**C75_1b**	124	135	54.58	92	92.78%	92.68%	3.51%	60.4	1612	79.1%
**C75_1c**	122.9	130	48.50	92	92.77%	92.59%	3.51%	60.4	1612	79.1%
**C75_2**	115.4	110	46.82	92	92.72%	92.41%	4.74%	60.2	1693	76.1%
**C75_3**	135.1	155	177.33	92	92.91%	92.86%	3.43%	63.8	1172	89.7%
**C76_1a**	133.4	150	55.24	93	93.85%	93.70%	3.51%	60.4	1612	79.1%
**C76_1b**	125.8	135	66.23	93	93.87%	93.74%	3.51%	60.4	1612	79.1%
**C76_1c**	132.8	150	65.32	93	93.88%	93.75%	3.51%	60.4	1612	79.1%
**C76_2**	116.6	115	52.05	93	93.82%	93.58%	4.74%	60.2	1693	76.1%
**C76_3**	134.3	115	112.71	93	93.92%	93.84%	3.43%	63.8	1172	89.7%
**C103_1**	138.5	180	85.05	85	85.68%	85.47%	3.67%	60.7	1272	88.9%
**C103_2a**	156.8	210	132.67	85	85.79%	85.65%	2.88%	68.7	1149	91.2%
**C103_2b**	154.5	205	121.21	85	85.77%	85.63%	2.88%	68.7	1149	91.2%
**C103_2c**	165.6	190	126.14	85	85.77%	85.62%	2.88%	68.7	1149	91.2%
**C103_3**	148	185	110.66	85	85.72%	85.56%	3.15%	62.3	1545	84.0%
**C104_1**	137.9	185	134.95	90	91.95%	91.80%	3.67%	60.7	1272	88.9%
**C104_2a**	160.6	215	127.98	90	92.03%	91.86%	2.88%	68.7	1149	91.2%
**C104_2b**	157.7	215	126.83	90	92.05%	91.89%	2.88%	68.7	1149	91.2%
**C104_2c**	170.8	220	127.20	90	92.06%	91.86%	2.88%	68.7	1149	91.2%
**C104_3**	145.7	190	141.78	90	91.96%	91.78%	3.15%	62.3	1545	84.0%
**C105_1**	130.4	150	70.47	95	99.96%	99.82%	3.67%	60.7	1272	88.9%
**C105_2a**	121.7	160	101.10	95	99.98%	99.93%	2.88%	68.7	1149	91.2%
**C105_2b**	158.4	160	63.65	95	99.92%	99.64%	2.88%	68.7	1149	91.2%
**C105_2c**	158.2	160	72.61	95	99.95%	99.78%	2.88%	68.7	1149	91.2%
**C105_3**	150.1	160	65.83	95	99.93%	99.70%	3.15%	62.3	1545	84.0%
**C106_1**	127.1	140	71.55	95	93.82%	93.24%	3.67%	60.7	1272	88.9%
**C016_2a**	153.6	185	97.31	95	94.28%	93.90%	2.88%	68.7	1149	91.2%
**C106_2b**	161.3	180	68.14	95	94.18%	93.66%	2.88%	68.7	1149	91.2%
**C106_2c**	158.8	180	102.22	95	94.44%	94.05%	2.88%	68.7	1149	91.2%
**C106_3**	144.3	165	82.85	95	94.05%	93.54%	3.15%	62.3	1545	84.0%

Appendix 9. Detection of antibiotic resistance genes from WGS data

Open ResFinder webpage (https://cge.cbs.dtu.dk/services/ResFinder/) at Center for Genome Epidemiology.

Select all antimicrobials, 100% threshold for %ID and 60% minimum length.

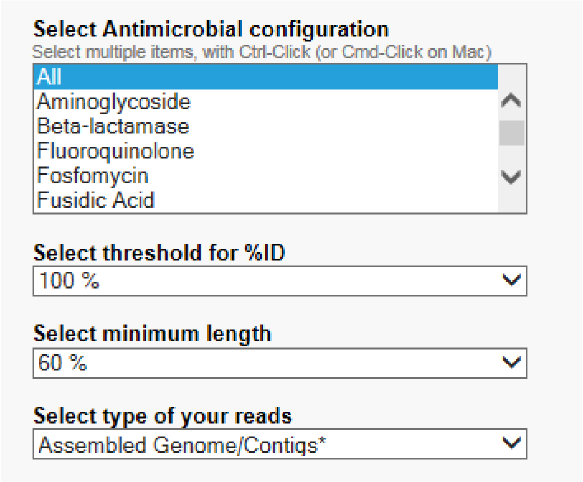

Use either *de novo* assembled contigs in fasta format or raw paired reads in fastq forma.

Results

Positive controls

C50 Pseudomonas aeruginosa ATCC 27853. According to CLSI M100-S24 document “contains inducible AmpC β-lactamase”

ResFinder-2.1 Server - Results

Aminoglycoside
No resistance genes found.

Beta-lactam						
Resistance gene	%Identity	Query/HSP length	Contig	Position in contig	Predicted phenotype	Accession number
blaOXA-50	100.00	789 / 789	C50_1a_trimmed_(paired)_contig_8	162091..162879	Beta-lactam resistance	AY306135

Fluoroquinolone
No resistance genes found.

Fosfomycin
No resistance genes found.

Fusidic Acid
No resistance genes found.

MLS - Macrolide, Lincosamide and Streptogramin B
No resistance genes found.

Nitroimidazole
No resistance genes found.

Oxazolidinone
No resistance genes found.

Phenicol
No resistance genes found.

Rifampicin
No resistance genes found.

Sulphonamide
No resistance genes found.

Tetracycline
No resistance genes found.

Trimethoprim
No resistance genes found.

Glycopeptide
No resistance genes found.

extended output

Results as text | Results tab separated | Hit in genome sequences | Resistance gene sequences

Selected %ID threshold: *100.00 %*

Selected minimum length: *60 %*

Input Files: *C50_1a_trimmed_assembly.fa*

C103 Bacteroides fragilis ATCC 25285. According to CLSI M100-S24 document is “β-lactamase positive”

ResFinder-2.1 Server - Results

Aminoglycoside
No resistance genes found.

Beta-lactam						
Resistance gene	%Identity	Query/HSP length	Contig	Position in contig	Predicted phenotype	Accession number
cepA	100.00	903 / 903	C103_1_trimmed_(paired)_contig_11	178605..179507	Beta-lactam resistance	U05887

Fluoroquinolone
No resistance genes found.

Fosfomycin
No resistance genes found.

Fusidic Acid
No resistance genes found.

MLS - Macrolide, Lincosamide and Streptogramin B
No resistance genes found.

Nitroimidazole
No resistance genes found.

Oxazolidinone
No resistance genes found.

Phenicol
No resistance genes found.

Rifampicin
No resistance genes found.

Sulphonamide
No resistance genes found.

Tetracycline
No resistance genes found.

Trimethoprim
No resistance genes found.

Glycopeptide
No resistance genes found.

extended output

Results as text | Results tab separated | Hit in genome sequences | Resistance gene sequences

Selected %ID threshold: *100.00 %*

Selected minimum length: *60 %*

Input Files: *C103_1_trimmed_assembly.fa*

Negative controls

C5 Staphylococcus aureus ATCC 25923. According to CLSI M100-S24 document strain is susceptible.

ResFinder-2.1 Server - Results

Aminoglycoside
No resistance genes found.

Beta-lactam
No resistance genes found.

Fluoroquinolone
No resistance genes found.

Fosfomycin
No resistance genes found.

Fusidic Acid
No resistance genes found.

MLS - Macrolide, Lincosamide and Streptogramin B
No resistance genes found.

Nitroimidazole
No resistance genes found.

Oxazolidinone
No resistance genes found.

Phenicol
No resistance genes found.

Rifampicin
No resistance genes found.

Sulphonamide
No resistance genes found.

Tetracycline
No resistance genes found.

Trimethoprim
No resistance genes found.

Glycopeptide
No resistance genes found.

extended output

Results as text | Results tab separated | Hit in genome sequences | Resistance gene sequences

Selected %ID threshold: *100.00 %*

Selected minimum length: *60 %*

Input Files: *C5_2_assembly.fa*

C55 *Escherichia coli* ATCC 25922. According to CLSI M100-S24 document strain is susceptible.

ResFinder-2.1 Server - Results

Aminoglycoside

No resistance genes found.

Beta-lactam

No resistance genes found.

Fluoroquinolone

No resistance genes found.

Fosfomycin

No resistance genes found.

Fusidic Acid

No resistance genes found.

MLS - Macrolide, Lincosamide and Streptogramin B

No resistance genes found.

Nitroimidazole

No resistance genes found.

Oxazolidinone

No resistance genes found.

Phenicol

No resistance genes found.

Rifampicin

No resistance genes found.

Sulphonamide

No resistance genes found.

Tetracycline

No resistance genes found.

Trimethoprim

No resistance genes found.

Glycopeptide

No resistance genes found.

extended output

Results as text | Results tab separated | Hit in genome sequences | Resistance gene sequences

Selected %ID threshold: *100.00 %*

Selected minimum length: *60 %*

Input Files: *C55_3c_assembly.fa*

C46 Enterococcus faecalis 29212. According to CLSI M100-S24 document strain is susceptible.

ResFinder-2.1 Server - Results

Aminoglycoside
No resistance genes found.

Beta-lactam
No resistance genes found.

Fluoroquinolone
No resistance genes found.

Fosfomycin
No resistance genes found.

Fusidic Acid
No resistance genes found.

MLS - Macrolide, Lincosamide and Streptogramin B
No resistance genes found.

Nitroimidazole
No resistance genes found.

Oxazolidinone
No resistance genes found.

Phenicol
No resistance genes found.

Rifampicin
No resistance genes found.

Sulphonamide
No resistance genes found.

Tetracycline
No resistance genes found.

Trimethoprim
No resistance genes found.

Glycopeptide
No resistance genes found.

extended output

Results as text | Results tab separated | Hit in genome sequences | Resistance gene sequences

Selected %ID threshold: *100.00 %*

Selected minimum length: *60 %*

Input Files: *C46_1_assembly_CLC.fa*

Appendix 10. MLST reproducibility and repeatability validation results

Each biological sample has 5 replicates. One of the replicates for each sample was used to estimate the accuracy of the MLST assay. Refer to corresponding results log document.

Replicates used in accuracy evaluation of MLST		MLST results for other replicates			
**Sample**	**ST**	**Between-run-2**	**Between-run-3**	**Within-run-2**	**Within-run-3**
C1_2	ST-11	ST-11	ST-11	ST-11	ST-11
C2_1	ST-1	ST-1	ST-1	ST-1	ST-1
C3_2a	ST-3021	ST-3021	ST-3021	ST-3021	ST-3021
C4_2a	ST-1	ST-1	ST-1	ST-1	ST-1
C5_2	ST-243	ST-243	ST-243	ST-243	ST-243
C6_3a	ST-19	ST-19	ST-19	ST-19	ST-19
C46_1	ST-30	ST-30	ST-30	ST-30	ST-30
C47_1c	ST-8	ST-8	ST-8	ST-8	ST-8
C49_1	ST-4840	ST-4840	ST-4840	ST-4840	ST-4840
C50_1a	ST-155	ST-155	ST-155	ST-155	ST-155
C51_3a	ST-14	ST-14	ST-14	ST-14	ST-14
C53_1	ST-98	ST-98	ST-98	ST-98	ST-98
C54_1	ST-836	ST-836	ST-836	ST-836	ST-836
C55_3c	ST-73	ST-73	ST-73	ST-73	ST-73
C73_1a	ST-11	ST-11	ST-11	ST-11	ST-11
C74_1a	ST-32	ST-32	ST-32	ST-32	ST-32
C75_1a	ST-440	ST-440	ST-440	ST-440	ST-440
C76_1a	ST-592	ST-592	ST-592	ST-592	ST-592
C72_1a	ST-655	ST-655	ST-655	ST-655	ST-655
C104_1	ST-44	ST-44	ST-44	ST-44	ST-44
C106_1	ST-11075	ST-11075	ST-11075	ST-11075	ST-11075

All *in silico* MLST results for replicates are documented in a separate report.

Appendix 11. 16S rRNA reproducibility and repeatability validation results

Each biological sample has 5 replicates. One of the replicates for each sample was used to estimate the accuracy of the 16S rRNA ID assay. Refer to corresponding results log document.

Replicates used in accuracy evaluation for 16S rRNA ID assay		16S ID results for other replicates			
**Sample**	**Consensus ID**	**Between-run-2**	**Between-run-3**	**Within-run-2**	**Within-run-3**
C1_2	*Escherichia coli*	*Escherichia coli*	*Escherichia coli*	*Escherichia coli*	*Escherichia coli*
C2_1	*Aeromonas hydrophila*	*Aeromonas hydrophila*	*Aeromonas hydrophila*	*Aeromonas hydrophila*	*Aeromonas hydrophila*
C3_2a	*Escherichia coli*	*Escherichia coli*	*Escherichia coli*	*Escherichia coli*	*Escherichia coli*
C4_2a	*Enterobacter cloacae*	*Enterobacter cloacae*	*Enterobacter cloacae*	*Enterobacter cloacae*	*Enterobacter cloacae*
C5_2	*Staphylococcus aureus*	*Staphylococcus aureus*	*Staphylococcus aureus*	*Staphylococcus aureus*	*Staphylococcus aureus*
C6_3a	*Salmonella enterica*	*Salmonella enterica*	*Salmonella enterica*	*Salmonella enterica*	*Salmonella enterica*
C46_1	*Enterococcus faecalis*	*Enterococcus faecalis*	*Enterococcus faecalis*	*Enterococcus faecalis*	*Enterococcus faecalis*
C47_1c	*Staphylococcus epidermidis*	*Staphylococcus epidermidis*	*Staphylococcus epidermidis*	*Staphylococcus epidermidis*	*Staphylococcus epidermidis*
C48_1a	*Staphylococcus saprophyticus*	*Staphylococcus saprophyticus*	*Staphylococcus saprophyticus*	*Staphylococcus saprophyticus*	*Staphylococcus saprophyticus*
C49_1	*Streptococcus pneumoniae*	*Streptococcus pneumoniae*	*Streptococcus pneumoniae*	*Streptococcus pneumoniae*	*Streptococcus pneumoniae*
C50_1a	*Pseudomonas aeruginosa*	*Pseudomonas aeruginosa*	*Pseudomonas aeruginosa*	*Pseudomonas aeruginosa*	*Pseudomonas aeruginosa*
C51_3a	*Stenotrophomonas maltophilia*	*Stenotrophomonas maltophilia*	*Stenotrophomonas maltophilia*	*Stenotrophomonas maltophilia*	*Stenotrophomonas maltophilia*
C52_1b	*Legionella pneumophila*	*Legionella pneumophila*	*Legionella pneumophila*	*Legionella pneumophila*	*Legionella pneumophila*
C53_1	*Moraxella catarrhalis*	*Moraxella catarrhalis*	*Moraxella catarrhalis*	*Moraxella catarrhalis*	*Moraxella catarrhalis*
C54_1	*Acinetobacter baumannii*	*Acinetobacter baumannii*	*Acinetobacter baumannii*	*Acinetobacter baumannii*	*Acinetobacter baumannii*
C55_3c	*Escherichia coli*	*Escherichia coli*	*Escherichia coli*	*Escherichia coli*	*Escherichia coli*
C72_1a	*Escherichia coli*	*Escherichia coli*	*Escherichia coli*	*Escherichia coli*	*Escherichia coli*
C73_1a	*Salmonella enterica*	*Salmonella enterica*	*Salmonella enterica*	*Salmonella enterica*	*Salmonella enterica*
C74_1a	*Salmonella enterica*	*Salmonella enterica*	*Salmonella enterica*	*Salmonella enterica*	*Salmonella enterica*
C75_1a	*Salmonella enterica*	*Salmonella enterica*	*Salmonella enterica*	*Salmonella enterica*	*Salmonella enterica*
C76_1a	*Salmonella enterica*	*Salmonella enterica*	*Salmonella enterica*	*Salmonella enterica*	*Salmonella enterica*
C103_1	*Bacteroides fragilis*	*Bacteroides fragilis*	*Bacteroides fragilis*	*Bacteroides fragilis*	*Bacteroides fragilis*
C104_1	*Haemophilus influenzae*	*Haemophilus influenzae*	*Haemophilus influenzae*	*Haemophilus influenzae*	*Haemophilus influenzae*
C105_1	*Corynebacterium jeikeium*	*Corynebacterium jeikeium*	*Corynebacterium jeikeium*	*Corynebacterium jeikeium*	*Corynebacterium jeikeium*
C106_1	*Neisseria gonorrhoeae*	*Neisseria gonorrhoeae*	*Neisseria gonorrhoeae*	*Neisseria gonorrhoeae*	*Neisseria gonorrhoeae*
C56_1a	*Mycobacterium tuberculosis*	*Mycobacterium tuberculosis*	*Mycobacterium tuberculosis*	*Mycobacterium tuberculosis*	*Mycobacterium tuberculosis*
C57_1a	*Mycobacterium tuberculosis*	*Mycobacterium tuberculosis*	*Mycobacterium tuberculosis*	*Mycobacterium tuberculosis*	*Mycobacterium tuberculosis*
C58_1a	*Mycobacterium tuberculosis*	*Mycobacterium tuberculosis*	*Mycobacterium tuberculosis*	*Mycobacterium tuberculosis*	*Mycobacterium tuberculosis*
C59_1a	*Mycobacterium tuberculosis*	*Mycobacterium tuberculosis*	*Mycobacterium tuberculosis*	*Mycobacterium tuberculosis*	*Mycobacterium tuberculosis*

C61_1a	*Mycobacterium tuberculosis*	*Mycobacterium tuberculosis*	*Mycobacterium tuberculosis*	*Mycobacterium tuberculosis*	*Mycobacterium tuberculosis*
C65_1	*Mycobacterium tuberculosis*	*Mycobacterium tuberculosis*	*Mycobacterium tuberculosis*	*Mycobacterium tuberculosis*	*Mycobacterium tuberculosis*
C67_1	*Mycobacterium tuberculosis*	*Mycobacterium tuberculosis*	*Mycobacterium tuberculosis*	*Mycobacterium tuberculosis*	*Mycobacterium tuberculosis*
C68_1	*Mycobacterium tuberculosis*	*Mycobacterium tuberculosis*	*Mycobacterium tuberculosis*	*Mycobacterium tuberculosis*	*Mycobacterium tuberculosis*
C69_1	*Mycobacterium tuberculosis*	*Mycobacterium tuberculosis*	*Mycobacterium tuberculosis*	*Mycobacterium tuberculosis*	*Mycobacterium tuberculosis*

All 16S rRNA ID results for replicates are documented in a separate report.

Appendix 12. Metrics of the negative controls collected during validation

Number of the reads after trimming for negative controls = 10-3921 (median 785)

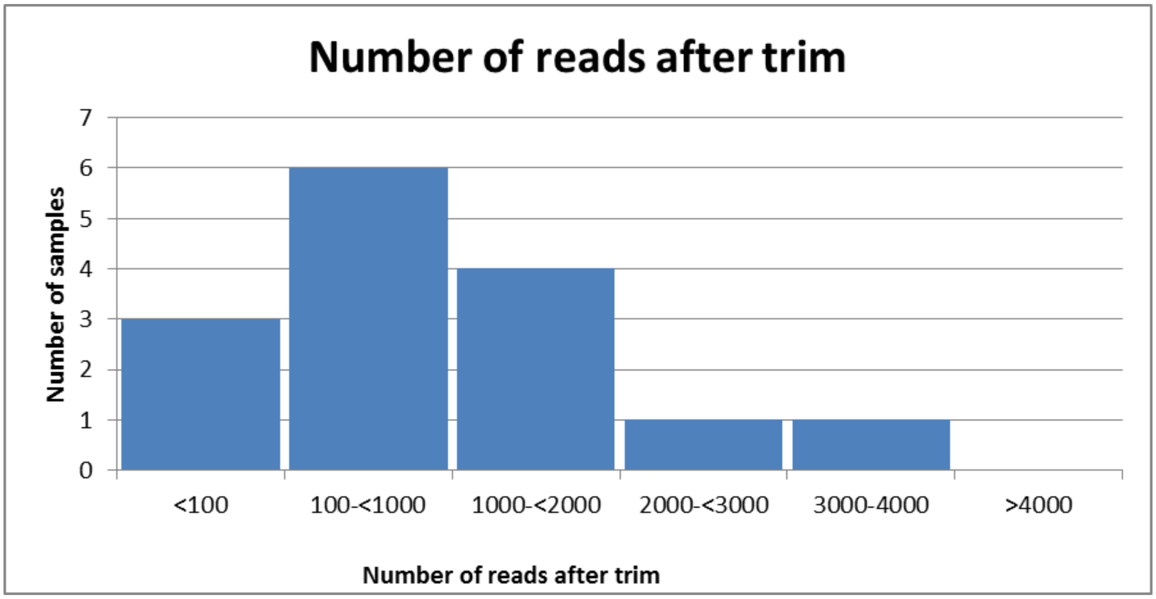

N50 for *de novo* assembled negative control reads = 238-498 (median 312)

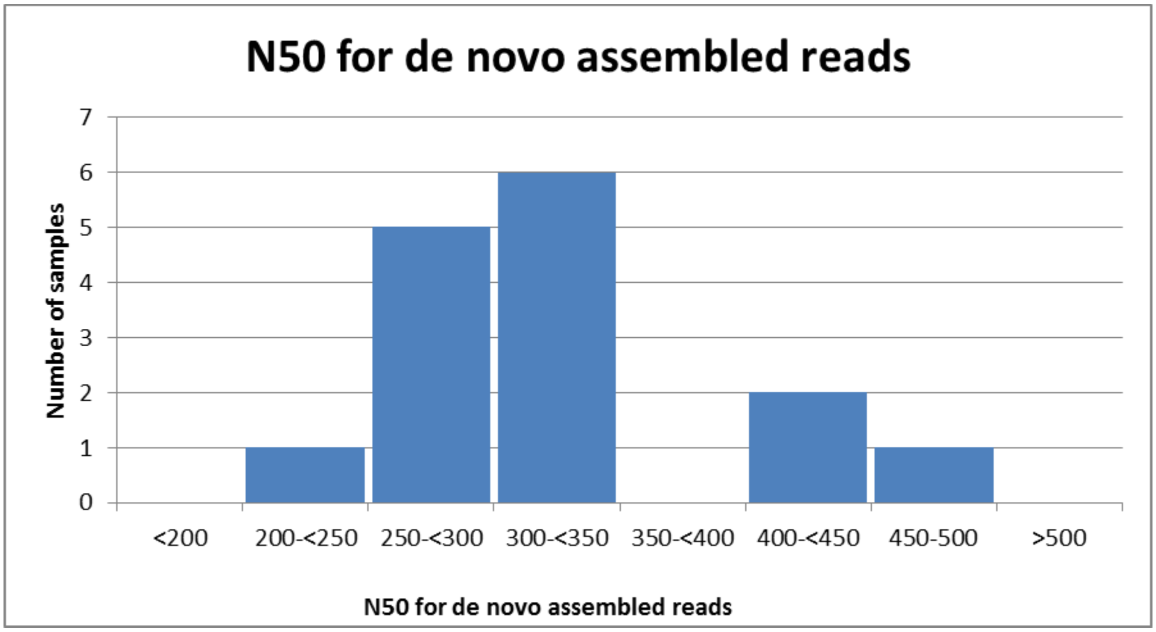

The highest coverage of *de novo* contigs assembled from negative control samples= 1.94-9.96 (median 3.43)

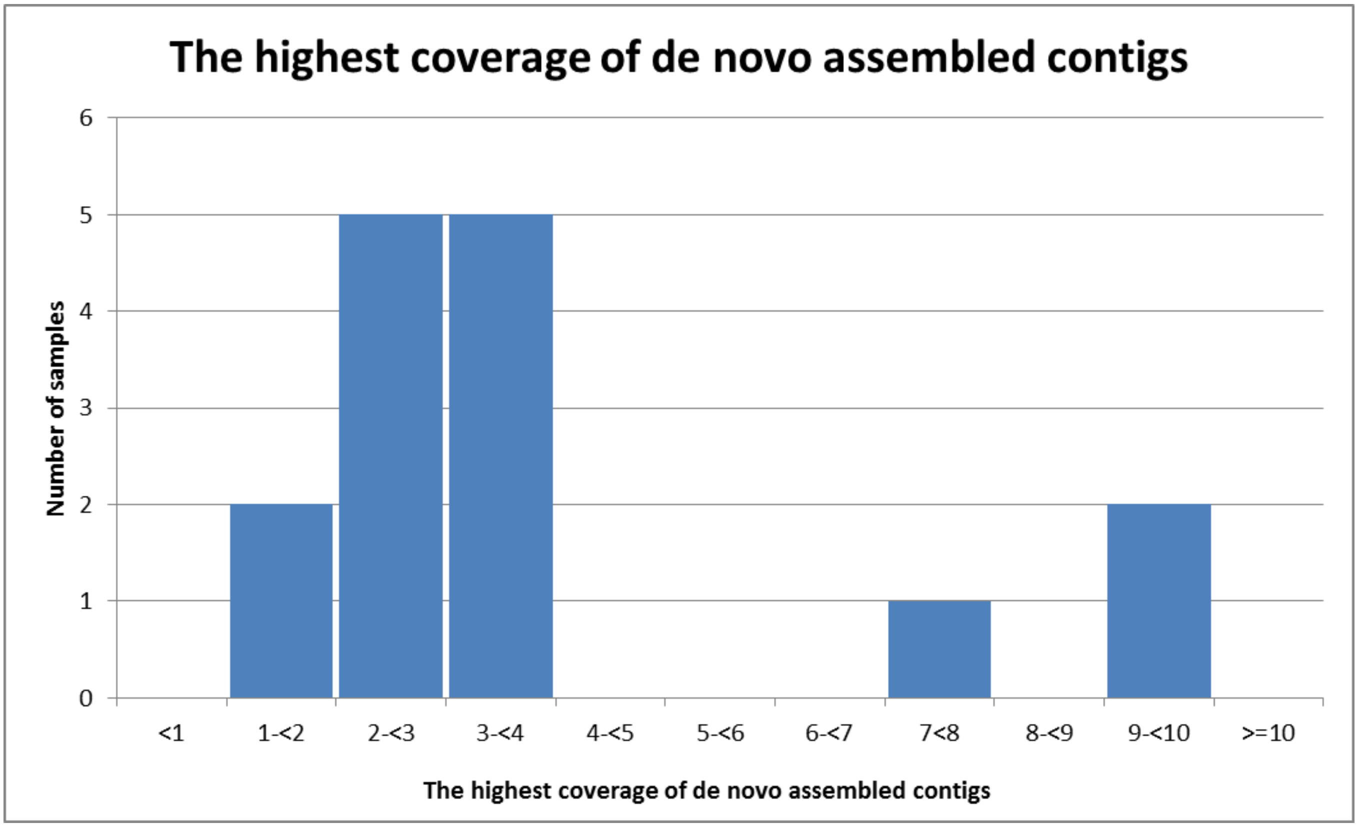

For all negative controls following parameters were met:

- Number of reads after trimming remained <10,000
- N50 for *de novo* assembled reads was < 1,000
- The highest coverage of *de novo* assembled contigs was < 10x

Appendix 13. VirulenceFinder example of the output for positive *E.coli* ATCC 25922 control

Analysis settings:

VirulenceFinder-1.5 (https://cge.cbs.dtu.dk/services/VirulenceFinder/)

Database- E.coli

Analysis file- fasta (assembled genome/contigs)

Selected %ID threshold: 90.00 %

Selected minimum length: 60 %

VirulenceFinder-1.5 Server - Results

SETTINGS:
Selected %ID threshold: *90.00*

Virulence - E. coli

Virulence factor	%Identity	Query/HSP length	Contig	Position in contig	Protein function	Accession number
mchB	100.00	294 / 294	C55_S15_L001_R1_001_15_(paired)_trimmed_(paired)_contig_26	10413..10706	Microcin H47 part of colicin H	AE014075
mchC	100.00	1551 / 1551	C55_S15_L001_R1_001_15_(paired)_trimmed_(paired)_contig_26	10978..12528	MchC protein	AE014075
iss	100.00	294 / 294	C55_S15_L001_R1_001_15_(paired)_trimmed_(paired)_contig_38	11839..12132	Increased serum survival	CP002167
mchF	100.00	2115 / 2115	C55_S15_L001_R1_001_15_(paired)_trimmed_(paired)_contig_26	14408..16522	ABC transporter protein MchF	AE014075
mcmA	100.00	279 / 279	C55_S15_L001_R1_001_15_(paired)_trimmed_(paired)_contig_26	16776..17054	Microcin M part of colicin H	AJ586887
iha	100.00	2091 / 2091	C55_S15_L001_R1_001_15_(paired)_trimmed_(paired)_contig_40	18099..20189	Adherence protein	AE014075
sat	100.00	3888 / 3888	C55_S15_L001_R1_001_15_(paired)_trimmed_(paired)_contig_40	25685..29572	Secreted autotransporter toxin	AE014075
vat	100.00	4131 / 4131	C55_S15_L001_R1_001_15_(paired)_trimmed_(paired)_contig_12	28136..32266	Vacuolating autotransporter toxin	AE014075
iroN	100.00	2178 / 2178	C55_S15_L001_R1_001_15_(paired)_trimmed_(paired)_contig_26	31819..33996	Enterobactin siderophore receptor protein	AE014075
pic	99.98	4116 / 4116	C55_S15_L001_R1_001_15_(paired)_trimmed_(paired)_contig_27	3239..7354	serine protease autotransporters of Enterobacteriaceae (SPATE)	AE014075

stx - Holotoxins

No virulence factors found.

- All virulence genes *mchB, mchC, iss, mchF, mcmA, iha, sat, vat,* and *iroN* are deleted with 100% ID and 100% query length coverage.
- Gene *pic* has ID <100% therefore is ignored.

Appendix 14. Resolution of the discrepancies between validation base calling results and reference sequences with Sanger sequencing

Samples selected for the confirmatory testing

Sample	Microorganisms	Total # of SNP difference with the reference	# of sequencing errors (SNP is supported only by 4 or less validation replicates)	Reference sequence	Targeted regions sequenced by Sanger	Number of SNPs confirmed by Sanger sequencing
C1	*Escherichia coli* O157:H7 CDC EDL 933	5	0	NZ_CP008957.1	5 SNP/2 sites-repetitive regions*	NA
C2	*Aeromonas hydrophilia* ATCC 7966	1	0	NC_008570	1 SNP/1 site	1
C46	*Enterococcus faecalis* ATCC 29212	3	0	NZ_CP008816	3 SNPs/3 sites	3
C47	*Staphylococcus epidermidis* ATCC 12228	184	2	NC_004461	region with discrepancies between replicates is a repetitive sequence*	NA
C52	*Legionella pneumophila* SG-12 ATCC 43290	2	0	NC_016811	2 SNPs/2 sites	2
C55	*Escherichia coli* ATCC 25922	14	1	NZ_CP009072.1	repetitive region*	NA

*Was impossible to design specific primers within the reach of Sanger sequencing read length because SNPs were located in the vast repeat regions.

Sanger sequencing results

Sample C2:

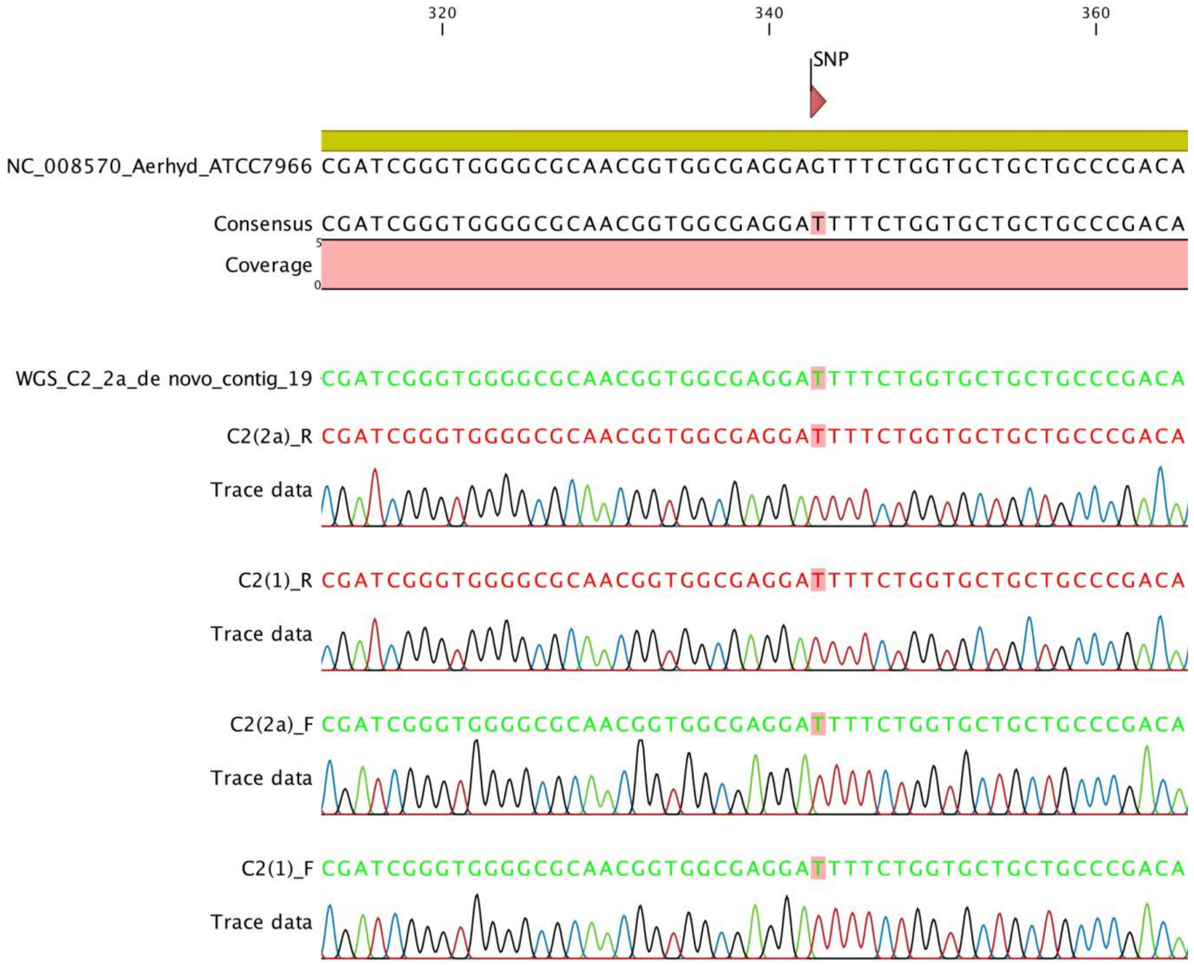

Previous mapping result: G -> T in all 5 replicates.

WGS_C2_2a_de_novo_contig_19- validation sample C2 sequenced by the laboratory and assembled *de novo*.

Sample C46:

SNP position #1

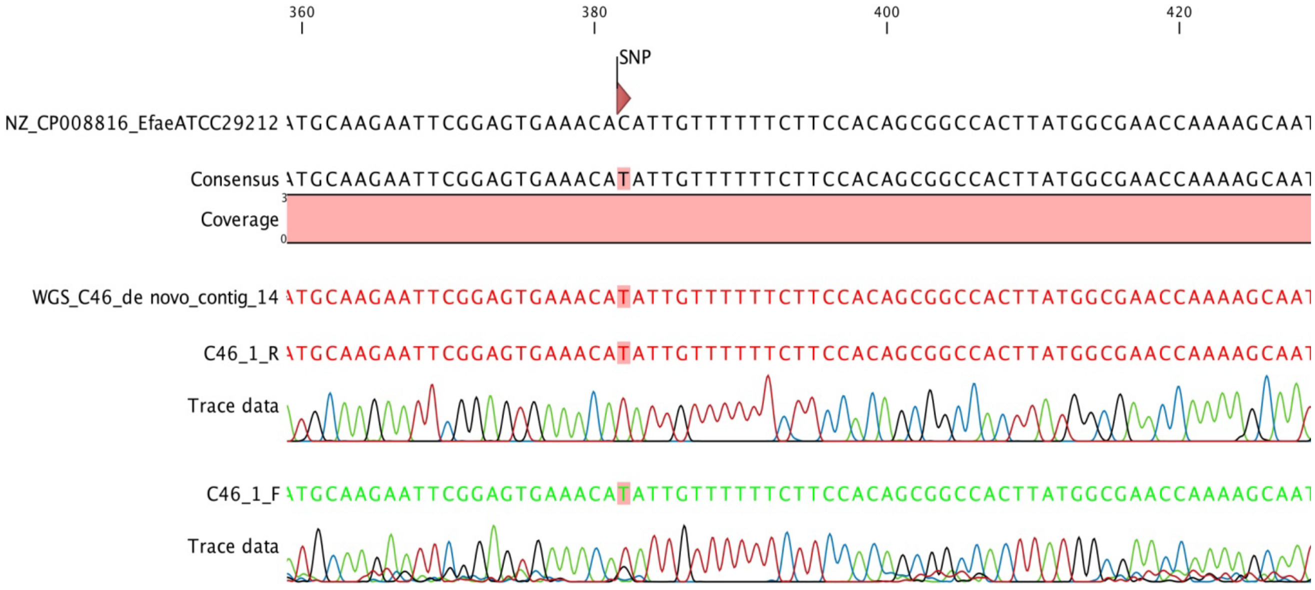

Previous mapping result: C -> T in all 5 replicates.

WGS_C46_de_novo_contig_14- validation sample C46 sequenced by the laboratory and assembled *de novo*.

SNP positions ##2 and 3

The regions represent a sequence which is repeated twice in the genome *of Enterococcus faecalis* ATCC 29212. Both repeats had the same SNP between the validation sequence and the reference (NCBI) sequence upon mapping. In the de novo assembled validation sequence 2 contigs can be found: one- matching the SNP in the reference (A), and another- matching the mapping results for all 5 replicates and confirmed by Sanger sequencing (T). In this case errors in de novo assembly as well as

existence of two repeat variants is possible and cannot be resolved with short-read technology.

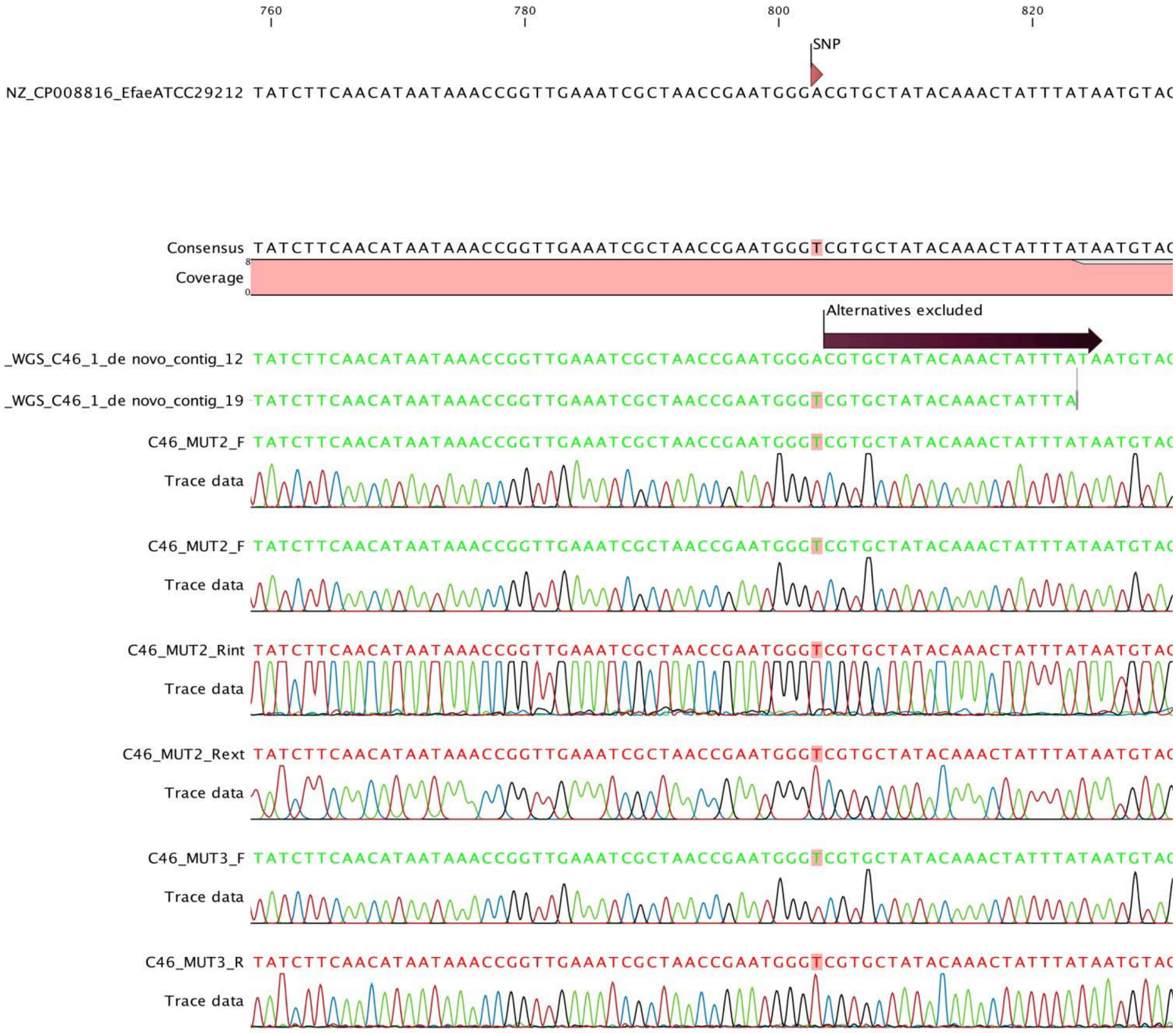

Previous mapping result: A -> T in all 5 replicates.

WGS_C46_1_de_novo_contig_12 and WGS_C46_1_de_novo_contig_19- two contigs belonging to the same *de novo* assembly of the validation sample C46.

Sample C52:

<u>SNP position #1</u>

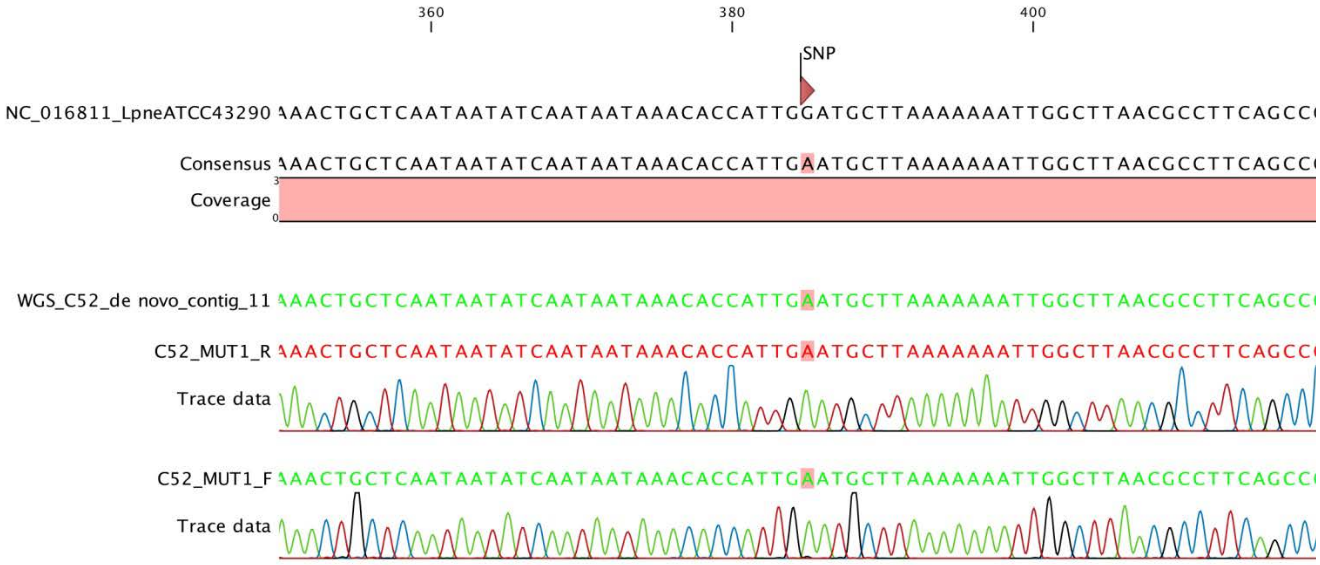

Previous mapping result: G -> A in all 5 replicates.

WGS_C52_de_novo_contig_11 validation sample C52 sequenced by the laboratory and assembled *de novo*.

SNP position #2

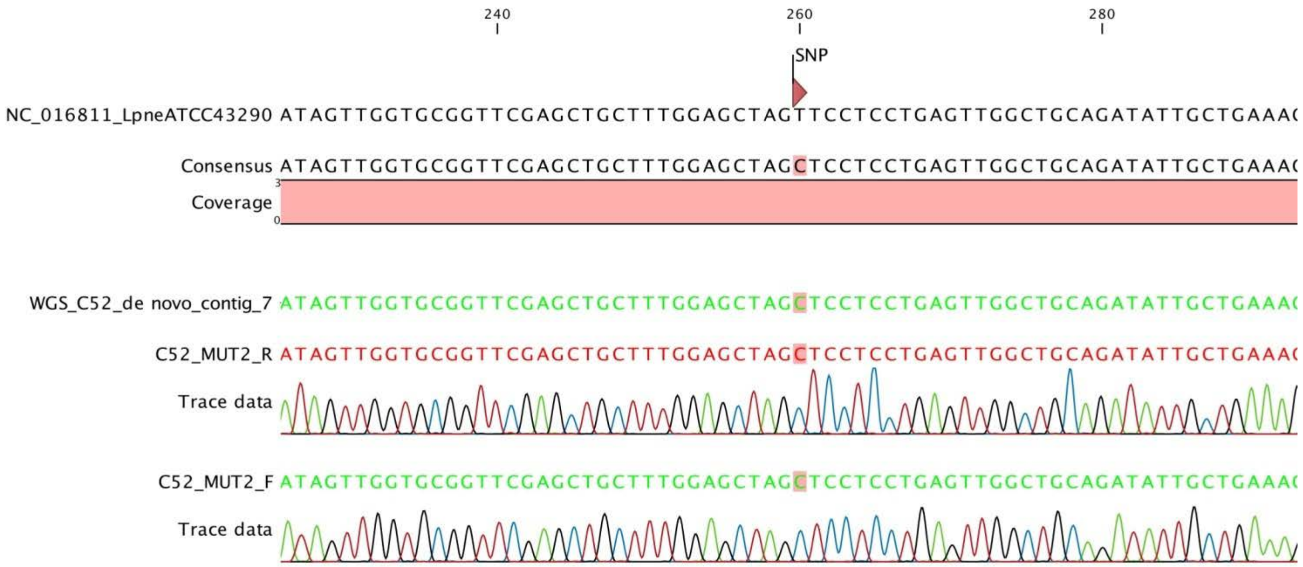

Previous mapping result: T -> C in all 5 replicates.

WGS_C52_de_novo_contig_7 validation sample C52 sequenced by the laboratory and assembled *de novo*.

Summary

Sample	SNP position number	Reference	Mapping	De novo	Sanger
C2	1	G	T	T	T
C46	1	C	T	T	T
	2	A	T	A/T	T
	3	A	T	A/T	T
C52	1	G	A	A	A
	2	T	C	C	C

Conclusion

Tested discrepancies between validation sequences and NCBI reference were confirmed by Sanger sequencing. Mapping to repetitive regions should be avoided when possible.

Appendix 15. Comparison of the antibiotic resistance genes detection with PCR-based methods and ResFinder for the isolates from CDC/FDA AR Isolate Bank

Bank sample ID	Organism	Analyzed by	PCR-based detection results	Total Number AR Genes	False Positive	False Negative	Results in agrement	Beta-lactamases	Colistin	Aminoglycoside	Fluoroquinolones	Fosfomycin	Macrolide-Lincosamide-Streptogramin	Phenicols	Rifampicin	Sulfonamides	Tetracyclines	Trimethoprim	BioSample	SRA file name
32	Enterobacter cloacae	CDC results	KPC 3, TEM-1	10				KPC-3,TEM-1B,ACT-16		strB,aac(6')-IIc,strA	QnrB2					sul1,sul2		dfrA18	SAMN04014873	SRR3242004
		**Validation results**	blaTEM-1B, blaKPC-3	11	1	0	2111	blaACT-16, blaTEM-1B, blaKPC-3		aac(6')-IIc, strB, strA, **aph(3')-Ia**	QnrB2					sul1, sul2		dfrA18		
46	Klebsiella pneumoniae	CDC results	VIM	14				VIM-27,CTX-M-15,SHV-11,OXA-1		strB,aph(3')-Ia,strA,aac(3)-IIa,aac(6')Ib-cr	oqxA		mph(A)			sul1	tet(A)	dfrA1	SAMN04014887	SRR3112314
		**Validation results**	blaVIM-27	14	0	0	2112	blaSHV-11, blaCTX-M-15, blaVIM-27, blaOXA-1		aph(3')-Ia, aac(3)-IIa, strA, strB, aac(6')Ib-cr	oqxA		mph(A)			sul1	tet(A)	dfrA1		
71	Klebsiella Oxytoca	CDC results	CRE (-)	1				OXY-2-8											SAMN04014912	SRR3242014
		**Validation results**	no CRE detected	1	0	0	2112	blaOXY-2-8												
79	Klebsiella pneumoniae	CDC results	CTX-M14; DHA-1 [OMPF/ OMPK35][1]	16				TEM-1B,CTX-M-14,SHV-11,DHA-1		strB,aadA2,strA,aac(3)-IId,rmtB	oqxB,QnrB4, oqxA		erm(42)			sul2	tet(G)	dfrA12	SAMN04014920	SRR3112327
		**Validation results**	blaDHA-1, blaCTX-M-14	16	0	0	2112	blaDHA-1, blaTEM-1B, blaCTX-M-14, blaSHV-11		rmtB, aac(3)-IId, strA, strB, aadA2	QnrB4, oqxB, oqxA		erm(42)			sul2	tet(G)	dfrA12		
82	Providencia rettgeri	CDC results	NDM	4				NDM-1		aadA2						sul1		dfrA12	SAMN04014923	SRR3290652
		**Validation results**	blaNDM-1	4	0	0	2112	blaNDM-1		aadA2						sul1		dfrA12		
91	Serratia marcescens	CDC results	SME	2				SME-3		aac(6')-Ic									SAMN04014932	SRR3242023
		**Validation results**	blaSME-3	3	1	0	2111	**blaSRT-2**, blaSME-3		aac(6')-Ic										
92	Pseudomonas aeruginosa	CDC results	IMP-14;	10				IMP-14,OXA-50,VEB-1,PAO,OXA-10		aadB,aac(3)-Ic,aadA6				cmlA1			tet(G)		SAMN04014933	SRR3112336
		**Validation results**	blaIMP-14	11	1	0	2111	blaPAO, blaOXA-50, blaIMP-14, blaVEB-1, blaOXA-10		aadB, aac(3)-Ic, aadA6		fosA		cmlA1		**sul1**	tet(G)			
215	Staphylococcus aureus	CDC results	NA	5				mecA,blaZ		aadD,spc			erm(A)						SAMN04901605	SRR4417439
		**Validation results**		5	0	0	2112	mecA, blaZ		aadD, spc			erm(A)							
216	Staphylococcus aureus	CDC results	NA	4				mecA		aph(3')-III			mph(C), ***msr(A)***[2]						SAMN04901606	SRR4417440
		**Validation results**		4	1	1	2110	**blaZ**, mecA		aph(3')-III			mph(C)							
217	Staphylococcus aureus	CDC results	NA	3				mecA,blaZ										dfrG	SAMN04901607	SRR4417445
		**Validation results**		3	0	0	2112	mecA, blaZ										dfrG		
218	Staphylococcus aureus	CDC results	NA	5				***mecA***[3]		aph(3')-III,spc			erm(A)				tet(K)		SAMN04901608	SRR4417446
		**Validation results**		5	1	1	2110	**blaZ**		aph(3')-III, spc			erm(A)				tet(K)			
219	Staphylococcus aureus	CDC results	NA	6				mecA		aadD,spc,aac(6')-aph(2'')			erm(A)				tet(M)		SAMN04901609	SRR4417447
		**Validation results**		6	0	0	2112	mecA		aadD, spc, aac(6')-aph(2'')			erm(A)				tet(M)			
221	Staphylococcus aureus	CDC results	NA	3				mecA		aac(6')-aph(2'')							tet(M)		SAMN04901611	SRR4417449
		**Validation results**		4	1	0	2111	mecA, **blaZ**		aac(6')-aph(2'')							tet(M)			

1- ResFinder does not have ability to detect the truncation of porin genes; therefore porin-related resistance mechanisms mentioned in the CDC database could not be detected with ResFinder.

2- msr(A) gene was detected at 98.8% ID and was not included into the final result

3- mecA gene was detected at query 2007/1662 and was not included into the final result

The genes detected by the PHL but not by the CDC are marked with **red** font color. The genes detected by the CDC but not by the PHL are marked with ***cursive and blue*** font color

Appendix 16. Determination of the Limit of SNP Detection. Results

Below the SNPs detected in the downsampled and original samples are presented. The replicate subjected to downsampling is marked with corresponding coverage values in front of the sequence ID. Only the results for lowest "accurate" coverage and highest "inaccurate" coverage are shown.

Sample C2_2a

Number of SNPs between reference and sample

5x_C2-2a_S3_L001_R1_001 T 1

Original_C2-2a_S3_L001_R1_001 T 1

Sample C5_1

0 SNP difference with the reference at all tested coverages

Sample C6_3a

Number of SNPs between reference and sample

40x_C6_S5_L001_R1_001 CA- -TC GAA -A- 8

50x_C6_S5_L001_R1_001 CAC ATC GAA CAC 12

Original_C6_S5_L001_R1_001 CAC ATC GAA CAC 12

Sample C46_3

Number of SNPs between reference and sample

5x_C46_S10_L001_R1_001 TTT A 4

10x_C46_S10_L001_R1_001 TTT - 3

Original_C46_S10_L001_R1_001 TTT - 3

Sample C52_2

Number of SNPs between reference and sample

5x_C52_S5_L001_R1_001 AC 2

Original_C52_S5_L001_R1_001 AC 2

Sample C72_2

0 SNP difference with the reference at all tested coverages

Sample C73_1c

0 SNP difference with the reference at all tested coverages

Addendum 1. Validation Report for MiSeq Automated Pipeline (MiSeqPipeline Version 1.0 05/11/2016)

A. Scope of report

In the substitute for Core lab bioinformatics pipeline with manual processing of intermediate analysis files, an automated pipeline script was created to process data from MiSeq sequencer. This report details the results of validation of the automated pipeline script.

B. Background

This automated processing pipeline was implemented for the following reasons:

1. Automated pipelines reduce/eliminate monotonous work. This leads to elimination of errors.
2. Automated pipelines allow standardization of several steps for quality assurance purposes.
3. Automated pipeline expedite workflow.

In this validation report, we have validated the user guide for the automated pipeline (installation and usage). We have confirmed the accuracy of the output of the pipeline as measured by comparison of the manual and the automated processes.

C. Validation of installation instructions of automated pipeline

We followed the installation instructions for the automated pipeline script to make sure that the install process was repeatable (SOP: CORE-PROC_WGS_001). We found the installation instructions to be accurate and easy to follow. We noted that the script uses SAMTools, vcfutils, BCFTools, VCFTools, and vcf-tab-to-fasta utilities. We noted that in the original bioinformatics analysis as described in SOP: CORE-PROC_WGS_001, SVAMP was used to convert vcf files to fasta files. In the automated pipeline, vcf-tab-to-fasta utility has been used for the same purpose. This discrepancy was noted and its effect, if any, is discussed in the accuracy validation section.

D. Validation of usage instructions of automated pipeline

The usage instructions for the automated pipeline were followed during the validation process and were found to be accurate. We found that all output from the automated pipeline as seen on the screen were also recorded in timestamped log files.

E. Accuracy of MiSeq Automated Pipeline

We used whole genome sequencing data (BAM files after they were mapped to reference genome) of bacterial isolates from two outbreaks as described in the Assay Validation Report of SOP: CORE-PROC_WGS_001. Accuracy of the MiSeq Automated Pipeline was assessed by obtaining a

phylogenetic tree and comparing it to the previously documented phylogenetic tree in the assay validation report. One of the two outbreaks dealt with gram-positive pathogen. The other outbreak dealt with gram-negative pathogen. BAM files are generated by CLC-bio after mapping sequenced data to a reference genome. These BAM files are the same inputs for both the manual and automated pipelines. The accuracy criteria will be fulfilled if the clustering achieved by the MiSeq automated pipeline matches with the clustering pattern reported in the validation report of the Core Lab (using manual pipeline).

**Study**	**Outbreak 1**<br>SR Harris et al. Lancet Infect Dis 2013; 13: 130–36	**Outbreak 2**<br>P Leekitcharoenphon et al. PLoS ONE 2014; 9(2): e87991
**Microorganism**	Methicillin-resistant *Staphylococcus aureus*	*Salmonella enterica* serovar Typhimurium
**Accession ## of corresponding samples**	ERR128708, ERR124429, ERR124433, ERR070042, ERR070043, ERR070044, ERR070045, ERR070041, ERR072248	ERR277220, ERR277221, ERR277222, ERR277223, ERR277226, ERR277227, ERR277228, ERR277233, ERR277234, ERR277203, ERR277224
**# of clusters in Core Lab validation report tree (manual pipeline)**	1	4
**# of clusters in MiSeq automated pipeline validation tree**	1	4
**# of outbreak isolates in each cluster in the Core Lab validation report tree (manual pipeline)**	Cluster 1= 7	Cluster 1= 2, Cluster 2= 3, Cluster 3= 2, Cluster 4= 2
**# of outbreak isolates in each cluster in MiSeq automated pipeline validation tree**	Cluster 1= 7	Cluster 1= 2, Cluster 2= 3, Cluster 3= 2, Cluster 4= 2
**# of epidemiologically unrelated isolates in the set (manual pipeline)**	2<br>(ERR070041, ERR072248)	2<br>(ERR277203, ERR277224)
**# of epidemiologically unrelated isolates in the set (automated pipeline)**	2<br>(ERR070041, ERR072248)	2<br>(ERR277203, ERR277224)
**# of epidemiologically unrelated isolates clustered with outbreak isolates (manual pipeline)**	0	0
**# of epidemiologically unrelated isolates clustered with outbreak isolates (automated pipeline)**	0	0
**% agreement= (# of outbreak isolates clustered correctly in MiSeq automated pipeline validation tree) x100 / (Total # of outbreak isolates clustered together in the Core Lab validation report tree)**	(7x100/7) = 100%	(9x100/9) = 100%

a. *Clustering pattern is identical for outbreak 1*

The clustering pattern of tree generated by MiSeq automated pipeline is identical to the Core Lab validation tree (manual pipeline). MiSeq automated pipeline uses same bash commands but SVAMP was replaced by vcf-tab-to-fasta utility. We noted that the new utility provided the same results as the older utility SVAMP. The two pictures below show that the results are identical.

Core Lab validation report tree

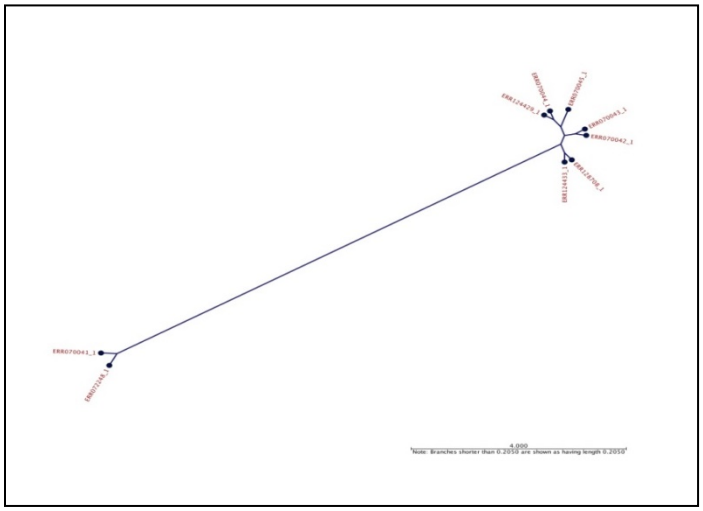

MiSeq automated pipeline validation tree

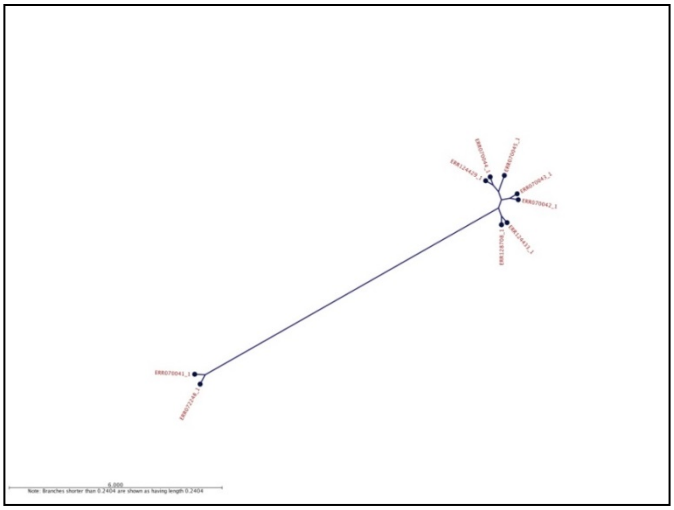

b. *Clustering pattern is identical for outbreak 2*

The clustering pattern of phylogenetic tree for study 2 is identical for both MiSeq automated pipeline and Core Lab validation (manual pipeline). MiSeq automated pipeline uses same bash commands but SVAMP was replaced by vcf-tab-to-fasta utility. We noted that the new utility provided the same results as the older utility SVAMP. The two pictures below show that the results are identical.

Core Lab validation report tree

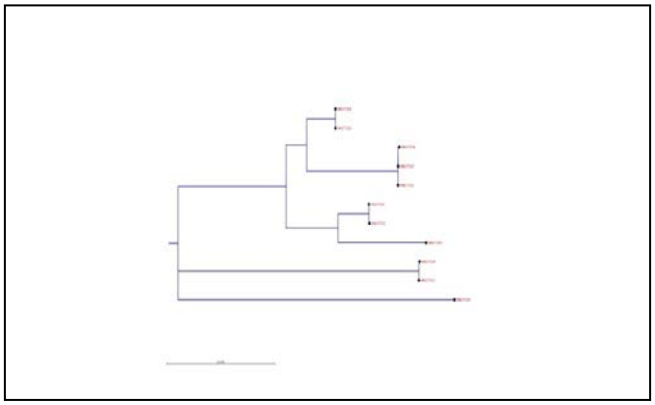

MiSeq automated pipeline validation tree

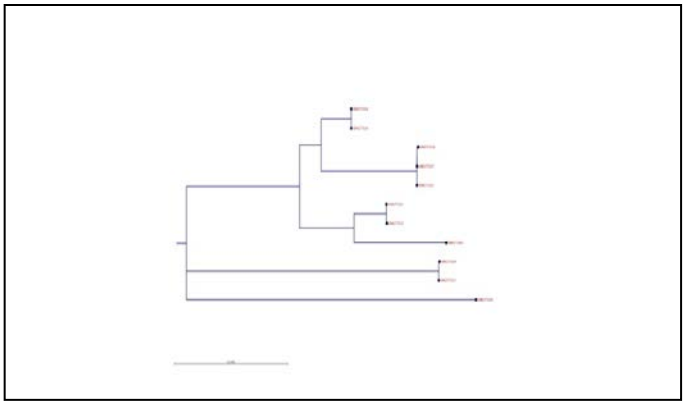

F. Accuracy of MiSeq Automated Pipeline estimated by an Independent Operator

An independent operator performed the analysis for both outbreak 1 and outbreak 2. The results obtained were identical to the original Core Lab validation study. Here are the phylogenetic trees from two outbreaks.

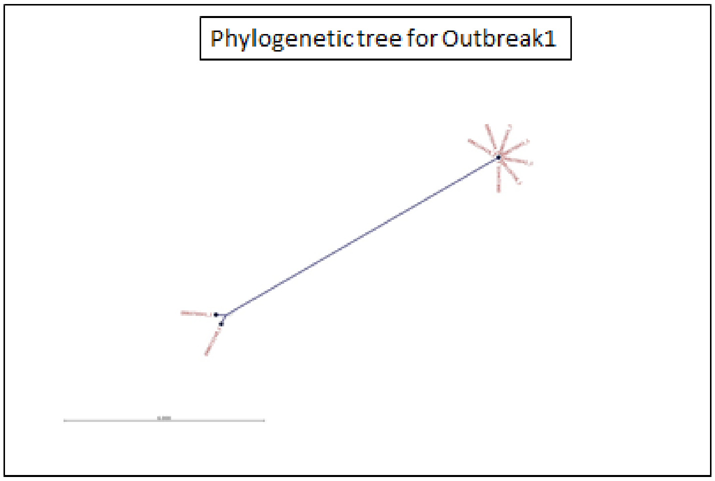

Phylogenetic tree for Outbreak1

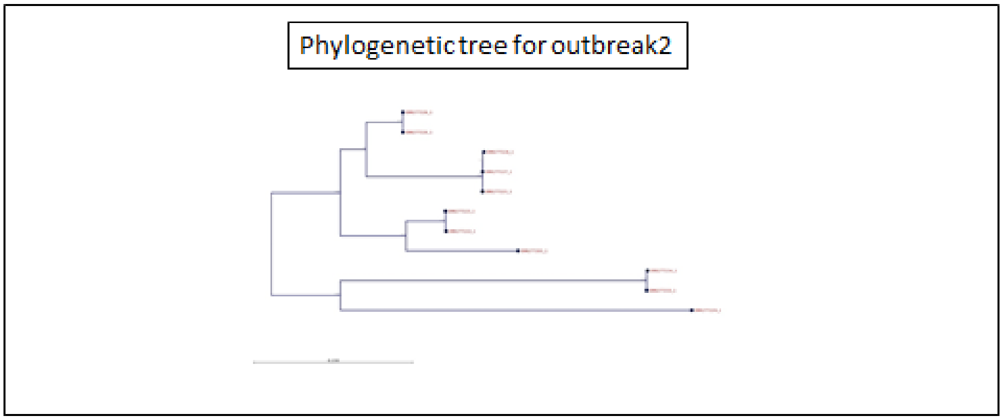

Phylogenetic tree for outbreak2

In conclusion, MiSeq automated pipeline reproduces the same phylogenetic trees for both outbreaks used in the Core Lab validation study. This validation report concludes that the installation instructions and usage instructions of the automated pipeline are accurate. The output of the automated pipeline is also accurate.

Addendum 2. Correlation study for two MiSeq Illumina sequencers

Standard Operating Procedure: Correlation Testing for Whole Genome Sequencing on Multiple MiSeq Instruments

Purpose:

A correlation study is required to ensure that results generated from multiple MiSeq sequencers are equivalent.

Planning and preparation:

Correlation testing must be done every 6 months. Before starting the correlation study, ensure that all instruments have up-to-date maintenance and calibration, and Quality Control results that are within the acceptable range.

Sample selection:

1. A library will be loaded on both instruments at the same time.

   OR:

2. A frozen stored 4nM DNA library, which has completed sequencing on the primary instrument, will be loaded onto the new instrument. Ensure that the chosen library is stored under appropriate conditions. Check quality of the library.

Procedure:

1. Prepare a frozen DNA library starting at the 4nM dilution stage, as stated in the most recent version of the Core Lab SOP. Or, prepare a new library from the DNA extraction stage, as stated in the most recent version of the Core Lab SOP.
2. Load the library at the same concentration as its' correlated counterpart, and use the reagent kit with the same number of cycles.
3. Perform data analysis as stated in the Core Lab SOP. Perform mapping using the same reference for each correlated pair of samples, and generate a SNP pairwise comparison table.

4. Note the following QC measurements from the same run on both instruments:
    a. Read length at which 75% of bases have quality score ≥Q30
    b. Average read length after trimming
    c. Depth of coverage after mapping
    d. Fraction of genome covered after mapping

    For each of the above data sets, calculate the standard deviation.

Acceptability Criteria

For each QC measurement, the standard deviations used for the validation of the primary instrument will be designated as the acceptable limit for correlation testing.

1. Experimental values that are at or below the acceptable limits will be considered passing.
2. The same samples run on different instruments should differ by 0-1 SNPs to be considered as passing.

QC measurement	Standard Deviation Acceptable Limit (values ≤ will pass)
Read length at which 75% of bases have quality score ≥Q30	28.9
Average read length after trimming	13.4
Depth of coverage after mapping	30.5
Fraction of genome covered after mapping	4.3

Whole Genome Sequencing Correlation Report

Note:

The same pooled library was tested on two instruments to ensure that results generated from multiple MiSeq sequencers are equivalent. Samples included in the pooled library were a part of the original validation set of the microorganisms.

Compared instruments:

Primary instrument, serial number M02201

New instrument, serial number M04924

List of isolates:

MDL Core lab ID	Sequence date on Primary instrument M02201	Sequence date on New instrument M04924	Species	Number of replicates within run
C4	04/21/2015	02/08/2017	*Enterobacter cloacae*	1
C72	04/21/2015	02/08/2017	*Escherichia coli*	3
C73	04/21/2015	02/08/2017	*Salmonella enterica* serovar Enteritidis	3
C74	04/21/2015	02/08/2017	*Salmonella enterica* serovar Infantis	3
C75	04/21/2015	02/08/2017	*Salmonella enterica* serovar Adelaide	3
C76	04/21/2015	02/08/2017	*Salmonella enterica* serovar Worthington	3

Consistency of the following quality metrics was estimated:

Read length at which 75% of bases have quality score ≥Q30, bp

Sample	M04924 replicate a	M04924 replicate b	M04924 replicate c	M04924 average	M02201 replicate a	M02201 replicate b	M02201 replicate c	M02201 average	Standard deviation	Standard Deviation Acceptable Limit	Pass/ Fail
C4	190			190	140			140.00	28.9	28.9	Pass
C72	200	160	210	190	155	140	155	150.00	26.0	28.9	Pass
C73	190	160	190	180	150	135	150	145.00	21.4	28.9	Pass
C74	180	150	200	177	145	130	150	141.67	23.7	28.9	Pass
C75	200	160	160	173	150	135	130	138.33	23.1	28.9	Pass
C76	190	180	210	193	150	135	150	145.00	27.5	28.9	Pass

Average read length after trimming, bp

Sample	M04924 replicate a	M04924 replicate b	M04924 replicate c	M04924 average	M02201 replicate a	M02201 replicate b	M02201 replicate c	M02201 average	Standard deviation	Standard Deviation Acceptable Limit	Pass/ Fail
C4	145.1			145.10	130.3			130.30	8.5	13.4	pass
C72	148.7	135.8	146.2	143.57	136	125.2	133.3	131.50	8.0	13.4	pass
C73	150.5	134.8	143.5	142.93	137.8	124.8	134.5	132.37	7.9	13.4	pass
C74	148.3	135.8	149.1	144.40	138.4	126.7	135.6	133.57	7.8	13.4	pass
C75	149.2	135.4	133.8	139.47	135.1	124	122.9	127.33	8.7	13.4	pass
C76	147.7	136.9	145.5	143.37	133.4	125.8	132.8	130.67	7.8	13.4	pass

Depth of coverage, x

Sample	M04924 replicate a	M04924 replicate b	M04924 replicate c	M04924 average	M02201 replicate a	M02201 replicate b	M02201 replicate c	M02201 average	Standard deviation	Standard Deviation Acceptable Limit	Pass/ Fail
C4	52.60			52.60	55.33			55.33	1.58	30.5	Pass
C72	48.98	32.76	50.17	43.97	54.32	39.20	54.21	49.24	7.52	30.5	Pass
C73	57.80	54.98	63.24	58.67	64.16	65.68	75.08	68.31	6.45	30.5	Pass
C74	48.56	35.05	71.38	51.66	62.01	51.80	80.72	64.84	14.40	30.5	Pass
C75	81.87	48.50	41.71	57.36	87.92	54.58	48.50	63.67	16.49	30.5	Pass
C76	51.78	58.81	60.47	57.02	55.24	66.23	65.32	62.26	4.96	30.5	Pass

Fraction of genome covered after mapping

Sample	M04924 replicate a	M04924 replicate b	M04924 replicate c	M04924 average	M02201 replicate a	M02201 replicate b	M02201 replicate c	M02201 average	Standard deviation	Standard Deviation Acceptable Limit	Pass/ Fail
C4	0.98			0.98	0.98			0.98	0	4.3	Pass
C72	0.85	0.85	0.85	0.85	0.86	0.86	0.86	0.86	0	4.3	Pass
C73	0.99	0.99	0.99	0.99	0.99	0.99	0.99	0.99	0	4.3	Pass
C74	0.93	0.93	0.93	0.93	0.97	0.97	0.97	0.97	0	4.3	Pass
C75	0.92	0.92	0.92	0.92	0.92	0.92	0.92	0.92	0	4.3	Pass
C76	0.93	0.93	0.93	0.93	0.93	0.93	0.93	0.93	0	4.3	Pass

Consistency of the base calling between two instruments was estimated:

Distance matrix (pairwise comparison): *Enterobacter cloacae* samples

Value in intersection shows the number of SNPs difference between two isolates.

More similar — More different

		1	2
C4_M02201	1		0
C4_M04924	2	0	

Distance matrix (pairwise comparison): *Escherichia coli* samples
Value in intersection shows the number of SNPs difference between two isolates.
More similar [color scale] More different

		1	2	3	4	5	6
C72-1a_M02201	1		0	0	0	0	0
C72-1a_M04924	2	0		0	0	0	0
C72-1b_M02201	3	0	0		0	0	0
C72-1b_M04924	4	0	0	0		0	0
C72-1c_M02201	5	0	0	0	0		0
C72-1c_M04924	6	0	0	0	0	0	

Distance matrix (pairwise comparison): *Salmonella enterica* serovar Enteritidis samples
Value in intersection shows the number of SNPs difference between two isolates.
More similar [color scale] More different

		1	2	3	4	5	6
C73-1_M02201	1		0	0	0	0	0
C73-1_M04924	2	0		0	0	0	0
C73-2_M02201	3	0	0		0	0	0
C73-2_M04924	4	0	0	0		0	0
C73-3_M02201	5	0	0	0	0		0
C73-3_M04924	6	0	0	0	0	0	

Distance matrix (pairwise comparison): *Salmonella enterica* serovar Infantis samples
Value in intersection shows the number of SNPs difference between two isolates.

More similar [color scale] More different

		1	2	3	4	5	6
C74-1_M02201	1		0	0	0	0	0
C74-1_M04924	2	0		0	0	0	0
C74-2_M02201	3	0	0		0	0	0
C74-2_M04924	4	0	0	0		0	0
C74-3_M02201	5	0	0	0	0		0
C74-3_M04924	6	0	0	0	0	0	

Distance matrix (pairwise comparison): *Salmonella enterica* serovar Adelaide samples

Value in intersection shows the number of SNPs difference between two isolates.

More similar [color scale] More different

		1	2	3	4	5	6
C75-1_M02201	1		0	0	0	0	0
C75-1_M04924	2	0		0	0	0	0
C75-2_M02201	3	0	0		0	0	0
C75-2_M04924	4	0	0	0		0	0
C75-3_M02201	5	0	0	0	0		0
C75-3_M04924	6	0	0	0	0	0	

Distance matrix (pairwise comparison): *Salmonella enterica* serovar Worthington samples

Value in intersection shows the number of SNPs difference between two isolates.

More similar [color scale] More different

		1	2	3	4	5	6
C76-1_M02201	1		0	0	0	0	0
C76-1_M04924	2	0		0	0	0	0
C76-2_M02201	3	0	0		0	0	0
C76-2_M04924	4	0	0	0		0	0
C76-3_M02201	5	0	0	0	0		0
C76-3_M04924	6	0	0	0	0	0	

Summary:

- Four metrics were used to test correlation in the quality of the sequences generated on both instruments. The standard deviations for all samples in all four metrics passed the acceptable limit.
- Quality measurements such as cluster density, Q30 score, and PhiX error rate were not used because they are not unique for each sample in a run.
- For all tested samples, distance matrixes showed 0 SNP difference among the replicates within run or between runs performed on different machines.
- Results generated from MiSeq sequencers M02201 and M04924 are equivalent.

Date of analysis: 2/14/17
Correlation Passed: ☒ Correlation Failed: ☐
Reported by: Date:
Approved by: Date:
